# Blue-Emitting *N*,*O*‑Coordinated Boron Difluoride Complexes with Benzochalcogenazole-Containing Donor–Acceptor Frameworks Featuring Amplified Spontaneous Emission and Delayed Fluorescence

**DOI:** 10.1021/acs.inorgchem.5c05992

**Published:** 2026-04-28

**Authors:** Hanna Zinchenko, Enzo Jean-Woldemar, Andrii Hotynchan, Khrystyna Ivaniuk, Yuliia Sadova, Roman Luboradzki, Paulina H. Marek-Urban, Sébastien Chénais, Pavlo Stakhira, Krzysztof Durka, Sébastien Forget, Mykhaylo A. Potopnyk

**Affiliations:** † Institute of Organic Chemistry, Polish Academy of Sciences, Kasprzaka 44/52, Warsaw 01-224, Poland; ‡ Department of Organic Chemistry, Faculty of Chemistry, 112865 Ivan Franko National University of Lviv, Kyryla i Mefodiya 6, Lviv 79005, Ukraine; § Laboratoire de Physique des Lasers, Université Sorbonne Paris Nord, CNRS, UMR 7538, Villetaneuse F-93430, France; ∥ Department of Electronic Engineering, 226328 Lviv Polytechnic National University, Sviatoho Yura sq. 1, Lviv 79013, Ukraine; ⊥ V. Bakul Institute for Superhard Materials, National Academy of Sciences of Ukraine, Avtozavodska 2, Kyiv 04074, Ukraine; # Institute of Physical Chemistry, 49559 Polish Academy of Sciences, Kasprzaka 44/52, Warsaw 01-224, Poland; g Faculty of Chemistry, 49566 Warsaw University of Technology, Noakowskiego 3, Warsaw 00-664, Poland

## Abstract

Six novel *N*,*O*-coordinated benzochalcogenazole-based boron difluoride complexes (**1a,b–3a,b**) have been synthesized and spectroscopically characterized. The influence of the chalcogen atom (O, S, Se) in the benzochalcogenazole unit on the photophysical properties was systematically investigated. Although the complexes exhibit negligible fluorescence in solution, they display aggregation-induced emission and intense solid-state luminescence, achieving photoluminescence quantum yields of up to 85% in the crystalline state and 69% in poly­(methyl methacrylate) films. The benzoxazole- and benzothiazole-based derivatives exhibit blue amplified spontaneous emission with maxima in the range of 429–455 nm (λ_ex_ = 337 nm), thresholds as low as 12.4 μJ/cm^2^, and full widths at half-maximum as narrow as 9.5 nm. These compounds also display both prompt and delayed fluorescence, indicating efficient exciton utilization. The results demonstrate that rational chalcogen substitution effectively modulates the emission behavior, providing valuable design principles for next-generation organic photonic and optoelectronic materials.

## Graphical abstract

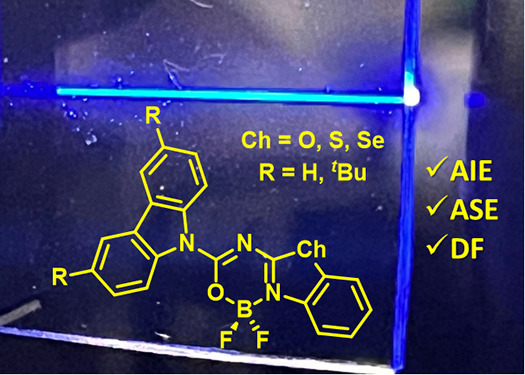

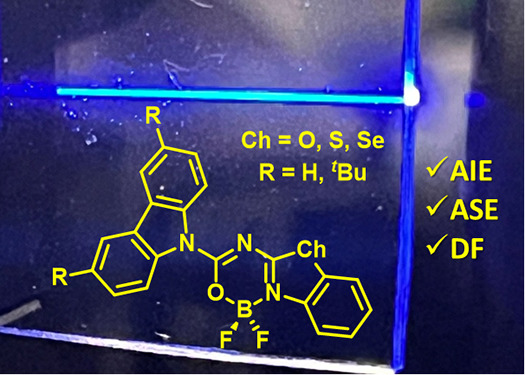

## Introduction

Organic luminescent materials have attracted considerable attention in recent decades due to their potential in optoelectronic and photonic technologies, including organic light-emitting diodes,^1^ organic field-effect transistors,^2^ solid-state lasers,^3,4^ bioimaging probes,^5^ and sensing platforms.^6^ Their appeal lies in the unique advantages offered by organic chromophores, such as lightweight molecular design, synthetic versatility, broad spectral tunability, and the possibility of solution processability for low-cost device fabrication. Among the diverse applications, the realization of organic laser materials with high performance is of particular interest, as such systems promise compact, tunable, and cost-effective coherent light sources.^7^ For practical use, however, organic gain media must simultaneously satisfy stringent requirements, including high photoluminescence quantum yields (PLQYs), narrowband emission, strong resistance to photodegradation, and low thresholds for amplified spontaneous emission (ASE).^8^

Over the years, significant progress has been made in developing molecular systems capable of ASE, with families such as conjugated polymers,^9^ polyaromatic hydrocarbons,^10–14^ perylene diimides,^15^ carbazole-containing styryls,^16–19^ oligophenylenevinylene derivatives,^20,21^ indigo derivatives,^22^ diketopyrrolopyrroles,^23^ phosphorus-containing heterocyclic dyes,^24^ Cibalackrot,^25^ boron dipyrromethene (BODIPY) dyes,^26^ and other boron-containing chromophores^27^ being widely investigated.

Despite these advances, achieving efficient blue ASE remains a formidable challenge. Short-wavelength emitting materials require large optical bandgaps (typically >2.7 eV), which intrinsically impose several constraints at the molecular and device levels.^28^ A wider bandgap often correlates with reduced transition dipole moments and lower oscillator strengths for the lowest-energy singlet transition (S_1_ → S_0_), particularly when strong donor–acceptor interactions are avoided to preserve high emission energy.^29^ In many conjugated systems, extending π-conjugation enhances oscillator strength but simultaneously red-shifts emission, creating a fundamental trade-off between high radiative rates and short-wavelength output.^30^ Moreover, wide-bandgap materials frequently exhibit higher exciton binding energies and lower dielectric screening, which can limit efficient radiative recombination and increase susceptibility to nonradiative decay pathways.^31^

In the solid state, blue-emitting chromophores are especially vulnerable to aggregation-induced quenching. Strong π–π stacking interactions, facilitated by planar backbones and high packing densities, promote the formation of excimers or nonemissive H-aggregates.^32^ These aggregated species introduce additional nonradiative decay channels and spectral broadening, both of which are detrimental to optical gain. Because ASE requires population inversion and stimulated emission to outcompete all loss mechanisms, even modest increases in nonradiative decay rates or reductions in PLQY can dramatically raise the ASE threshold.^33^ Furthermore, triplet-state accumulation under high excitation fluence can lead to singlet–triplet annihilation and triplet–triplet absorption, processes that are particularly problematic in wide-bandgap systems with long-lived triplet states.^34^

Collectively, these factors – limited oscillator strength at large bandgaps, aggregation-induced quenching, reabsorption losses, exciton annihilation processes, and insufficient photostability – converge to hinder the realization of low-threshold, efficient blue ASE. Therefore, the rational molecular design of wide-bandgap chromophores with high radiative decay rates, suppressed intermolecular interactions, large effective Stokes shifts, balanced excited-state dynamics, and robust chemical stability remains a critical unmet need in organic photonics and the development of next-generation blue organic laser and ASE-active devices.^35–41^

In this context, boron difluoride (BF_2_) complexes represent an important class of luminescent materials that have shown remarkable potential for ASE applications.^41–48^ Their structural rigidity, strong electron-withdrawing nature of the boron center, and versatile coordination chemistry enable efficient stabilization of π-conjugated frameworks, thereby enhancing fluorescence efficiency and color purity.^49^ Among these, BODIPY dyes have been studied extensively and are recognized for their high PLQYs and narrowband emission.^50^ However, they typically emit in the green-to-red spectral range, and extensive chemical modification is often required to access the blue region.

To overcome these limitations, alternative BF_2_ systems incorporating *N*,*N*-, *N*,*O*-, or *O*,*O*-chelating ligands have emerged as promising scaffolds for modulating emission wavelength, photostability, and solid-state properties.^51–53^ In particular, *N*,*O*-chelated boron difluoride complexes offer a powerful molecular design strategy, providing enhanced structural rigidity, stabilized frontier molecular orbitals, and tunable donor–acceptor (D–A) electronic interactions.^54–59^ By judiciously engineering the donor and acceptor units, controlled intramolecular charge transfer (ICT) can be achieved, enabling fine color tuning across the visible spectrum and access to the challenging blue region, while also optimizing excited-state lifetimes and emission efficiency.^60,61^

On the other hand, the incorporation of heavy atoms – particularly heavier chalcogens – into boron difluoride dyes is currently attracting significant attention.^62,63^ Heavy atoms enhance spin–orbit coupling (SOC), which promotes delayed fluorescence, offering distinct advantages for optoelectronic applications.^64^

In this regard, heteroaromatic building blocks such as benzochalcogenazoles have emerged as versatile building blocks for constructing D–A chromophores.^65^ The benzochalcogenazole motif, incorporating oxygen, sulfur, or selenium atoms, offers strong electronic tunability owing to the varying electronegativity, polarizability, and heavy-atom effects across the chalcogen series.^66^ When integrated into D–A architectures, these heterocycles enable precise control over orbital localization and ICT,^67^ which are key parameters governing emission color, quantum efficiency, and ASE behavior. Despite these advantages, systematic studies on benzochalcogenazole-based BF_2_ complexes as ASE-active materials – particularly in the blue regime – remain scarce.

Recently, we developed a series of benzochalcogenazole-containing boron difluoride complexes that exhibit locally excited emission characteristics (compounds **Ph-ONB-bCh**, Figure 1a).^68^ These complexes display chalcogen-dependent tunable absorption and photoluminescence with moderate PLQYs in a polymer matrix and demonstrate promising potential for lasing applications. Structural modification of this molecular scaffold through the incorporation of electron-donating groups can provide additional advantages, such as enabling thermally activated delayed fluorescence (TADF).

**Figure 1:**
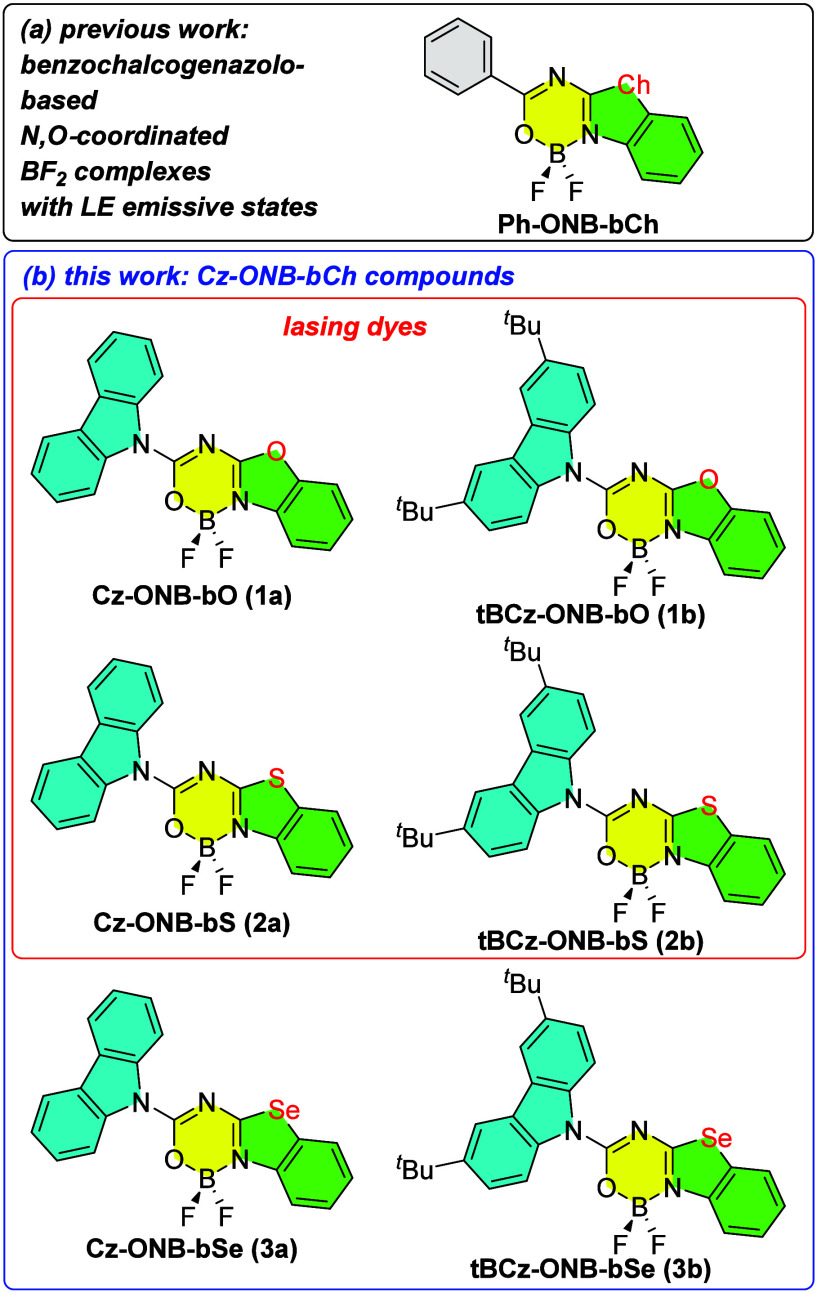
(a) Previously reported dyes. (b) Benzochalcogenazolo-based boron difluoride complexes **1a,b–3a,b**.

Herein, we present a new carbazole-containing class of blue donor–acceptor benzochalcogenazolo-based *N*,*O*-coordinated boron difluoride complexes **Cz-ONB-bCh** (**1a,b–3a,b**, Figure 1b) that combine efficient emission with remarkably low ASE thresholds. Through rational molecular design, we demonstrate that tuning the D–A interactions and exploiting heteroatomic coordination enables the construction of highly emissive and structurally rigid chromophores. Detailed photophysical characterization reveals strong solid-state fluorescence with high PLQYs, blue emission, and excellent ASE performance. Importantly, these complexes exhibit significantly reduced ASE thresholds compared to many previously reported blue-emitting BF_2_ systems, highlighting the potential of benzochalcogenazole scaffolds for organic laser applications. Complementary computational studies further provide insights into the structure–property relationships governing their emission and ASE characteristics.

Overall, this work establishes benzochalcogenazolo-based BF_2_ complexes as a promising platform for the development of deep-blue organic photonic materials. By addressing the longstanding challenge of low-threshold ASE in the blue spectral region, our findings open new avenues for the rational design of high-performance chromophores suitable for integration into next-generation optoelectronic and laser technologies.

## Results and Discussion

### Synthesis

The synthesis of compounds **1a,b–3a,b** (Scheme 1) was started from carbazole (**4a**) and di-*tert*-butyl carbazole (**4b**) (Scheme 1). Compounds **4a,b** were treated with triphosgene at −30 °C in dichloromethane with the presence of catalytic amount of pyridine to give 9*H*-carbazole-9-carbonyl chloride (**5a**) and 3,6-di-*tert*-butyl-9*H*-carbazole-9-carbonyl chloride (**5b**) in high yield (86–89%). The resulting carbamoyl chlorides **5a,b** were converted into corresponding isothiocyanates **6a,b** by reaction with potassium thiocyanate in hot acetone. Intermediate compounds **6a,b** were then in situ introduced in the reactions with anilines **7–9**. The reaction of isothiocyanates **6a,b** with 2-aminophenol (**7**) afforded *N*-((2-hydroxyphenyl)­carbamothioyl)-9*H*-carbazole-9-carboxamide (**10a**) and 3,6-di-*tert*-butyl-*N*-((2-hydroxyphenyl)­carbamothioyl)-9*H*-carbazole-9-carboxamide (**10b**) with the yields of 60% and 72%, respectively. Analogical reactions of isothiocyanates **6a,b** with 2-aminobenzenethiol (**8**) or 2,2’-diselenediyldianiline (**9**) gave the intermediates **11a,b** and **12a,b**, which spontaneously underwent cyclization to benzothiazole or benzoselenazole rings giving amides **15a,b** and **16a,b** with good yields (43–74%). To obtain the benzoxazole analogues **14a,b**, thioureas **10a,b** were treated with Burgess reagent [methyl *N*-(triethylammoniumsulfonyl)­carbamate, **13**] in hot toluene to give products **14a,b** in 79–80% yield. Finally, amides **14a,b**, **15a,b**, and **16a,b** were treated with boron trifluoride etherate (BF_3_·OEt_2_) in the presence of *N*,*N*-diisopropylethylamine (DIPEA) yielding final oxadiazaborinines **1a,b**, **2a,b**, and **3a,b**. Reaction conditions for the synthesis of compound **2b** were optimized by varying the BF_3_·OEt_2_/DIPEA ratio and temperature, using different solvents (Table S1 in the Supporting Information, SI). The optimal yield (86%) was obtained using six equivalents of BF_3_·OEt_2_ and three equivalents of DIPEA at 100 °C for 4 h. These optimized conditions were subsequently applied to amides **14a,b**, **15a**, and **16a,b**, affording boron complexes **1a,b, 2a, and 3a,b** in 44–76% yield. The synthesized boron dyes and their precursors were fully characterized by ^1^H, ^13^C, ^19^F, ^77^Se NMR spectroscopy and high-resolution mass spectrometry (HRMS). Due to the limited intramolecular rotation between the carbazole and oxadiazaborinine units, some signals in the spectra measured at room temperature were broadened. Therefore, to clarify these spectra, they were also recorded at −40 °C.

**Scheme 1:**
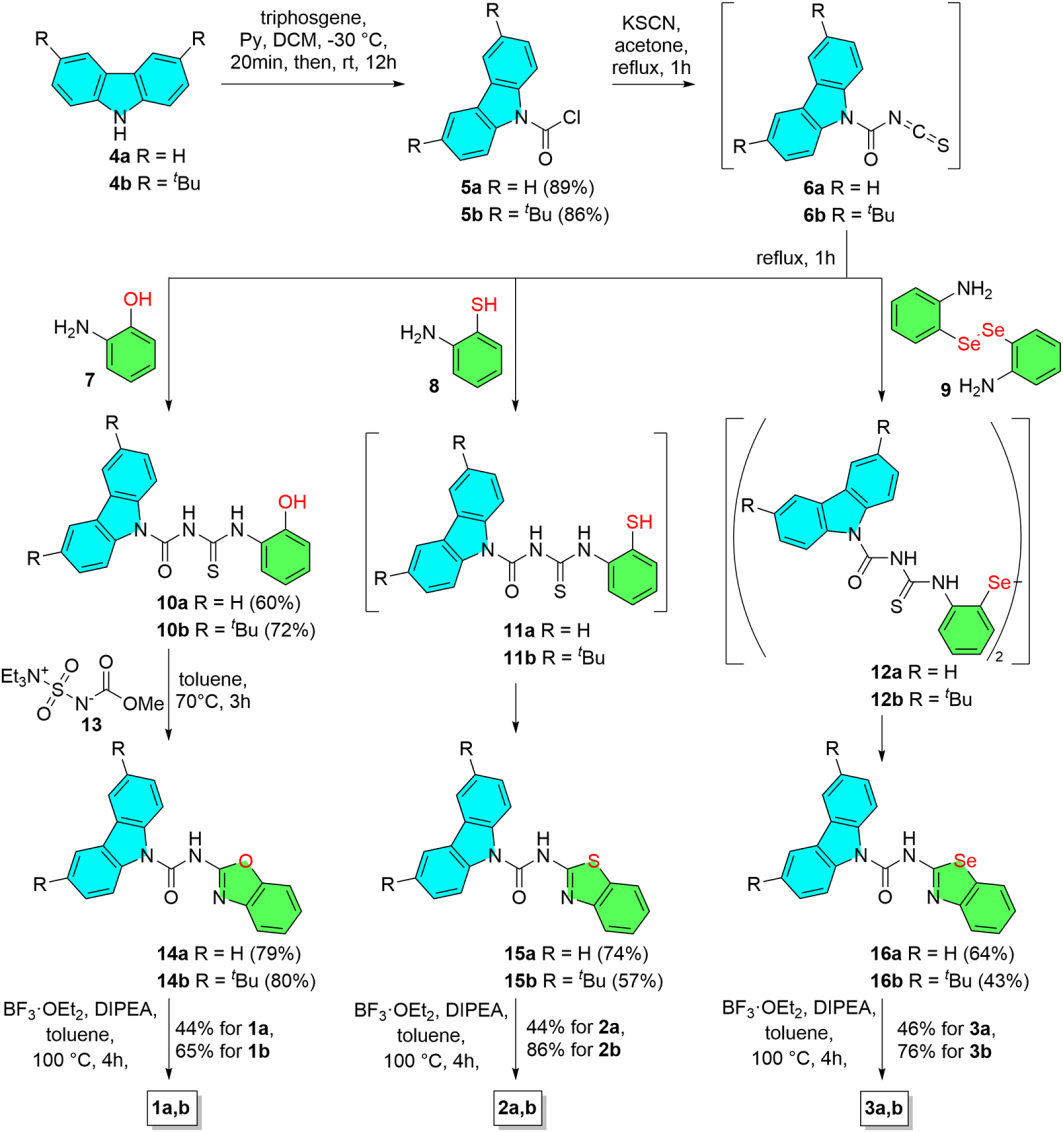
Synthesis of Boron Difluoride Complexes **1a,b–3a,b**

### Crystal Structure Analysis

The structures of compounds **1a,b, 2a, and 3a,b** were confirmed by single-crystal X-ray diffraction analysis (Figures S1–S5 and Tables S2–S6 in the SI). The analysis of molecular geometries of all five structures revealed that the carbazole unit remains nearly coplanar with the heterocyclic units, as indicated by the small torsion angles C1–N1–C13-N3 (Θ/Θ_A_ = 2.6°–6.1°, Figures 2a,d,g, 3a; Figure S6a and Table S7 in the SI). This near-to-planar conformation is stabilized by intramolecular C–H···N and C–H···O interactions (2.22–2.35 Å; Figures 2a,d,g and 3a; Figure S6a in the SI) between the carbazole and oxadiazaborinine rings. Furthermore, a pronounced trend is observed in the C15–Ch–C14 angle (Ch = chalcogen) of the benzochalcogenazole framework: this angle reduces markedly from 105.2–105.3° in the benzoxazole derivatives to 90.3° in the benzothiazole analogue and 85.7–86.0° in the benzoselenazole derivatives (Table S7 in the SI), reflecting the increasing covalent radii and decreasing (X)­np-π­(C) (*n* = 2 for X = O, *n* = 3 for X = S, *n* = 4 for X = Se) orbital overlap of the heavier chalcogens.

**Figure 2:**
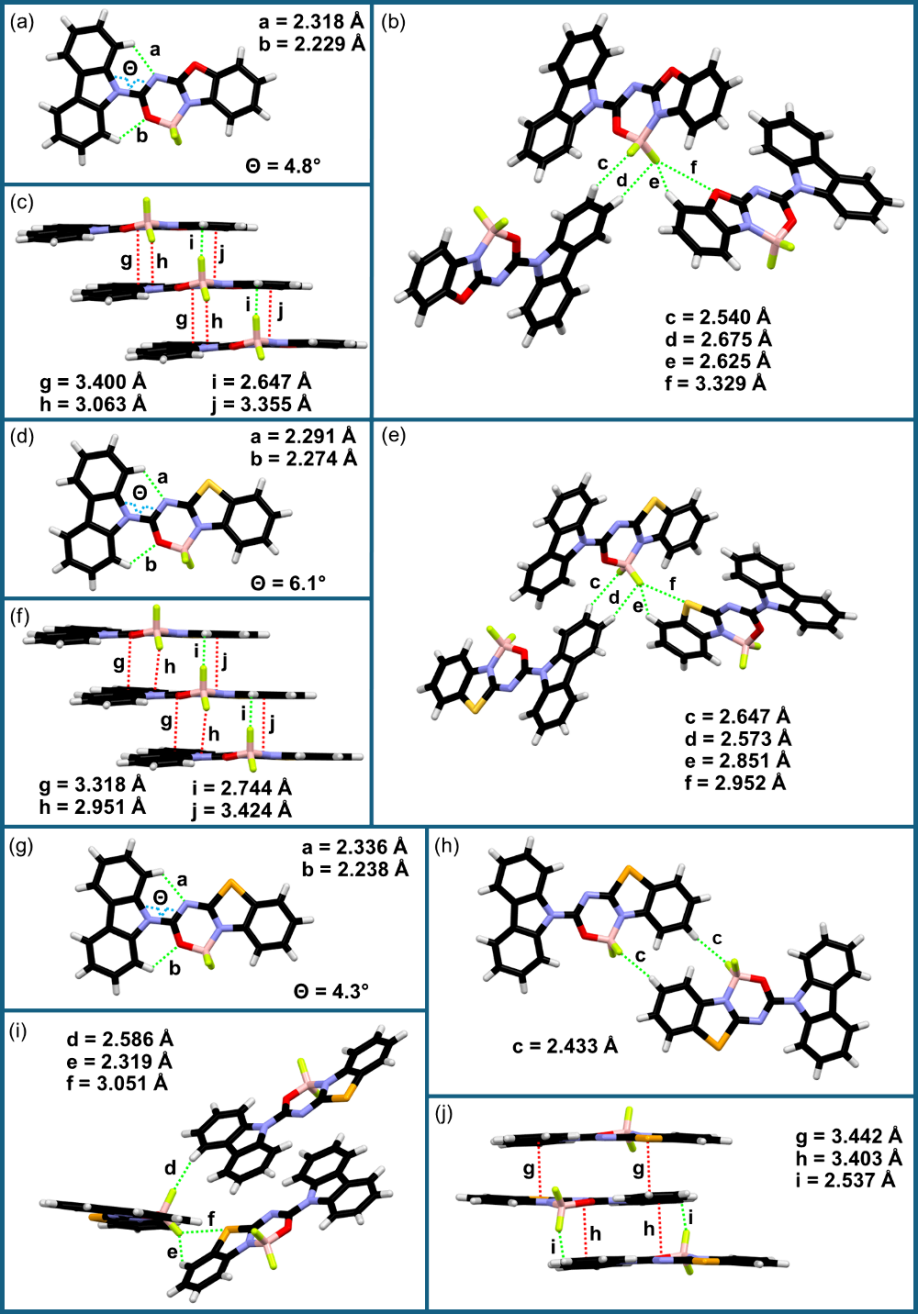
X-ray molecular structure of dyes **1a** (a), **2a** (d), and **3a** (g), showing intramolecular C–H···N and C–H···O hydrogen bonds (green dashed lines). Fragments of the crystal packing showing: intermolecular C–H···F hydrogen bonds and F···Ch interactions (green dashed lines) of structures **1a** (b), **2a** (e), **3a** (h, i); intermolecular C­(π)···C­(π)/n···C­(π) interactions (red dashed lines) and C–H···F hydrogen bonds (green dashed lines) of structures **1a** (c), **2a** (f), **3a** (j).

Boron complexes **1a** and **2a** are isostructural and crystallize in the orthorhombic crystal system with *Pna*2_1_ space group with four molecules in the unit cell (Figure S7 and S8 in the SI). Boron complex **3a** crystallizes in the monoclinic *P*2_1_/c space group also with four molecules in the unit cell (Figure S9 in the SI). Crystal packings of BF_2_ complexes **1a–3a** are governed by multiple C–H···F hydrogen bonds, as well as C–H···C­(π) and C­(π)···C­(π)/n···C­(π) interactions, and σ-hole F···Ch interactions (Figures 2b,c,e,f). Specifically, in structures **1a** and **2a**, the molecules assemble into π-stacking slipped columnar motif propagating along [001] crystallographic direction, with adjacent molecules shifted relative to each other to enhance overlap between electron-rich carbazole and electron-deficient diazaoxaborinine fragments. In case of structure **3a**, the π-stacking columns are also observed, however, their formation involves antiparallel dimers held by relatively short C­(π)···C­(π)/n···C­(π) interactions between overlapping pairs of carbazole and benzoselenazole fragments. These dimers are further connected with each other through weaker π-stacking interactions formed between carbazole and diazaoxaborinine units. The distance between root-mean-square planes of stacked molecules is 3.357 Å for compound **1a**, 3.352 Å for **2a**, and 3.450 Å and 3.532 Å for **3a**. In all three structures, the π-stacking columns are interconnected via C–H···F and C–H···C­(π) interactions, further supported by σ-hole F···Ch interactions with *d*_F···S_ = 2.952(1) Å for benzothiazole derivative **2a** and *d*_F···Se_ = 3.051(1) Å for benzoselenazole analogue **3a**, resulting in 3D supramolecular network.

In case of compounds **1b** and **3b** (Figure S10 and S11 in the SI), the *tert*-butyl groups significantly influence the molecular packing, reducing C­(π)···C­(π)/n···C­(π) overlap and participating in C–H···F and C–H···C­(π) interactions. They crystallize in the monoclinic *P*2_1_/n and triclinic *P-1* space groups of symmetry, respectively. Structure **3b** contains two distinct conformers in the asymmetric part of the unit cell. The molecules in both crystal structures are linked through multiple hydrogen bonds (C–H···F and C–H···O/C–H···Se, Figures 3a–c; Figures S6b,c in the SI) accompanied by some C­(π)···C­(π)/n···C­(π) interactions (Figure 3c; Figure S6d in the SI). As in the case of analogues **1a–3a**, the columnar motifs can be distinguished. However, due to the presence of sterically bulky *tert*-butyl group, the interplanar distance between molecules increases to 3.469–3.728 Å for compound **1b** and 3.571–3.712 Å for benzoselenazole analogue **3b**.

**Figure 3:**
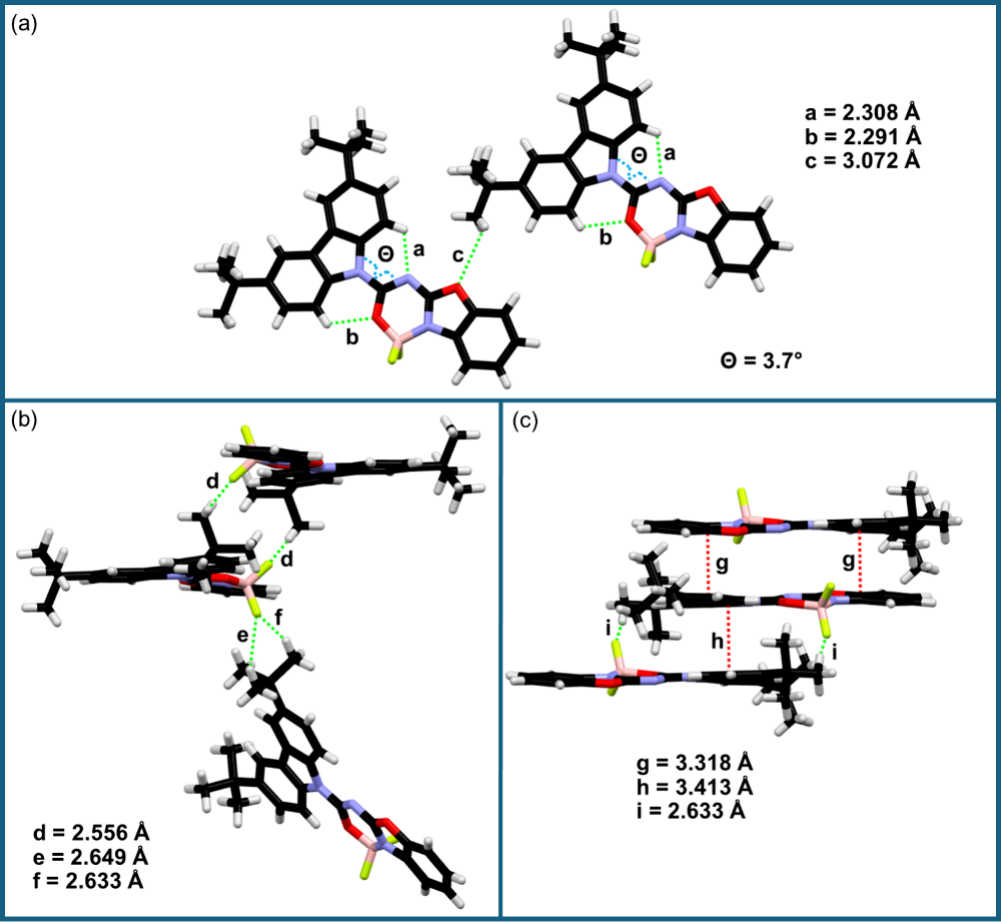
X-ray molecular structure of dyes **1b**. Fragments of the crystal packing showing: intramolecular C–H···N and C–H···O hydrogen bonds and intermolecular C–H···O hydrogen bonds (green dashed lines) (a); intermolecular C–H···F hydrogen bonds (green dashed lines) (b); intermolecular C–H···F hydrogen bonds (green dashed lines) and C­(π)···C­(π)/n···C­(π) interactions (red dashed lines) (c).

### Quantum Chemical Calculations

To elucidate the electronic structures of dyes **1a,b–3a,b**, density functional theory (DFT) calculations were carried out. All geometries were optimized at the B3LYP/6–31G­(d) level of theory, with an additional SDD basis set applied for the selenium-containing derivatives **3a,b**. For molecules **1a**, **2a**, and **3a**, the optimized structures are nearly planar, exhibiting torsion angles of 0.16°, 0.38°, and 0.02°, respectively, between the carbazole and oxadiazaborinine fragments. This correlates well with the crystallographic parameters. The highest occupied molecular orbitals (HOMOs) and lowest unoccupied molecular orbitals (LUMOs) are delocalized across the entire π-system; however, the HOMO density is significantly shifted toward the carbazole unit, while the LUMO density is more localized on the oxadiazaborinine scaffold, indicating a slight ICT character of the compounds. The corresponding HOMO–LUMO energy gaps are 4.35, 4.13, and 4.09 eV. Substitution of oxygen with sulfur or selenium decreases the LUMO energy and increases the HOMO energy, while the overall band gap remains largely unchanged (Figure 4). *tert*-Butyl derivatives **1b**, **2b**, and **3b** exhibit similarly planar geometries (torsion angles of 0.13°, 0.48°, and 0.09°). Their HOMO–LUMO gaps are slightly reduced to 4.23, 4.00, and 3.98 eV, respectively – owing to the enhanced electron-donating character of the di-*tert*-butylcarbazole unit relative to unsubstituted carbazole (Figure 4).

**Figure 4:**
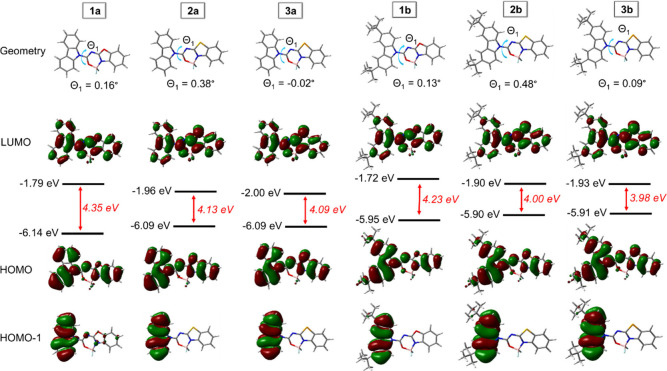
Optimized ground-state geometries and frontier molecular orbitals (HOMO–1, HOMO, and LUMO) of compounds **1a,b**, **2a,b**, and **3a,b**.

Time-dependent DFT (TD-DFT) calculations at the same level of theory were performed to analyze the electronic transitions (Table 1). For compound **1a**, two intense bands at 330 and 329 nm correspond to the S_0_→S_1_ and S_0_→S_2_ transitions with oscillator strengths of 0.5972 and 0.4446, attributed to HOMO→LUMO and HOMO–1→LUMO excitations. The first transition (87% HOMO→LUMO, 11.5% HOMO–1→LUMO) has a locally excited (LE) character, while the second (11.4% and 86.9%) exhibits charge-transfer (CT) nature. For compounds **2a** and **3a**, single strong absorptions at 348 and 352 nm (oscillator strengths 0.9620 and 0.9521) arise from HOMO→LUMO excitations of predominant LE character (98.5% and 98.7%, respectively).

**Table 1:** Calculated Absorption Properties of **1a,b–3a,b**

Dye	Excited state	E, eV	λ, nm	f	Nature
**1a**	S_1_	3.76	330	0.5972	LE
	S_2_	3.78	329	0.4446	LE+CT
**2a**	S_1_	3.56	348	0.9620	LE
**3a**	S_1_	3.52	352	0.9521	LE
**1b**	S_1_	3.68	337	1.0823	LE
**2b**	S_1_	3.47	357	1.0615	LE
**3b**	S_1_	3.44	360	1.0494	LE

Similar behavior is observed for *tert*-butyl analogues **1b–3b**, where the strongest absorption corresponds to the S_1_ state (LE, 97.8–98.6% contribution). The *tert*-butyl groups induce a bathochromic shift and enhance oscillator strengths, leading to higher molar absorptivity and PLQY values. The computed values are in well agreement with the experimental spectra (Figures S21–S26 in the SI).

To gain further insight into the photophysical properties, excited-state calculations and natural transition orbital (NTO) analysis were performed using LC-ωPBE/6–31+G­(d,p) method. In the first singlet excited state, compounds **1a–3a** adopt a twisted configuration (≈ 90° rotation of the carbazole unit). NTO analysis indicates that the highest occupied natural transition orbital (HONTO) resides on the carbazole fragment and the lowest unoccupied natural transition orbital (LUNTO) on the diazaoxaborinine unit (Figure S13–S15 in the SI). Consequently, upon excitation, a twisted intramolecular charge-transfer (TICT) with decoupled donor and acceptor fragments can be formed.^69,70^ The first two triplet states of boron complexes **1a** and **2a** exhibit LE character, with both HONTO and LUNTO localized on the diazaoxaborinine moiety (T_1_) or carbazole unit (T_2_). For benzoselenazole-containing compound **3a**, both triplets also display LE character, but the orbital localization is reversed. The *tert*-butyl analogues (**1b–3b**) show similar behavior: the first singlet states are of CT type, while both triplets possess LE character. In compounds **1b** and **2b**, the T_1_ orbitals are localized on the diazaoxaborinine part and T_2_ on the carbazole; in selenium derivative **3b**, this order is inverted (Figure S16–S18 in the SI).

The S_1_–T_1_ singlet–triplet energy gaps are 0.67, 0.81, and 0.79 eV for boron difluoride complexes **1a–3a**, and 0.40, 0.58, and 0.65 eV for compounds **1b–3b**; the S_1_-T_2_ gaps are 0.39–0.70 eV for **1a–3a**, and 0.27–0.54 eV for **1b–3b**, respectively (Table S9 in the SI). In order to further conclude on the S-T intersystem crossing, spin–orbit coupling matrix elements (SOCME) were computed in Orca 6.0 using the PBE0/def2-TZVP method (Table S10 in the SI). For compounds **1a**, **2a**, and **3a**, SOCME values between S_1_–T_1_ are 0.03, 0.05, and 1.25 cm^–1^, and between S_1_–T_2_ are 0.02, 0.07, and 0.16 cm^–1^, respectively. These indicate negligible ISC for dyes **1a** and **2a**, whereas compound **3a** shows enhanced coupling due to the heavy-atom effect. For boron complexes **1b**, **2b**, and **3b**, SOCME values between S_1_–T_1_ are 0.01, 0.15, and 1.63 cm^–1^, and between S_1_–T_2_ 0.03, 0.04, and 0.15 cm^–1^, respectively, indicating that *tert*-butyl substitution increases SOC for compounds **2b** and **3b**. Overall, the S_1_–T_1_ and S_1_–T_2_ energy gaps and SOCME values indicate the possibility for S_1_–T_1_ intersystem crossing for Se-containing **3a** and **3b** difluoroboron complexes. However, the reverse process (RISC) is rather unlikely due to the high singlet–triplet energy gap, precluding the occurrence of TADF.

### Photophysical Properties in the Solutions and the Solid State

In toluene solution, benzoxazole-containing boron difluoride complexes **1a** and **1b** exhibit broad lowest-energy absorption peak maximized at 329 and 334 nm, respectively, with a bathochromic shoulder. In contrast, benzothiazole- and benzoselenazole-containing analogues **2a**, **2b**, **3a**, and **3b** show double-maximum peak at 347/357 nm, 351/363 nm, 354/364 nm, 357/369 nm, respectively. In all cases, the respective absorption bands are independent of the solvent polarity (Table S11 in the SI), which is agreement with LE character of these transitions. The molar absorption coefficient of the dyes is in the range 4.5–6.0 × 10^4^ M^–1^·cm^–1^ (Figure 5a, Table S11 in the SI). The progressive bathochromic shift of absorption maxima across the benzoxazole → benzothiazole → benzoselenazole series (**1a–3a** and **1b–3b**) is consistent with the expected electronic effects of chalcogen atom.

**Figure 5:**
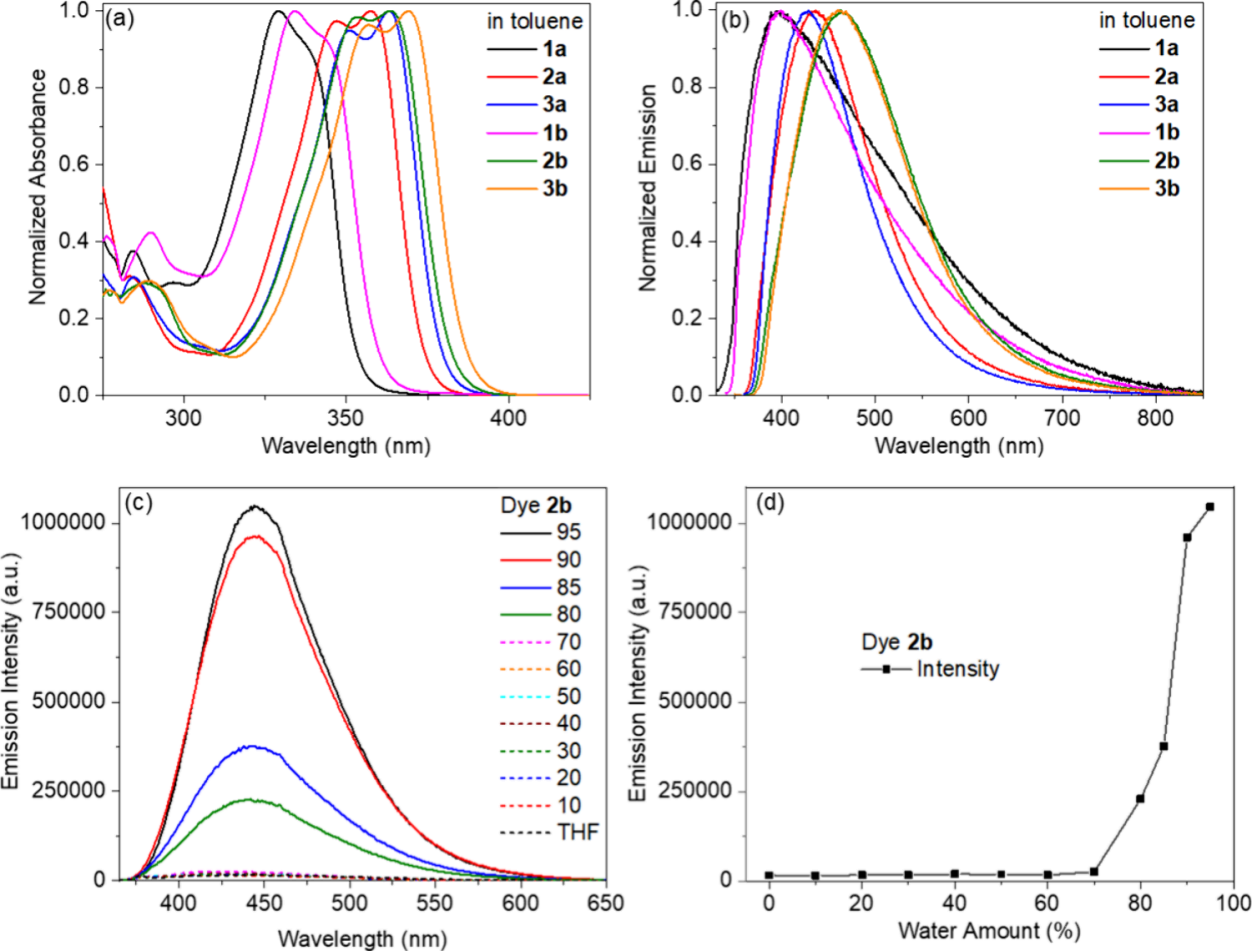
Absorption (a) and photoluminescence (b) spectra of solutions of boron dyes **1a,b**, **2a,b**, and **3a,b** in toluene. (c) Photoluminescence spectra of the dispersions of compound **2b** in THF/water mixtures of varying water contents. *C* = 5.0 × 10^–6^ M, λ_ex_ = 340 nm. (d) Plot of emission intensity of dye **2b** versus f_w_.

The investigated compounds display broad photoluminescence spectra in toluene (Figure 5b). Benzoxazole derivatives **1a** and **1b** exhibit nearly identical emission spectra, with maxima (λ_em_) at 398–399 nm. In contrast, the corresponding benzothiazole and benzoselenazole analogues display a pronounced bathochromic shift, with λ_em_ values of 436 and 464 nm for **2a** and **2b**, respectively, and 428 and 462 nm for compounds **3a** and **3b**, respectively (Figure 5b; Table S11 in the SI). PLQYs in toluene are generally low (<0.1–4% for **1a,b**, **2b**, and **3a,b**), with the notable exception of the benzothiazole derivative **2a**, which exhibits a PLQY of 12% (Table S11 in the SI). Some shifts of the emission bands are observed in solvents of varying polarity (toluene, CH_2_Cl_2_, THF), but without a clear positive solvatochromic trend. In turn, PLQY gradually decreases with increasing of the solvent polarity and it is completely quenched in MeCN (Figures S19–S20, Table S11 in the SI). These observations suggest that, upon excitation, the molecules either relax directly from the ICT state or, as suggested by quantum-chemical calculations, populate a nonemissive TICT state. Regardless, low PLQY results from the conformational lability of the carbazole unit.

The aggregation-induced emission (AIE) behavior of all complexes was investigated in THF/water mixtures with varying water content. Thus, dye **2b** displays negligible emission in pure THF and in mixtures containing up to 80% water (Figures 5c,d). At water fractions ≥ 80%, however, the PL intensity increases sharply, reaching a maximum at 95% water with *a* > 60-fold enhancement. Analogous behavior is observed for **1a,b**, **2a**, and **3a,b** (Figure S27 and S28 in the SI).

In the subsequent step, we investigated photophysical properties of the compounds in the crystalline solid-state. The complexes exhibit emission maxima at 386 nm (dye **1a**), 440 nm (dye **2a**), 438 nm (dye **3a**), 404 nm (dye **1b**), 420 nm (dye **2b**), and 431 nm (dye **3b**) (Figure S29, Table S12 in the SI). The positions of the emission bands are generally preserved from the toluene solutions, with the exception of **2b** and **3b**, which showed hypsochromic shift in the solid state. Interestingly, benzoxazole-containing boron complexes display markedly enhanced PLQYs (42% for dye **1a** and 85% for **1b**), attributable to suppression of carbazole rotational freedom, which reduces carbazole–benzochalcogenazole π-conjugation. In contrast, benzoselenazole-containing analogues (**3a, 3b**) exhibit significantly lower efficiencies (∼1%), consistent with the heavy-atom effect of selenium that facilitates ISC and further nonradiative decay. Surprisingly, a very different situation is observed for benzothiazole-containing dyes: compound **2a** demonstrated low PLQY value (∼2%), while *tert*-butyl analogue **2b** exhibited much intensive photoluminescence (PLQY = 57%). The comparison of **1a**/**2a** with their **1b**/**2b** counterparts clearly shows the role of bulky *tert*-butyl groups, in suppressing the π-stacking interactions in the solid-state promoting emission behavior.

Interestingly, isostructural benzoxazole **1a** and benzothiazole **2a** difluoroboron complexes exhibit very different photophysical properties. This is reflected in their emission color, which changes from λ_PL_ = 386 nm for **1a** to λ_PL_ = 440 nm for **2a**, and strong fluorescence quenching for **2a** (PLQY = 2%) relative to **1a** (PLQY = 42%). As discussed later, both compounds display very similar emission patterns in PMMA films (λ_PL_ = 397 nm for **1a** and λ_PL_ = 400 nm for **2a**) with significantly enhanced PLQY for **2a** (69%) and comparable values between PMMA film (35%) and bulk solid-state for **1a**. Comparison of the crystal structures of **1a** and **2a** revealed subtle differences in their intermolecular interactions. The interplanar distances in the π-stacking dimers are 3.298 Å for **1a** and 3.438 Å for **2a.** Additionally, **2a** displays higher in-plane shift with respect to **1a** (Figure S12, SI). Altogether this leads to the less efficient π-stacking interactions in **2a**, thus basing on the sole comparison of π-stacking motifs it could be expected that **2a** should be better emitter than **1a**, which does not agree with our observations. Another important difference between both structures relies in the chalcogen interaction, which is present for **2a** (*d*_S···F_ = 2.952(2) Å) and absent for **1a** (*d*_O···F_ = 3.329(2) Å). The σ-hole interaction increases the electron density at benzothiazole ring, elevating LUMO and reducing intramolecular charge transfer. Thus, hypsochromic shift of the emission band could be observed, which again contradicts the observed trend. There can be also other structural factors such as different polarity of crystal environments, however the origin of the observed differences between solid-state samples remains unclear. It should be also noted that emission maximum of *tert*-butyl analogue **2b** (λ_PL_ = 420 nm) is red-shifted compared to **1b** (λ_PL_ = 404 nm), and the PLQY decreases from 85% (**1b**) to 57% (**2b**).

Time-resolved photoluminescence measurements reveal short nanosecond lifetimes of 0.2–2.8 ns. Interestingly, all complexes also display microsecond components, spanning a wide range: short-lived for **1a,b**, **2b**, and **3a,b** (1.02–5.95 μs), and much longer for **2a** (68.8 μs), which again points that the delayed fluorescence emission is less effective in the later system. Finally, given the large singlet–triplet energy gap, it is unlikely that observed delayed fluorescence (DF) arises from TADF, but rather results from another process, such as triplet–triplet annihilation (TTA). Such behavior was observed in other boron complexes.^71,72^

### Photophysical Properties of the Molecular Dispersions in the Polymer Matrix

In order to obtain comprehensive information regarding properties of boron complexes **1a,b**, **2a,b**, and **3a,b** photophysical behavior was also investigated in polymer matrix. Poly­(methyl methacrylate) (PMMA) was chosen as the suitable polymer owing to its transparency and exceptional film-forming capabilities. The ratio between dye and PMMA was chosen 5:95 in the case of compounds **1a,b**, **2b**, and **3a,b**, and 2:98 for low-soluble boron difluoride complex **2a**. The obtained results reveal that absorption and emission spectra shapes are quite similar to the toluene solution ones. Specifically, benzoxazole derivatives **1a** and **1b** exhibit absorption maxima centered at 327 and 333 nm, respectively. While benzothiazole- and benzoselenazole-containing analogues **2a**, **2b**, **3a**, and **3b** exhibit bathochromically shifted double-peaks maximized at 347/354, 352/359, 350/360, and 354/363 nm, respectively (Figure 6a).

**Figure 6:**
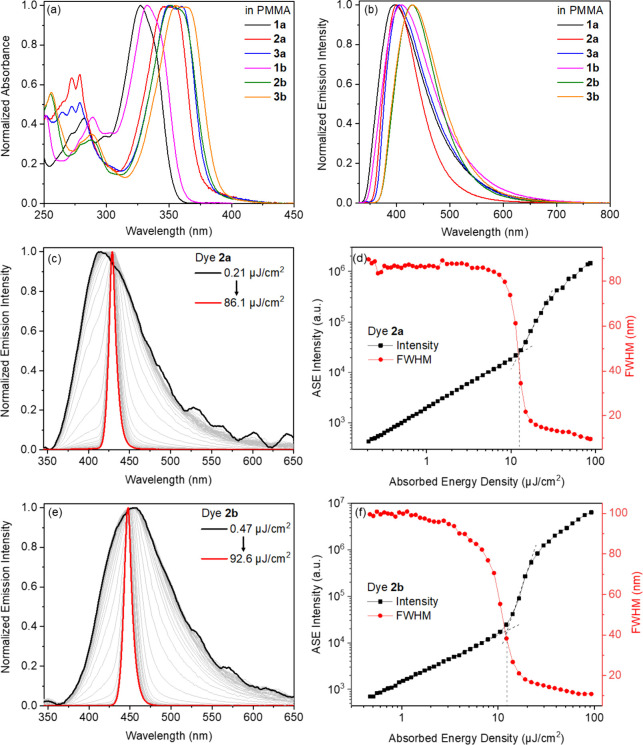
Absorption (a) and emission (b) spectra of dyes **1a**,**b**, **2a**,**b**, and **3a**,**b** doped in PMMA films. Normalized spectra of amplified spontaneous emission of PMMA films doped with dyes **2a** (c) and **2b** (e). Fwhm and ASE intensity values as a function of absorbed energy density for dyes **2a** (d) and **2b** (f).

The dyes in PMMA films are characterized with photoluminescence and, interestingly, in the line benzoxazole-benzothiazole-benzoselenazole, PL maximum is bathochromically shifted and reaches values of 397, 400, and 405 nm for unsubstituted carbazole-based dyes **1a**, **2a**, and **3a**, as well as 409, 429, and 430 nm for *tert*-butyl analogs **1b**, **2b**, and **3b,** respectively (Figure 6b, Table 2). Full width at half-maximum (fwhm) of the films equals to approximately 100 nm with the smallest value of 76 nm for benzothiazole derivative **2a** and with the highest one of 111 nm for boron complex **1b**. Benzoxazole-based dyes possess PLQY of 35 and 27% for **1a** and **1b**, respectively. PMMA films with benzothiazole derivative **2a** and **2b** show the highest PLQY reaching 69 and 61%, respectively. Meanwhile, benzoselenazole-containing compounds exhibit the smallest values of PLQY (8 and 12% for **3a** and **3b** respectively) mostly because of the presence of heavy atom (selenium) resulting in triplet states involvement in dyes photophysics.

**Table 2:** Photoluminescence Properties of the Films of PMMA Doped with Compounds **1a,b–3a,b**

Dye	λ_abs_, nm^a^	λ_PL_, nm^b^	fwhm_PL_, nm^c^	PLQY,%^d^	τ_p av_, ns^e^	τ_d av_, μs^f^	λ_ASE_, nm^g^	fwhm_ASE_, nm^h^	E_th‑ASE_, μJ/cm^2^ ^i^
**1a**	327	397	102	35	1.4	4.6	444	8.6	31.8
**2a**	347/354	400	76	69	2.4	7.4	429	9.5	12.5
**3a**	350/360	405	92	8	0.5	40.5	–	–	–
**1b**	333	409	111	27	1.3	4.7	455	8.2	47.3
**2b**	352/359	429	91	61	2.6	9.4	448	10.9	12.4
**3b**	354/363	430	100	12	0.4	58.2	–	–	–

a Wavelength of absorption maximum.

b Wavelength of photoluminescence maximum.

c Full width at half-maximum of the photoluminescence spectrum.

d Photoluminescence quantum yield measured in ambient atmosphere.

e Average excited-state lifetime in nanosecond range.

f Average excited-state lifetime in microsecond range.

g Wavelength of ASE maximum.

h Full width at half-maximum of the ASE spectrum.

i ASE threshold.

Excited-state lifetimes were further investigated to elucidate the photophysical behavior of the compounds. All compounds exhibited short prompt fluorescence lifetimes (τ_p_): 1.3–1.4 ns for the benzoxazole-containing derivatives (**1a**, **1b**), 2.4–2.6 ns for the benzothiazole-containing derivatives (**2a**, **2b**), and 0.4–0.5 ns for the benzoselenazole-containing dyes (**3a**, **3b**). In addition, all compounds displayed delayed fluorescence lifetimes (τ_d_) in the microsecond range: 4.6–4.7 μs for **1a,b**, 7.4–9.4 μs for **2a** and **2b**, and significantly longer values of 40.5–58.2 μs for **3a** and **3b**. These results indicate a clear trend of increasing delayed fluorescence lifetime with the heavier chalcogen atom, suggesting that the incorporation of selenium enhances the intersystem crossing and stabilizes the triplet states, thus favoring DF (e.g., through TTA). On the other hand, short value of τ_p_ accompanied by high PLQY (for compounds **1a,b** and **2a,b**) indicates lasing ability of the emitters. Therefore, we investigated ASE of the boron complexes.

The ASE characteristics of boron difluoride complexes **Cz-ONB-bCh** were subsequently investigated in PMMA-blended films (5 wt % for dyes **1a,b**, **2b**, and **3a,b**, and 2 wt % for compound **2a**) under ambient conditions using a nitrogen laser (λ = 337 nm, line-shaped excitation). The benzoselenazole-containing complexes **3a,b** exhibited no detectable ASE, most likely due to their low PLQYs and long delayed fluorescence lifetimes. In contrast, both the benzoxazole-based (**1a,b**) and benzothiazole-based (**2a,b**) analogues displayed pronounced ASE activity.

Boron difluorides **1a** and **1b** showed intense ASE peaks centered at 444 and 455 nm (Figure S33 in the SI), respectively, accompanied by a significant narrowing of the emission bandwidth (fwhm reduced to 8.6 and 8.2 nm, respectively). The corresponding ASE thresholds (E_th–ASE_) were determined to be 31.8 μJ/cm^2^ for compound **1a** and 47.3 μJ/cm^2^ for *tert*-butyl analogue **1b** (Table 2, Figure S33 in the SI).

The benzothiazole-containing derivatives **2a** and **2b** exhibited slightly hypsochromically shifted ASE emissions (λ_ASE_ = 429 and 448 nm, respectively, Table 2, Figures 6c,e) compared to their benzoxazole analogues. The small redshift of the ASE peak relatively to the fluorescence maximum observed for compound **2b** can be explained by the residual self-absorption around 429 nm (Figure 6a), which induces more photon losses in the short-wavelength part of the fluorescence spectrum all along the photon path in the stripe defined by the pump laser stripe. This effect is negligible for compound **2a** as this residual self-absorption is almost zero around 448 nm. The noisy aspect of the measured fluorescence for boron difluoride complex **2a** is due to its lower solubility, which leads to a smaller doping ratio (2%) and consequently comparatively weaker absorption and the fewer fluorescence photons. This does not affect the threshold as long as absorbed pump energy is considered. The fwhm values of both compounds remained in a comparable range (9.5–10.9 nm), while their ASE thresholds were remarkably lower (12.4–12.5 μJ/cm^2^, Figures 6d,f) than for boron difluorides **1a** and **1b**, which is consistent with their higher PLQY (Table 2). This low ASE thresholds highlights the highly efficient stimulated emission in those compounds, making them promising candidates for potential lasing operation.

Comparison of the ASE characteristics of **Cz-ONB-bCh** with those previously reported for **Ph-ONB-bCh** ^68^ reveals that the lowest ASE thresholds are always observed for the sulfur-containing derivatives, which can be attributed to their highest PLQY in the polymer matrix. Replacement of the phenyl substituent with a carbazole unit induces a hypsochromic shift of the ASE emission (λ_ASE_ = 463–468 nm for **Ph-ONB-bCh** vs 429–455 nm for **Cz-ONB-bCh**), accompanied by an increase in the ASE threshold (e.g., *E*_th‑ASE_ = 4.7 μJ/cm^2^ for **Ph-ONB-bS** compared to 12.4–12.5 μJ/cm^2^ for **Cz-ONB-bS** and **tBCz-ONB-bS**). On the other hand, incorporation of the carbazole moiety leads to the emergence of a delayed fluorescence component, even in the heavy-atom-free benzoxazole derivatives **Cz-ONB-bO** and **tBCz-ONB-bO**.

These findings reveal that subtle structural modifications in the heterocyclic fragment of boron difluoride complexes exert a pronounced influence on their ASE performance. The benzoxazole and benzothiazole derivatives exhibit efficient and tunable ASE in the blue spectral region, characterized by narrow emission bandwidths and low excitation thresholds. Such properties underscore their potential as promising candidates for incorporation into next-generation organic laser media. Conversely, the benzoselenazole analogues appear less suitable for ASE applications due to their intrinsically lower emissive efficiencies and prolonged excited-state dynamics.

## Conclusions

In summary, we elaborated a new family of benzochalcogene-containing donor–acceptor boron difluoride complexes (**1a,b–3a,b**). Systematic variation of the chalcogen atom (O, S, Se) within the benzochalcogenazole core revealed a pronounced influence on the photophysical properties. While the complexes are nonemissive in solution, they exhibit strong aggregation-induced emission and intense solid-state luminescence, with photoluminescence quantum yields reaching up to 85% in the crystalline state and 69% in PMMA films. The benzoxazole- and benzothiazole-based derivatives demonstrated amplified spontaneous emission with low thresholds (12.4 μJ/cm^2^) and narrow emission bands (fwhm up to 9.5 nm), indicating their suitability for laser applications. Furthermore, the observation of both prompt and delayed fluorescence confirms efficient exciton utilization. These findings establish benzochalcogenazole-based boron difluoride complexes as promising emissive materials for organic photonic and optoelectronic devices. The results underscore the importance of molecular design and chalcogen substitution in controlling emission characteristics, offering valuable guidelines for the development of next-generation, high-performance, and thermally stable light-emitting materials.

## Experimental Section

### General Methods

All reagents and chemicals (at least analytical grade) were purchased from commercial sources (ThermoScientific, TCI, Acros Organics, or Roth) and used without further purification. Synthesized compounds were purified via flash column chromatography on silica gel (Merck, 230–400 mesh). Reactions progress was controlled by thin layer chromatography (TLC), carried out on commercially available aluminum plates covered with silica gel (60 F_254_, Merck).

### Instrumental Methods

NMR spectra were recorded on Varian Mercury 400 MHz (400, 100, and 375 MHz for ^1^H, ^13^C, and ^19^F NMR spectra, respectively), Varian V NMRS 500 MHz (500, 125, 470, and 95 MHz for ^1^H, ^13^C, ^19^F, and ^77^Se NMR spectra, respectively), or Varian V NMRS 600 MHz (600 and 150 MHz for ^1^H and ^13^C NMR spectra, respectively) spectrometers at room temperature unless otherwise stated. CDCl_3_ or DMSO-*d*_*6*_ was used as the solvent, and chemical shifts were referenced externally to SiMe_4_. Signal multiplicities are reported as “s”, “br s”, “d”, “t”, or “m”, corresponding to singlet, broad singlet, doublet, triplet, and multiplet, respectively. Data were processed using MestReNova software. High-resolution mass spectra (HRMS) were collected on a Synapt G2-S HDMS (Waters Inc.) mass spectrometer equipped with an atmospheric-pressure chemical ionization (APCI) ion source or electrospray ionization (ESI) ion source and quadrupole-Time-of-Flight (q-TOF) mass analyzer. The instrument was controlled and recorded data were processed using MassLynx V4.1 software package (Waters Inc.). Spectra were collected in ESI or APCI mode.

Single crystals of boron difluoride complexes **1a,b**, **2a**, **3a,b** were grown by slow evaporation of their solution in hexane/dichloromethane (10:1) mixture for structure **1a**, hexane/dichloromethane (5:1) mixture for structure **1b**, toluene for structures **2a**, **3a**, and **3b** under ambient conditions. Single crystal X-ray diffraction measurements were carried out on a Agilent Supernova diffractometer, at 100 K with monochromated CuKα radiation (1.54184 Å). The data reduction was made by using *CrysAlisPRO* software.^73^ The structures were solved by direct methods and refined with the olex2.refine^74^ refinement package using Gauss–Newton minimization. All non-hydrogen atoms were refined as anisotropic while hydrogen atoms were placed in calculated positions and refined in riding mode. Compound **3b** contains two symmetry-independent molecules in the unit cell. Crystallographic data of compounds **1a,b**, **2a**, **3a,b** have been deposited with the Cambridge Crystallographic Data Centre (CCDC) and can be obtained, free of charge, from CCDC via https://www.ccdc.cam.ac.uk/structures/.

The ground-state geometries of compounds **1a,b–3a,b** were optimized using the B3LYP functional with the 6–31G­(d) basis set as implemented in Gaussian 16.^75^ For main-group elements, the 6–31G­(d) basis set was used, while heavy atoms were treated with the SDD (Stuttgart/Dresden) effective core potential,^76^ which incorporates relativistic effects. This B3LYP/6–31G­(d)/SDD computational scheme has been extensively applied and validated in previous investigations of heavy-atom-containing systems.^77,78^ For excited-state analyses, the LC-ωPBE functional paired with the 6–31+G­(d,p) basis set was employed, as this combination offers enhanced reliability for modeling charge-transfer behavior in dye molecules.^79,80^ Molecular orbitals were visualized using GaussView 6.0.^81^

UV–vis spectra in solutions were recorded on a Shimadzu UV-3600i Plus spectrophotometer at room temperature. Steady-state fluorescence emission spectra were performed at room temperature on a Edinburgh Instruments Spectrophotometer (FS5, Edinburgh, UK) with xenon lamp as the light source. The absolute fluorescence quantum yields were measured with a calibrated SC-30 Integrating Sphere and were excited at the appropriate absorption wavelength in each case. Fluorescence decays of the solutions and of the solid-state samples were recorded with the PicoQuant PDL 820 ps pulsed diode laser as an excitation source (λ_ex_ = 340 nm) using a time-correlated single photon counting technique (TCSPC). Delayed fluorescence was measured for degassed solutions using Multi-Channel Scaling (MCS) technique with microsecond xenon flashlamp as an excitation source. All measurements were performed at room temperature. Solutions were degassed using three freeze–pump–thaw cycles. Average excited-state lifetimes in the nanosecond and microsecond domains were derived using two- or three-exponential decay models and calculated with the following equation: *τ*_*av*_ = ∑_*i* = 1_^*n*^ (*A*_*i*_ *τ*_*i*_^2^)/∑_*i* = 1_^*n*^ *A*_*i*_ *τ*_*i*_, where A_i_ is the pre-exponential factor, and τ_i_ is the lifetime of the *i*_th_ component.

To investigate AIE, THF/water mixtures of varying ratios were prepared by gradually adding distilled water to THF solutions of compounds **1a,b**, **2a,b**, or **3a,b** maintaining a final concentration of 5 × 10^–6^ M. Photoluminescence measurements were performed immediately after sample preparation.

ASE measurements were performed using a nitrogen laser (337 nm; Stanford Research Systems NL100) with a pulse duration of 3.5 ns as the pump source. The experimental setup (Figure S32a in the SI) followed a previously reported configuration.^46^ A stripe-shaped excitation was generated by focusing the laser onto an adjustable slit using a cylindrical UV lens (focal length 300 mm). The slit was relay-imaged onto the sample through a 1:1 telescope consisting of two identical UV-coated singlet lenses (focal length 100 mm; Thorlabs LA4380-UV-ML). Imaging the slit onto the sample, rather than placing the sample directly at the slit, ensured a sharp transition between pumped and unpumped regions and eliminated Fresnel diffraction effects.^82^ The resulting excitation stripe on the organic thin film measured 11.8 mm × 200 μm (length × width), as determined at 50% of the maximum intensity (Figure S32b in the SI), promoting efficient one-dimensional optical amplification. To ensure proper waveguiding, dye-doped PMMA layers (refractive index = 1.50 at 475 nm) were spin-coated onto silica substrates (refractive index = 1.47 at 475 nm) at 1500 rpm for 60 s, followed by drying at 70 °C for 10 min. The resulting film thickness (∼500 nm) was selected to support only the fundamental transverse electric (TE_00_) mode. Prior to spectral measurements, the substrates were cleaved to provide a clean edge for efficient collection of the directional ASE emission. Emission spectra were acquired using a fiber-coupled spectrometer (Horiba iHR550, 150 lines/mm grating) with a 400 μm core optical fiber positioned in close proximity to the cleaved facet. All spectra were normalized and smoothed to reduce noise and enable reliable determination of the fwhm. The ASE threshold was defined as the pump energy or peak power at which the slope of the integrated emission intensity exhibited its maximum change.

### Synthesis

#### 9*H*-Carbazole-9-carbonyl chloride (5a)

The reaction was conducted under an argon atmosphere. A solution of triphosgene (1.11 g, 3.74 mmol, 0.5 equiv) in dry dichloromethane (5 mL) was cooled at −30 °C and dry pyridine (2.37 g, 29.9 mmol, 4.0 equiv) was slowly added to the flask. After stirring for 20 min at −30 °C, a solution of carbazole (**4a**, 1.25 g, 7.48 mmol, 1.0 equiv) in dry dichloromethane (15 mL) was slowly added. The reaction mixture was warmed to room temperature and stirred for 12 h at room temperature. Next, 1N aqueous solution of HCl (15 mL) was carefully added to quench the reaction. Then, the layers were separated, and the aqueous phase was extracted with dichloromethane (3 × 20 mL). The combined organic layers were washed with water and brine, then dried over Na_2_SO_4_. After the filtration, the solution was concentrated in vacuo, and the resulting residue was purified by flash column chromatography on silica gel (hexanes/dichloromethane from 8:1 to 2:1, v/v) to afford pure compound **5a** (1.53 g, 6.66 mmol, 89%) as a white powder. ^1^H NMR (400 MHz, CDCl_3_): δ = 8.42 (2H, d, *J* = 8.3 Hz, Ar–H), 7.98 (2H, d, *J* = 7.4 Hz, Ar–H), 7.53–7.43 (4H, m, Ar–H) ppm; ^13^C­{H} NMR (100 MHz, CDCl_3_): δ = 144.6, 138.4 (2C), 127.8 (2C), 126.9 (2C), 125.3 (2C), 120.0 (2C), 117.1 (2C) ppm.

#### 3,6-Di-*tert*-butyl-9*H*-carbazole-9-carbonyl chloride (5b)

The reaction was conducted under an argon atmosphere. A solution of triphosgene (1.06 g, 3.58 mmol, 0.5 equiv) in dry dichloromethane (5 mL) was cooled at −30 °C and dry pyridine (2.31 mL, 28.63 mmol, 4.0 equiv) was slowly added to the flask. After stirring for 20 min at −30 °C, a solution of 3,6-di-*tert*-butylcarbazole (**4b**, 2.00 g, 7.16 mmol, 1.0 equiv) in dry dichloromethane (15 mL) was slowly added. The reaction mixture was warmed to room temperature and stirred for 12 h at room temperature. Next, 1N aqueous solution of HCl (15 mL) was carefully added to quench the reaction. Then, the layers were separated, and the aqueous phase was extracted with dichloromethane (3 × 20 mL). The combined organic layers were washed with water and brine, then dried over Na_2_SO_4_. After the filtration, the solution was concentrated in vacuo, and the resulting residue was purified by flash column chromatography on silica gel (hexanes/dichloromethane from 100:0 to 4:1, v/v) to afford pure compound **5b** (2.11 g, 6.17 mmol, 86%) as a white powder. ^1^H NMR (400 MHz, CDCl_3_): δ = 8.31 (2H, d, *J* = 8.9 Hz, Ar–H), 7.97 (2H, d, *J* = 1.9 Hz, Ar–H), 7.54 (2H, dd, *J* = 8.9 Hz, *J* = 2.0 Hz, Ar–H), 1.46 (18H, s, 6CH_3_) ppm; ^13^C­{H} NMR (100 MHz, CDCl_3_): δ = 148.6, 144.4 (2C), 136.7 (2C), 127.1 (2C), 125.3 (2C), 116.7 (2C), 116.3 (2C), 35.0 (2C), 31.8 (6C) ppm.

#### *N*-((2-Hydroxyphenyl)­carbamothioyl)-9*H*-carbazole-9-carboxamide (10a)

Compound **5a** (653 mg, 2.84 mmol) was dissolved in acetone (15 mL). KSCN (276 mg, 2.84 mmol) was added, and the mixture was refluxed for 1h. 2-Aminophenol (**7**, 310 mg, 2.84 mmol) was added and the refluxing was continued for 1h. After cooling, the solvent was evaporated, water (20 mL) was added. The mixture was extracted with dichloromethane (3 × 30 mL). The combined organic layers were dried over Na_2_SO_4_, filtered and concentrated. The resulting residue was purified by flash column chromatography on silica gel (hexanes/dichloromethane = 1:1, v/v) to afford pure product **10a** (620 mg, 1.72 mmol, 60%) as a white powder. ^1^H NMR (400 MHz, DMSO-*d*_6_): δ = 12.05 (1H, br s, NH), 11.59 (1H, br s, NH), 10.24 (1H, br s, OH), 8.54 (1H, d, *J* = 8.1 Hz, Ar–H), 8.22 (2H, d, *J* = 7.7 Hz, Ar–H), 7.99 (2H, d, *J* = 8.3 Hz, Ar–H), 7.58 (2H, dd, *J* = 7.8 Hz, *J* = 1.3 Hz, Ar–H), 7.44 (2H, dd, *J* = 7.5 Hz, *J* = 1.3 Hz, Ar–H), 7.11 (1H, ddd, *J* = 9.3 Hz, *J* = 7.6 Hz, *J* = 1.6 Hz, Ar–H), 6.98 (1H, dd, *J* = 8.1 Hz, *J* = 1.4 Hz, Ar–H), 6.88 (1H, ddd, *J* = 8.3 Hz, *J* = 6.9 Hz, *J* = 1.4 Hz, Ar–H) ppm; ^13^C­{H} NMR (100 MHz, DMSO-*d*_6_): δ = 176.6, 150.1, 149.0, 137.7 (2C), 127.1 (2C), 126.6, 126.0, 124.9 (2C), 123.3, 123.2 (2C), 120.5 (2C), 118.5, 115.2, 114.4 (2C) ppm. HRMS (APCI) calcd for C_20_H_16_N_3_O_2_S [M + H]^+^: 362.0963, found: 362.0965.

#### 3,6-Di-*tert*-butyl-*N*-((2-hydroxyphenyl)­carbamothioyl)-9*H*-carbazole-9-carboxamide (10b)

Compound **5b** (377 mg, 1.10 mmol) was dissolved in acetone (15 mL). KSCN (107 mg, 1.10 mmol) was added, and the mixture was refluxed for 1h. 2-Aminophenol (**7**, 120 mg, 1.10 mmol) was added and the refluxing was continued for 1h. After cooling, the solvent was evaporated, water (25 mL) was added. The mixture was extracted with dichloromethane (3 × 30 mL). The combined organic layers were dried over Na_2_SO_4_, filtered and concentrated. The resulting residue was purified by flash column chromatography on silica gel (hexanes/dichloromethane = 1:1, v/v), to afford pure product **10b** (378 mg, 0.80 mmol, 72%) as a white powder. ^1^H NMR (600 MHz, DMSO-*d*_6_): δ = 12.07 (1H, br s, NH), 11.46 (1H, br s, NH), 10.26 (1H, br s, OH), 8.55 (1H, d, *J* = 7.9 Hz, Ar–H), 8.29 (2H, d, *J* = 1.7 Hz, Ar–H), 7.90 (2H, d, *J* = 8.7 Hz, Ar–H), 7.61 (2H, dd, *J* = 8.7 Hz, *J* = 1.3 Hz, Ar–H), 7.10 (1H, ddd, *J* = 8.9 Hz, *J* = 7.9 Hz, *J* = 1.5 Hz, Ar–H), 6.98 (1H, dd, *J* = 8.0 Hz, *J* = 1.1 Hz, Ar–H), 6.88 (1H, ddd, *J* = 9.0 Hz, *J* = 8.1 Hz, *J* = 0.9 Hz, Ar–H), 1.43 (18H, s, 6CH_3_) ppm; ^13^C­{H} NMR (150 MHz, DMSO-*d*_6_): δ = 176.6, 150.0, 149.0, 146.0 (2C), 136.0 (2C), 126.5, 126.0, 125.2 (2C), 124.5 (2C), 123.2, 118.4, 116.9 (2C), 115.2, 113.9 (2C), 34.7 (2C), 31.6 (6C) ppm. HRMS (APCI) calcd for C_28_H_32_N_3_O_2_S [M + H]^+^: 474.2215, found: 474.2220.

#### *N*-(Benzo­[*d*]­oxazol-2-yl)-9*H*-carbazole-9-carboxamide (14a)

A mixture of compound **10a** (600 mg, 1.66 mmol) and *N*,*N*-diethyl-*N*-{[(methoxycarbonyl)­amino]­sulfonyl}-ethanaminium inner salt (**13**, 435 mg, 1.83 mmol, 1.05 equiv) in toluene (12 mL) was stirred for 3h at 70 °C. After cooling to room temperature the solution was concentrated in vacuo, and the resulting residue was purified by flash column chromatography on silica gel (hexanes/dichloromethane = from 100:0 to 1:1, v/v) to give pure product **14a** (430 mg, 1.31 mmol, 79%) as a white powder. ^1^H NMR (400 MHz, DMSO-*d*_6_): δ = 13.08 (1H, br s, NH), 8.83 (2H, d, *J* = 8.4 Hz, Ar–H), 8.17 (2H, d, *J* = 7.4 Hz, Ar–H), 7.62 (1H, dd, *J* = 7.7 Hz, *J* = 1.3 Hz, Ar–H), 7.52 (3H, dddd, *J* = 9.8 Hz, *J* = 8.6 Hz, *J* = 7.2 Hz, *J* = 1.4 Hz, Ar–H), 7.40–7.32 (4H, m, Ar–H) ppm; ^13^C­{H} NMR (100 MHz, DMSO-*d*_6_): δ = 160.8, 158.9, 143.3, 138.6 (2C), 130.0, 126.8 (2C), 125.1 (2C), 125.1, 123.9, 122.7 (2C), 119.7 (2C), 117.2 (2C), 112.3, 110.4 ppm. HRMS (ESI-TOF) calcd for C_20_H_14_N_3_O_2_ [M + H]^+^: 328.1086, found: 328.1090.

#### *N*-(Benzo­[*d*]­oxazol-2-yl)-3,6-di-*tert*-butyl-9*H*-carbazole-9-carboxamide (14b)

A mixture of compound **10b** (340 mg, 0.72 mmol) and *N*,*N*-diethyl-*N*-{[(methoxycarbonyl)­amino]­sulfonyl}-ethanaminium inner salt (**13**, 188 g, 0.79 mmol, 1.05 equiv) in toluene (15 mL) was stirred for 3h at 70 °C. After cooling to room temperature the solution was concentrated in vacuo, and the resulting residue was purified by flash column chromatography on silica gel (hexanes/dichloromethane = from 100:0 to 1:1, v/v) to give pure product **14b** (253 mg, 0.58 mmol, 80%) as a white powder. ^1^H NMR (500 MHz, DMSO-*d*_6_): δ = 13.03 (1H, br s, NH), 8.68 (2H, d, *J* = 8.8 Hz, Ar–H), 8.22 (2H, d, *J* = 1.7 Hz, Ar–H), 7.63 (1H, d, *J* = 7.5 Hz, Ar–H), 7.55 (2H, dd, *J* = 8.8 Hz, *J* = 2.0 Hz, Ar–H), 7.48 (1H, d, *J* = 7.6 Hz, Ar–H), 7.36 (1H, ddd, *J* = 8.5 Hz, *J* = 7.5 Hz, *J* = 1.0 Hz, Ar–H), 7.31 (1H, ddd, *J* = 9.1 Hz, *J* = 7.8 Hz, *J* = 1.5 Hz, Ar–H), 1.42 (18H, s, 6CH_3_) ppm; ^13^C­{H} NMR (125 MHz, DMSO-*d*_6_): δ = 160.7, 158.9, 145.6 (2C), 143.3, 137.0 (2C), 130.1, 125.4 (2C), 125.1, 124.1 (2C), 123.8, 116.7, 116.1 (2C), 112.3, 110.4 (2C), 34.5 (2C), 31.6 (6C) ppm. HRMS (ESI-TOF) calcd for C_28_H_30_N_3_O_2_ [M + H]^+^: 440.2338, found: 440.2335.

### General Procedure A: Synthesis of Ligands 15a,b and 16a,b

Compound **5a,b** (1.0 equiv) was dissolved in acetone (15 mL). KSCN (1.1 equiv) was added, and the mixture was refluxed for 1h. Aniline **8** (1.0 equiv) or **9** (0.5 equiv) was added and the refluxing was continued for 1h. After cooling to room temperature, the solvent was evaporated, was added water (20 mL), and the phases were separated. The aqueous phase was extracted with CH_2_Cl_2_ (3 × 20 mL). The combined organic phase was dried over anhydrous Na_2_SO_4_, filtered and concentrated. The crude product was purified by column chromatography on silica gel (hexanes/CH_2_Cl_2_ from 8:1 to 1:3, v/v).

#### *N*-(Benzo­[*d*]­thiazol-2-yl)-9*H*-carbazole-9-carboxamide (15a)

was obtained as a white solid in 74% yield (488 mg) from compound **5a** (441 mg) and 2-aminobenzenethiol (**8**, 240 mg) using general procedure A. ^1^H NMR (500 MHz, DMSO-*d*_6_): δ = 13.57 (1H, br s, NH), 8.89 (2H, d, *J* = 8.3 Hz, Ar–H), 8.14 (2H, d, *J* = 7.6 Hz, Ar–H), 7.88 (1H, d, *J* = 7.8 Hz, Hz, Ar–H), 7.49–7.55 (3H, m, Ar–H), 7.47 (1H, dd, *J* = 7.4 Hz, *J* = 7.2 Hz, Ar–H), 7.36 (2H, dd, *J* = 7.6 Hz, *J* = 7.3 Hz, Ar–H), 7.30 (1H, dd, *J* = 7.8 Hz, *J* = 7.2 Hz, Ar–H) ppm; ^13^C­{H} NMR (125 MHz, DMSO-*d*_6_): δ = 169.4, 159.6, 138.6 (2C), 136.1, 127.2, 126.8 (2C), 126.6, 125.2 (2C), 123.5, 122.8, 122.6 (2C), 119.7 (2C), 117.4 (2C), 113.1 ppm. HRMS (ESI-TOF) calcd for C_20_H_14_N_3_OS [M + H]^+^: 344.0858, found: 344.0861.

#### *N*-(Benzo­[*d*]­thiazol-2-yl)-3,6-di-*tert*-butyl-9*H*-carbazole-9-carboxamide (15b)

was obtained as a white solid in 57% yield (724 mg) from compound **5b** (950 mg) and 2-aminobenzenethiol (**8**, 349 mg) using general procedure A. ^1^H NMR (600 MHz, DMSO-*d*_6_): δ = 13.48 (1H, br s, NH), 8.74 (2H, d, *J* = 7.0 Hz, Ar–H), 8.23 (2H, d, *J* = 1.9 Hz, Ar–H), 7.88 (1H, d, *J* = 7.8 Hz, Ar–H), 7.51–7.55 (3H, m, Ar–H), 7.47 (1H, ddd, *J* = 8.2 Hz, *J* = 7.3 Hz, *J* = 1.1 Hz, Ar–H), 7.32 (1H, ddd, *J* = 8.3 Hz, *J* = 7.9 Hz, *J* = 1.1 Hz, Ar–H), 1.43 (18H, s, 6CH_3_) ppm; ^13^C­{H} NMR (150 MHz, DMSO-*d*_6_): δ = 169.1, 159.5, 145.1 (2C), 136.9 (2C), 136.0, 127.1, 126.5, 125.3 (2C), 124.0 (2C), 123.4, 122.8, 116.8 (2C), 116.1 (2C), 112.9, 34.5 (2C), 31.6 (6C) ppm. HRMS (ESI-TOF) calcd for C_28_H_30_N_3_OS [M + H]^+^: 456.2110, found: 456.2112.

#### *N*-(Benzo­[*d*]­[1,3]­selenazol-2-yl)-9*H*-carbazole-9-carboxamide (16a)

was obtained as a light brown solid in 64% yield (414 mg) from compound **5a** (380 mg) and 2,2’-diselenediyldianiline (**9**, 283 mg) using general procedure A. ^1^H NMR (500 MHz, DMSO-*d*_6_): δ = 13.52 (1H, br s, NH), 8.90 (2H, d, *J* = 8.4 Hz, Ar–H), 8.18 (2H, d, *J* = 7.4 Hz, Ar–H), 7.98 (1H, d, *J* = 7.7 Hz, Ar–H), 7.57–7.51 (3H, m, Ar–H), 7.47 (1H, ddd, *J* = 8.1 Hz, *J* = 6.9 Hz, *J* = 1.3 Hz, Ar–H), 7.39 (2H, ddd, *J* = 8.0 Hz, *J* = 7.0 Hz, *J* = 1.0 Hz, Ar–H), 7.27 (1H, ddd, *J* = 8.0 Hz, *J* = 6.8 Hz, *J* = 1.3 Hz, Ar–H) ppm; ^13^C­{H} NMR (125 MHz, DMSO-*d*_6_): δ = 172.2, 160.1, 138.5 (2C), 137.5, 127.1, 126.9 (3C), 126.2, 125.2 (2C), 123.5, 122.8 (2C), 119.8 (2C), 117.5 (2C), 114.2 ppm; ^77^Se NMR (95 MHz, DMSO-*d*_6_): δ = 544.24 ppm. HRMS (APCI) calcd for C_20_H_14_N_3_OSe [M + H]^+^: 392.0302, found: 392.0298.

#### *N*-(Benzo­[*d*]­[1,3]­selenazol-2-yl)-3,6-di-*tert*-butyl-9*H*-carbazole-9-carboxamide (16b)

was obtained as a light brown solid in 43% yield (253 mg) from compound **5b** (400 mg) and 2,2’-diselenediyldianiline (**9**, 200 mg) using general procedure A. ^1^H NMR (600 MHz, DMSO-*d*_6_): δ = 13.40 (1H, br s, NH), 8.75 (2H, d, *J* = 8.6 Hz, Ar–H), 8.24 (2H, d, *J* = 1.9 Hz, Ar–H), 7.96 (1H, d, *J* = 8.3 Hz, Ar–H), 7.55–7.52 (3H, m, Ar–H), 7.46 (1H, dd, *J* = 8.3 Hz, *J* = 1.2 Hz, Ar–H), 7.26 (1H, dd, *J* = 8.3 Hz, *J* = 1.1 Hz, Ar–H), 1.43 (18H, s, 6CH_3_) ppm; ^13^C­{H} NMR (150 MHz, DMSO-*d*_6_): δ = 171.7, 160.0, 158.1, 145.3 (2C), 137.6, 136.8 (2C), 127.0, 126.8, 126.1, 125.4 (2C), 124.0 (2C), 123.4, 116.9, 116.2 (2C), 114.1, 34.5 (2C), 31.6 (6C) ppm. HRMS (ESI-TOF) calcd for C_28_H_28_N_3_OSe [M - H]^−^: 502.1398, found: 502.1397.

### Synthesis of Boron Difluoride Complexes 1a,b, 2a,b, and 3a,b

#### Optimization of the Synthesis of 3-(3,6-Di-*tert*-butyl-9*H*-carbazol-9-yl)-1,1-difluoro-1*H*-1λ^4^,10λ^4^-benzo­[4,5]­thiazolo­[3,2-*c*]­[1,3,5,2]­oxadiazaborinine (2b)

DIPEA (20 eq 0.765 mL, 4.39 mmol or 3 eq 0.115 mL, 0.66 mmol) was added to a suspension of compound **15b** (1 eq., 100 mg, 0.21 mmol) in solvent (CH_2_Cl_2_, CHCl_3_, or toluene, 10 mL). The mixture was stirred for 15 min, during which the substrate completely dissolved. Subsequently, BF_3_·Et_2_O (10 eq., 0.271 mL, 2.19 mmol, or 6 eq., 0.163 mL, 1.32 mmol) was added dropwise. The reaction mixture was stirred for 24 or 4 h at 20 °C, 60 °C, or 100 °C (see Table S1 in the SI). After completion, the mixture was quenched with water (10 mL), and the layers were separated. The aqueous phase was extracted with CH_2_Cl_2_ (2 × 20 mL). The combined organic layers were dried over anhydrous Na_2_SO_4_, filtered, and concentrated under reduced pressure. The crude residue was purified by column chromatography on silica gel (hexanes/CH_2_Cl_2_, 8:1 to 1:1, v/v) to afford the product as a yellowish crystalline solid.

^1^H NMR (600 MHz, CDCl_3_, room temperature): δ = 8.74 (1H, br s, Ar–H), 8.47 (1H, br s, Ar–H), 7.99–7.98 (3H, m, Ar–H), 7.75 (1H, dd, *J* = 8.0 Hz, *J* = 1.8 Hz, Ar–H), 7.59–7.56 (3H, m, Ar–H), 7.43 (1H, ddd, *J* = 7.3 Hz, *J* = 7.4 Hz, *J* = 1.1 Hz, Ar–H), 1.47 (18H, s, 6CH_3_) ppm; ^13^C­{H} NMR (150 MHz, CDCl_3_, room temperature): δ = 174.2, 156.6, 148.4, 140.2, 136.3, 128.2 (2C), 127.5 (br), 126.2, 125.9 (2C), 125.3 (br, 2C), 122.2 (2C), 118.1, 117.9 (2C), 116.1 (2C), 35.0 (2C), 31.8 (6C) ppm; ^19^F NMR (375 MHz, CDCl_3_, room temperature): δ = −138.08 (2F, m, BF_2_) ppm. ^1^H NMR (500 MHz, CDCl_3_, −40 °C): δ = 8.78 (1H, d, *J* = 8.8 Hz, Ar–H), 8.45 (1H, d, *J* = 8.8 Hz, Ar–H), 7.97–7.95 (3H, m, Ar–H), 7.78 (1H, d, *J* = 8.0 Hz, Ar–H), 7.60–7.56 (3H, m, Ar–H), 7.46 (1H, dd, *J* = 7.7 Hz, *J* = 7.6 Hz, Ar–H), 1.44 (18H, d, *J* = 2.8 Hz, 6CH_3_) ppm; ^13^C­{H} NMR (125 MHz, CDCl_3_, −40 °C): δ = 173.7, 156.0, 148.1, 148.0, 139.6, 135.9, 135.7, 128.2, 127.3, 126.9, 125.9 (2C), 125.5, 125.0, 122.2, 118.0, 117.7, 117.4, 116.2, 116.1, 34.9 (2C), 31.7 (3C), 31.7 (3C) ppm; ^19^F NMR (470 MHz, CDCl_3_, −40 °C): δ = −137.35 (2F, m, BF_2_) ppm.

HRMS (APCI) calcd for C_28_H_29_BN_3_O_2_F_2_S [M + H]^+^: 504.2092, found: 504.2091.

### General Procedure B: Synthesis of Compounds 1a,b, 2a, and 3a,b

DIPEA (3 equiv) was added to a suspension of ligand **14a,b**, **15a**, or **16a,b** (1 equiv) in toluene (10 mL). The mixture was stirred for 15 min, where the substrate was totally dissolved. Then, BF_3_·Et_2_O (6 equiv) was added dropwise. The reaction was stirred for 4h at 100 °C. After that, the solution was mixed with water (20 mL), and the phases were separated. The aqueous phase was extracted with CH_2_Cl_2_ (2 × 20 mL). The combined organic phase was dried over anhydrous Na_2_SO_4_, filtered and concentrated. The crude product was purified by column chromatography on silica gel (hexanes/CH_2_Cl_2_ from 8:1 to 1:1, v/v).

#### 3-(9*H*-Carbazol-9-yl)-1,1-difluoro-1*H*-1λ^4^,10λ^4^-benzo­[4,5]­oxazolo­[3,2-*c*]­[1,3,5,2]­oxadiazaborinine (1a)

was obtained as a white solid in 44% yield (202 mg) using general procedure B from ligand **14a** (400 mg). ^1^H NMR (600 MHz, CDCl_3_): δ = 8.92 (1H, br s, Ar–H), 8.58 (1H, br s, Ar–H), 7.98 (2H, dd, *J* = 7.6 Hz, *J* = 0.6 Hz, Ar–H), 7.72 (1H, d, *J* = 7.6 Hz, Ar–H), 7.60 (1H, dd, *J* = 7.7 Hz, *J* = 0.9 Hz, Ar–H), 7.55 (2H, ddd, *J* = 8.6 Hz, *J* = 7.4 Hz, *J* = 1.3 Hz, Ar–H), 7.51–7.42 (4H, m, Ar–H) ppm; ^13^C­{H} NMR (150 MHz, CDCl_3_): δ = 163.2, 160.1, 146.1, 138.0, 130.0, 128.0, 126.7 (2C), 125.9 (2C), 125.4 (2C), 119.9 (2C), 119.1, 118.8, 114.8 (2C), 111.4 (2C) ppm; ^19^F NMR (375 MHz, CDCl_3_): δ = −138.06 (2F, m, BF_2_) ppm. HRMS (ESI-TOF) calcd for C_20_H_13_BN_3_O_2_F_2_ [M + H]^+^: 376.1069, found: 376.1068.

#### 3-(3,6-Di-*tert*-butyl-9*H*-carbazol-9-yl)-1,1-difluoro-1*H*-1λ^4^,10λ^4^-benzo­[4,5]­oxazolo­[3,2-*c*]­[1,3,5,2]­oxadiazaborinine (1b)

was obtained as a white solid in 65% yield (159 mg) using general procedure B from ligand **14b** (220 mg). ^1^H NMR (600 MHz, CDCl_3_, room temperature): δ = 8.83 (1H, d, *J* = 8.1 Hz, Ar–H), 8.44 (1H, d, *J* = 8.3 Hz, Ar–H), 7.97 (2H, d, *J* = 1.9 Hz, Ar–H), 7.70 (1H, d, *J* = 7.8 Hz, Ar–H), 7.56–7.60 (3H, m, Ar–H), 7.47 (1H, ddd, *J* = 8.2 Hz, *J* = 7.3 Hz, *J* = 1.0 Hz, Ar–H), 7.42 (1H, ddd, *J* = 9.2 Hz, *J* = 8.2 Hz, *J* = 1.2 Hz, Ar–H), 1.46 (18H, s, 6CH_3_) ppm; ^13^C­{H} NMR (150 MHz, CDCl_3_, room temperature): δ = 163.4, 159.8, 148.8, 146.1, 136.2, 130.2, 127.6, 126.5 (2C), 125.7 (2C), 125.3, 118.6, 118.1, 116.2 (2C), 114.7 (2C), 111.3 (2C), 35.1 (2C), 31.8 (6C) ppm; ^19^F NMR (375 MHz, CDCl_3_, room temperature): δ = −138.46 (2F, m, BF_2_) ppm. ^1^H NMR (600 MHz, CDCl_3_, −40 °C): δ = 8.81 (1H, d, *J* = 8.8 Hz, Ar–H), 8.43 (1H, d, *J* = 8.8 Hz, Ar–H), 7.96 (2H, t, *J* = 2.0 Hz, Ar–H), 7.68 (1H, d, *J* = 7.7 Hz, Ar–H), 7.60 (1H, d, *J* = 8.0 Hz, Ar–H), 7.57 (2H, dd, *J* = 5.3 Hz, *J* = 1.8 Hz, Ar–H), 7.47 (1H, ddd, *J* = 8.7 Hz, *J* = 7.7 Hz, *J* = 1.0 Hz, Ar–H), 7.43 (1H, ddd, *J* = 7.9 Hz, *J* = 7.7 Hz, *J* = 1.0 Hz, Ar–H), 1.44 (18H, d, *J* = 4.2 Hz, 6CH_3_) ppm; ^13^C­{H} NMR (150 MHz, CDCl_3_, −40 °C): δ = 162.8, 159.2, 148.5, 148.3, 145.6, 135.8, 135.6, 129.6, 127.4, 127.1, 126.5 (2C), 125.7, 125.6, 125.2, 118.3, 117.8, 116.2, 114.4, 111.4, 35.0, 34.9, 31.67 (3C), 31.65 (3C) ppm. HRMS (APCI) calcd for C_28_H_29_BN_3_O_2_F_2_ [M + H]^+^: 488.2321, found: 488.2322.

#### 3-(9*H*-Carbazol-9-yl)-1,1-difluoro-1*H*-1λ^4^,10λ^4^-benzo­[4,5]­thiazolo­[3,2-*c*]­[1,3,5,2]­oxadiazaborinine (2a)

was obtained as a yellowish crystalline solid in 44% yield (221 mg) using general procedure B from ligand **15a** (440 mg). ^1^H NMR (500 MHz, CDCl_3_, room temperature): δ = 8.73 (2H, br s, Ar–H), 8.02–7.99 (3H, m, Ar–H), 7.79 (1H, dd, *J* = 8.0 Hz, *J* = 1.0 Hz, Ar–H), 7.60 (1H, ddd, *J* = 8.5 Hz, *J* = 7.5 Hz, *J* = 1.2 Hz, Ar–H), 7.55 (2H, ddd, *J* = 8.6 Hz, *J* = 7.4 Hz, *J* = 1.3 Hz, Ar–H), 7.48–7.44 (3H, m, Ar–H) ppm; ^19^F NMR (375 MHz, CDCl_3_, room temperature): δ = −137.77 (2F, m, BF_2_) ppm. ^1^H NMR (500 MHz, CDCl_3_, −40 °C): δ = 8.91 (1H, d, *J* = 8.6 Hz, Ar–H), 8.56 (1H, d, *J* = 8.5 Hz, Ar–H), 8.01–7.98 (3H, m, Ar–H), 7.82 (1H, d, *J* = 8.1 Hz, Ar–H), 7.61 (1H, t, *J* = 7.6 Hz, Ar–H), 7.58–7.54 (2H, m, Ar–H), 7.50–7.45 (3H, m, Ar–H) ppm; ^13^C­{H} NMR (125 MHz, CDCl_3_, −40 °C): δ = 173.7, 156.3, 137.7, 137.5, 128.3, 128.0, 127.5, 127.2, 126.9, 126.1, 126.0, 125.1, 125.0, 122.3, 120.0, 119.9, 118.6, 118.3, 117.6, 101.7 pm.

HRMS (ESI-TOF) calcd for C_20_H_13_BN_3_OF_2_S [M + H]^+^: 392.0840, found: 392.0843.

#### 3-(9*H*-Carbazol-9-yl)-1,1-difluoro-1*H*-1λ^4^,10λ^4^-benzo­[4,5]­[1,3]­selenazolo­[3,2-*c*]­[1,3,5,2]­oxadiazaborinine (3a)

was obtained as a yellowish crystalline solid in 46% yield (205 mg) using general procedure B from ligand **16a** (395 mg). ^1^H NMR (600 MHz, CDCl_3_): δ = 8.71 (2H, br s, Ar–H), 8.07 (1H, d, *J* = 8.1 Hz, Ar–H), 7.99 (2H, d, *J* = 7.6 Hz, Ar–H), 7.79 (1H, d, *J* = 7.9 Hz, Ar–H), 7.57–7.53 (3H, m, Ar–H), 7.47 (2H, ddd, *J* = 8.4 Hz, *J* = 7.5 Hz, *J* = 1.6 Hz, Ar–H), 7.39 (1H, ddd, *J* = 8.1 Hz, *J* = 7.9 Hz, *J* = 0.7 Hz, Ar–H) ppm; ^13^C­{H} NMR (150 MHz, CDCl_3_): δ = 178.5, 155.6, 141.7, 138.1, 128.2 (2C), 127.9, 127.4, 126.1 (2C), 125.2 (2C), 125.2 (2C), 120.0 (2C), 119.70, 119.68, 118.6 (2C) ppm; ^19^F NMR (470 MHz, CDCl_3_): δ = −137.26 (2F, m, BF_2_) ppm; ^77^Se NMR (95 MHz, CDCl_3_): δ = 529.85 ppm. HRMS (APCI) calcd for C_20_H_13_BN_3_OF_2_Se [M + H]^+^: 440.0285, found: 440.0284.

#### 3-(3,6-Di-*tert*-butyl-9*H*-carbazol-9-yl)-1,1-difluoro-1*H*-1λ^4^,10λ^4^-benzo­[4,5]­[1,3]­selenazolo­[3,2-*c*]­[1,3,5,2]­oxadiazaborinine (3b)

was obtained as a yellowish crystalline solid in 76% yield (166 mg) using general procedure B from ligand **16b** (200 mg). ^1^H NMR (600 MHz, CDCl_3_, room temperature): δ = 8.73 (1H, br s, Ar–H), 8.46 (1H, br s, Ar–H), 8.04 (1H, d, *J* = 7.8 Hz, Ar–H), 7.97 (2H, d, *J* = 1.9 Hz, Ar–H), 7.76 (1H, d, *J* = 8.8 Hz, Ar–H), 7.58 (2H, dd, *J* = 8.8 Hz, *J* = 2.0 Hz, Ar–H), 7.55 (1H, ddd, *J* = 8.4 Hz, *J* = 8.4 Hz, *J* = 1.2 Hz, Ar–H), 7.37 (1H, ddd, *J* = 8.2 Hz, *J* = 8.1 Hz, *J* = 1.0 Hz, Ar–H), 1.46 (18H, s, 6CH_3_) ppm; ^13^C­{H} NMR (150 MHz, CDCl_3_, room temperature): δ = 178.4, 155.3, 148.5, 141.7, 136.6, 128.1 (2C), 127.7, 127.6 (br), 125.9 (2C), 125.4 (br, 2C), 125.1 (2C), 119.5, 118.1 (2C), 116.2 (2C), 35.0 (2C), 31.8 (6C) ppm; ^19^F NMR (375 MHz, CDCl_3_, room temperature): δ = −137.54 (2F, m, BF_2_) ppm; ^77^Se NMR (95 MHz, CDCl_3_): δ = 526.69 ppm. ^1^H NMR (600 MHz, CDCl_3_, −40 °C): δ = 8.75 (1H, d, *J* = 8.8 Hz, Ar–H), 8.44 (1H, d, *J* = 8.8 Hz, Ar–H), 8.01 (1H, d, *J* = 8.0 Hz, Ar–H), 7.97 (2H, d, *J* = 1.9 Hz, Ar–H), 7.77 (1H, d, *J* = 7.9 Hz, Ar–H), 7.58–7.54 (3H, m, Ar–H), 7.38 (1H, ddd, *J* = 8.0 Hz, *J* = 7.2 Hz, *J* = 0.8 Hz, Ar–H), 1.44 (18H, d, *J* = 2.4 Hz, 6CH_3_) ppm; ^13^C­{H} NMR (150 MHz, CDCl_3_, −40 °C): δ = 178.1, 154.7, 148.2, 148.0, 141.1, 135.9, 135.7, 128.0, 127.5, 127.3, 127.0, 125.9, 125.5, 125.1, 125.0, 122.9, 119.0, 117.9, 117.7, 116.18, 116.13, 34.9 (2C), 31.7 (3C), 31.7 (3C) ppm. HRMS (APCI) calcd for C_28_H_29_BN_3_OF_2_Se [M + H]^+^: 552.1537, found: 552.1534.

## Supplementary Material



## Notes

ORTEP diagrams for the X-ray structures and crystal data of compounds **1a,b, 2a**, and **3a,b**; (TD)­DFT calculations data; additional photophysical spectra and data; NMR spectra of the synthesized compounds (PDF)

## Abbreviations

PLQYs: photoluminescence quantum yields
ASE: amplified spontaneous emission
BODIPY: boron dipyrromethene
BF_2_: boron difluoride
D–A: donor–acceptor
ICT: intramolecular charge transfer
SOC: spin–orbit coupling
TADF: thermally activated delayed fluorescence
BF_3_·OEt_2_: boron trifluoride etherate
DIPEA: *N*,*N*-diisopropylethylamine
HRMS: high-resolution mass spectrometry
Ch: chalcogen
DFT: density functional theory
HOMO: highest occupied molecular orbital
LUMO: lowest unoccupied molecular orbital
TD-DFT: Time-dependent density functional theory
LE: locally excited
CT: charge-transfer
NTO: natural transition orbital
HONTO: highest occupied natural transition orbital
LUNTO: lowest unoccupied natural transition orbital
TICT: twisted intramolecular charge-transfer
SOCME: spin–orbit coupling matrix elements
ISC/RISC: intersystem crossing/reverse intersystem crossing
AIE: aggregation-induced emission
PMMA: poly­(methyl methacrylate)
DF: delayed fluorescence
TTA: triplet–triplet annihilation
fwhm: full width at half-maximum
TLC: thin layer chromatography
APCI: atmospheric-pressure chemical ionization
ESI: electrospray ionization
q-TOF: quadrupole-Time-of-Flight
CCDC: Cambridge Crystallographic Data Centre
KSCN: potassium thiocyanate
CDCl_3_: deuterated chloroform
DMSO-*d*_6_: deuterated dimethyl sulfoxide.
