# Supplementary material for: Blue-Emitting N,O‑Coordinated Boron Difluoride Complexes with Benzochalcogenazole-Containing Donor–Acceptor Frameworks Featuring Amplified Spontaneous Emission and Delayed Fluorescence
Source: Inorg Chem. 2026 Apr 28;65(18):9834–49. doi: 10.1021/acs.inorgchem.5c05992 (PMC13298887; doi:10.1021/acs.inorgchem.5c05992)
Supplement: Supplementary file 1 [file ic5c05992_si_001.pdf]

Supporting Information

Blue-Emitting *N,O*-Coordinated Boron Difluoride Complexes with Benzochalcogenazole-Containing Donor–Acceptor Frameworks Featuring Amplified Spontaneous Emission and Delayed Fluorescence

Hanna Zinchenko[a,b], Enzo Jean-Woldemar[c], Andrii Hotynchan[b], Khrystyna Ivaniuk[a,d], Yuliia Sadova[a,e], Roman Luboradzki[f], Paulina H. Marek-Urban[g], Sébastien Chénais[c], Pavlo Stakhira[d], Krzysztof Durka[g,*], Sébastien Forget[c,*], Mykhaylo A. Potopnyk[a,b,*]

[a] Institute of Organic Chemistry, Polish Academy of Sciences, Kasprzaka 44/52, Warsaw, 01-224, Poland, Email: mykhaylo.potopnyk@icho.edu.pl

[b] Department of Organic Chemistry, Faculty of Chemistry, Ivan Franko National University of Lviv, Kyryla i Mefodiya 6, Lviv, 79005, Ukraine, Email: potopnyk@gmail.com

[c] Laboratoire de Physique des Lasers, Université Sorbonne Paris Nord, CNRS, UMR 7538, Villetaneuse, F-93430, France, Email: sebastien.forget@univparis13.fr

[d] Department of Electronic Engineering, Lviv Polytechnic National University, Sviatoho Yura sq. 1, Lviv, 79013, Ukraine

[e] V. Bakul Institute for Superhard Materials, National Academy of Sciences of Ukraine, Avtozavodska 2, Kyiv, 04074, Ukraine

[f] Institute of Physical Chemistry, Polish Academy of Sciences, Kasprzaka 44/52, Warsaw, 01-224, Poland

[g] Faculty of Chemistry, Warsaw University of Technology, Noakowskiego 3, Warsaw, 00-664, Poland, Email: krzysztof.durka@pw.edu.pl

Table of Contents

1. Single Crystal X-ray Diffraction S3
2. Theoretical Calculations S18
3. Photophysical Properties in Solutions S37
4. Photophysical Properties in Crystalline State S45
5. Photophysical Properties of Dye-Doped PMMA Films S47
6. ASE Measurements S48
7. Copies of NMR Spectra S50

1. Synthesis

Table S1. Optimization of reaction conditions for synthesis of dye **2b**.

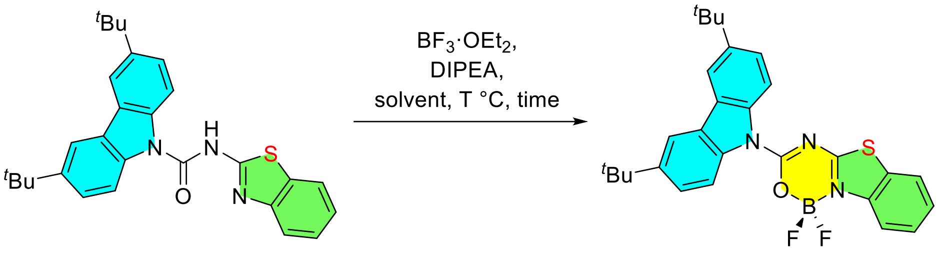

15b **2b**

entry	Solvent	Eq. of $BF_3·OEt_2$	Eq. of DIPEA	T, °C	Time, h	Yield of product, %
1	CH_2Cl_2	6	3	20	4	43
2	CH_2Cl_2	6	3	20	24	48
3	CH_2Cl_2	10	20	20	24	43
4	$CHCl_3$	6	3	60	24	73
5	$CHCl_3$	10	20	60	24	63
6	toluene	6	3	100	4	86

2. Single Crystal X-ray Diffraction

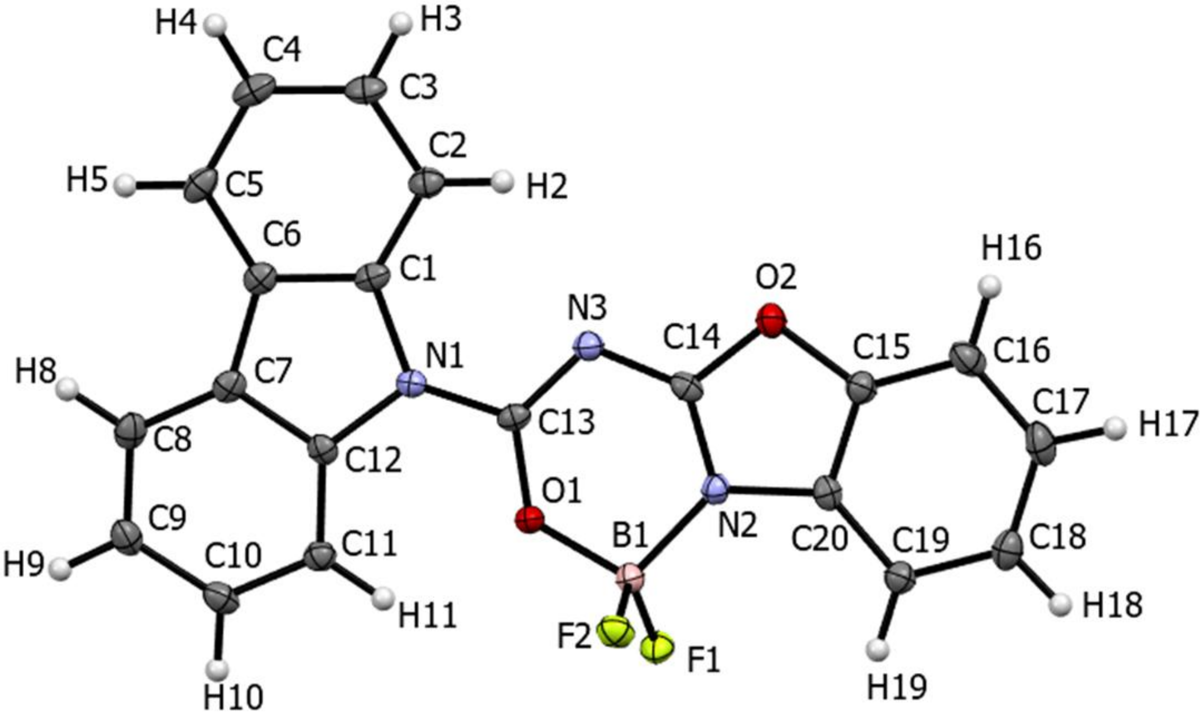

Figure S1. ORTEP diagram of compound **1a**. The ellipsoid contour of probability level is 50%.

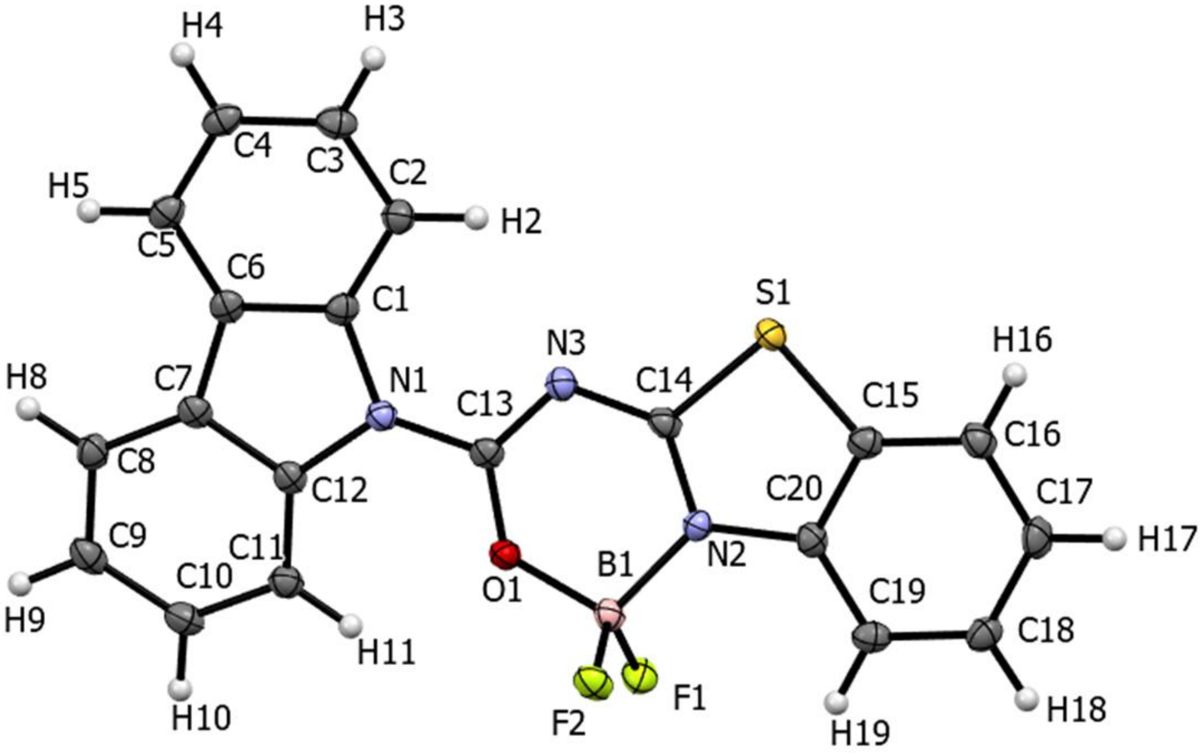

Figure S2. ORTEP diagram of compound **2a**. The ellipsoid contour of probability level is 50%.

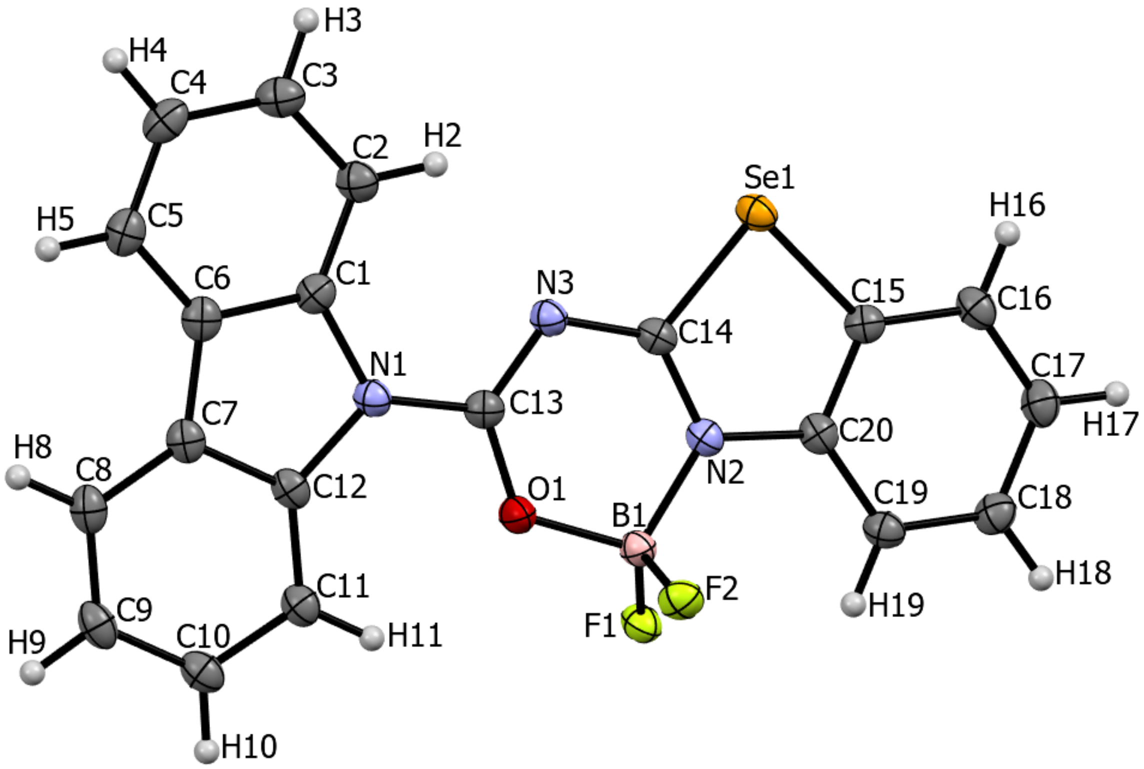

Figure S3. ORTEP diagram of compound **3a**. The ellipsoid contour of probability level is 50%.

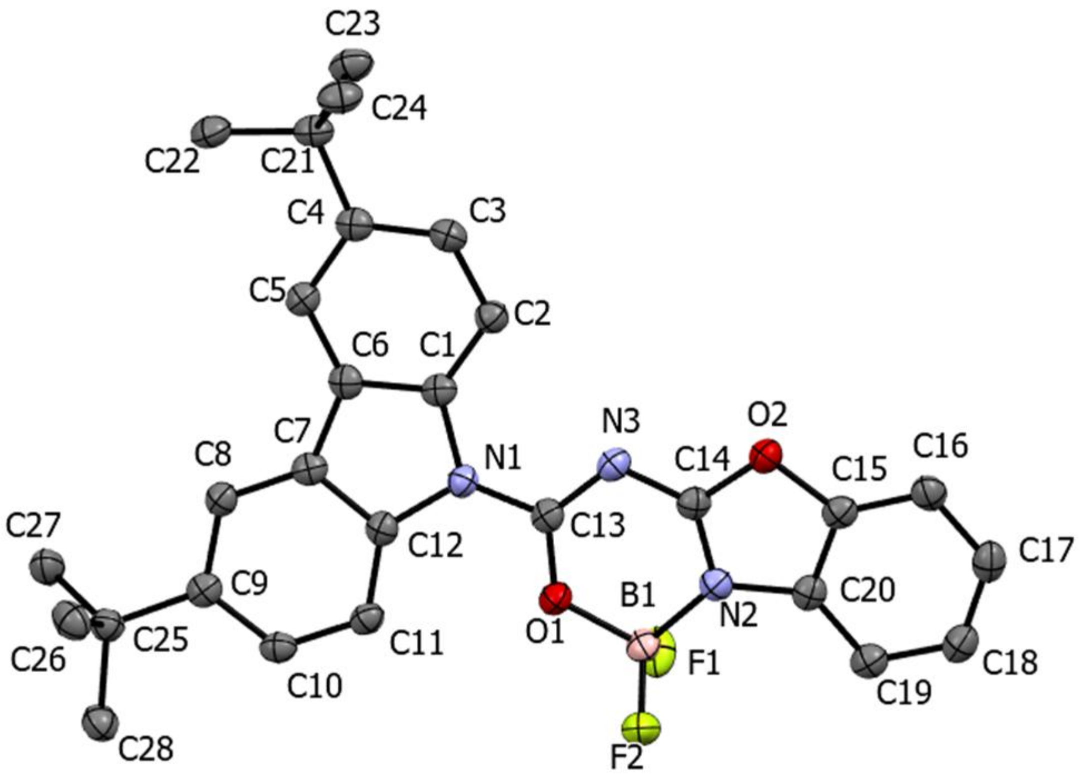

Figure S4. ORTEP diagram of compound **1b**. The ellipsoid contour of probability level is 50%. Hydrogen atoms are omitted for a clear view.

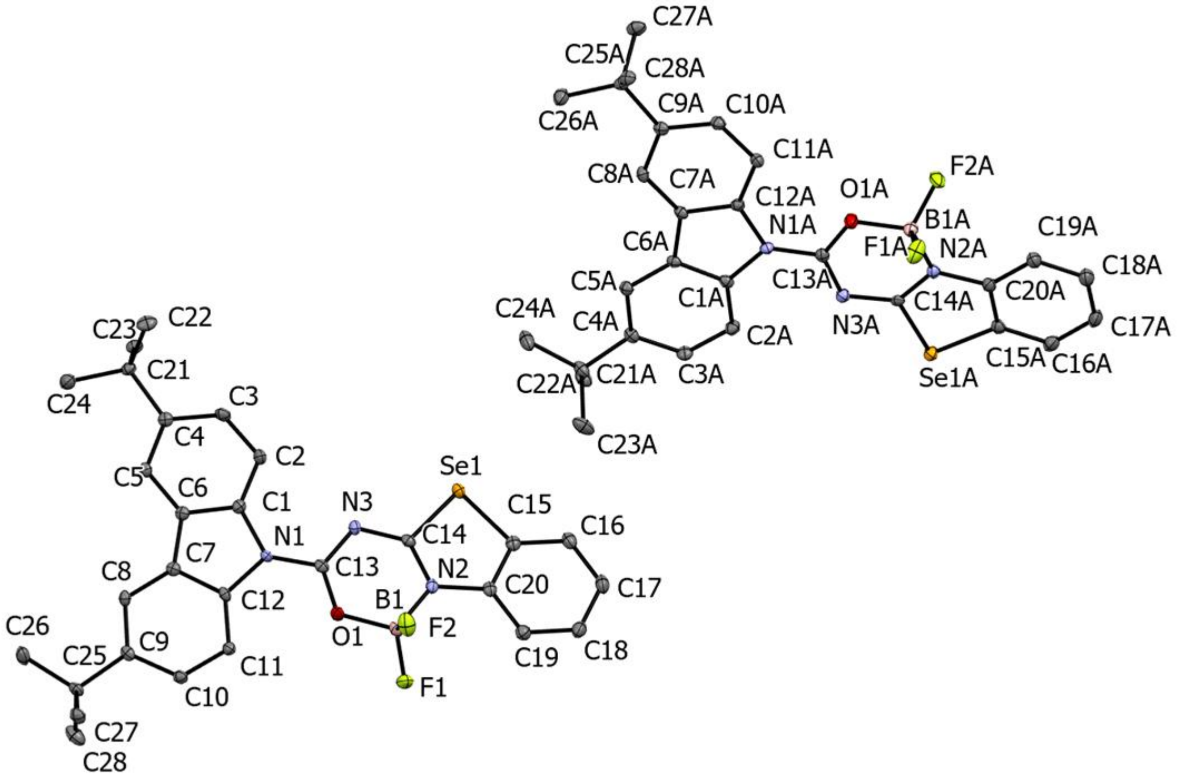

Figure S5. ORTEP diagram of compound **3b**. The ellipsoid contour of probability level is 50%. Hydrogen atoms are omitted for a clear view.

Table S2. Crystal data for compound **1a**.

Compound	**1a**	
Empirical formula	$C_{20}H_{12}BF_2N_3O_2$	
Moiety formula	$C_{20}H_{12}BF_2N_3O_2$	
Formula weight	375.158	
CCDC No.	CCDC2485478	
Wavelength	1.54184	
Crystal system	orthorhombic	
Space group	$Pna2_1$	
Unit cell dimensions	a = 12.3628(4)Å	
	b = 26.1906(7)Å	
	c = 5.00915(14)Å	
Volume	1621.91(8)$Å^3$	
Z	4	
Density Calc.	1.536 g/cm^3	
Absorption coefficient	0.116 mm^{-1}	
F(000)	768.596	
Crystal	Colourless needle	
Crystal size	0.5 × 0.08 × 0.08 mm	
Index ranges	-17≤ h ≤ 17, -36 ≤ k ≤ 36, -7 ≤ l ≤ 6	
Reflections collected (all / independent)	38274 / 4560 [R_{int} = 0.0650]	
Absorption correction	multi-scan	
Refinement method	Gauss-Newton minimization	
Restraints / parameters	1 / 253	
Goodness-of-fit on F^2	1.0467	
Final R indices [$F^2 > 2\sigma(F^2)$]	R_1 = 0.0408, wR_2 = 0.0878	
R indices (all data)	R_1 = 0.0492, wR_2 = 0.0912	

Table S3. Crystal data for compound **2a**.

Compound	**2a**	
Empirical formula	$C_{20}H_{12}BF_2N_3OS$	
Moiety formula	$C_{20}H_{12}BF_2N_3OS$	
Formula weight	391.225	
CCDC No.	CCDC2485477	
Wavelength	1.54184	
Crystal system	orthorhombic	
Space group	$Pna2_1$	
Unit cell dimensions	a = 12.2441(2) Å	
	b = 26.6599(5) Å	
	c = 5.02362(10) Å	
Volume	1639.85(6) Å^3	
Z	4	
Density Calc.	1.585 g/cm^3	
Absorption coefficient	0.237 mm^{-1}	
F(000)	801.049	
Crystal	colourless block	
Crystal size	0.3 × 0.04 × 0.04 mm	
Index ranges	$-17 \leq h \leq 16$, $-37 \leq k \leq 37$, $-7 \leq l \leq 7$	
Reflections collected (all / independent)	38320 / 4542 [R_{int} = 0.0734]	
Absorption correction	multi-scan	
Refinement method	Gauss-Newton minimisation	
Restraints / parameters	1 / 253	
Goodness-of-fit on F^2	1.0563	
Final R indices [$F^2 > 2\sigma(F^2)$]	R_1 = 0.0413, wR_2 = 0.0885	
R indices (all data)	R_1 = 0.0485, wR_2 = 0.0916	

Table S4. Crystal data for compound **3a**.

Compound	**3a**	
Empirical formula	$C_{20}H_{12}BF_2N_3OSe$	
Moiety formula	$C_{20}H_{12}BF_2N_3OSe$	
Formula weight	438.118	
CCDC No.	CCDC2485488	
Wavelength	1.54184	
Crystal system	monoclinic	
Space group	$P2_1/c$	
Unit cell dimensions	*a* = 7.75954(7)Å	
	b = 14.26981(11)Å	β = 94.5432(8) º
	c = 15.19951(13)Å	
Volume	1677.71(2) $Å^3$	
Z	4	
Density Calc.	1.735 g/cm^3	
Absorption coefficient	3.376 mm^{-1}	
F(000)	871.696	
Crystal	colourless block	
Crystal size	0.3 × 0.3 × 0.1 mm	
Index ranges	-9≤ h ≤ 8, -17 ≤ k ≤ 17, -18 ≤ l ≤ 18	
Reflections collected (all / independent)	30920 / 3218 [R_{int} = 0.0790]	
Absorption correction	multi-scan	
Refinement method	Gauss-Newton minimisation	
Restraints / parameters	0 / 278	
Goodness-of-fit on F^2	1.0505	
Final R indices [$F^2 > 2\sigma(F^2)$]	R_1 = 0.0347, wR_2 = 0.0900	
R indices (all data)	R_1 = 0.0356, wR_2 = 0.0910	

Table S5. Crystal data for compound **1b**.

Compound	**1b**	
Empirical formula	$C_{28}H_{28}BF_2N_3O_2$	
Moiety formula	$C_{28}H_{28}BF_2N_3O_2$	
Formula weight	487.374	
CCDC No.	CCDC2491457	
Wavelength	1.54184	
Crystal system	monoclinic	
Space group	$P2_1/n$	
Unit cell dimensions	a = 9.7532(4) Å	
	b = 24.1878(12)Å	β = 103.967(4)°
	c = 10.4785(5)Å	
Volume	2398.89(19)Å^3	
Z	4	
Density Calc.	1.349 g/cm^3	
Absorption coefficient	0.785 mm^{-1}	
F(000)	1027.603	
Crystal	Colourless needle	
Crystal size	0.5 × 0.03 × 0.02 mm	
Index ranges	$-11 \leq h \leq 10, -29 \leq k \leq 29, -12 \leq l \leq 12$	
Reflections collected (all / independent)	13804 / 4563 [R_{int} = 0.1089]	
Absorption correction	multi-scan	
Refinement method	Gauss-Newton minimisation	
Restraints / parameters	0 / 331	
Goodness-of-fit on F^2	1.0317	
Final R indices [$F^2 > 2\sigma(F^2)$]	R_1 = 0.0642, wR_2 = 0.1678	
R indices (all data)	R_1 = 0.0896, wR_2 = 0.1947	

Table S6. Crystal data for compound **3b**.

Compound	**3b**	
Empirical formula	$2(C_{28}H_{28}BF_2N_3OSe)$	
Moiety formula	$C_{28}H_{28}BF_2N_3OSe$	
Formula weight	1100.669	
CCDC No.	CCDC2485479	
Wavelength	1.54184	
Crystal system	triclinic	
Space group	*P-1*	
Unit cell dimensions	*a* = 9.7850(2) Å	α = 71.252(2) °
	b = 13.3357(3) Å	β = 88.9935(19) °
	c = 20.0199(5) Å	γ = 87.4552(19) °
Volume	2471.33(11) $Å^3$	
Z	2	
Density Calc.	1.479 g/cm^3	
Absorption coefficient	1.562 mm^{-1}	
F(000)	1128.649	
Crystal	colourless block	
Crystal size	0.4 × 0.1 × 0.05 mm	
Index ranges	$-13 \leq h \leq 13$, $-18 \leq k \leq 18$, $-27 \leq l \leq 28$	
Reflections collected (all / independent)	60642 / 13354 [R_{int} = 0.0444]	
Absorption correction	multi-scan	
Refinement method	Gauss-Newton minimisation	
Restraints / parameters	0 / 661	
Goodness-of-fit on F^2	1.0534	
Final R indices [$F^2 > 2\sigma(F^2)$]	R_1 = 0.0458, wR_2 = 0.1000	
R indices (all data)	R_1 = 0.0588, wR_2 = 0.1053	

Table S7. Selected geometrical parameters of structures of compounds **1a**, **2a**, **3a**, **1b**, and **3b**.

**Molecule**	**1a**	**1b**	**2a**	**3a**	**Conformer 3b**	**Conformer 3b-A**
The B1-N2 bond distances (Å)	1.547(2)	1.551(3)	1.560(3)	1.552(3)	1.571(4)	1.572(4)
The B1-O1 bond distances (Å)	1.496(2)	1.500(4)	1.490(3)	1.486(3)	1.493(4)	1.490(4)
The B1-F1 and B1-F2 bond distances (Å)	1.370(2)<br>1.375(2)	1.367(4)<br>1.370(4)	1.371(3)<br>1.384(2)	1.376(3)<br>1.377(3)	1.366(3)<br>1.376(4)	1.379(3)<br>1.363(4)
The O1-C13 bond distances (Å)	1.303(2)	1.306(3)	1.304(2)	1.306(2)	1.298(3)	1.298(4)
The C13-N3 bond distances (Å)	1.326(2)	1.321(3)	1.319(2)	1.317(2)	1.329(4)	1.329(3)
The C14-N3 bond distances (Å)	1.328(2)	1.319(3)	1.339(2)	1.339(2)	1.340(4)	1.336(4)
The C14-N2 bond distances (Å)	1.329(2)	1.324(4)	1.335(2)	1.332(2)	1.327(3)	1.331(4)
The N2-C20 bond distances (Å)	1.401(2)	1.397(3)	1.406(2)	1.401(2)	1.409(4)	1.406(4)
The C20-C15 bond distances (Å)	1.389(2)	1.384(4)	1.404(3)	1.396(3)	1.404(4)	1.400(4)
The C15-O2/S1/Se1 bond distances (Å)	1.400(2)	1.399(4)	1.749(2)	1.890(2)	1.883(2)	1.882(3)
The O2/S1/Se1-C14 bond distances (Å)	1.353(2)	1.349(3)	1.729(2)	1.879(2)	1.879(3)	1.880(3)
The N1-C13 bond distances (Å)	1.360(2)	1.354(3)	1.362(2)	1.361(2)	1.360(4)	1.360(4)
The F-B-F angle (deg)	111.3(1)	111.7(2)	110.6(2)	110.5(2)	111.7(3)	111.6(2)
The N-B-F angles (deg)	110.7(1)<br>111.0(1)	110.0(2)<br>111.4(2)	111.2(2)<br>109.9(2)	110.2(2)<br>111.3(2)	111.5(2)<br>109.3(2)	109.0(2)<br>111.2(2)
The angles O-B-F (deg)	109.5(1)<br>109.6(1)	109.0(2)<br>109.1(2)	109.3(2)<br>109.3(2)	109.3(2)<br>108.7(2)	109.0(2)<br>108.9(2)	108.9(2)<br>109.3(2)
The O-B-N angle (deg)	104.5(1)	105.4(2)	106.5(2)	106.7(2)	106.2(2)	106.7(2)
The B1-O1-C13 angle (deg)	124.6(1)	124.9(2)	123.5(2)	123.9(2)	124.2(2)	123.6(2)
The O1-C13-N3 angle (deg)	125.9(1)	125.5(2)	125.7(2)	125.7(2)	125.3(3)	125.2(3)
The C13-N3-C14 angle (deg)	113.8(1)	114.5(2)	115.7(2)	115.9(2)	115.8(2)	116.3(2)
The N3-C14-N2 angle (deg)	128.0(1)	129.0(3)	126.8(2)	126.5(2)	127.2(3)	126.9(3)
The C14-N2-B1 angle (deg)	120.4(1)	119.7(2)	119.1(2)	119.7(2)	119.1(2)	118.7(2)
The C14-N2-C20 angle (deg)	107.3(1)	107.3(2)	112.7(2)	115.4(2)	115.2(2)	114.9(2)
The N2-C20-C15 angle (deg)	106.3(1)	106.4(2)	112.8(2)	115.0(2)	114.8(2)	115.1(2)
The C20-C15-O2/S1/Se1 angle (deg)	108.6(1)	108.5(2)	110.3(1)	110.5(1)	110.4(2)	110.5(2)
The C15-O2/S1/Se1-C14 angle (deg)	105.3(1)	105.2(2)	90.27(9)	85.69(9)	86.0(1)	85.9(1)
The O2/S1/Se1-C14-N2 angle (deg)	112.5(1)	112.5(2)	114.0(1)	113.3(1)	113.5(2)	113.5(2)
The C1-N1-C13-N3 torsion angle (deg)	4.8(2)	6.1(4)	-4.3(3)	3.7(3)	-2.6(4)	3.7(4)
The C12-N1-C13-O1 torsion angle (deg)	0.4(2)	6.5(4)	1.0(3)	3.4(3)	-3.2(4)	1.9(4)

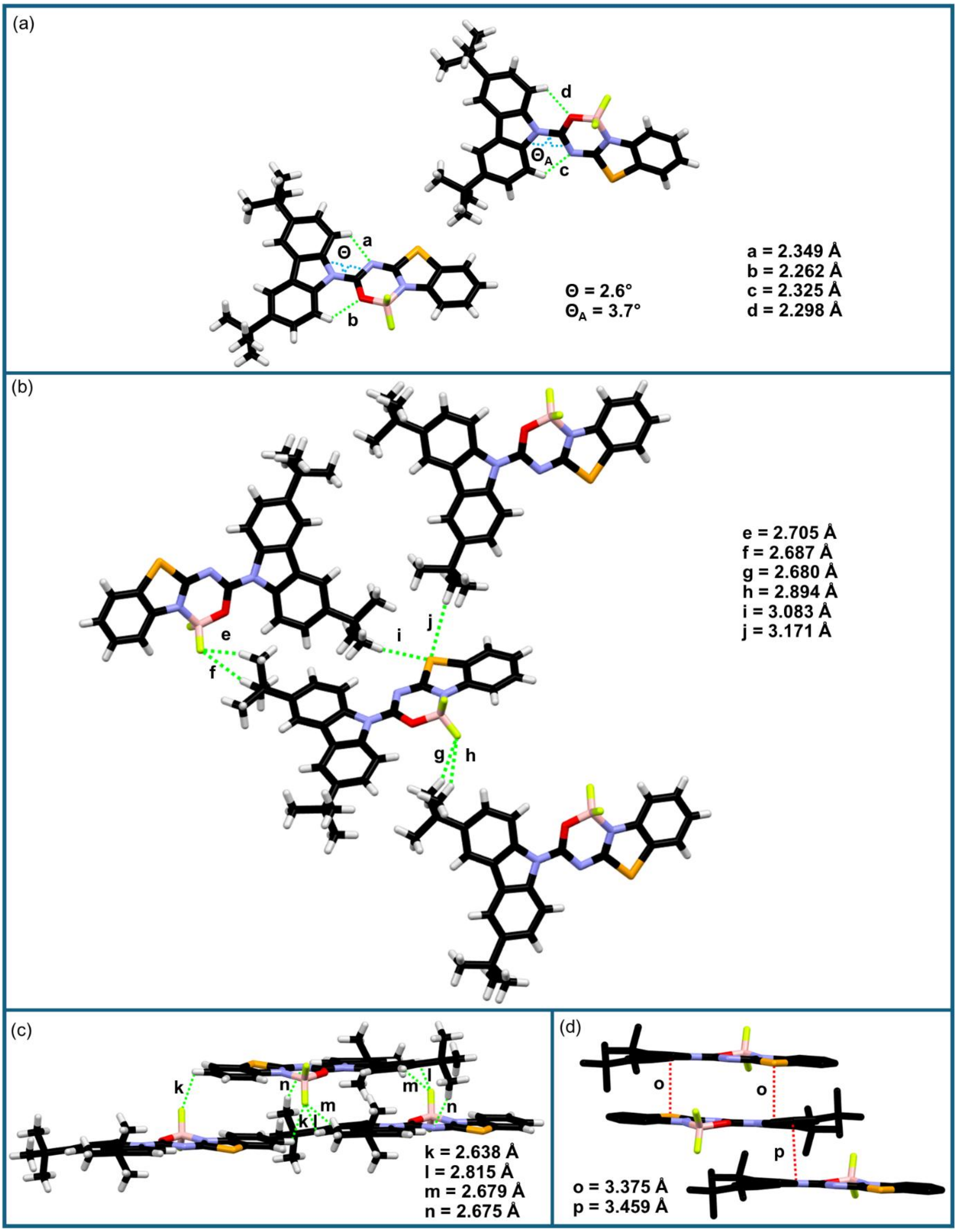

Figure S6. X-ray molecular structure of dyes **3b**, showing intramolecular CH···N and CH···O hydrogen bonds (green dashed lines). Fragments of the crystal packing of structure **3b** showing: intermolecular CH···F and CH···Se hydrogen bonds (green dashed lines) (b); intermolecular CH···F and CH···N hydrogen bonds (green dashed lines) (c); intermolecular π···π/n···π interactions (red dashed lines) (d) [hydrogen atoms are omitted for a clear view in (d)].

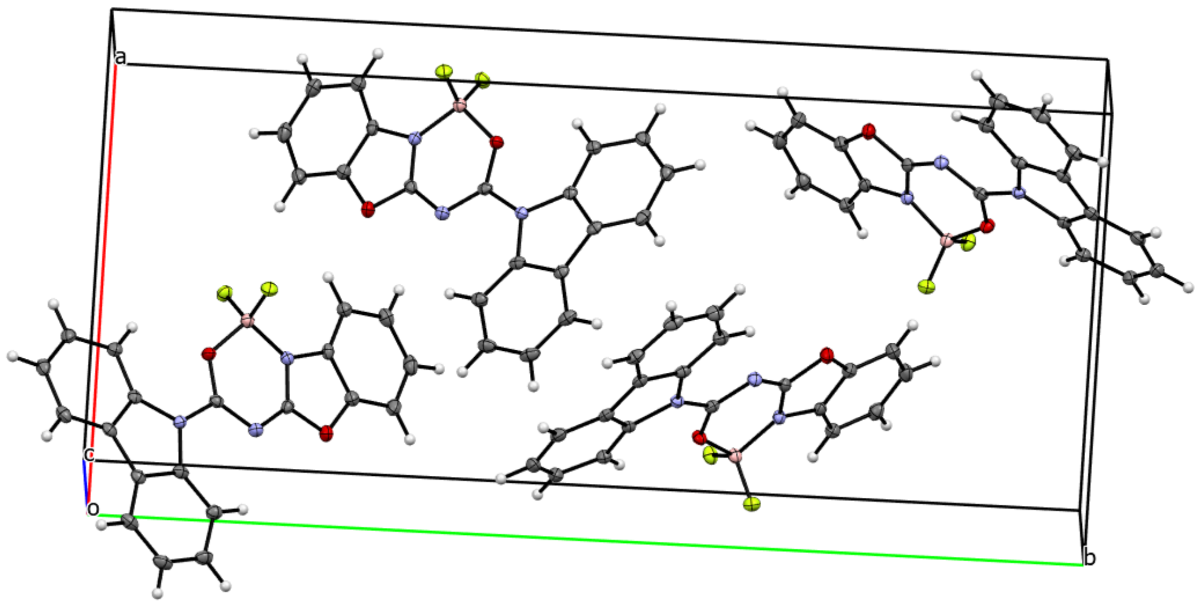

Figure S7. Unit cell of the crystal structure of complex **1a**.

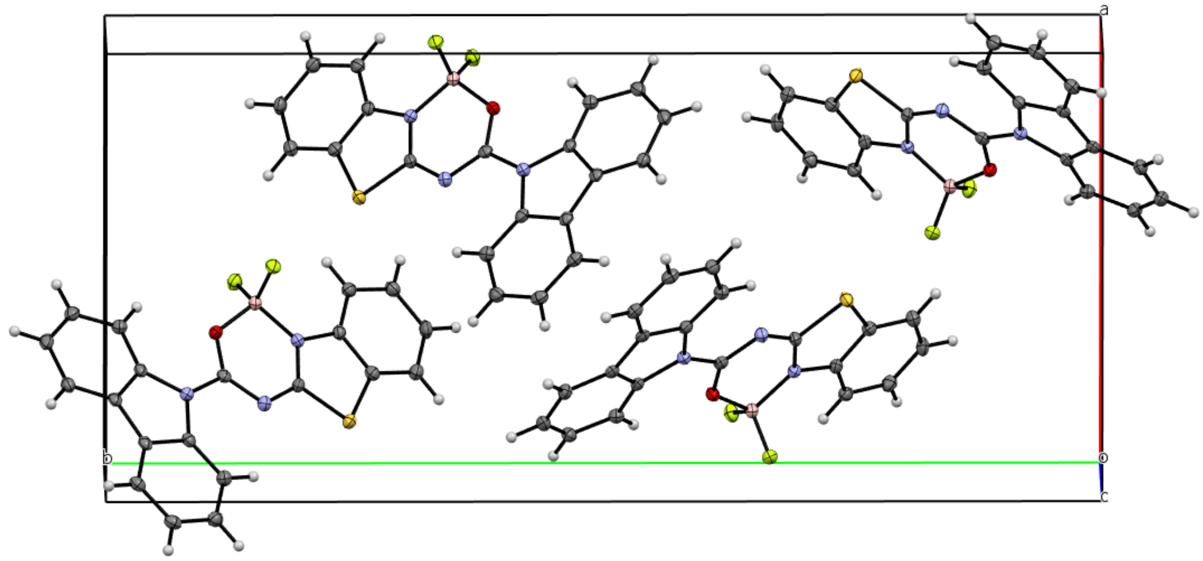

Figure S8. Unit cell of the crystal structure of complex **2a**.

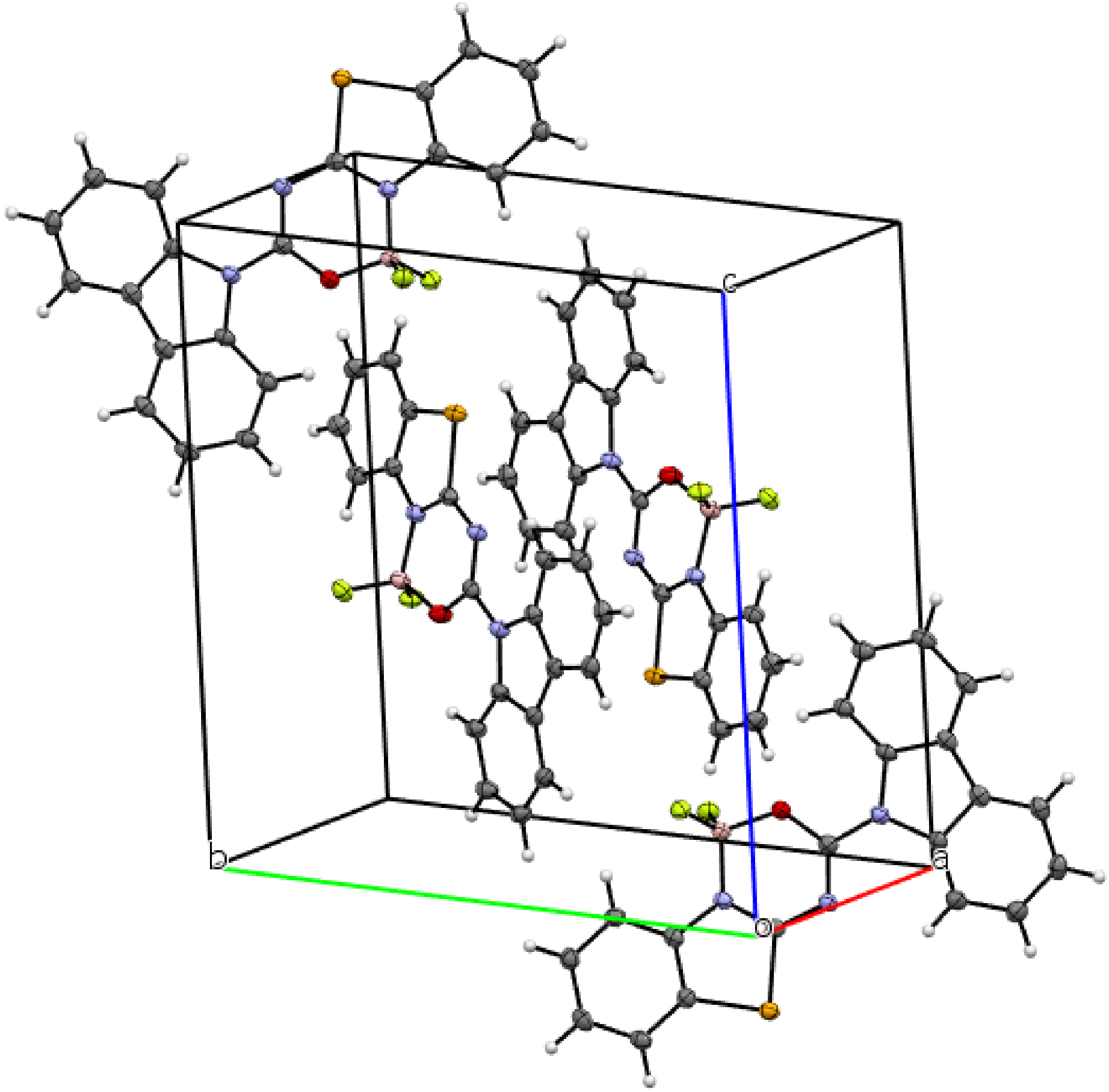

Figure S9. Unit cell of the crystal structure of complex **3a**.

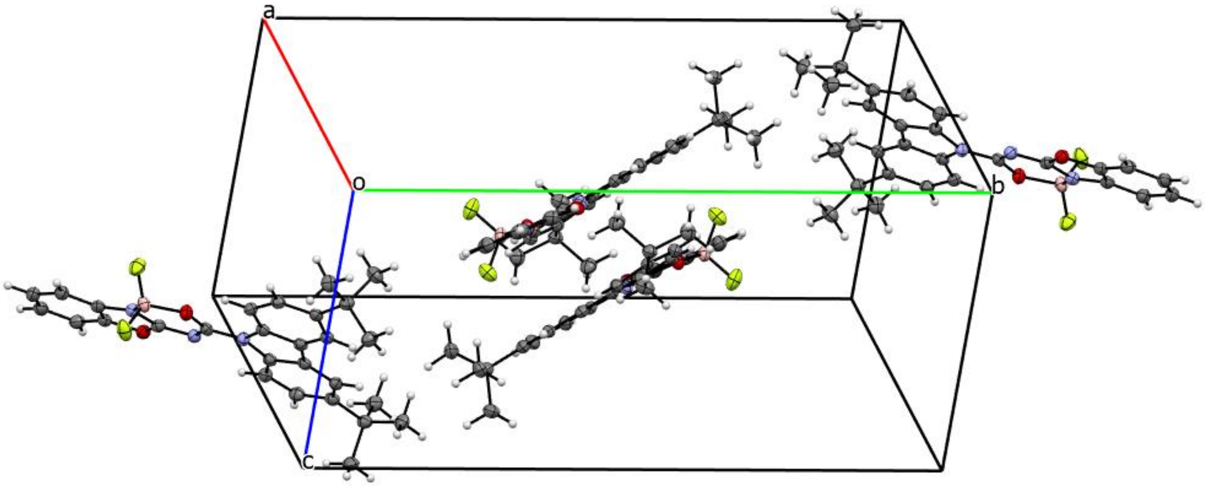

Figure S10. Unit cell of the crystal structure of complex **1b**.

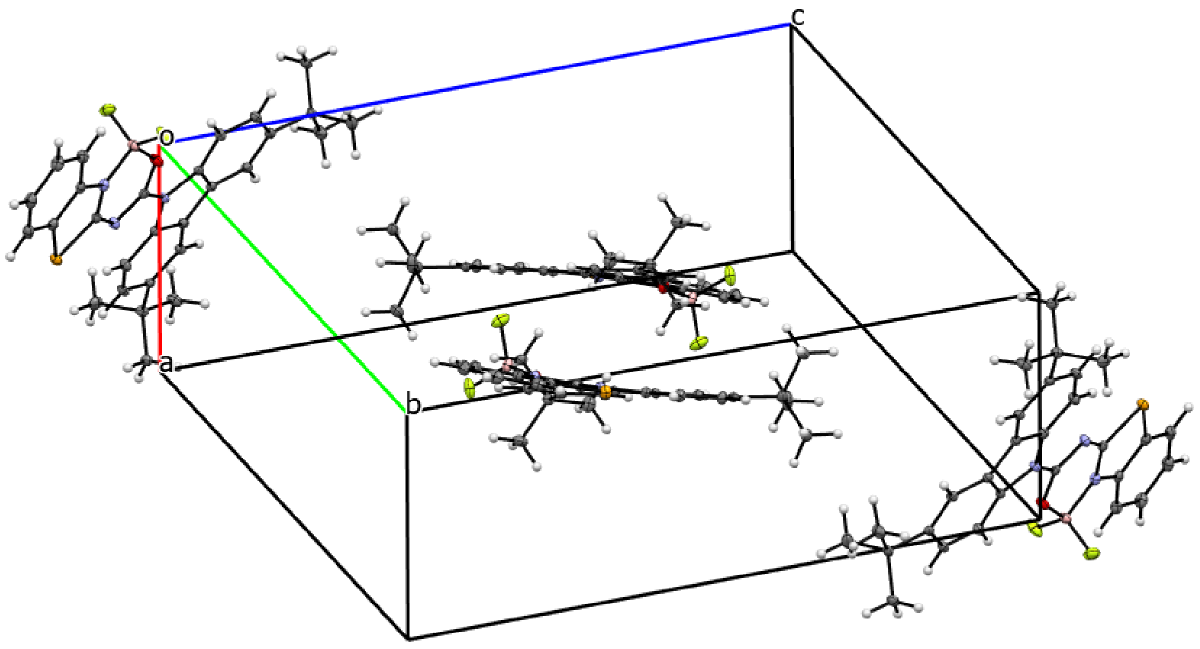

Figure S11. Unit cell of the crystal structure of complex **3b**.

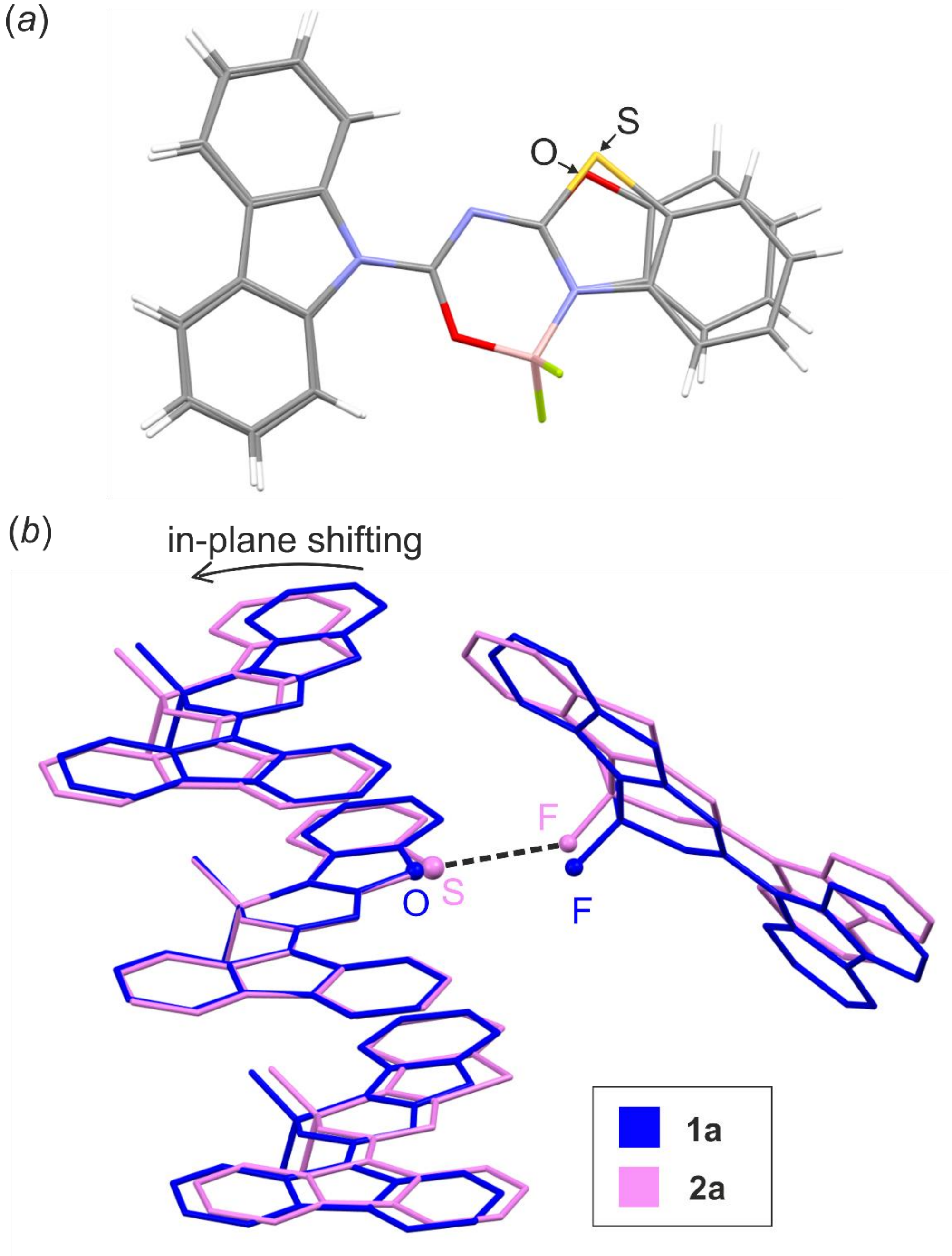

Figure S12. Overlay of the molecules (a) and supramolecular motifs (b) (π-stacking and σ-hole interactions) in isostructural crystals **1a** and **2a**.

3. Theoretical Calculations

Table S8. Calculated properties of the 12 lowest singlet excited states for complexes **1a,b**, **2a,b**, and **3a,b** determined through TD-DFT.

Comp.	Transition	Energy (eV)	Wavelength (nm)	Oscillator strength	Expansion coefficients for single-electron excitations*
**1a**	$S_0 \rightarrow S_1$	3.7612	329.64	0.5973	0.65970 H→L/ 0.23898 H-1→L
	$S_0 \rightarrow S_2$	3.7789	328.09	0.4447	-0.23817 H→L/ 0.65907 H-1→L
	$S_0 \rightarrow S_3$	4.4735	277.15	0.0300	0.28395 H-2→L/ -0.21653 H-1→L+1/ 0.21304 H-1→L+4/0.54855 H→L+2
	$S_0 \rightarrow S_4$	4.5491	272.54	0.0359	-0.24874 H-3→L / 0.52258 H-2→L
	$S_0 \rightarrow S_5$	4.6236	268.15	0.1617	-0.18347 H-1→L+1 / -0.33542 H→L+1/ 0.34179 H-3→L / 0.35775 H-2→L/ -0.47151 H-1→L+1
	$S_0 \rightarrow S_6$	4.8446	255.92	0.0553	0.61572 H-4→L / -0.21840 H-3→L / -0.13692 H→L+2/-0.10984 H→L+3
	$S_0 \rightarrow S_7$	4.8662	254.79	0.2495	0.23098 H-4→L / 0.49797 H-3→L / -0.35952 H-1→L+1 / -0.15650 H→L+1 / 0.18015 H→L+4
	$S_0 \rightarrow S_8$	5.1902	238.88	0.0077	0.17739 H-4→L / -0.20178 H-4→L+3 / -0.11281 H-2→L+1 / 0.12480 H-2→L +2/ -0.16602 H-1→L+2 / 0.59658 H→L+2
	$S_0 \rightarrow S_9$	5.2219	237.43	0.0040	-0.18891 H-3→L+1 / 0.45202 H-2→L+1/ -0.14493 H-1→L+1 / 0.32843 H-1→L+2 / -0.20432 H-1→L+4 / 0.19916 H→L+3
	$S_0 \rightarrow S_{10}$	5.2719	235.18	0.0013	-0.20300 H-3→L+1 / -0.35431 H-2→L+1 / 0.47558 H-1→L+2 / 0.21417 H-1→L+4/ -0.13609 H→L+1
	$S_0 \rightarrow S_{11}$	5.4670	226.79	0.0000	0.45412 H-6→L
	$S_0 \rightarrow S_{12}$	5.4890	225.88	0.0072	-0.58585 H-5→L / 0.14426 H-2→L+1 / 0.27790 H-1→L+4 / 0.16740 H-2→L+3

*H – HOMO. L – LUMO.

Table S8. (continues).

Comp.	Transition	Energy (eV)	Wavelength (nm)	Oscillator strength	Expansion coefficients for single-electron excitations*
**2a**	$S_0 \rightarrow S_1$	3.5612	348.15	0.9625	0.70195 H→L
	$S_0 \rightarrow S_2$	3.6097	343.47	0.0518	0.70214 H-1→L
	$S_0 \rightarrow S_3$	4.3465	285.25	0.0441	-0.34250 H-3→L/ 0.58119 H-2→L
	$S_0 \rightarrow S_4$	4.3763	283.31	0.0349	0.58830 H-3→L/ 0.33810 H-2→L/ 0.10630 H→L+2
	$S_0 \rightarrow S_5$	4.4412	279.17	0.0050	-0.10537 H-4→L+1/ -0.12255 H-2→L/ 0.19202 H-1→L+5/ 0.64504 H→L+1
	$S_0 \rightarrow S_6$	4.5342	273.44	0.0507	0.54903 H-4→L/ -0.12632 H-2→L/ 0.39622 H-1→L+1
	$S_0 \rightarrow S_7$	4.7730	259.76	0.3902	-0.40465 H-4→L/ 0.52742 H-1→L+1/ -0.19627 H-1→L+5
	$S_0 \rightarrow S_8$	5.0124	247.36	0.0031	0.10728 H-2→L+3/ -0.23133 H-2→L+4/ -0.18984 H→L+2/ -0.25008 H→L +3/ 0.55609 H→L+4
	$S_0 \rightarrow S_9$	5.0435	245.83	0.0322	-0.13959 H-3→L/ -0.10234 H-3→L+2/ 0.16348 H-3→L+3/ -0.11895 H-1→L+2/ 0.58417 H→L+2/ 0.21990 H→L+4
	$S_0 \rightarrow S_{10}$	5.0802	244.05	0.0004	0.69167 H6→L
	$S_0 \rightarrow S_{11}$	5.1795	239.38	0.0066	0.11967 H-4→L+1/ 0.55695 H-2→L+1/ -0.10597 H-1→L+1/ 0.14563 H-1→L+3/ 0.27436 H-4→L+5/ 0.16139 H→L+1
	$S_0 \rightarrow S_{12}$	5.2571	235.84	0.0040	0.27197 H-4→L+1/ -0.16316 H-2→L+1/ 0.47800 H-1→L+2/ 0.30541 H-1→L+3/ 0.11703 H-1→L+4/ -0.10732 H-1→L+5/ -0.14948 H-1→L+6
**3a**	$S_0 \rightarrow S_1$	3.5247	351.75	0.9523	0.70241 H→L
	$S_0 \rightarrow S_2$	3.5833	346.01	0.0414	0.70271 H-1→L
	$S_0 \rightarrow S_3$	4.1951	295.54	0.0456	-0.36767 H-3→L/ 0.58142 H-2→L
	$S_0 \rightarrow S_4$	4.3191	287.06	0.0349	0.57790 H-3→L/ 0.35929 H-2→L
	$S_0 \rightarrow S_5$	4.3873	282.59	0.0027	-0.28156 H-2→L+2/ -0.63351 H→L+2
	$S_0 \rightarrow S_6$	4.4350	279.56	0.0046	-0.10749 H-4→L/ 0.19457 H-1→L+5/ 0.64459 H→L+1
	$S_0 \rightarrow S_7$	4.5128	274.74	0.0320	0.56903 H-4→L/ -0.37403 H-1→L+14
	$S_0 \rightarrow S_8$	4.7634	260.29	0.4085	0.37883 H-4→L/ 0.54654 H-1→L+1/ -0.19391 H→L+4
	$S_0 \rightarrow S_9$	4.8490	255.69	0.0003	0.70167 H-1→L+2
	$S_0 \rightarrow S_{10}$	4.8704	254.57	0.0005	0.48172 H-3→L+2/ -0.46235 H-2→L+2/ -0.19211 H→L+2
	$S_0 \rightarrow S_{11}$	4.9705	249.44	0.0006	0.10061 H-7→L/ 0.68754 H-6→L
	$S_0 \rightarrow S_{12}$	5.0491	245.56	0.0390	-0.16646 H-3→L+3/ 0.13959 H-3→L+4/ 0.12154 H-2→L+1/ -0.16323 H-2→L+4/ 0.59497 H→L+3/ 0.10005 H→L+4

*H – HOMO. L – LUMO.

Table S8. (continues).

Comp.	Transition	Energy (eV)	Wavelength (nm)	Oscillator strength	Expansion coefficients for single-electron excitations*
**1b**	$S_0 \rightarrow S_1$	3.6776	337.13	1.0842	0.69936 H→L
	$S_0 \rightarrow S_2$	3.7483	330.78	0.0210	0.70074 H-1→L
	$S_0 \rightarrow S_3$	4.3658	283.99	0.0090	0.10293 H-2→L/ -0.20854 H-1→L+4/ 0.65311 H→L+1
	$S_0 \rightarrow S_4$	4.4399	279.25	0.0272	0.38605 H-3→L/ 0.46575 H-2→L/ 0.32754 H-1→L+1
	$S_0 \rightarrow S_5$	4.5573	272.05	0.1623	-0.38066 H-3→L / 0.50087 H-2→L/ -0.26849 H-1→L+1
	$S_0 \rightarrow S_6$	4.7327	261.97	0.4623	-0.43299 H-3→L+1/ 0.50994 H-1→L+1/ 0.18708 H→L+4
	$S_0 \rightarrow S_7$	4.8728	254.44	0.0092	0.63250 H-4→L/ -0.11954 H-2→L+2/ -0.20843 H→L+2/ -0.12946 H-1→L+3
	$S_0 \rightarrow S_8$	5.0960	243.29	0.0045	0.23119 H-4→L/ -0.14798 H-4→L+3/ 0.63553 H→L+2
	$S_0 \rightarrow S_9$	5.1550	240.51	0.0113	-0.29503 H-3→L+1/ -0.34151 H-2→L+1/ -0.14414 H-1→L+1/ 0.46232 H-1→L+2/ -0.11867 H-1→L+3/ 0.13932 H-1→L+5/ 0.48172 H-3→L+2/ -0.46235 H-2→L+2
	$S_0 \rightarrow S_{10}$	5.2415	236.54	0.0128	-0.20526 H-2→L/ 0.48289 H-2→L/ 0.28501 H-1→L+2/ -0.29855 H-1→L+4/ 0.14575 H→L+1
	$S_0 \rightarrow S_{11}$	5.3549	231.54	0.6968	-0.18532 H-3→L/ -0.16949 H-1→L/ -0.10577 H→L+2/ 0.63139 H→L+4
	$S_0 \rightarrow S_{12}$	5.3864	230.18	0.0562	-0.10439 H-5→L / 0.12849 H-4→L / 0.14464 H-2→L+1 / -0.12231 H-1→L+4/ 0.63709 H→L+3

*H – HOMO. L – LUMO.

Table S8. (continues).

Comp.	Transition	Energy (eV)	Wavelength (nm)	Oscillator strength	Expansion coefficients for single-electron excitations*
**2b**	$S_0 \rightarrow S_1$	3.4722	357.08	1.0622	0.70211 H→L
	$S_0 \rightarrow S_2$	3.5810	346.23	0.0099	0.70338 H-1→L
	$S_0 \rightarrow S_3$	4.2838	289.43	0.0688	-0.24450 H-3→L/ 0.63240 H-2→L/ 0.14162 H-1→L+2
	$S_0 \rightarrow S_4$	4.3275	286.50	0.0024	0.12784 H-3→L/ -0.19476 H-1→L+5/ 0.65394 H→L+2
	$S_0 \rightarrow S_5$	4.3866	282.65	0.0319	0.47921 H-4→L/ -0.41923 H-3→L/ -0.16228 H-2→L/ 0.16155 H-1→L+1/ -0.10602 H→L+2
	$S_0 \rightarrow S_6$	4.4066	281.36	0.0413	0.47533 H-4→L/ 0.40159 H-3→L/ 0.23323 H-2→L/ -0.16521 H-1→L+1
	$S_0 \rightarrow S_7$	4.6750	265.21	0.5025	0.27382 H-3→L/ 0.60374 H-1→L+1/ 0.18579 H→L+5
	$S_0 \rightarrow S_8$	4.9406	250.95	0.0108	0.11218 H-4→L+3/ -0.11919 H-2→L+3/ -0.16655 H-2→L+4/ -0.38001 H→L+2/ 0.28441 H→L+3/ 0.44082 H→L+4
	$S_0 \rightarrow S_9$	4.9575	250.09	0.0215	0.13181 H-4→L/ -0.14492 H-2→L+4/ 0.52177 H→L+2/ 0.12063 H→L+3/ 0.36924 H→L+4
	$S_0 \rightarrow S_{10}$	5.0905	243.56	0.0004	-0.13546 H-9→L / 0.68112 H-6→L
	$S_0 \rightarrow S_{11}$	5.1339	241.50	0.0169	-0.26879 H-3→L+1/ 0.46576 H-2→L+1/ -0.13698 H-1→L+1/ -0.14609 H-1→L+2/ 0.25208 H-1→L+3/ -0.13051 H-1→L+4/ -0.18129 H-1→L+5/ 0.14900 H→L+6
	$S_0 \rightarrow S_{12}$	5.1997	238.44	0.0063	0.26659 H-3→L+1/ 0.28651 H-2→L+1/ 0.39515 H-1→L+2/ -0.17980 H-1→L+3/ -0.22244 H-1→L+5/ -0.10122 H-1→L+6/ -0.11005 H→L+1/ -0.20336 H→L+3/ 0.11076 H→L+4

*H – HOMO. L – LUMO.

Table S8. (continues).

Comp.	Transition	Energy (eV)	Wavelength (nm)	Oscillator strength	Expansion coefficients for single-electron excitations*
**3b**	$S_0 \rightarrow S_1$	3.4722	360.35	1.0493	0.70198 H→L
	$S_0 \rightarrow S_2$	3.5546	348.80	0.0101	0.70348 H-1→L
	$S_0 \rightarrow S_3$	4.1985	295.31	0.0501	-0.10057 H-4→L/ 0.28412 H-3→L/ 0.61600 H-2→L
	$S_0 \rightarrow S_4$	4.2754	290.00	0.0477	0.21396 H-4→L/ -0.60220 H-3→L/ -0.24090 H→L+2/ -0.11408 H-1→L+1
	$S_0 \rightarrow S_5$	4.3188	287.08	0.0018	0.17317 H-4→L/ -0.20121 H-2→L+2/ 0.12677 H-1→L+5/ 0.44479 H→L+1/ 0.44843 H→L+2
	$S_0 \rightarrow S_6$	4.3283	286.45	0.0008	0.18946 H-2→L+2/ 0.14402 H-1→L+5/ 0.45712 H→L+1/ -0.45646 H→L+2
	$S_0 \rightarrow S_7$	4.3667	283.93	0.0299	0.57368 H-4→L/ -0.17311 H-3→L/ 0.20206 H-2→L/ -0.21658 H-1→L+1/ -0.18634 H→L+1
	$S_0 \rightarrow S_8$	4.6698	265.50	0.5107	0.25607 H-4→L/ 0.61142 H-1→L+1/ -0.18254 H→L+5
	$S_0 \rightarrow S_9$	4.7888	258.90	0.0002	0.70572 H-1→L+2
	$S_0 \rightarrow S_{10}$	4.8434	255.98	0.0003	-0.51811 H-3→L+2/ 0.41045 H-2→L+2/ 0.21589 H→L+2
	$S_0 \rightarrow S_{11}$	4.9735	249.29	0.0377	-0.13135 H-6→L/ -0.11126 H-3→L/ -0.14073 H-3→L+4/ -0.11303 H-2→L+3/ 0.62352 H→L+3
	$S_0 \rightarrow S_{12}$	4.9836	248.78	0.0020	-0.10794 H-10→L/ 0.67022 H-6→L/ 0.12584 H→L+3

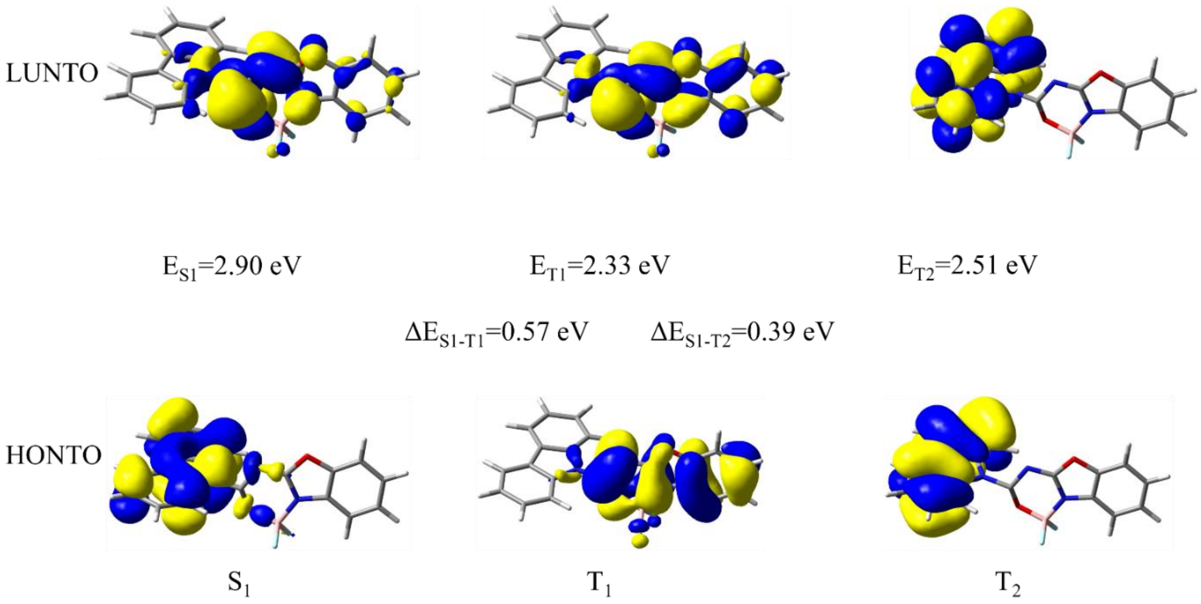

Figure S13. NTO analysis for first singlet and first two triplet excited states of compound **1a**.

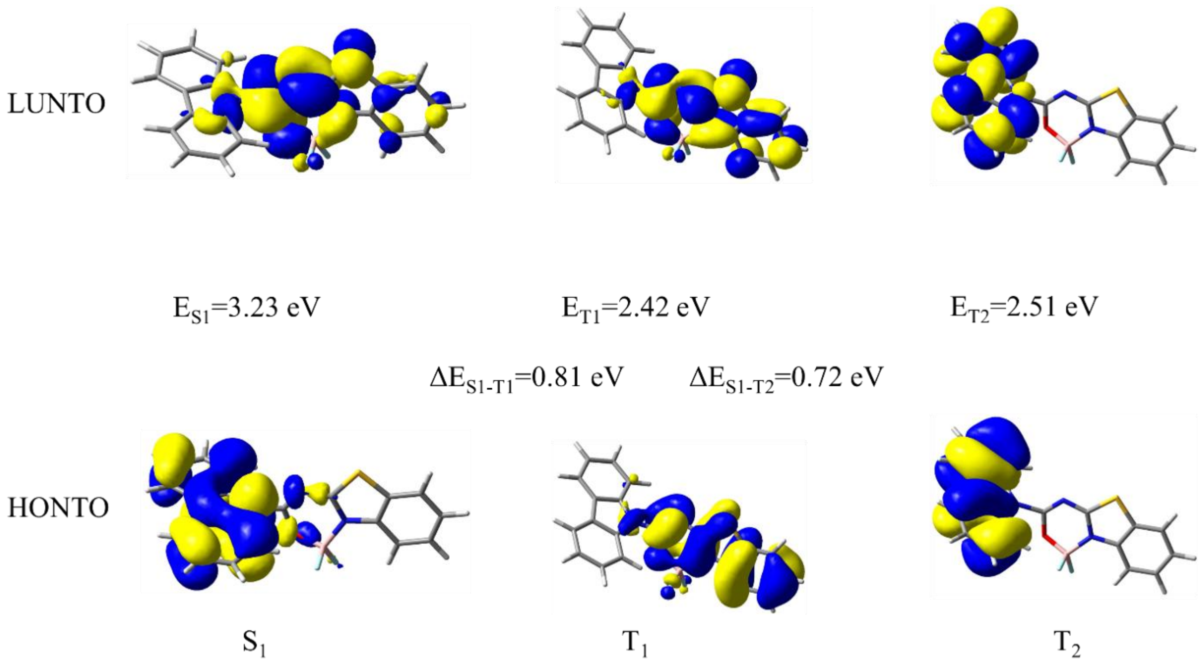

Figure S14. NTO analysis for first singlet and first two triplet excited states of compound **2a**.

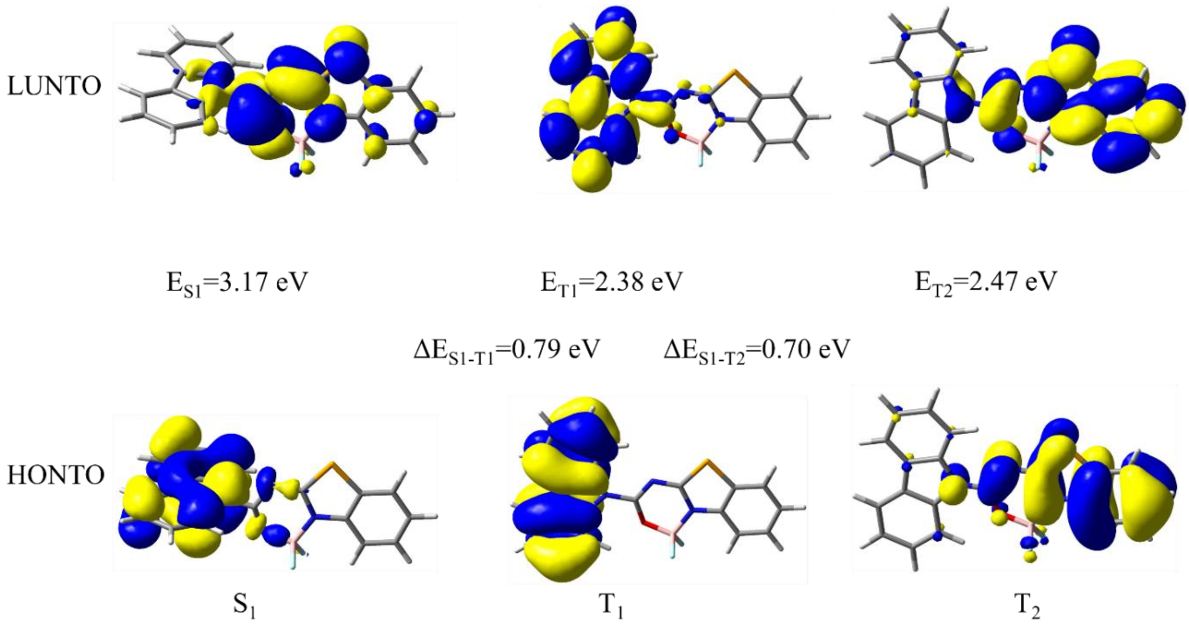

Figure S15. NTO analysis for first singlet and first two triplet excited states of compound **3a**.

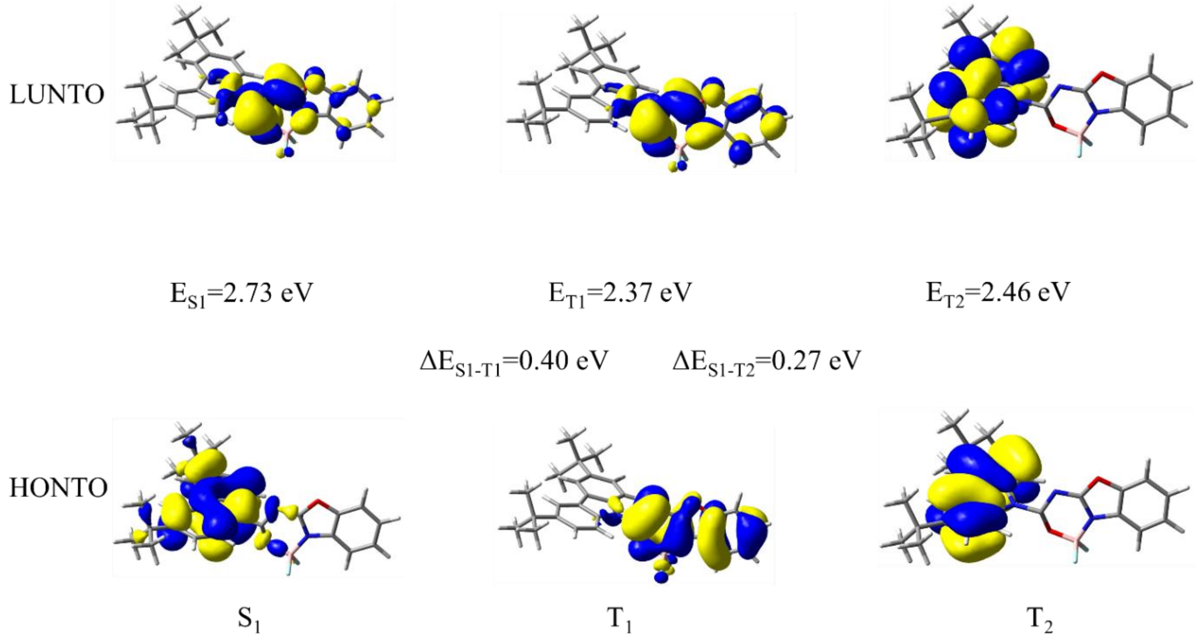

Figure S16. NTO analysis for first singlet and first two triplet excited states of compound **1b**.

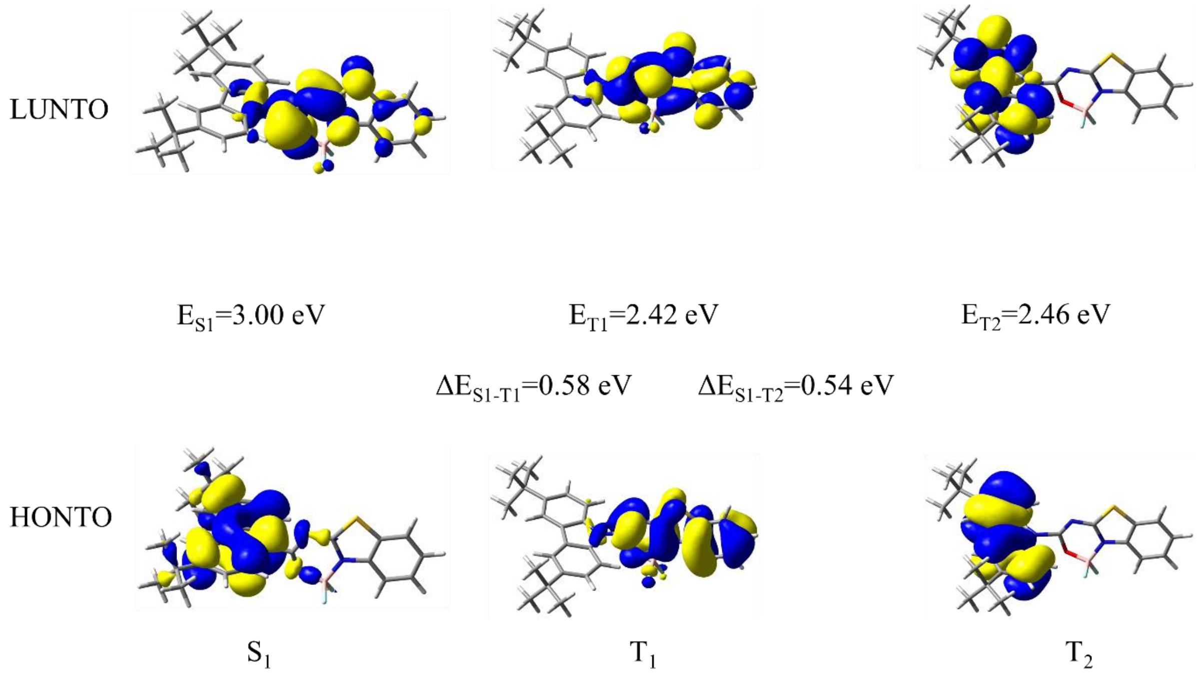

Figure S17. NTO analysis for first singlet and first two triplet excited states of compound **2b**.

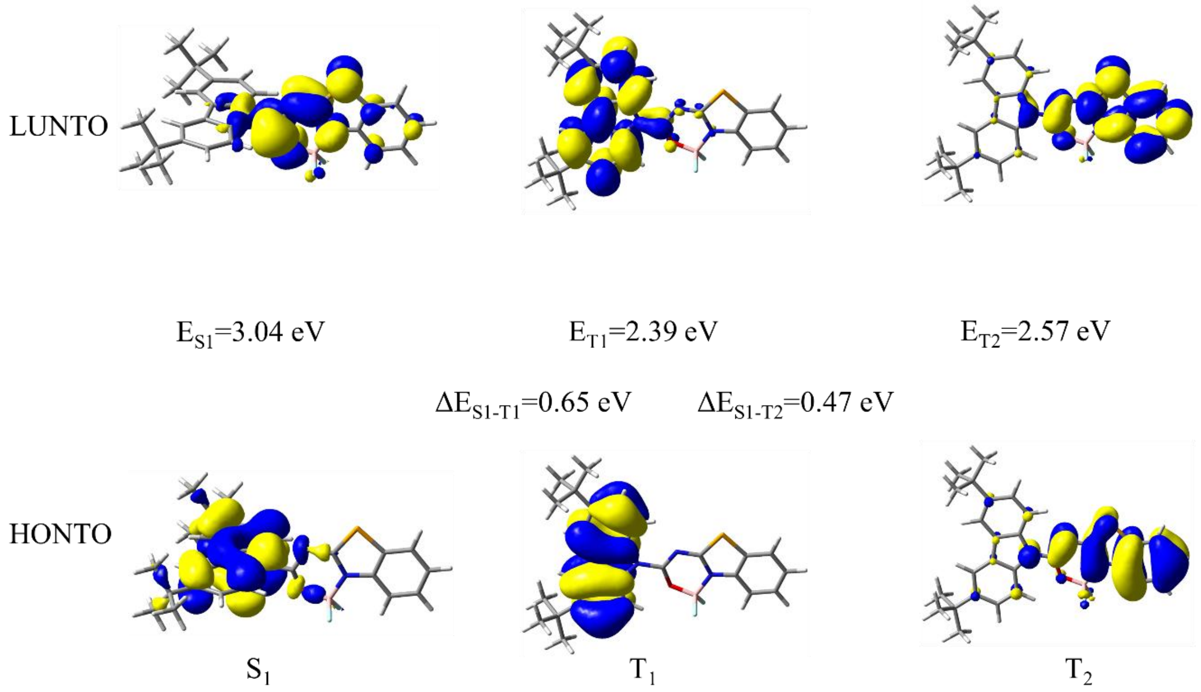

Figure S18. NTO analysis for first singlet and first two triplet excited states of compound **3b**.

Table S9. Calculated properties of **1a,b–3a,b** in S_1,T_1, and T_2.

Dye	Excited state	E, eV	λ, nm	f	Nature
**1a**	S_1	2.90	427	0.0005	CT
	T_1	2.33	531	0.0000	LE
	T_2	2.51	494	0.0000	LE
**2a**	S_1	3.23	384	0.0036	CT
	T_1	2.42	512	0.0000	LE
	T_2	2.51	494	0.0000	LE
**3a**	S_1	3.17	391	0.0005	CT
	T_1	2.38	521	0.0000	LE
	T_2	2.47	502	0.0000	LE
**1b**	S_1	2.73	454	0.0003	CT
	T_1	2.37	523	0.0000	LE
	T_2	2.46	504	0.0000	LE
**2b**	S_1	3.00	413	0.0003	CT
	T_1	2.42	512	0.0000	LE
	T_2	2.46	504	0.0000	LE
**3b**	S_1	3.04	408	0.0002	CT
	T_1	2.39	519	0.0000	LE
	T_2	2.57	482	0.0000	LE

Spin-orbit coupling (SOC) matrix elements for compounds **1a,b**, **2a,b**, and **3a,b** were calculated with PBE0 method using ZORA-DEF2-TZVP basis set and RI-SOMF(1X) function to accelerate the SOC integrals in Orca 6.0. For Te additional basis set SARC-ZORA-TZVP were implemented.

Table S10. Calculated SOCMEs of the triplet states with the singlet excited and ground states for dyes **1a,b**, **2a,b**, and **3a,b** calculated with PBE0 method using DEF2-TZVP basis set in Orca 6.0.

Dye	T	S	X	Y	Z	$\|\langle T_n\|HSO\|S_1\rangle\|^2$
**1a**	1	1	(0.00. 0.03)	(0.00. 0.00)	(0.00. 0.00)	0.03
	2	1	(0.00. 0.02)	(0.00. 0.00)	(0.00. 0.00)	0.02
	3	1	(0.00. 0.00)	(0.00. 0.00)	(0.00. 0.00)	0.00
	4	1	(0.00. -0.01)	(0.00. 0.00)	(0.00. 0.00)	0.00
	5	1	(0.00. 0.14)	(0.00. 0.00)	(0.00. 0.00)	0.14
	6	1	(0.00. 0.02)	(0.00. 0.00)	(0.00. 0.00)	0.02
**2a**	1	1	(0.00. 0.00)	(0.00. 0.04)	(0.00. -0.03)	0.05
	2	1	(0.00. -0.02)	(0.00. -0.07)	(0.00. 0.00)	0.07
	3	1	(0.00. 0.06)	(0.00. 0.09)	(0.00. 0.09)	0.14
	4	1	(0.00. 0.01)	(0.00. 0.01)	(0.00. 0.02)	0.02
	5	1	(0.00. 0.02)	(0.00. 0.03)	(0.00. 0.05)	0.06
	6	1	(0.00. 0.07)	(0.00. 0.19)	(0.00. 0.25)	0.32
**3a**	1	1	(0.00. 0.17)	(0.00. -0.54)	(0.00. 1.12)	1.25
	2	1	(0.00. 0.01)	(0.00. 0.11)	(0.00. 0.12)	0.16
	3	1	(0.00. 0.31)	(0.00. 0.19)	(0.00. 1.97)	2.00
	4	1	(0.00. -0.01)	(0.00. 0.06)	(0.00. -0.06)	0.09
	5	1	(0.00. -0.12)	(0.00. -2.33)	(0.00. -1.62)	2.84
	6	1	(0.00. 0.01)	(0.00. 0.08)	(0.00. -0.35)	0.36

Table S10. (continues).

Dye	T	S	X	Y	Z	$\vert\langle T_n \vert HSO \vert S_1\rangle\vert^2$
**1b**	1	1	(0.00. -0.01)	(0.00. 0.00)	(0.00. 0.00)	0.01
	2	1	(0.00. -0.03)	(0.00. 0.00)	(0.00. 0.00)	0.03
	3	1	(0.00. 0.02)	(0.00. 0.00)	(0.00. 0.00)	0.02
	4	1	(0.00. -0.21)	(0.00. 0.00)	(0.00. 0.00)	0.21
	5	1	(0.00. -0.13)	(0.00. 0.00)	(0.00. 0.00)	0.13
	6	1	(0.00. -0.18)	(0.00. 0.00)	(0.00. -0.01)	0.18
**2b**	1	1	(0.00. -0.06)	(0.00. 0.-11)	(0.00. 0.08)	0.15
	2	1	(0.00. 0.02)	(0.00. -0.03)	(0.00. -0.02)	0.04
	3	1	(0.00. 0.44)	(0.00. 0.32)	(0.00. 0.31)	0.63
	4	1	(0.00. 0.09)	(0.00. 0.01)	(0.00. 0.03)	0.10
	5	1	(0.00. 0.03)	(0.00. 0.03)	(0.00. 0.02)	0.05
	6	1	(0.00. 0.22)	(0.00. 0.69)	(0.00. 0.88)	1.14
**3b**	1	1	(0.00. 0.26)	(0.00. -0.66)	(0.00. 1.47)	1.63
	2	1	(0.00. 0.03)	(0.00. -0.11)	(0.00. -0.10)	0.15
	3	1	(0.00. 0.59)	(0.00. 1.86)	(0.00. 2.90)	2.53
	4	1	(0.00. 0.09)	(0.00. 0.01)	(0.00. 0.16)	0.18
	5	1	(0.00. -0.03)	(0.00. 0.02)	(0.00. 0.30)	0.30
	6	1	(0.00. -0.01)	(0.00. 3.65)	(0.00. 1.47)	3.93

Optimized geometry for compound 1a in its ground (S_0) state obtained using the B3LYP method and 6-31G(d) basis set with the inclusion of toluene solvent effect through the conductor-like polarizable continuum model (CPCM).

Symbolic Z-matrix:

C	6.13678182	-0.32978650	-0.01798534
C	6.25069310	1.06973475	-0.01613766
C	5.12123641	1.89682448	-0.00895772
C	3.90235144	1.24451314	-0.00339311
C	3.77134994	-0.14577135	-0.00432071
C	4.89348504	-0.96878418	-0.01238540
H	7.03885456	-0.93372587	-0.02424298
H	7.23647433	1.52400154	-0.02079467
H	5.19253177	2.97855163	-0.00809945
H	4.80386973	-2.04919426	-0.01496069
C	1.76752235	0.77056485	0.00603286
C	-0.30192423	-0.08216017	0.00255958
O	2.63310163	1.80650256	0.00286816
N	2.39741889	-0.40742377	0.00279167
N	0.46571145	1.00482865	0.01008881
O	0.13344426	-1.30655002	-0.00480101
B	1.58827247	-1.74279906	0.01652365
F	1.82467275	-2.44272223	1.17336387
F	1.84745588	-2.47925130	-1.11217755
C	-2.62325491	-0.99150812	-0.00483715
C	-2.37386543	1.31335807	0.00122288
C	-2.45571048	-2.37850605	-0.00798605
C	-3.91172527	-0.41559409	-0.00604962
C	-1.91133475	2.63080114	0.00498794
C	-3.75532304	1.02666219	-0.00232420
C	-3.60549170	-3.17193950	-0.01203252
H	-1.47837110	-2.83514449	-0.00766484
C	-5.04858120	-1.22701531	-0.01021388
C	-2.86354418	3.65312921	0.00504701
H	-0.85567457	2.85370071	0.00782523
C	-4.69128411	2.06363740	-0.00206704
C	-4.88888611	-2.61056100	-0.01316787
H	-3.49145404	-4.25206743	-0.01447884
H	-6.03967272	-0.78243654	-0.01118067
C	-4.23769301	3.38049520	0.00164042
H	-2.52073253	4.68387648	0.00795714
H	-5.75484571	1.84278623	-0.00476850
H	-5.76174682	-3.25680197	-0.01638893
H	-4.95144681	4.19916315	0.00177240
N	-1.66162796	0.06986120	-0.00010934

Imaginary Frequency = 0

E(RB3LYP) = - 1308.842077 Hartree.

E(TD-HF/TD-DFT) = - 1308.70385664 Hartree.

Optimized geometry for compound 2a in its ground (S_0) state obtained using the B3LYP method and 6-31G(d) basis set with the inclusion of toluene solvent effect through the conductor-like polarizable continuum model (CPCM).

Symbolic Z-matrix:

C	5.92915754	-0.95102933	-0.17773376
C	6.35882391	0.38479120	-0.18947468
C	5.43949973	1.43179313	-0.14080072
C	4.08413499	1.11172205	-0.07285363
C	3.65134863	-0.22726916	-0.05089030
C	4.57654771	-1.27390382	-0.11305319
H	6.66276459	-1.75001181	-0.22496630
H	7.41971042	0.60961305	-0.24175413
H	5.76921584	2.46559126	-0.15676417
H	4.23666926	-2.30258730	-0.12228059
C	1.61615984	0.82077911	0.04191271
C	-0.45207152	-0.09408309	0.02355978
N	2.26128922	-0.35089499	0.02428908
N	0.29585868	1.00074091	0.08628765
O	0.00909604	-1.30933741	-0.03349125
B	1.44845829	-1.67990976	0.22112572
F	1.57774724	-2.12718662	1.51543465
F	1.83191668	-2.61855818	-0.70580106
C	-2.75210869	-1.05316734	-0.06241384
C	-2.55633952	1.25448754	0.00885143
C	-2.55257197	-2.43557417	-0.10492138
C	-4.05376382	-0.50719293	-0.07140840
C	-2.12471178	2.58154143	0.05475063
C	-3.93085346	0.93748113	-0.02721182
C	-3.68336604	-3.25436608	-0.15113226
H	-1.56496759	-2.86957786	-0.10627471
C	-5.17123714	-1.34386323	-0.11851786
C	-3.09931818	3.58231424	0.06264234
H	-1.07439761	2.82643968	0.08430833
C	-4.88996903	1.95311846	-0.01768958
C	-4.97943660	-2.72271139	-0.15748042
H	-3.54424930	-4.33106411	-0.18401511
H	-6.17224141	-0.92207906	-0.12495706
C	-4.46672207	3.27925482	0.02733325
H	-2.78021981	4.62008287	0.09788624
H	-5.94804859	1.70883471	-0.04517495
H	-5.83681220	-3.38835926	-0.19440176
H	-5.19881312	4.08153280	0.03535316
N	-1.81589474	0.02887407	-0.00969892
S	2.69883543	2.20261006	-0.01426964

Imaginary Frequency = 0

E(RB3LYP) = -1631.814934 Hartree.

E(TD-HF/TD-DFT) = -1631.684063 Hartree.

Optimized geometry for compound 3a in its ground (S_0) state obtained using the B3LYP method and 6-31G(d) basis set with the inclusion of toluene solvent effect through the conductor-like polarizable continuum model (CPCM).

Symbolic Z-matrix:

C	5.58660698	-1.59267984	-0.15804467
C	6.17067081	-0.31854673	-0.16821796
C	5.37430647	0.82662750	-0.12794121
C	3.99188245	0.66966761	-0.07066150
C	3.39906115	-0.60520529	-0.05162927
C	4.20435734	-1.75012683	-0.10436994
H	6.21858183	-2.47464285	-0.19745510
H	7.25084413	-0.21695663	-0.21177287
H	5.82347889	1.81465896	-0.14158185
H	3.75005390	-2.73310599	-0.11565631
C	1.40110583	0.57757134	0.03385163
C	-0.71000243	-0.24188886	0.02600305
N	1.99703571	-0.61602335	0.01380510
N	0.09333521	0.81542712	0.07686936
O	-0.30688789	-1.47637303	-0.01781601
B	1.11771982	-1.90812044	0.21124268
F	1.25002613	-2.37052516	1.50006290
F	1.43642952	-2.85825735	-0.72991900
C	-3.05292383	-1.08839873	-0.05651331
C	-2.74587586	1.20773689	0.01049874
C	-2.91966851	-2.47901404	-0.09716493
C	-4.32688270	-0.48013813	-0.06672334
C	-2.25070751	2.51264475	0.05508415
C	-4.13432941	0.95723914	-0.02494683
C	-4.08886028	-3.24270105	-0.14271978
H	-1.95407553	-2.96008838	-0.09756663
C	-5.48354939	-1.26213891	-0.11300438
C	-3.17605624	3.55964583	0.06195502
H	-1.18979096	2.70675717	0.08428868
C	-5.04359584	2.01803861	-0.01669429
C	-5.35806782	-2.64906331	-0.15004158
H	-4.00184005	-4.32488607	-0.17415679
H	-6.46313051	-0.79282702	-0.12044069
C	-4.55673725	3.32264349	0.02676275
H	-2.80731828	4.58084948	0.09614002
H	-6.11220302	1.82504512	-0.04378335
H	-6.24639766	-3.27282680	-0.18622792
H	-5.24926455	4.15929608	0.03373706
N	-2.06552586	-0.05245642	-0.00585918
Se	2.64150430	2.04174247	-0.01638546

Imaginary Frequency = 0

E(RB3LYP) = -1242.936889 Hartree.

E(TD-HF/TD-DFT) = -1242.807357 Hartree.

Optimized geometry for compound 1b in its ground (S_0) state obtained using the B3LYP method and 6-31G(d) basis set with the inclusion of toluene solvent effect through the conductor-like polarizable continuum model (CPCM).

Symbolic Z-matrix:

C	7.68191663	-0.36227504	-0.02101693
C	7.77665809	1.03853928	-0.02216831
C	6.63566066	1.84984711	-0.01556402
C	5.42581097	1.18106958	-0.00742977
C	5.31381558	-0.21095984	-0.00538823
C	6.44733738	-1.01819236	-0.01287744
H	8.59213859	-0.95391102	-0.02693155
H	8.75605908	1.50643501	-0.02871603
H	6.69199433	2.93248734	-0.01694108
H	6.37253886	-2.09973874	-0.01320334
C	3.29714069	0.67788705	0.00486522
C	1.24048697	-0.20507011	0.00475243
O	4.14923509	1.72581617	-0.00084052
N	3.94390115	-0.49169563	0.00339656
N	1.99322008	0.89403371	0.00949517
O	1.69337576	-1.42416613	-0.00053730
B	3.15331069	-1.83824373	0.02036714
F	3.40197635	-2.53316418	1.17833533
F	3.42359877	-2.57380915	-1.10686945
C	-1.06679328	-1.14495105	-0.00000210
C	-0.84962051	1.16011198	0.00277863
C	-0.89553390	-2.52724878	-0.00140827
C	-2.36303047	-0.58986150	-0.00134861
C	-0.42120473	2.48461539	0.00457492
C	-2.22634123	0.85713689	0.00029964
C	-2.04003965	-3.33120627	-0.00387456
H	0.08279307	-2.98280668	-0.00093404
C	-3.48598357	-1.41422781	-0.00387698
C	-1.39429568	3.48940528	0.00373804
H	0.62839468	2.73608144	0.00662258
C	-3.17469305	1.87781946	-0.00039968
C	-3.34594001	-2.80993196	-0.00515386
H	-1.88849907	-4.40421334	-0.00491514
H	-4.47281751	-0.96049180	-0.00486040
C	-2.77493012	3.22238921	0.00129316
H	-1.04460805	4.51518981	0.00512661
H	-4.22918753	1.61752098	-0.00234309
N	-0.11829518	-0.07212334	0.00278080
C	-3.83861172	4.33656403	0.00040055
C	-4.72182201	4.20365597	-1.26326641
C	-4.72606914	4.20167525	1.26087335
C	-3.21451003	5.74513600	0.00255637
H	-4.12034957	4.30376399	-2.17409887

H	-5.23343179	3.23621006	-1.30401229
H	-5.48913179	4.98725983	-1.27461099
H	-4.12766165	4.30028837	2.17388360
H	-5.49337569	4.98529962	1.27089071
H	-5.23786367	3.23419537	1.29834832
H	-4.01019880	6.49844458	0.00182266
H	-2.59747292	5.91738303	0.89173701
H	-2.59450986	5.91879398	-0.88428587
C	-4.59987435	-3.70443707	-0.00793743
C	-5.44589325	-3.40782244	1.25335815
C	-5.44230601	-3.40504775	-1.27098074
C	-4.25139837	-5.20514058	-0.00909544
H	-4.87652729	-3.61937888	2.16573891
H	-5.76668764	-2.36145039	1.29330755
H	-6.34680719	-4.03327284	1.26199942
H	-4.87031401	-3.61450842	-2.18220311
H	-6.34313634	-4.03054846	-1.28360332
H	-5.76308061	-2.35861543	-1.30949718
H	-5.17446077	-5.79551372	-0.01101959
H	-3.67517626	-5.49028545	-0.89659439
H	-3.67763476	-5.49222266	0.87936910

Imaginary Frequency = 0

E(RB3LYP) = -1623.351557 Hartree.

E(TD-HF/TD-DFT) = -1623.216409 Hartree.

Optimized geometry for compound 2b in its ground (S_0) state obtained using the B3LYP method and 6-31G(d) basis set with the inclusion of toluene solvent effect through the conductor-like polarizable continuum model (CPCM).

Symbolic Z-matrix:

C	7.46931830	-0.98913552	-0.18927813
C	7.88736605	0.34981418	-0.22481481
C	6.95900262	1.38941487	-0.18685855
C	5.60697459	1.05896485	-0.10529006
C	5.18574898	-0.28315902	-0.05943991
C	6.11983492	-1.32242350	-0.11097122
H	8.20942205	-1.78257517	-0.22848596
H	8.94590660	0.58300182	-0.28735245
H	7.27954285	2.42568095	-0.22128788
H	5.78882185	-2.35399388	-0.10183548
C	3.14147945	0.74855537	0.02976983
C	1.08207075	-0.18580772	0.04098945
N	3.79747275	-0.41789248	0.02651833
N	1.82113045	0.91787390	0.07991536
O	1.55400125	-1.39858791	0.00090510
B	2.99775252	-1.75057374	0.25071916
F	3.14171260	-2.17616425	1.55133194

F	3.38453496	-2.70114437	-0.66335570
C	-1.20897077	-1.16404776	-0.01050203
C	-1.03261778	1.14313936	0.01703543
C	-1.01348982	-2.54328107	-0.02881023
C	-2.51508097	-0.63217701	-0.02021381
C	-0.62799914	2.47493409	0.03516884
C	-2.40397515	0.81675411	-0.00336566
C	-2.14348168	-3.36704913	-0.05161575
H	-0.02726603	-2.98150024	-0.02911049
C	-3.62318375	-1.47610969	-0.04364977
C	-1.61787637	3.46300861	0.03162003
H	0.41720058	2.74338834	0.05262756
C	-3.36979871	1.82098127	-0.00557020
C	-3.45847186	-2.86898779	-0.05893907
H	-1.97293279	-4.43713686	-0.06500627
H	-4.61786202	-1.03984459	-0.05021065
C	-2.99364203	3.17221596	0.01195319
H	-1.28606496	4.49464778	0.04567211
H	-4.41953868	1.54254202	-0.02120257
N	-0.27996281	-0.07509138	0.01566659
S	4.21262271	2.13899666	-0.05514173
C	-4.07646710	4.26778658	0.00948255
C	-4.94403936	4.13088781	-1.26456208
C	-4.97456486	4.10641789	1.25927418
C	-3.47703151	5.68688517	0.03053581
H	-4.33495434	4.24960950	-2.16807114
H	-5.43814128	3.15507110	-1.31920649
H	-5.72480702	4.90106726	-1.27716326
H	-4.38751821	4.20728306	2.17937648
H	-5.75549369	4.87648508	1.26799625
H	-5.46974094	3.12990760	1.28297890
H	-4.28562441	6.42631264	0.02799524
H	-2.87236217	5.86184212	0.92765169
H	-2.85095563	5.87921067	-0.84816152
C	-4.69632081	-3.78530990	-0.08354786
C	-5.55783165	-3.51894828	1.17401170
C	-5.53349891	-3.48543143	-1.34996705
C	-4.32136650	-5.27952972	-0.09984712
H	-4.99245606	-3.73189591	2.08853516
H	-5.89685069	-2.47877232	1.22395027
H	-6.44789095	-4.15979879	1.16744374
H	-4.95031064	-3.67345432	-2.25876688
H	-6.42286224	-4.12664646	-1.37787360
H	-5.87265544	-2.44449402	-1.37852261
H	-5.23376922	-5.88602171	-0.11657190
H	-3.73297864	-5.54367023	-0.98587204
H	-3.74982611	-5.56724809	0.78983062

Imaginary Frequency = 0

E(RB3LYP) = -1946.324390 Hartree.
E(TD-HF/TD-DFT) = -1946.196793 Hartree.

Optimized geometry for compound 3b in its ground (S_0) state obtained using the B3LYP method and 6-31G(d) basis set with the inclusion of toluene solvent effect through the conductor-like polarizable continuum model (CPCM).

Symbolic Z-matrix:

C	7.08076916	-1.68140048	-0.15682218
C	7.65811948	-0.40467720	-0.18878479
C	6.85563917	0.73668791	-0.16102930
C	5.47444030	0.57347828	-0.09407790
C	4.88821906	-0.70399000	-0.05317368
C	5.69955844	-1.84513948	-0.09345066
H	7.71705538	-2.56066809	-0.18663924
H	8.73750179	-0.29801551	-0.23956403
H	7.29935127	1.72683158	-0.19147414
H	5.25038480	-2.83051647	-0.08791890
C	2.88379461	0.46940577	0.02613200
C	0.77786159	-0.36263093	0.04365025
N	3.48679244	-0.72148555	0.02000396
N	1.57625884	0.70104900	0.07351935
O	1.18786567	-1.59625867	0.01460988
B	2.61559838	-2.01538257	0.24032984
F	2.75976567	-2.46035149	1.53460542
F	2.93542573	-2.97693226	-0.68959350
C	-1.55963092	-1.22098064	-0.00690262
C	-1.26483452	1.07459393	0.02007378
C	-1.43463163	-2.60857750	-0.02574433
C	-2.83686155	-0.62263388	-0.01700550
C	-0.79236253	2.38406487	0.03883369
C	-2.65139909	0.81891575	-0.00027002
C	-2.60563347	-3.37351158	-0.04950680
H	-0.47215759	-3.09682671	-0.02575171
C	-3.98697593	-1.40865873	-0.04112954
C	-1.73038448	3.42200131	0.03562775
H	0.26525674	2.59867039	0.05642605
C	-3.56464249	1.87142058	-0.00227667
C	-3.89359300	-2.80850167	-0.05700932
H	-2.49030446	-4.45090564	-0.06338056
H	-4.95795672	-0.92206921	-0.04792243
C	-3.11947563	3.20189361	0.01559871
H	-1.34633001	4.43532254	0.05009368
H	-4.62727017	1.64730035	-0.01783696
N	-0.57584952	-0.18097050	0.01909663
Se	4.11724831	1.93950146	-0.05248750
C	-4.14486806	4.35156279	0.01328480
C	-5.01909452	4.25870123	-1.26043001

C	-5.04930272	4.23658661	1.26389043
C	-3.47350051	5.73824320	0.03345215
H	-4.40517694	4.34604063	-2.16432961
H	-5.56226261	3.30923133	-1.31433917
H	-5.75960153	5.06773771	-1.27271659
H	-4.45718239	4.30778808	2.18359913
H	-5.79000012	5.04549679	1.27271513
H	-5.59344549	3.28645232	1.28806575
H	-4.24327215	6.51811547	0.03070927
H	-2.86046452	5.88242812	0.93044541
H	-2.83860802	5.89768774	-0.84558765
C	-5.17651842	-3.66066717	-0.08251738
C	-6.02411216	-3.35117900	1.17492710
C	-5.99673851	-3.31809095	-1.34949509
C	-4.87799680	-5.17214190	-0.09912470
H	-5.47064472	-3.59281714	2.08968475
H	-6.30992804	-2.29509161	1.22483127
H	-6.94551749	-3.94615958	1.16764951
H	-5.42340771	-3.53555018	-2.25808043
H	-6.91773436	-3.91305170	-1.37790353
H	-6.28212724	-2.26109282	-1.37781364
H	-5.82016492	-5.73140543	-0.11654294
H	-4.30326898	-5.46551006	-0.98499739
H	-4.32228771	-5.48873560	0.79085774

Imaginary Frequency = 0

E(RB3LYP) = -1557.441021 Hartree.

E(TD-HF/TD-DFT) = -1557.314580 Hartree.

4. Photophysical Properties in Solutions

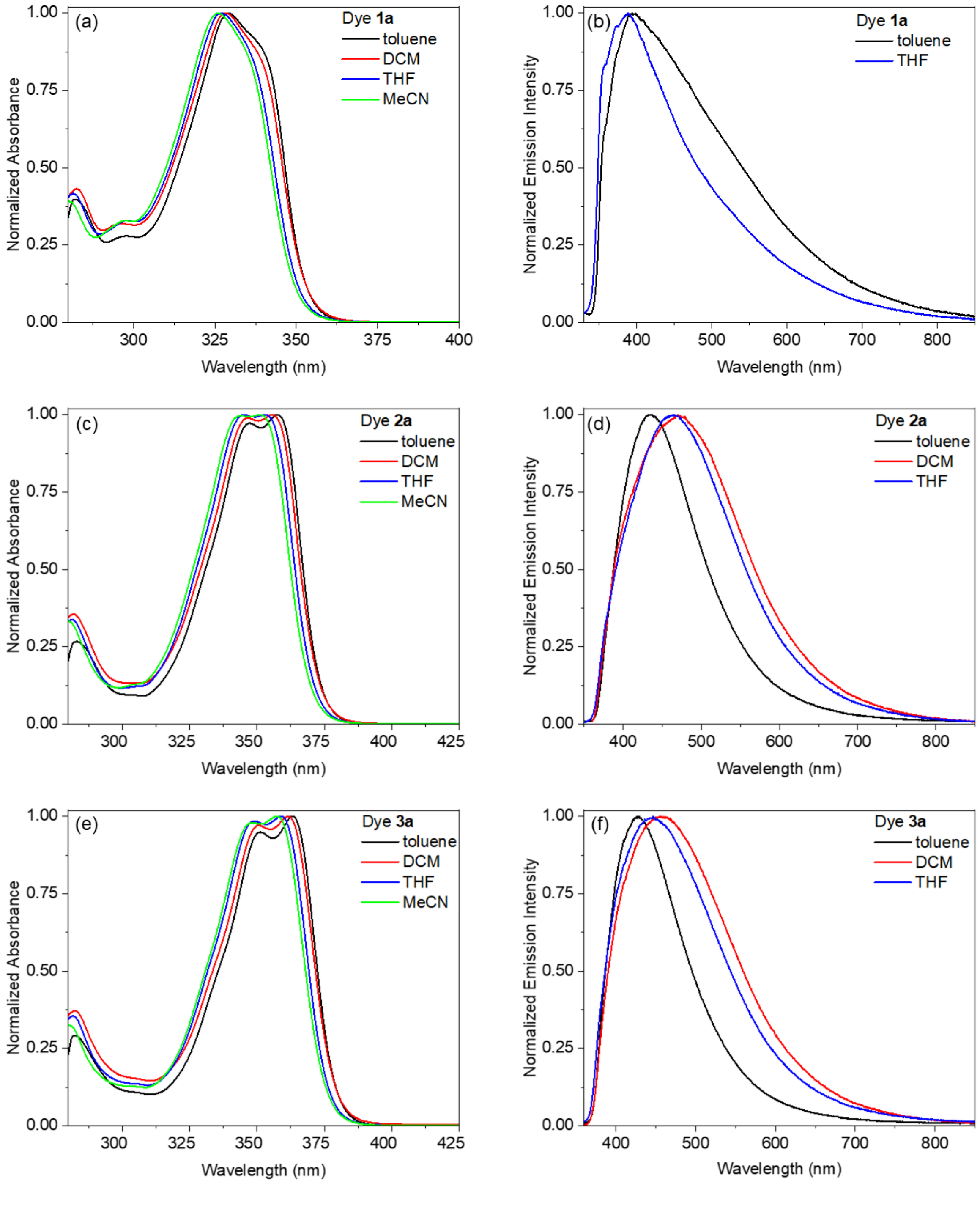

Figure S19. Absorption (a, c, e) and emission (b, d, f) spectra of solutions of compounds **1a** (a, b), **2a** (c, d), and **3a** (e, f) in different solvents.

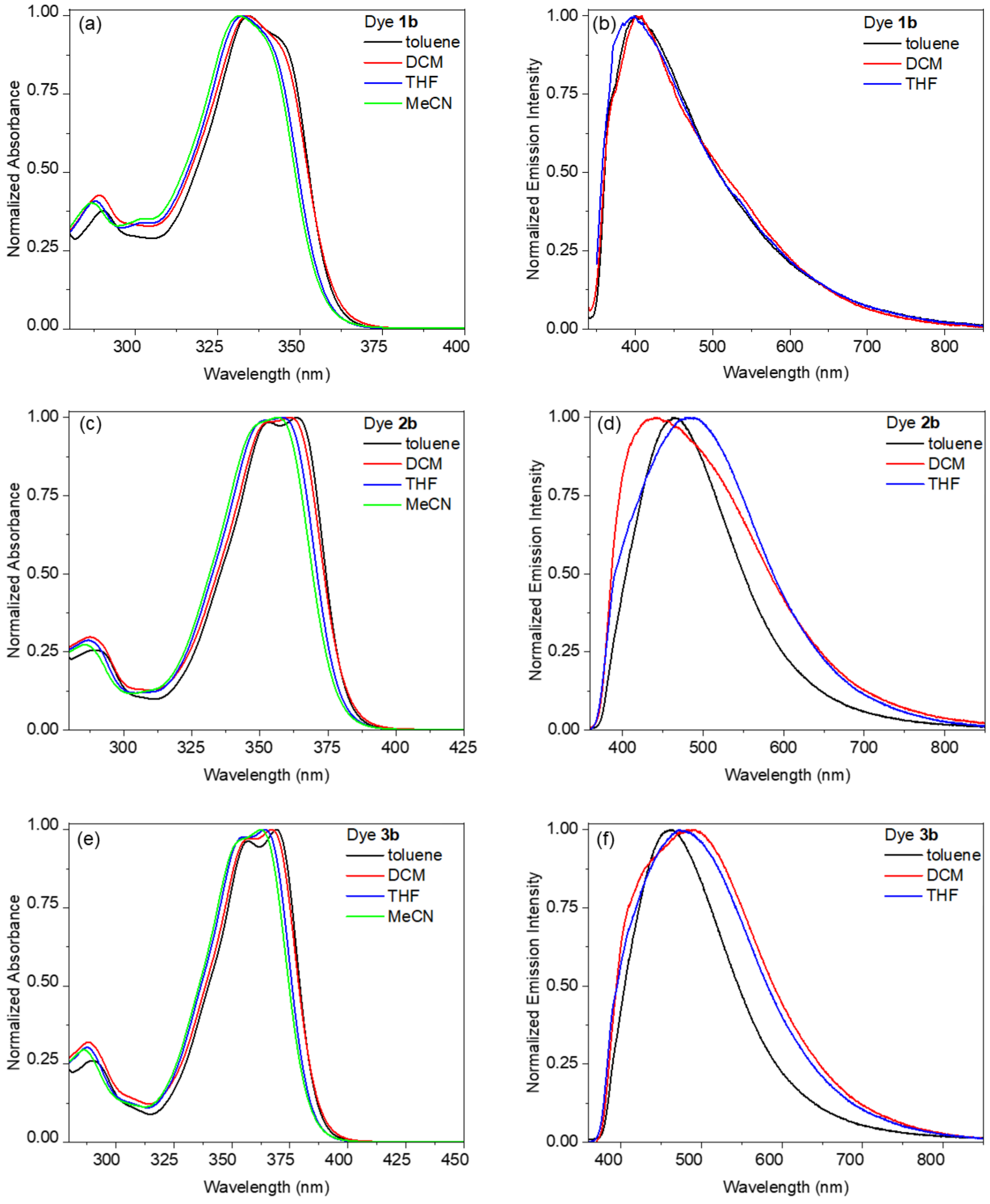

Figure S20. Absorption (a, c, e) and emission (b, d, f) spectra of solutions of compounds **1b** (a, b), **2b** (c, d), and **3b** (e, f) in different solvents.

Table S11. Photophysical properties of boron difluoride complexes **1a,b–3a,b** in different solvents.

Dye	Solvent	λ_{abs} (nm)[a]	ε ($M^{-1} \times cm^{-1}$)[b]	λ_{em} (nm)[c]	PLQY (%)[d]
**1a**	Toluene	329	45000	398	< 1
	CH_2Cl_2	328	47300	n.d.[e]	n.d.
	THF	327	51200	389	< 0.1
	MeCN	326	51000	n.d.	n.d.
**2a**	Toluene	347/357	43300/44700	436	12
	CH_2Cl_2	346/356	48700/49300	470	6
	THF	345/353	49700/49700	463	1
	MeCN	344/351	43300/43700	n.d.	n.d.
**3a**	Toluene	351/363	45000/60000	428	4
	CH_2Cl_2	350/361	50000/51700	457	< 1
	THF	349/359	51000/51700	446	< 1
	MeCN	347/357	41000/41700	n.d.	n.d.
**1b**	Toluene	334	41000	399	< 0.1
	CH_2Cl_2	334	49700	405	1
	THF	332	54700	399	< 0.1
	MeCN	331	18300	n.d.	n.d.
**2b**	Toluene	354/364	48300/49000	464	< 1
	CH_2Cl_2	353/361	57000/58300	444	< 1
	THF	351/358	52000/52300	481	< 0.1
	MeCN	353/356	38300/39300	n.d.	n.d.
**3b**	Toluene	357/369	48000/50000	462	1
	CH_2Cl_2	357/367	50000/51700	487	2
	THF	355/364	51300/52700	473	< 1
	MeCN	354/362	50000/52000	n.d.	n.d.

[a] Absorption maximum. [b] Molar absorption coefficient. [c] Emission maximum. [d] Photoluminescence quantum yield. [e] n.d. – not detectable due to too weak emission.

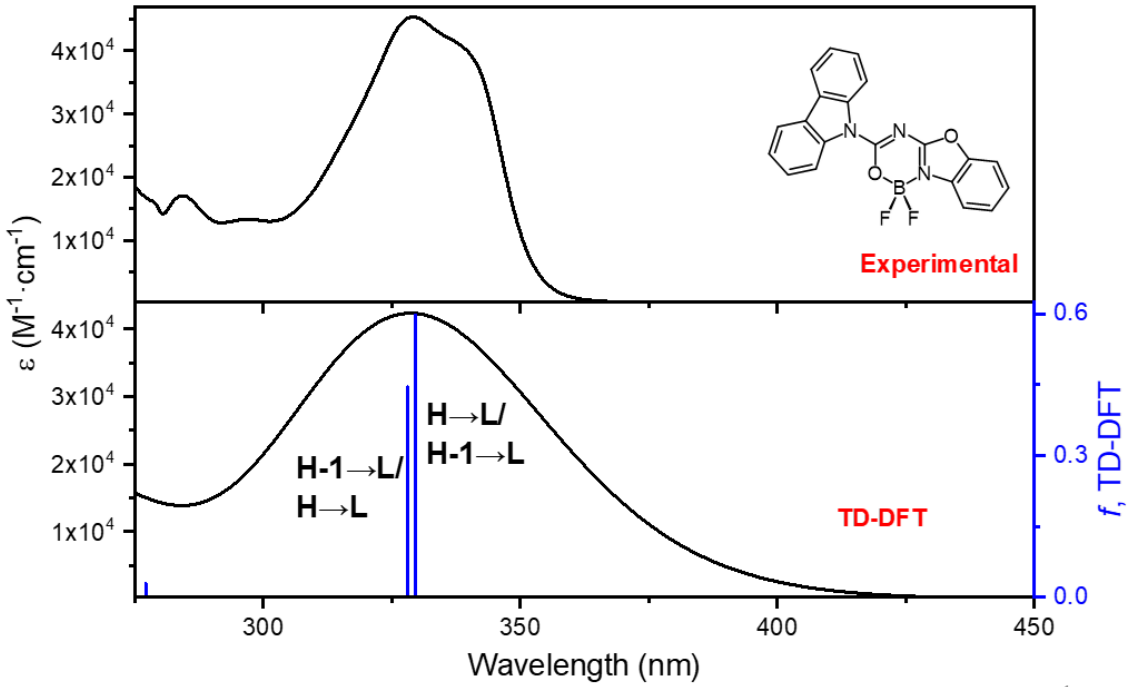

1

Figure S21. Experimental (top) and TD-DFT-predicted (bottom) absorption spectra of dye **1a** in toluene.

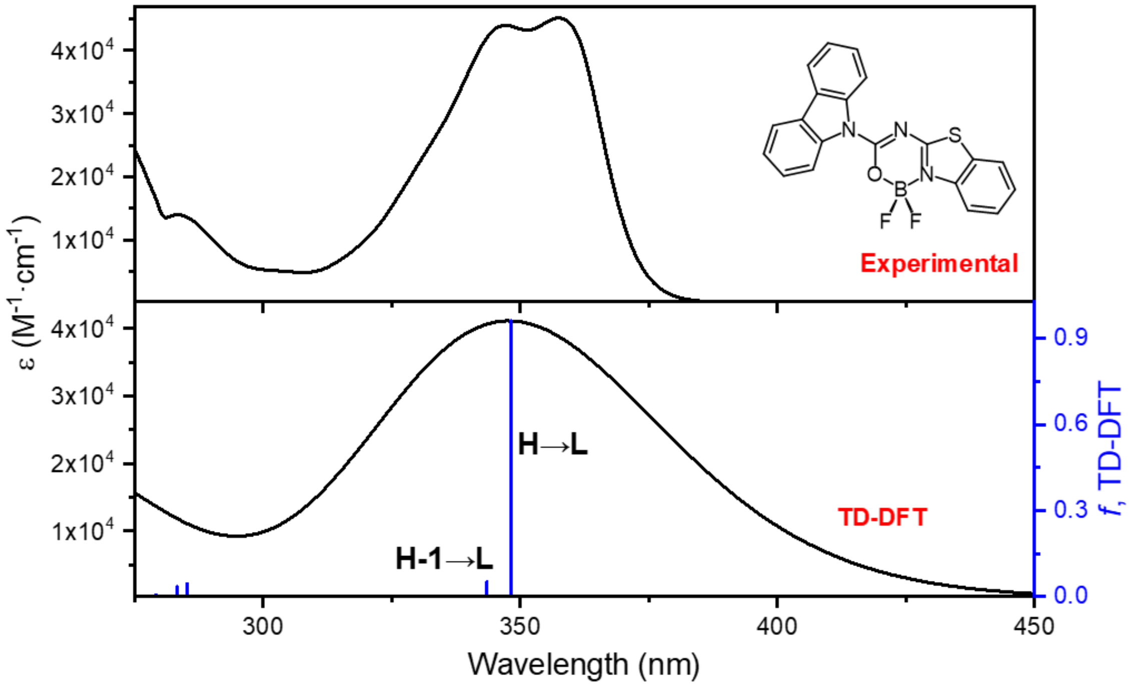

Figure S22. Experimental (top) and TD-DFT-predicted (bottom) absorption spectra of dye **2a** in toluene.

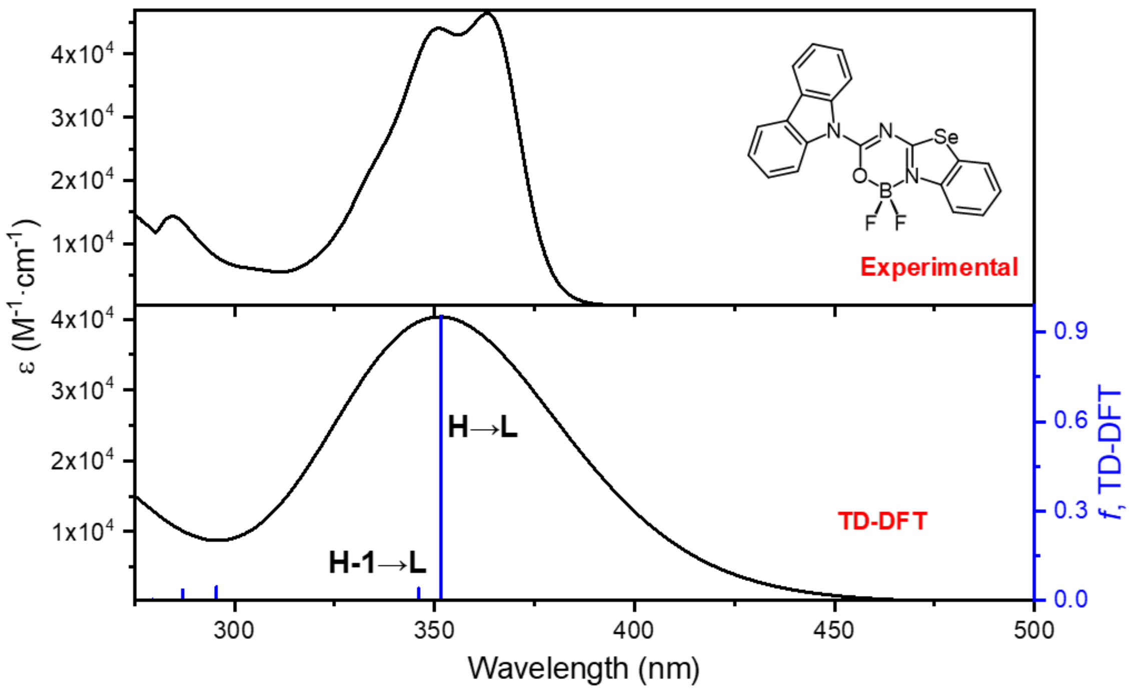

Figure S23. Experimental (top) and TD-DFT-predicted (bottom) absorption spectra of dye **3a** in toluene.

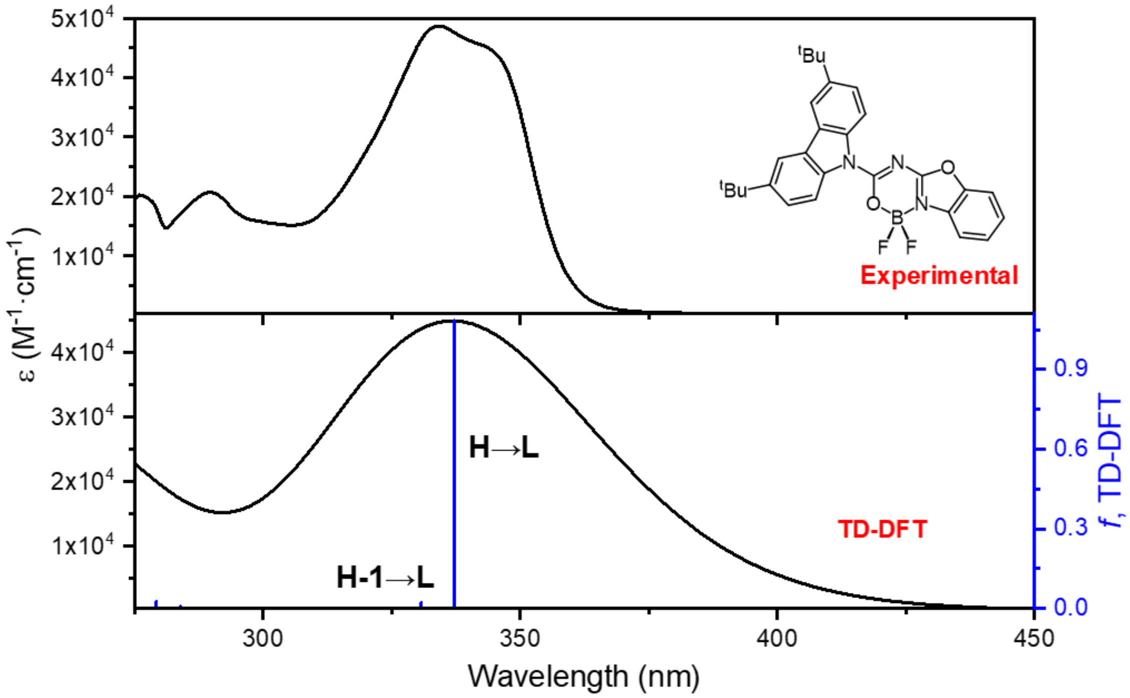

Figure S24. Experimental (top) and TD-DFT-predicted (bottom) absorption spectra of dye **1b** in toluene.

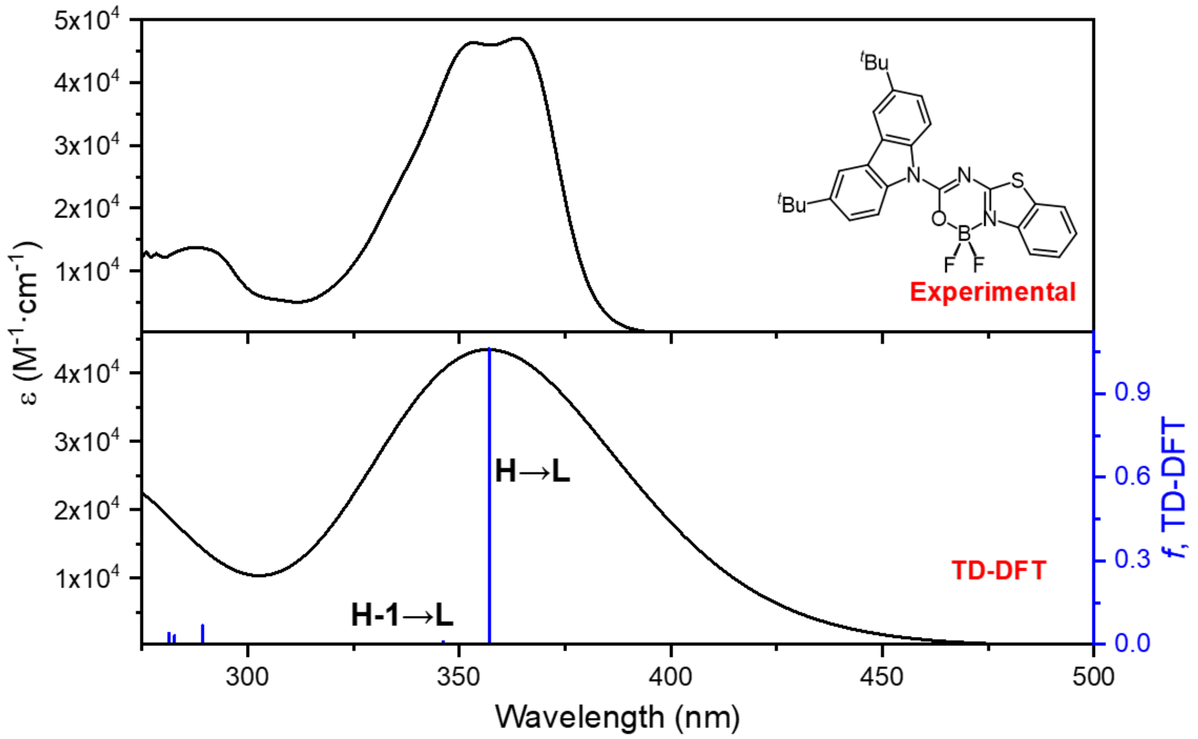

Figure S25. Experimental (top) and TD-DFT-predicted (bottom) absorption spectra of dye **2b** in toluene.

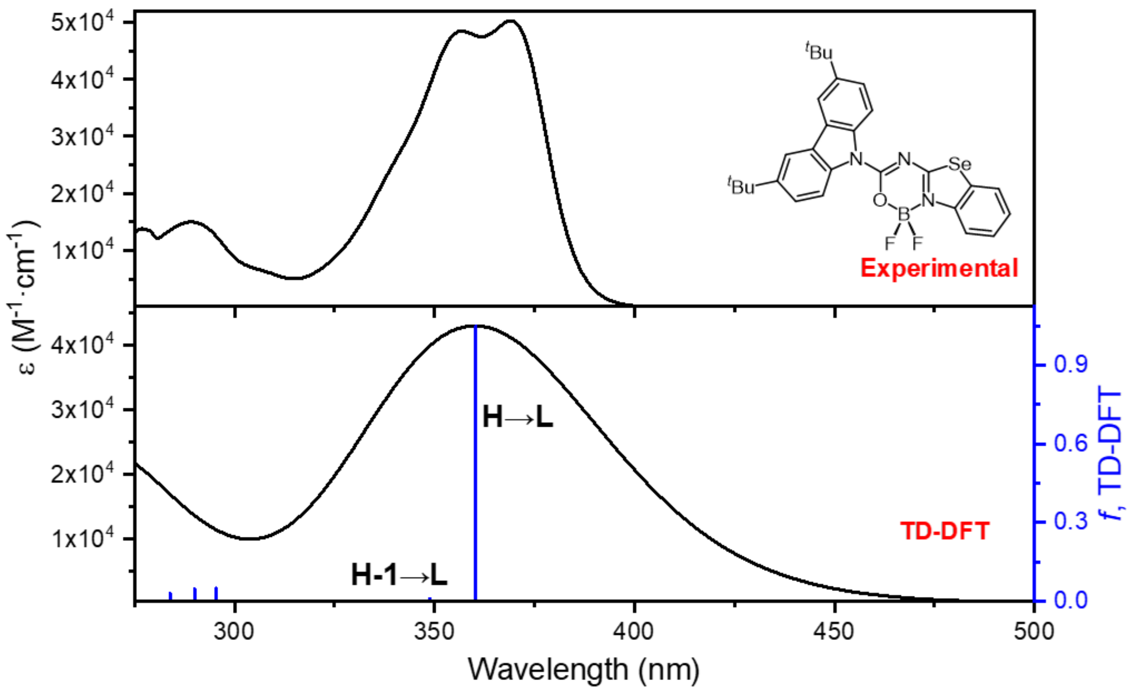

Figure S26. Experimental (top) and TD-DFT-predicted (bottom) absorption spectra of dye **3b** in toluene.

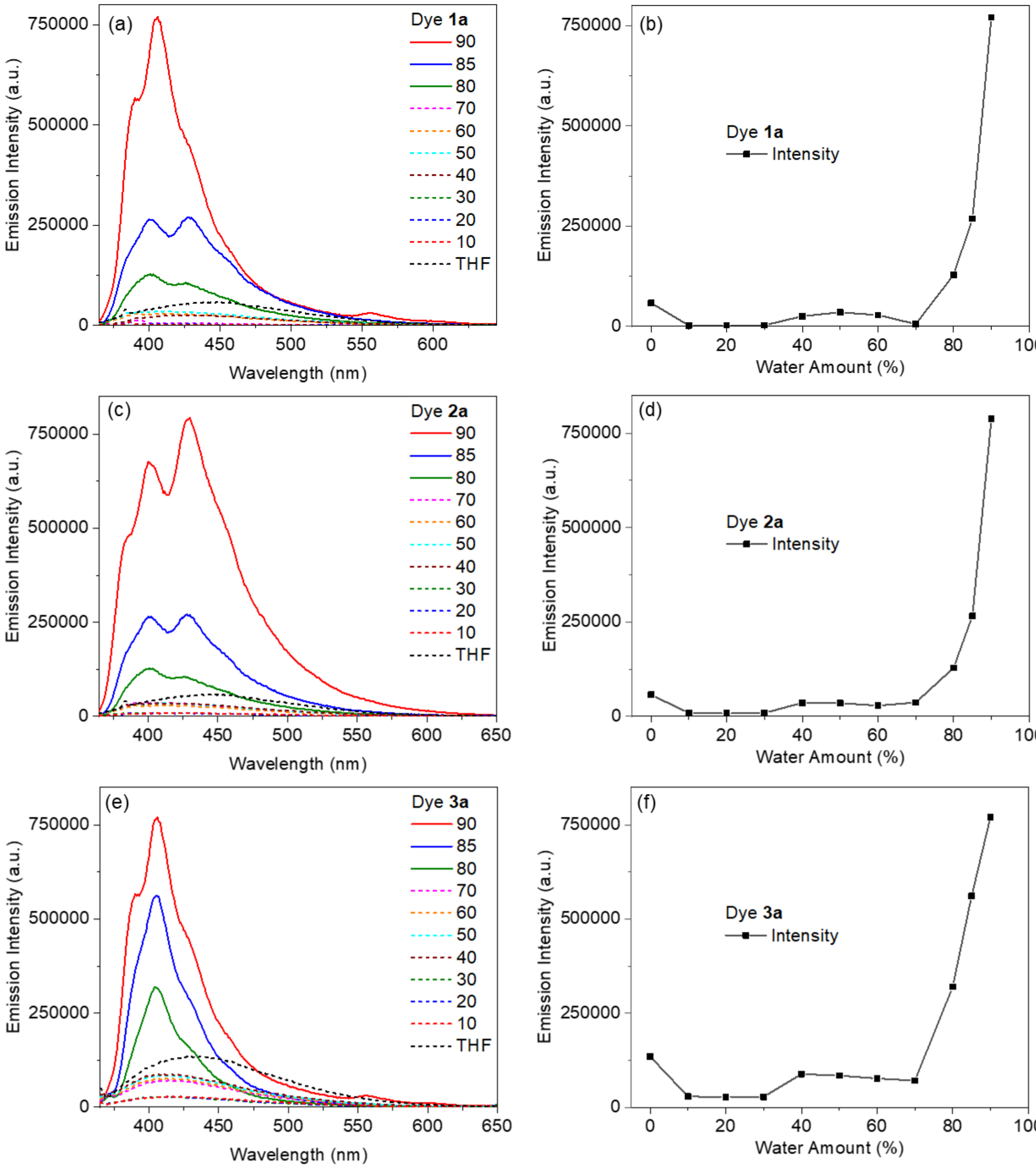

Figure S27. Photoluminescence spectra of the dispersions of boron complexes **1a** (a), **2a** (c), and **3a** (e) in THF/water mixtures of varying water contents. The dye concentration used was 5.0×10^{-6} M, λ_{ex} = 330 nm, 345 nm, and 347 nm for dyes **1a**, **2a**, and **3a**, respectively. Plots of emission intensity of dyes **1a** (b), **2a** (d), and **3a** (f) versus f_w.

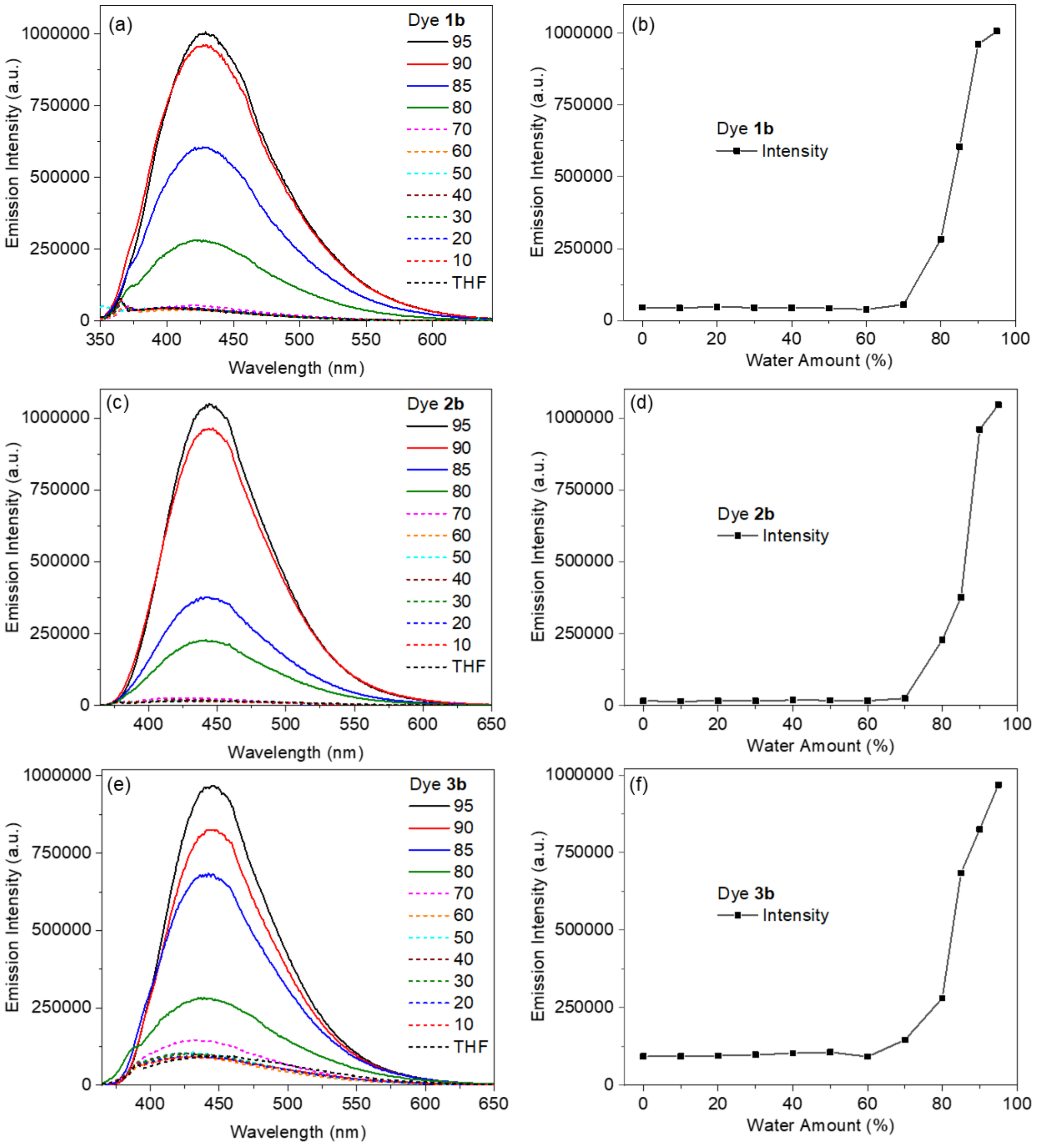

Figure S28. Photoluminescence spectra of the dispersions of boron complexes **1b** (a), **2b** (c), and **3b** (e) in THF/water mixtures of varying water contents. The dye concentration used was 5.0×10^{-6} M, λ_{ex} = 330 nm, 340 nm, and 350 nm for dyes **1b**, **2b**, and **3b**, respectively. Plots of emission intensity of dyes **1b** (b), **2b** (d), and **3b** (f) versus f_w.

5. Photophysical Properties in Crystalline State

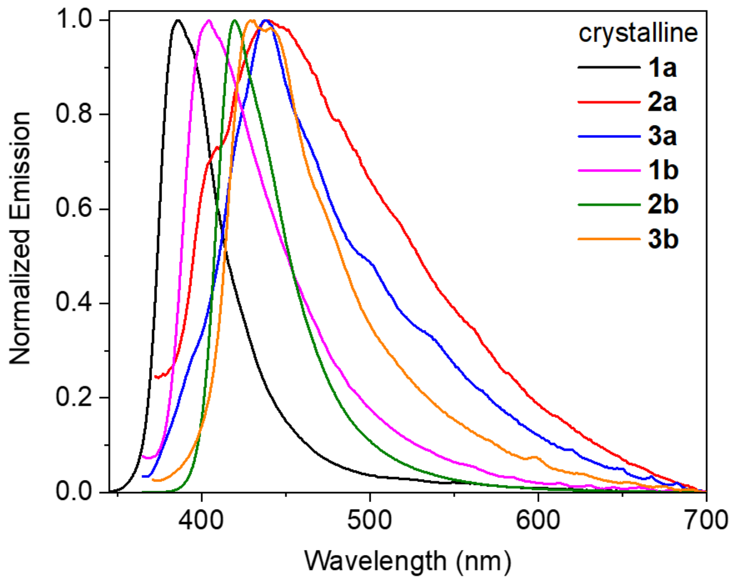

Figure S29. Photoluminescence spectra of the crystalline samples of dyes **1a,b**, **2a,b**, and **3a,b**.

Table S12. Photoluminescence properties of the crystalline samples of compounds **1a,b**, **2a,b**, and **3a,b**.

Dye	λ_{PL}, nm [a]	PLQY, % [b]	$\tau_{p\ av}$, ns [c]	$\tau_{d\ av}$, μs [d]
**1a**	386	42	1.54	5.95
**2a**	440	2	0.40	68.83
**3a**	438	1	0.30	1.99
**1b**	404	85	2.77	1.02
**2b**	420	57	1.85	4.83
**3b**	431	1	0.21	4.33

[a] Wavelength of photoluminescence maximum. [b] Photoluminescence quantum yield measured in ambient atmosphere. [c] Average excited-state lifetime in nanosecond range. [d] Average excited-state lifetime in microsecond range.

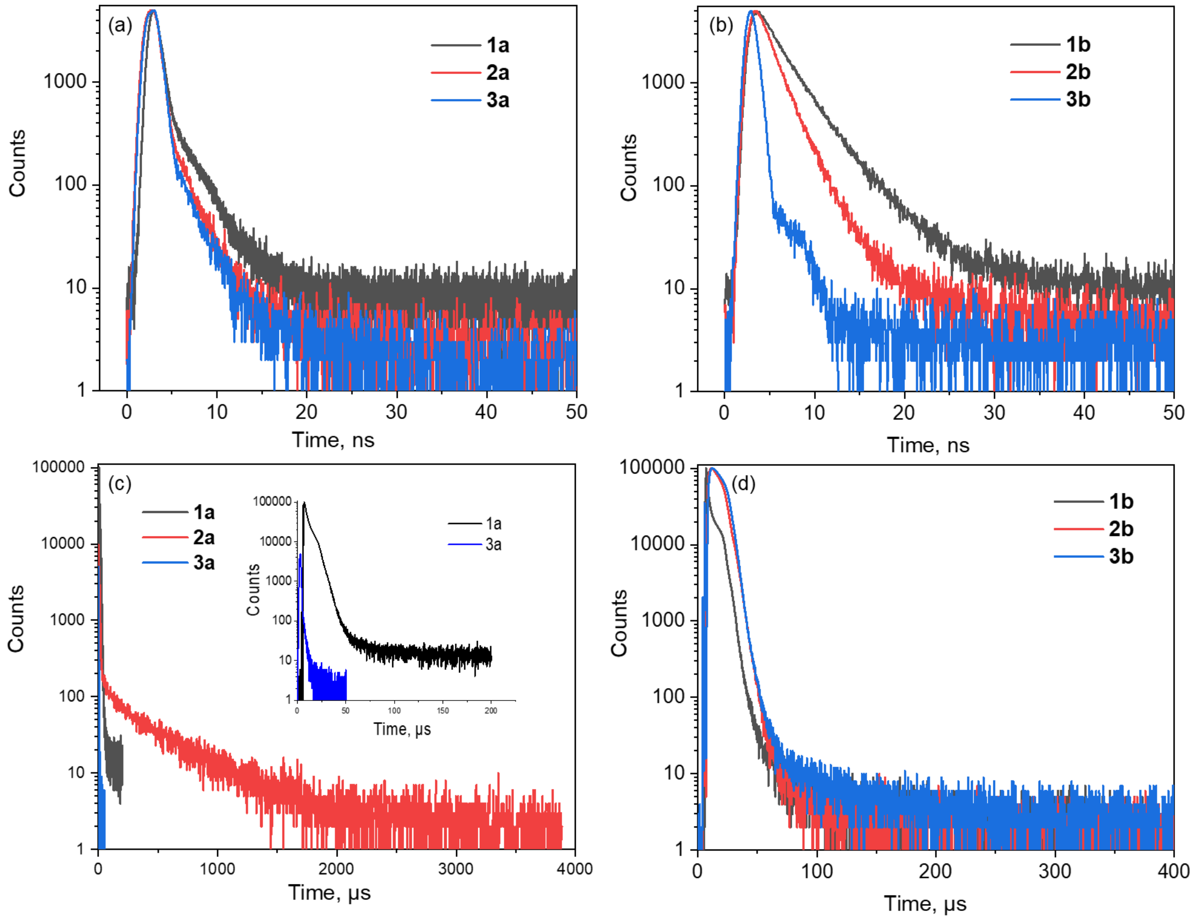

Figure S30. Photoluminescence decay curves of compounds **1a,b–3a,b** in crystalline state recorded at nanosecond (a, b) and microsecond (c, d) ranges.

6. Photophysical Properties of Dye-Doped PMMA Films

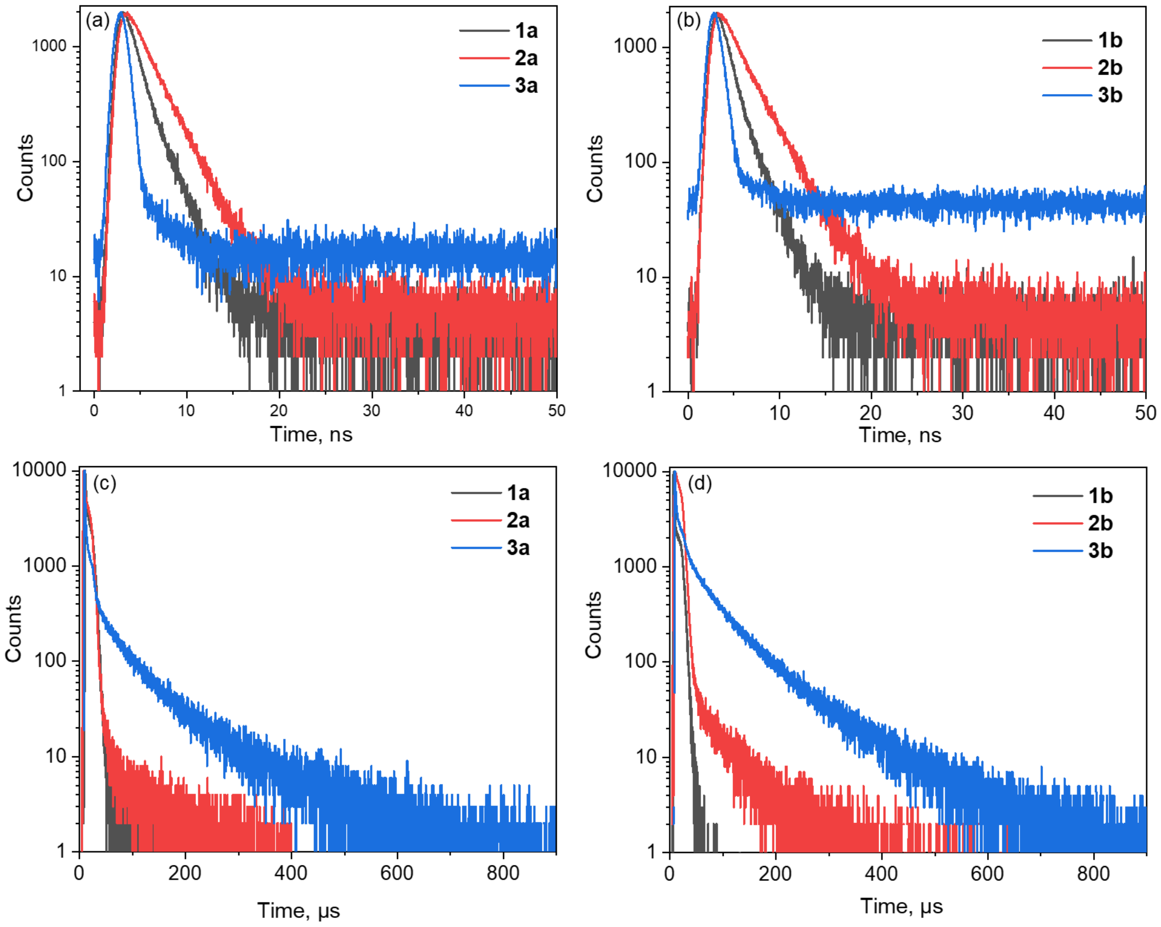

Figure S31. Photoluminescence decay curves of compounds **1a,b**, **2a,b**, and **3a,b** in PMMA films recorded at nanosecond (a, b) and microsecond (c, d) ranges.

7. ASE Measurements

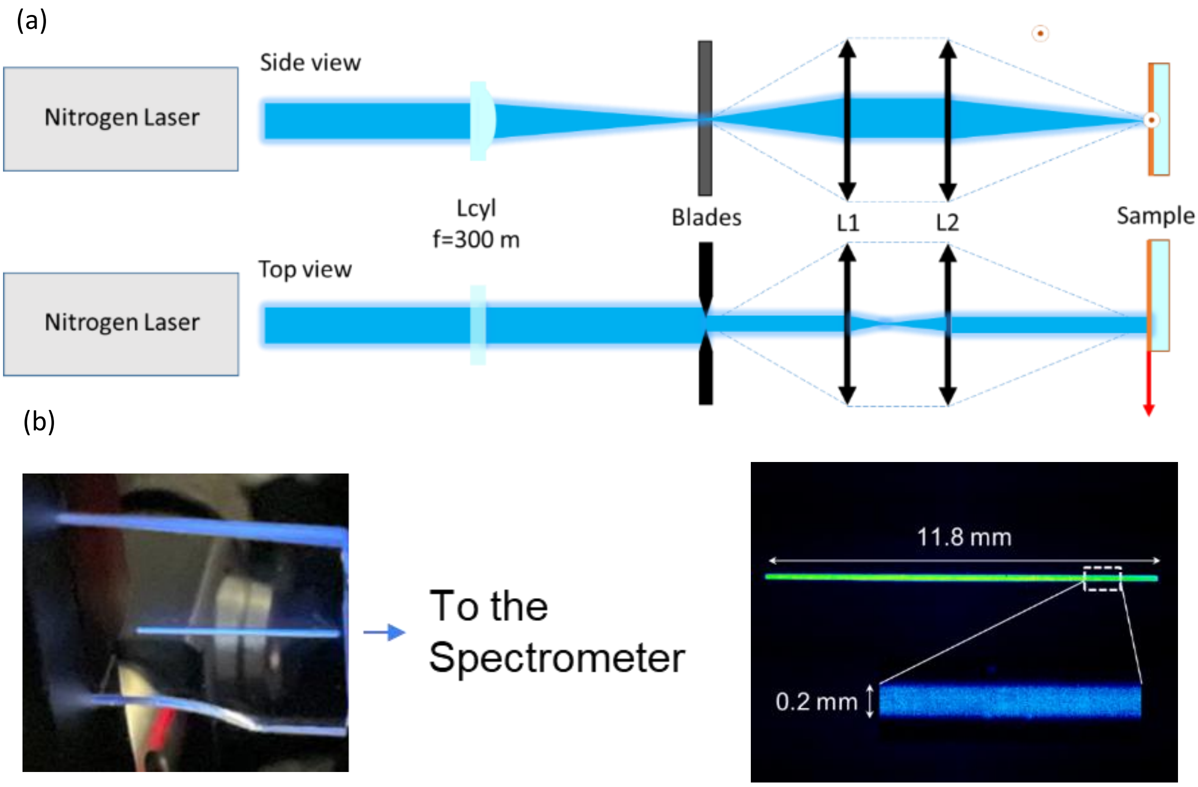

Figure S32. (a) Experimental setup for the ASE measurement (side and top view). (b) Photography (left) and CCD image (false colors, right) of the fluorescence of compound **1a** under stripe-shaped Nitrogen laser excitation.

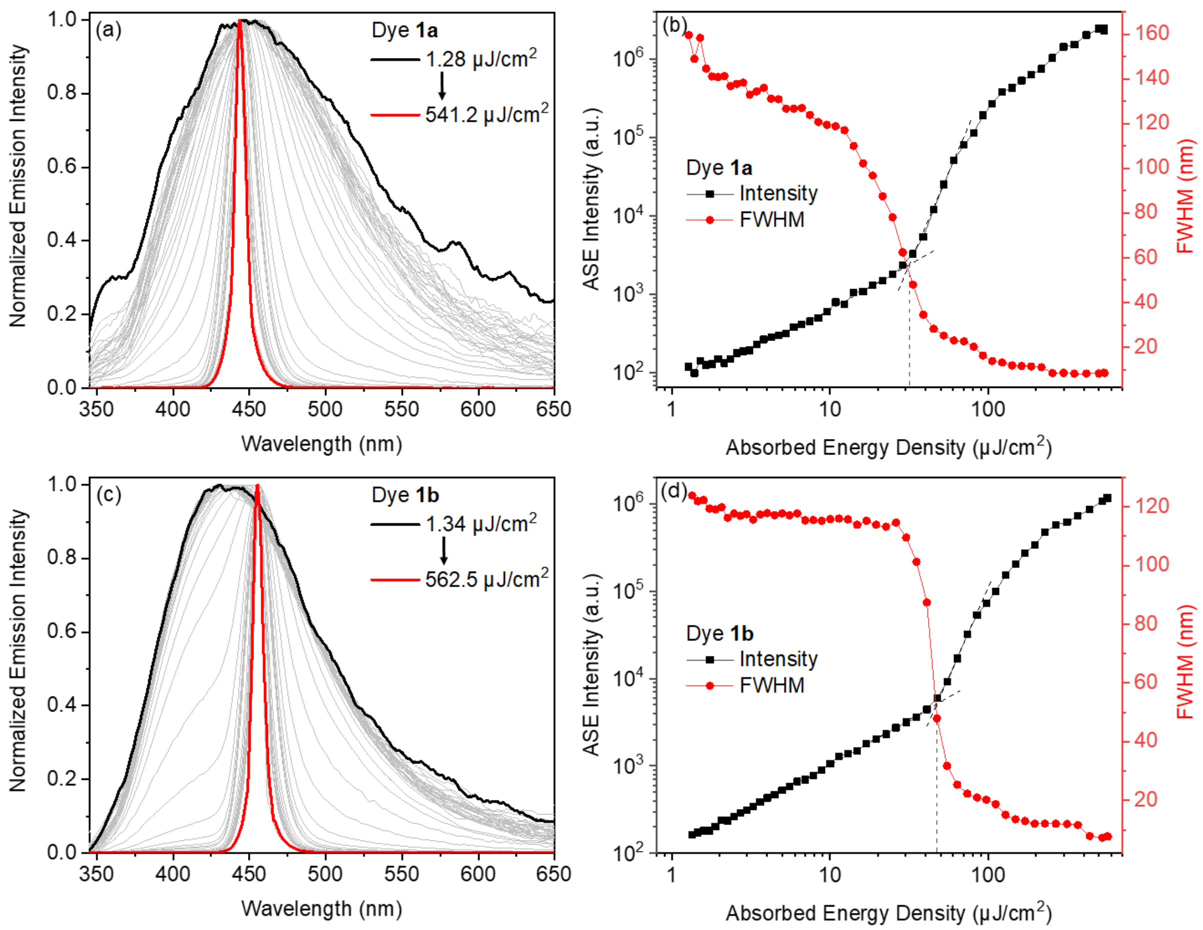

Figure S33. Normalized spectra of amplified spontaneous emission of dyes **1a** (a) and **1b** (c) in PMMA. FWHM values as a function of absorbed energy density for dyes **1a** (b) and **1b** (d).

8. Copies of NMR Spectra

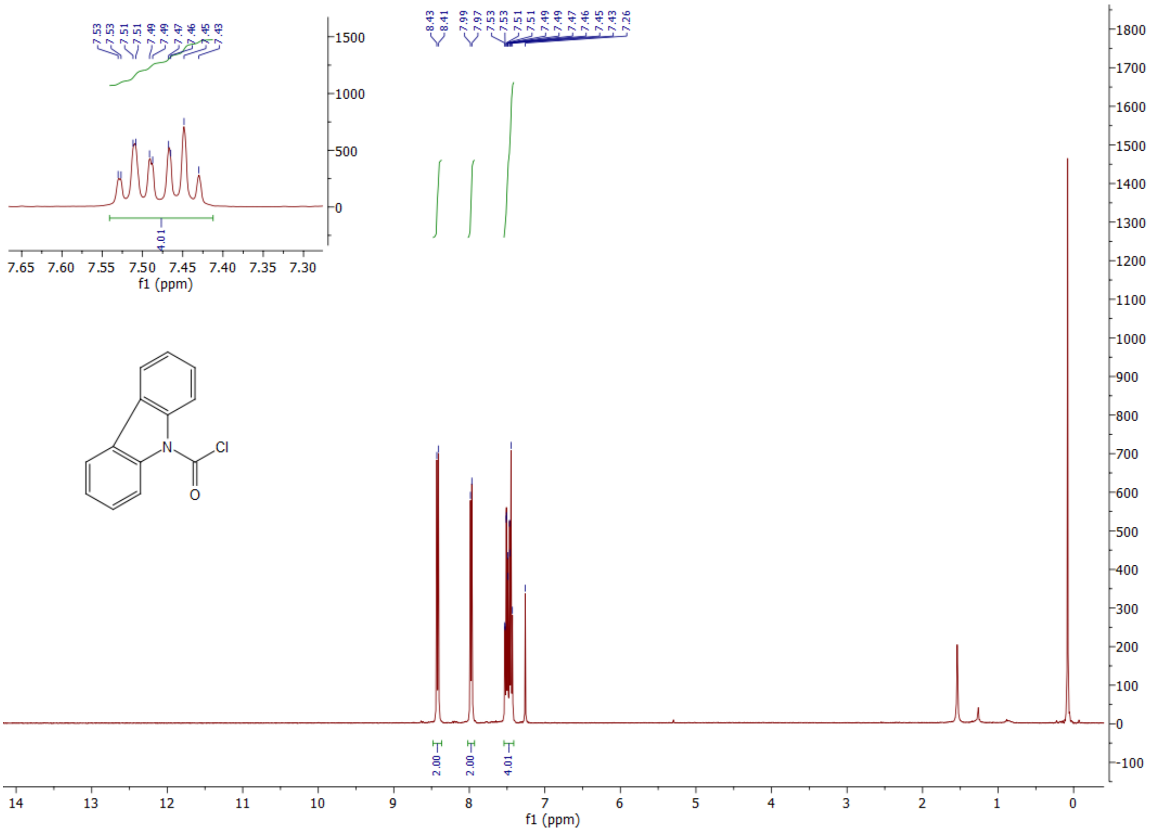

Figure S34. ^{1}H NMR (400 MHz, $CDCl_3$) spectrum of compound **5a**.

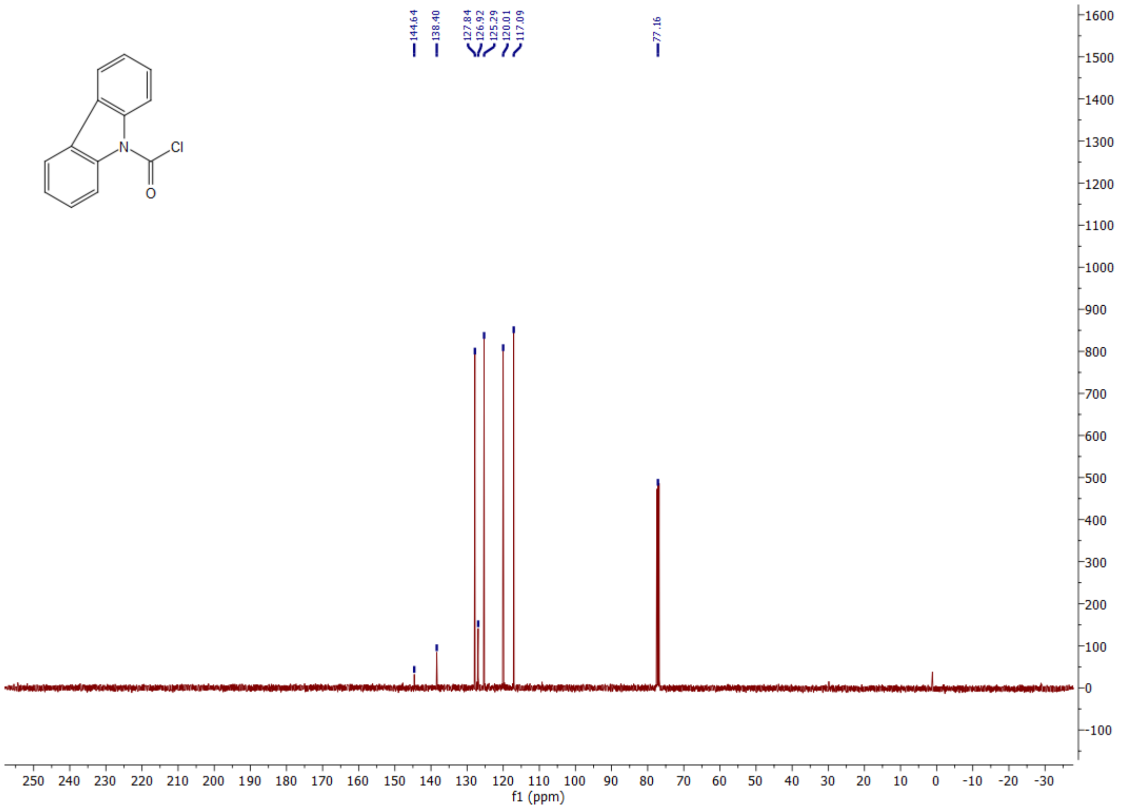

Figure S35. ^{13}C{H} NMR (100 MHz, $CDCl_3$) spectrum of compound **5a**.

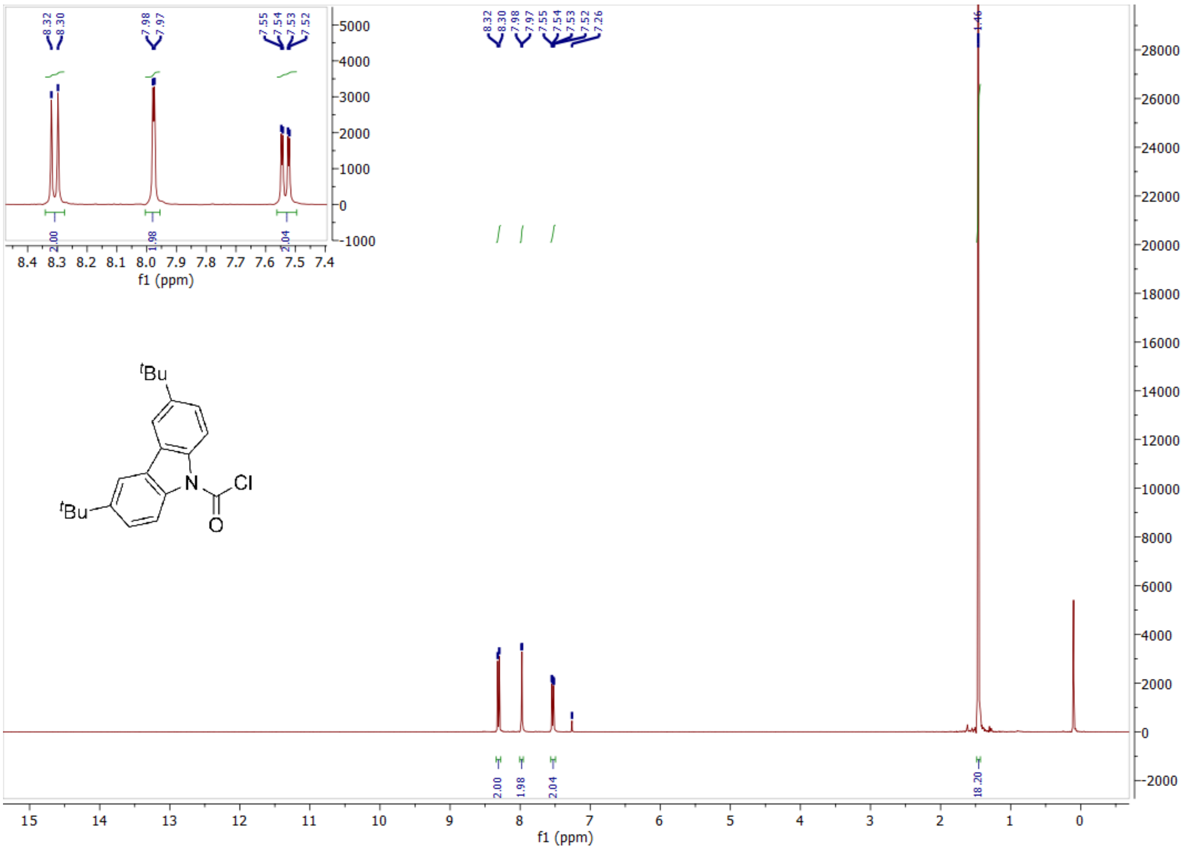

Figure S36. ^{1}H NMR (400 MHz, $CDCl_3$) spectrum of compound **5b**.

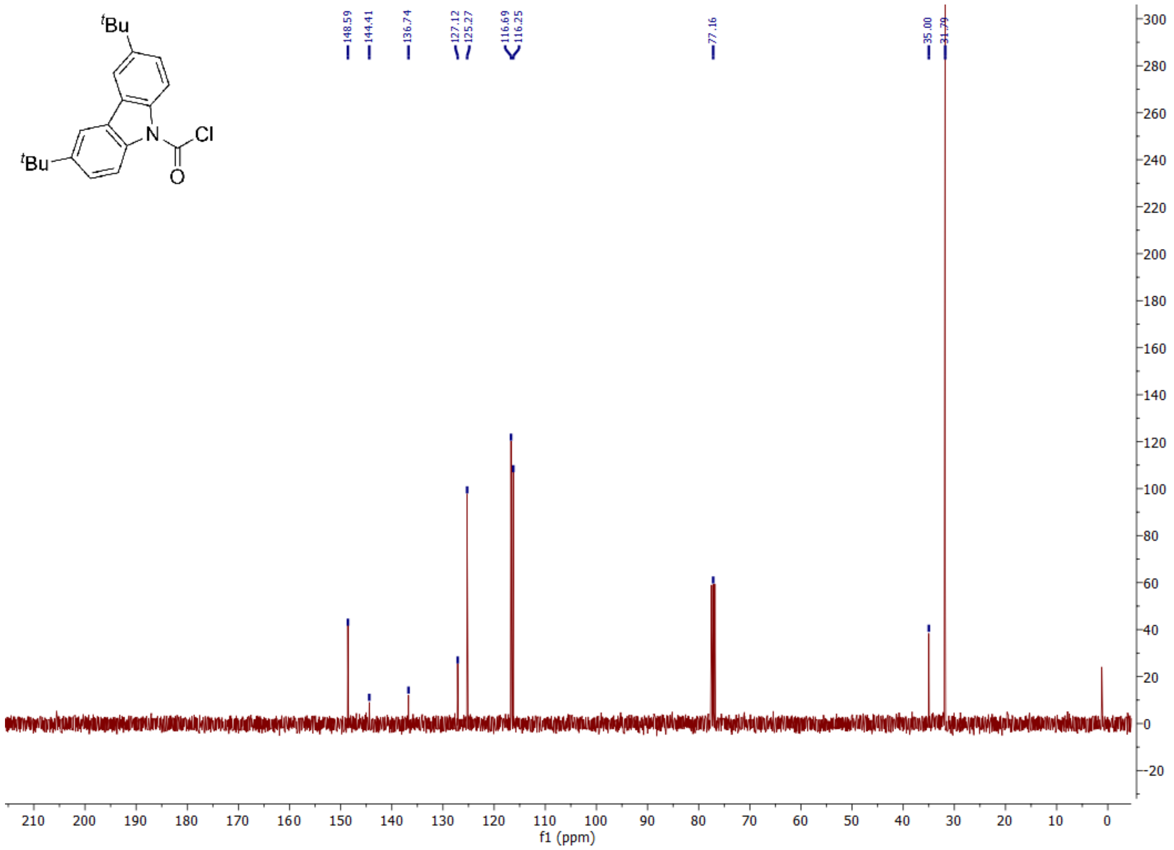

Figure S37. ^{13}C{H} NMR (100 MHz, $CDCl_3$) spectrum of compound **5b**.

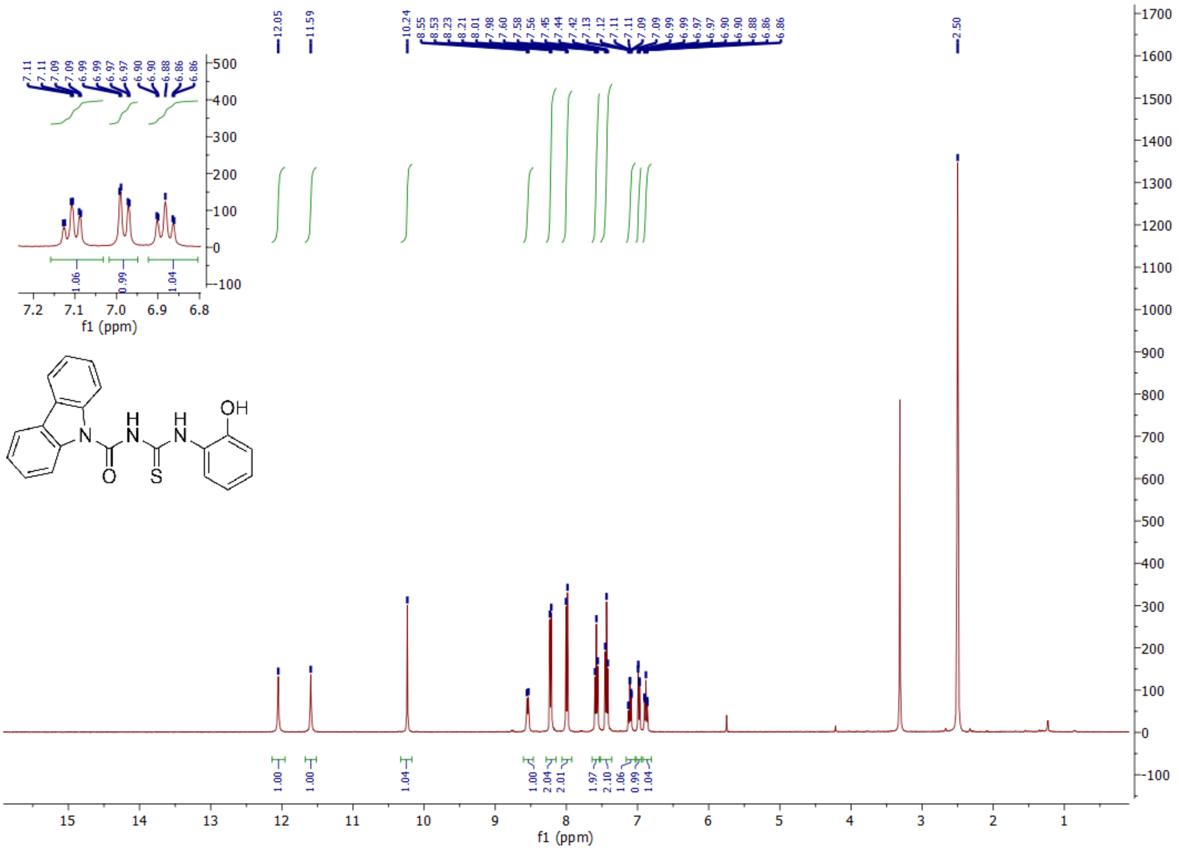

Figure S38. ^{1}H NMR (400 MHz, DMSO-d_6) spectrum of compound **10a**.

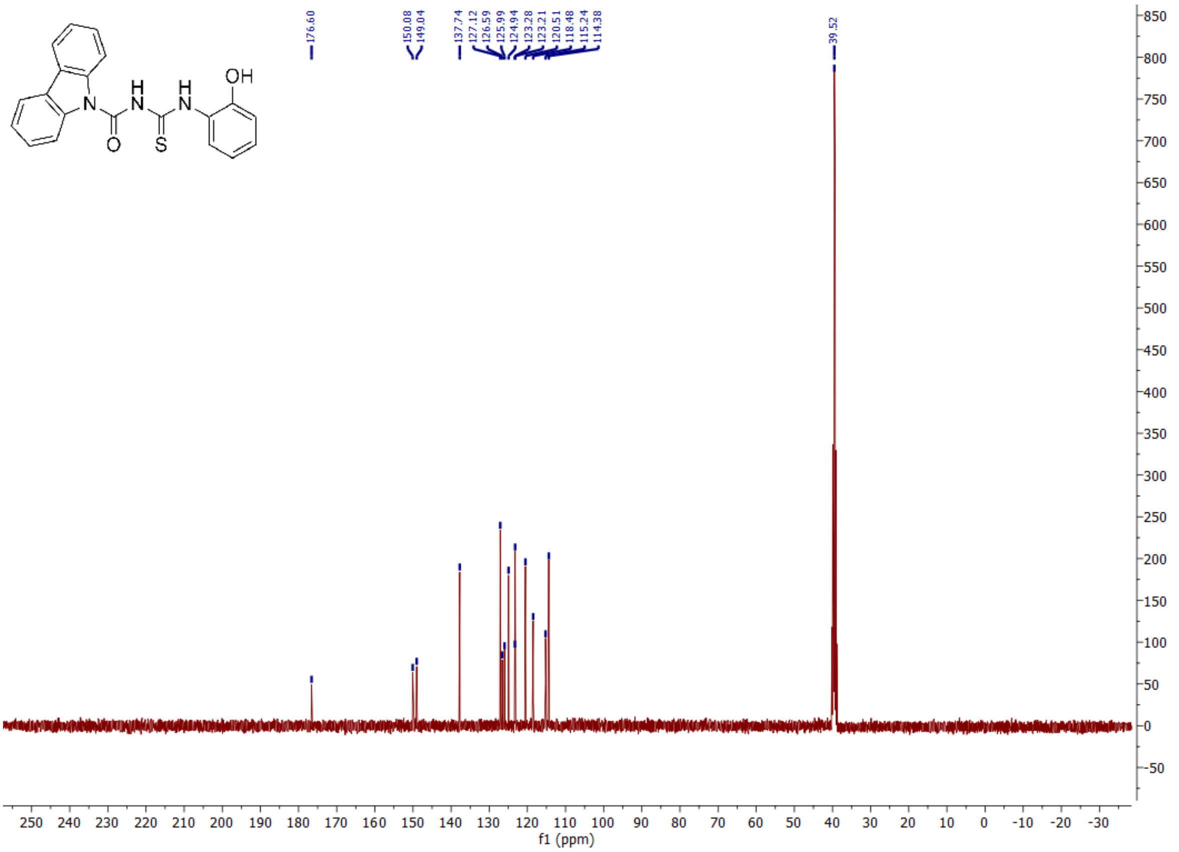

Figure S39. ^{13}C{H} NMR (100 MHz, DMSO-d_6) spectrum of compound **10a**.

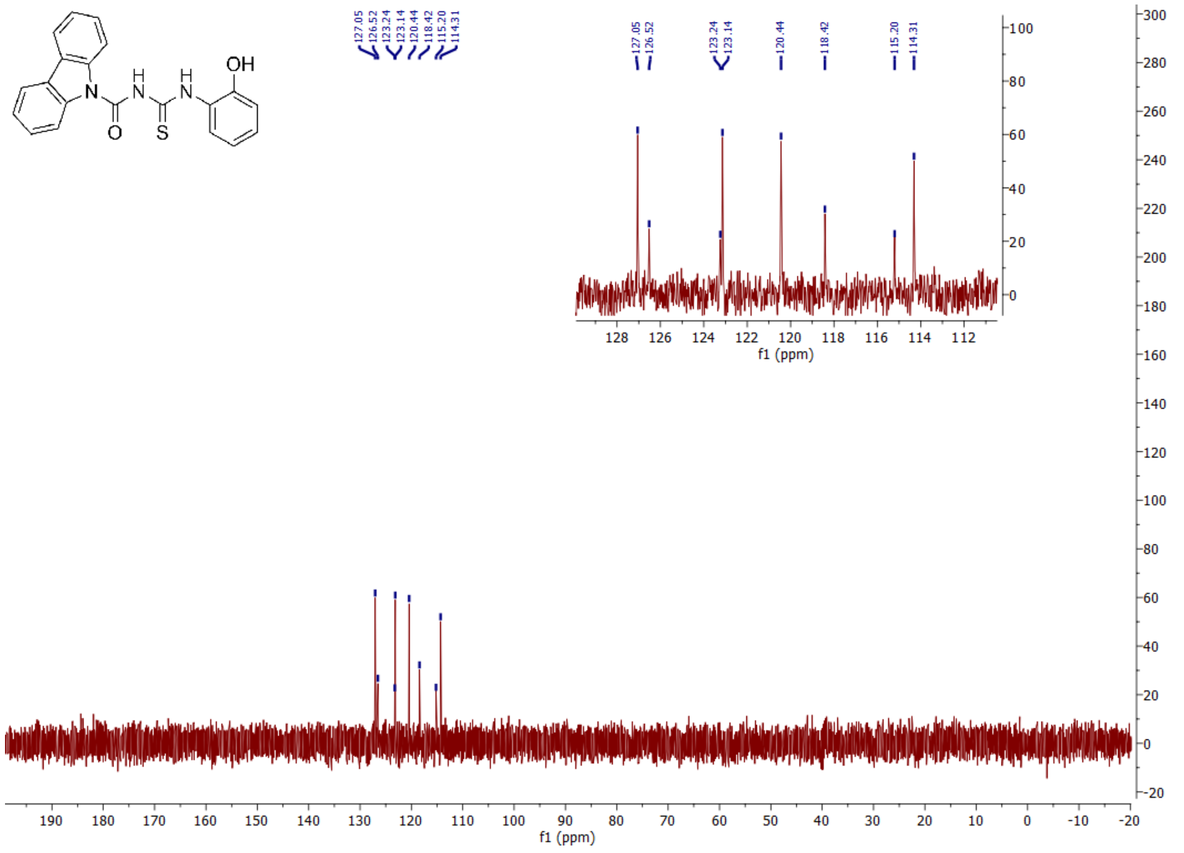

Figure S40. DEPT NMR (100 MHz, DMSO-d_6) spectrum of compound **10a**.

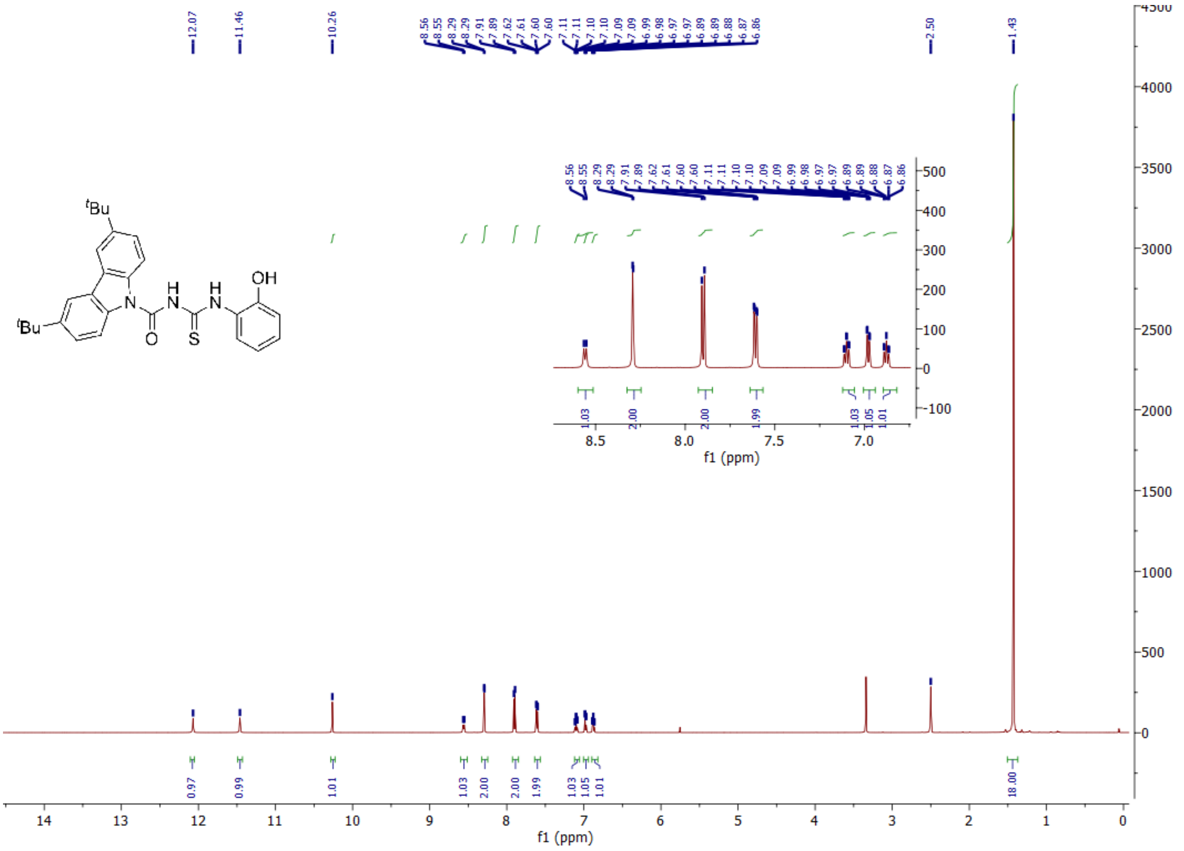

Figure S41. ^{1}H NMR (600 MHz, DMSO-d_6) spectrum of compound **10b**.

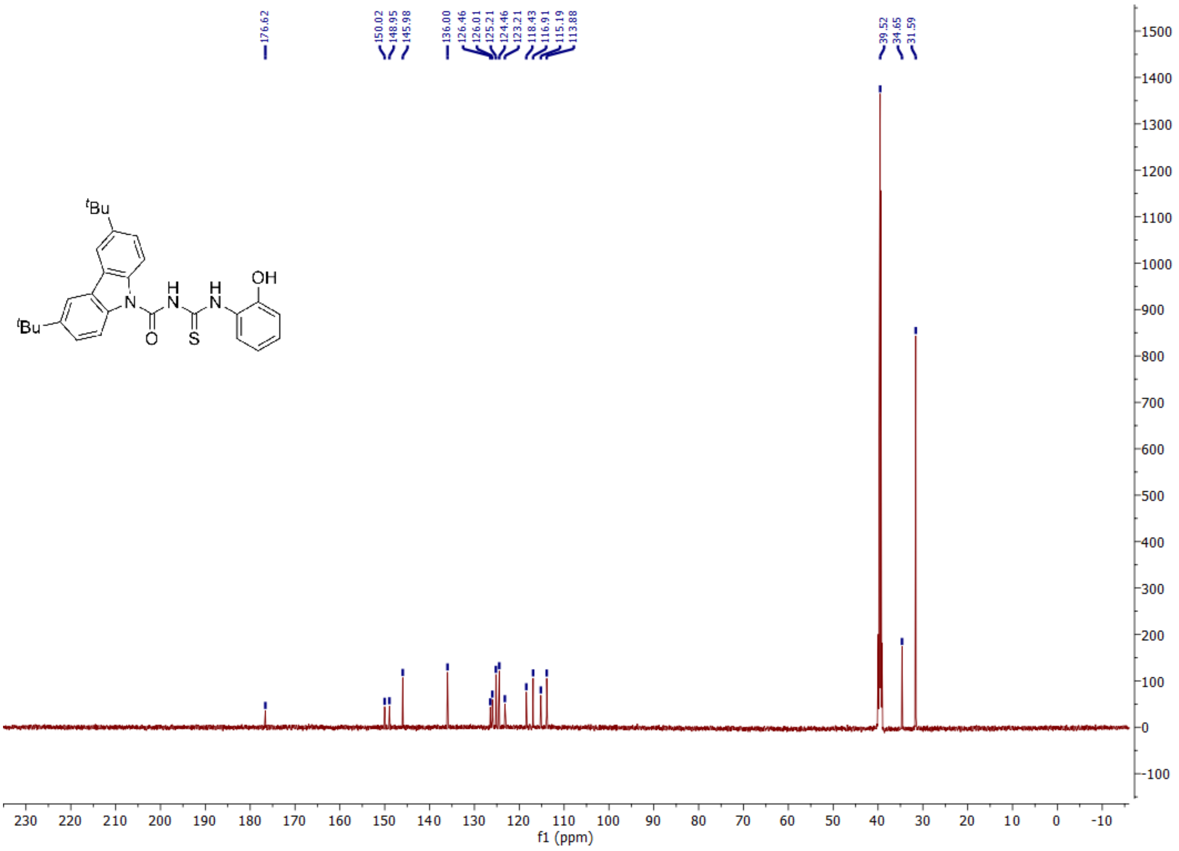

Figure S42. $^{13}C\{H\}$ NMR (150 MHz, DMSO-d_6) spectrum of compound **10b**.

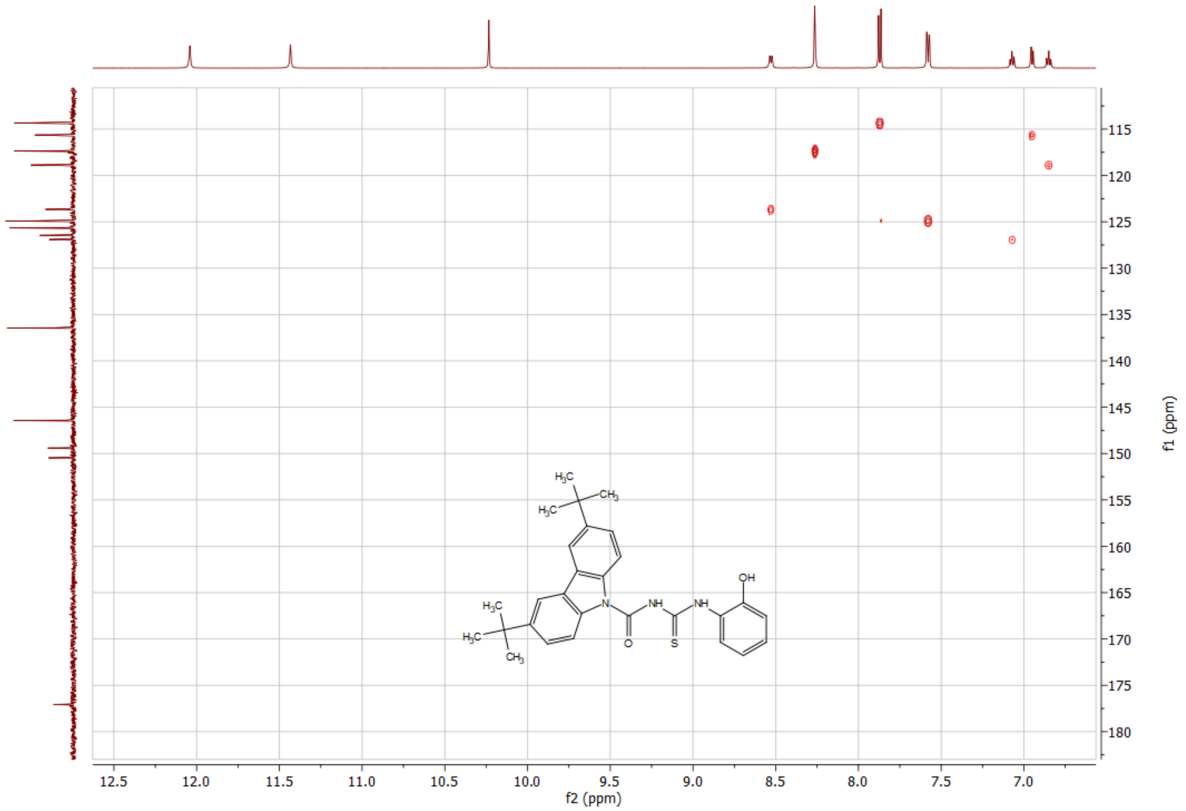

Figure S43. ^{1}H–^{13}C HSQC NMR (150 MHz, DMSO-d_6) spectrum of compound **10b**.

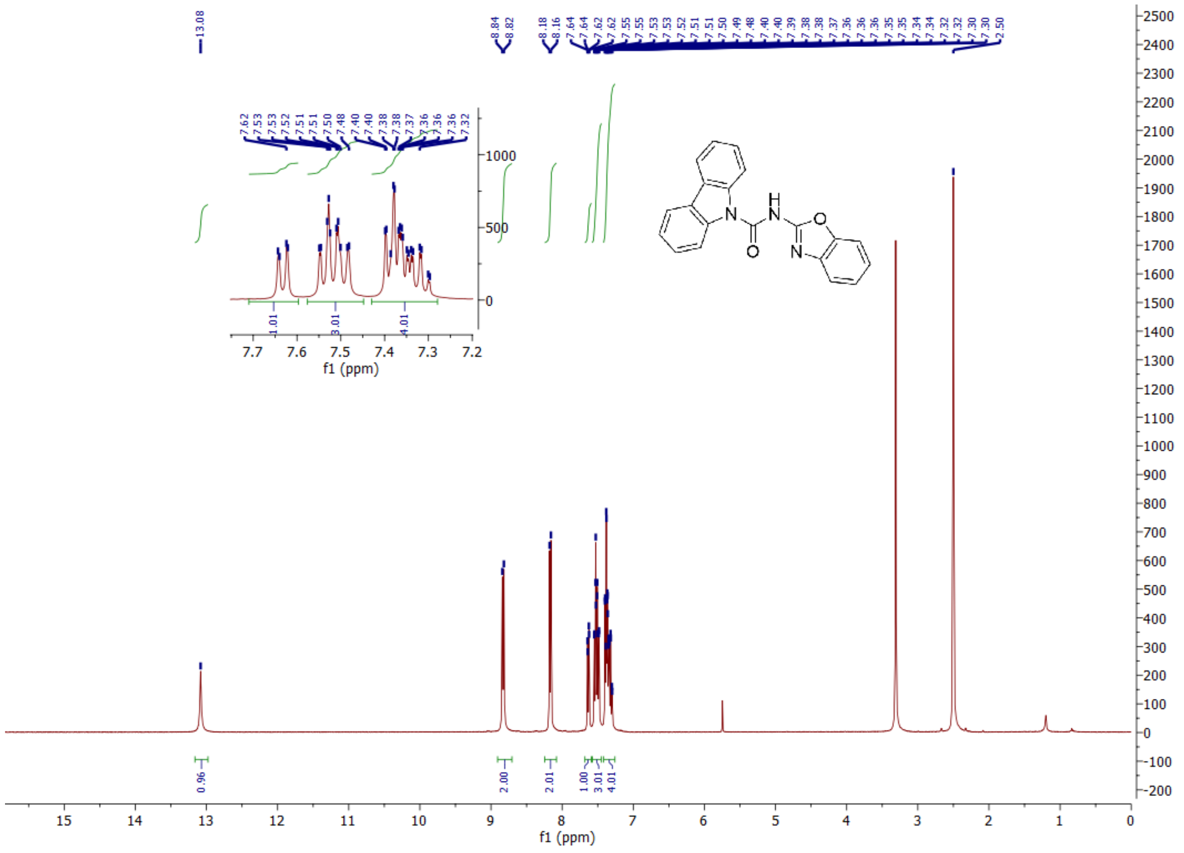

Figure S44. ^{1}H NMR (400 MHz, DMSO-d_6) spectrum of compound **14a**.

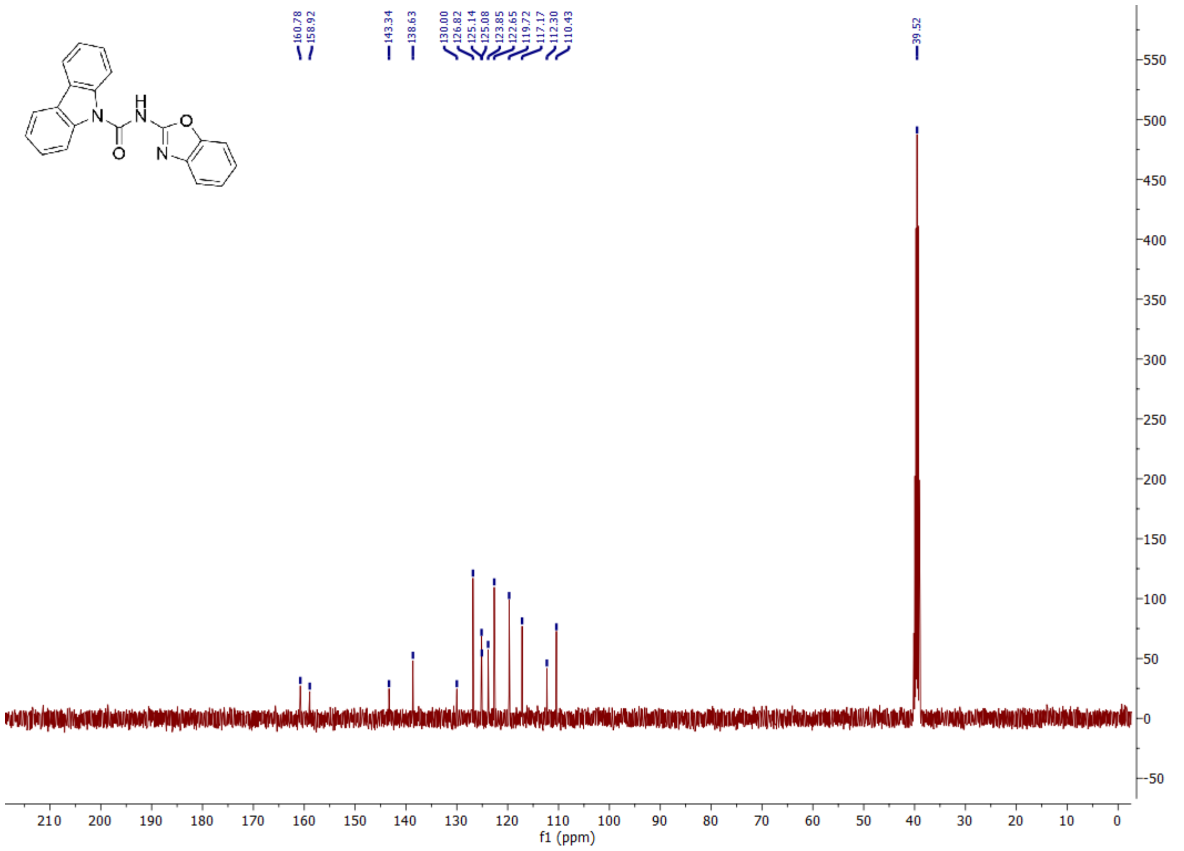

Figure S45. ^{13}C{H} NMR (100 MHz, DMSO-d_6) spectrum of compound **14a**.

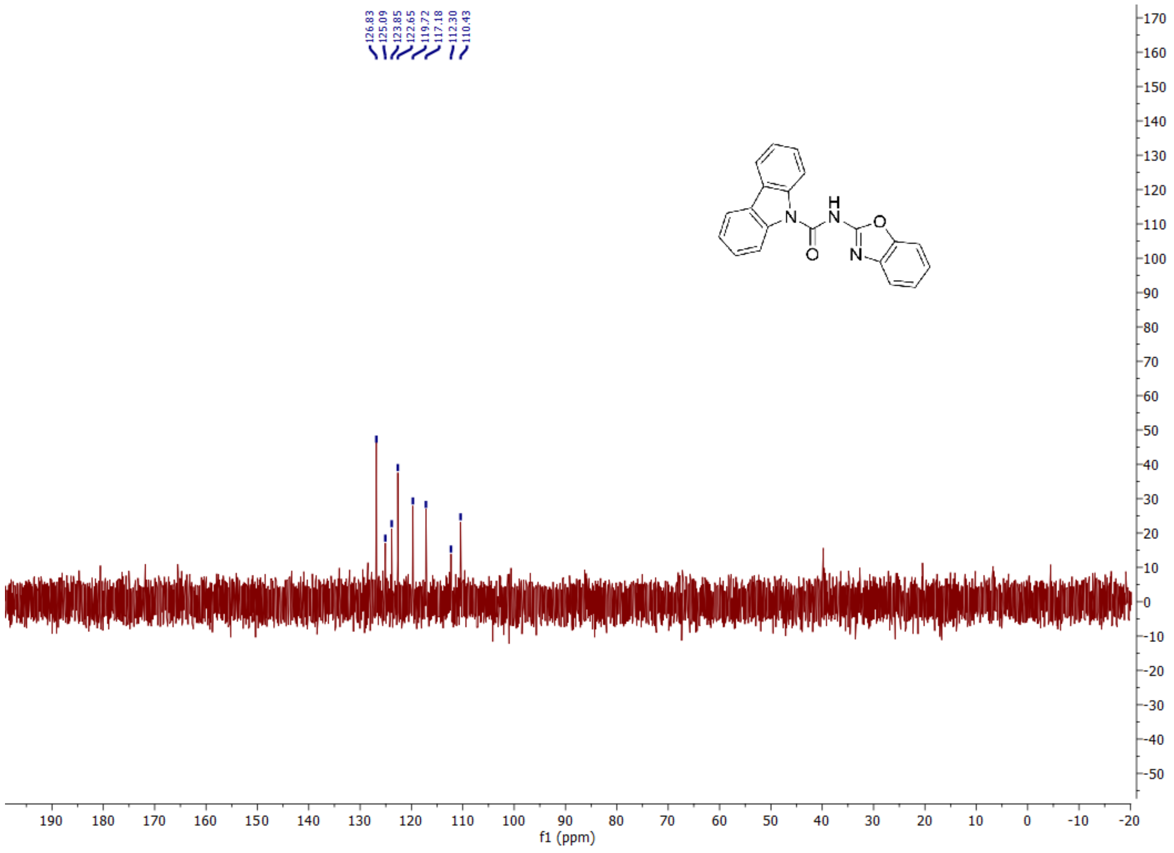

Figure S46. DEPT NMR (100 MHz, DMSO-d_6) spectrum of compound **14a**.

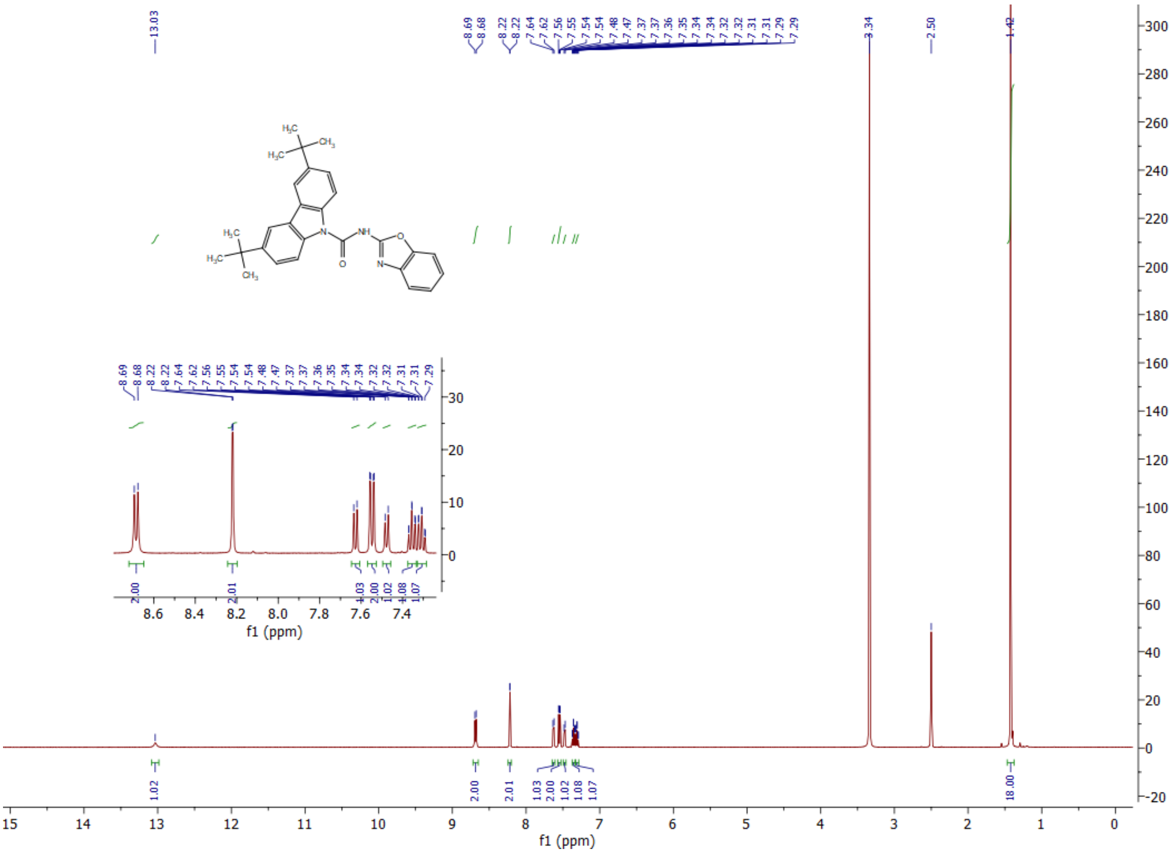

Figure S47. ^{1}H NMR (500 MHz, DMSO-d_6) spectrum of compound **14b**.

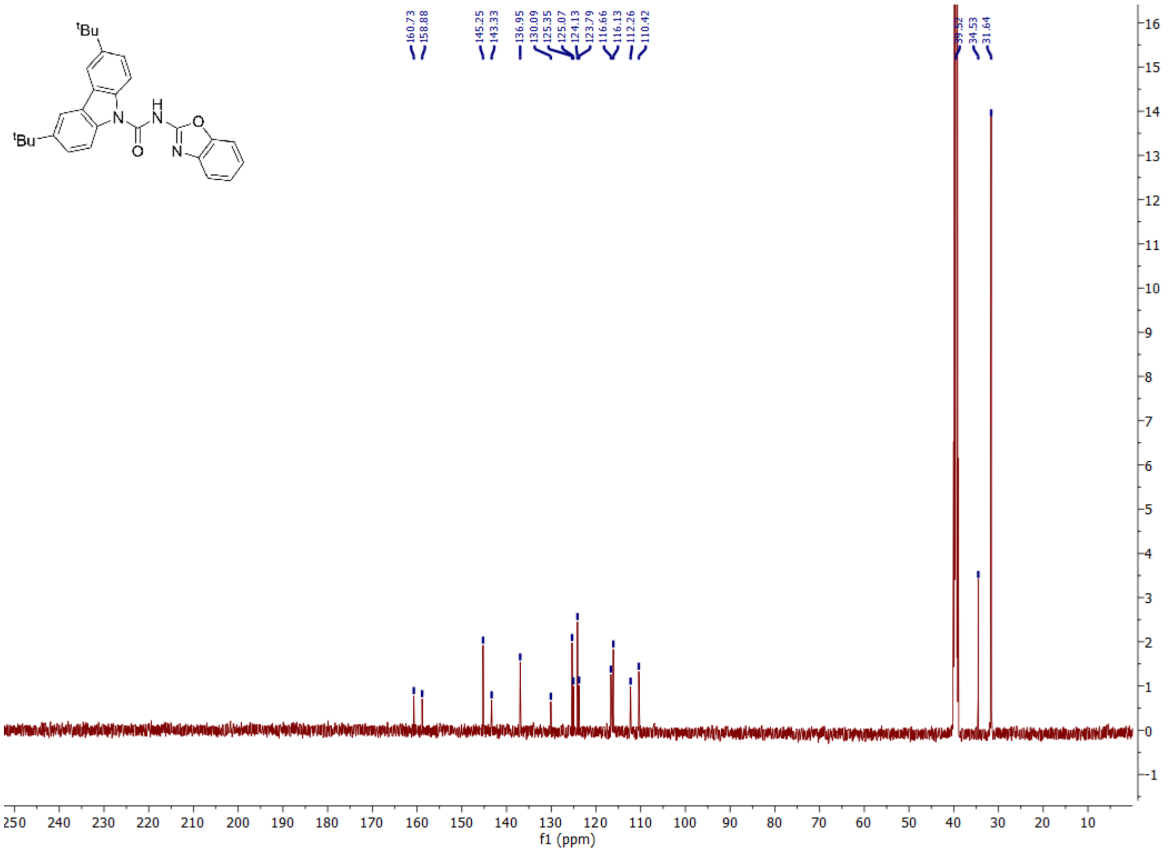

Figure S48. $^{13}C\{H\}$ NMR (125 MHz, DMSO-d_6) spectrum of compound **14b**.

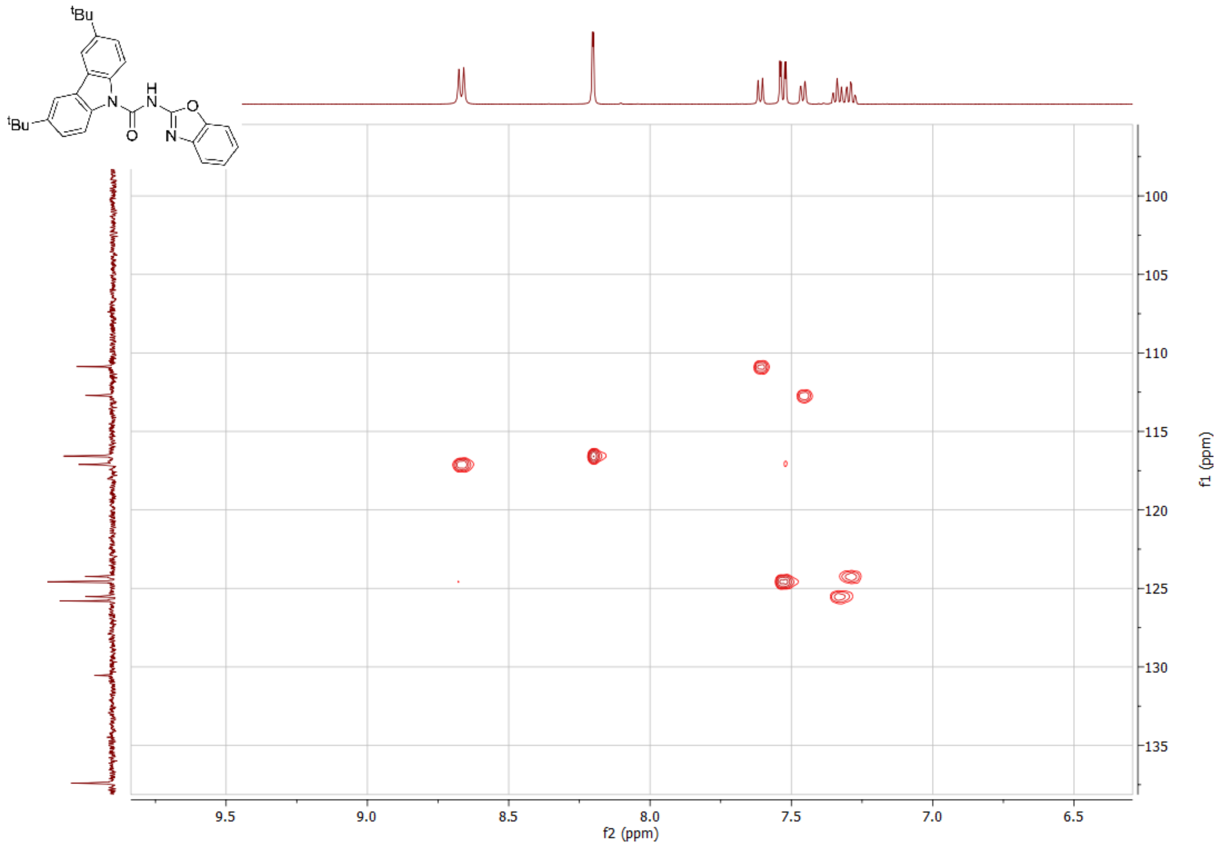

Figure S49. ^{1}H–^{13}C HSQC NMR (500 MHz, DMSO-d_6) spectrum of compound **14b.**

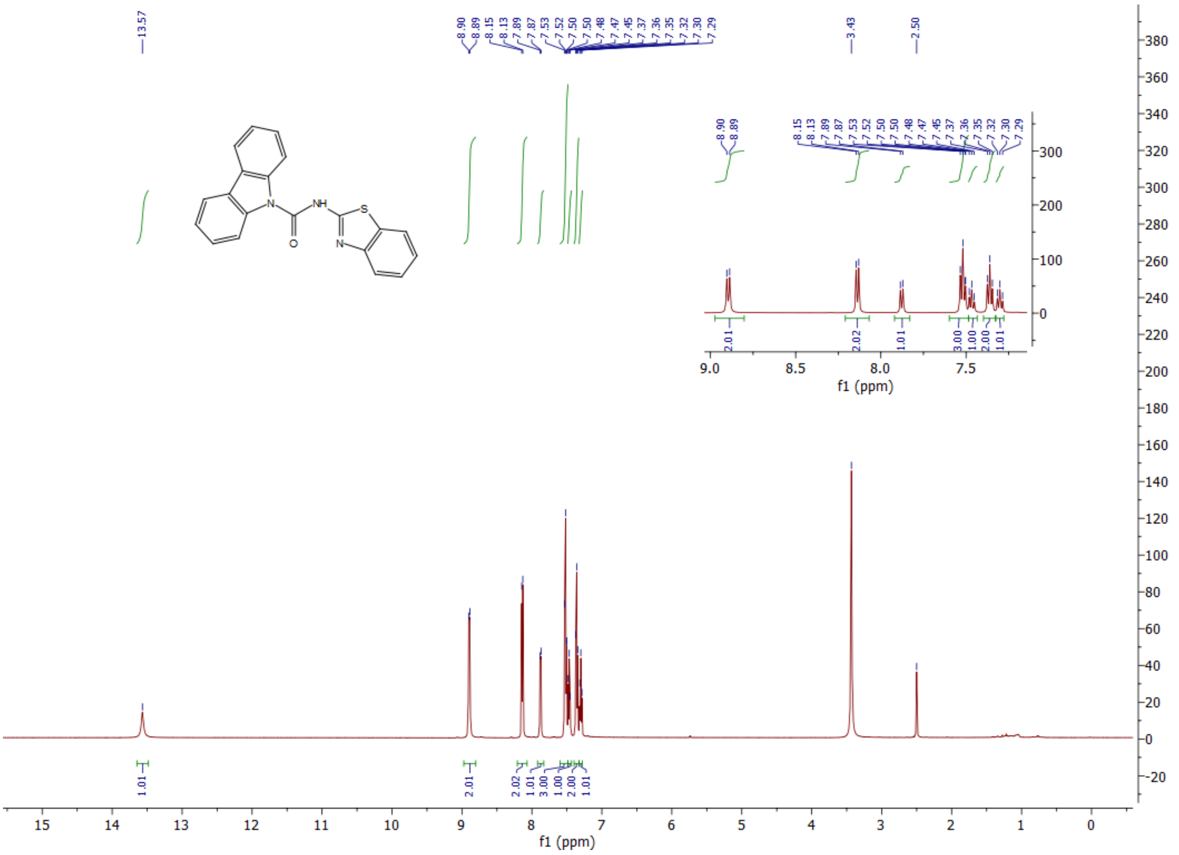

Figure S50. ^{1}H NMR (500 MHz, DMSO-d_6) spectrum of compound **15a**.

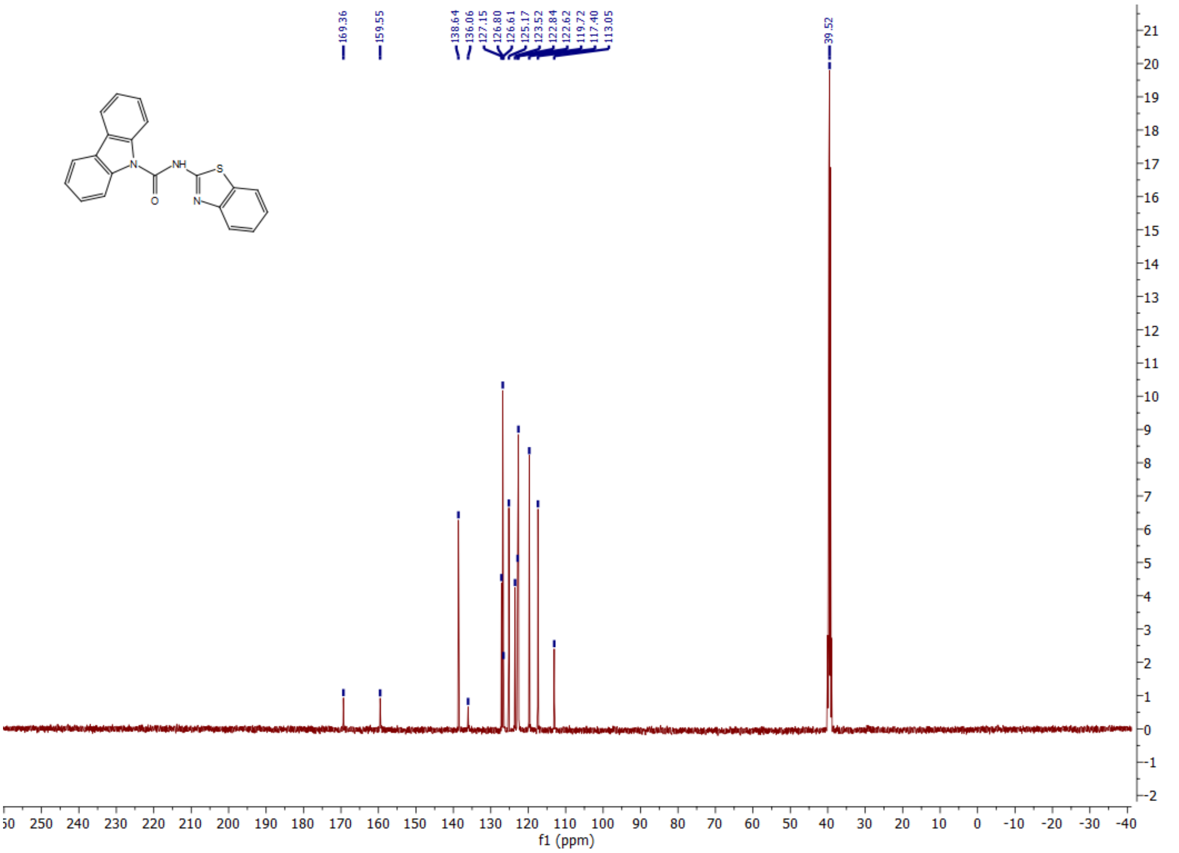

Figure S51. ^{13}C{H} NMR (125 MHz, DMSO-d_6) spectrum of compound **15a**.

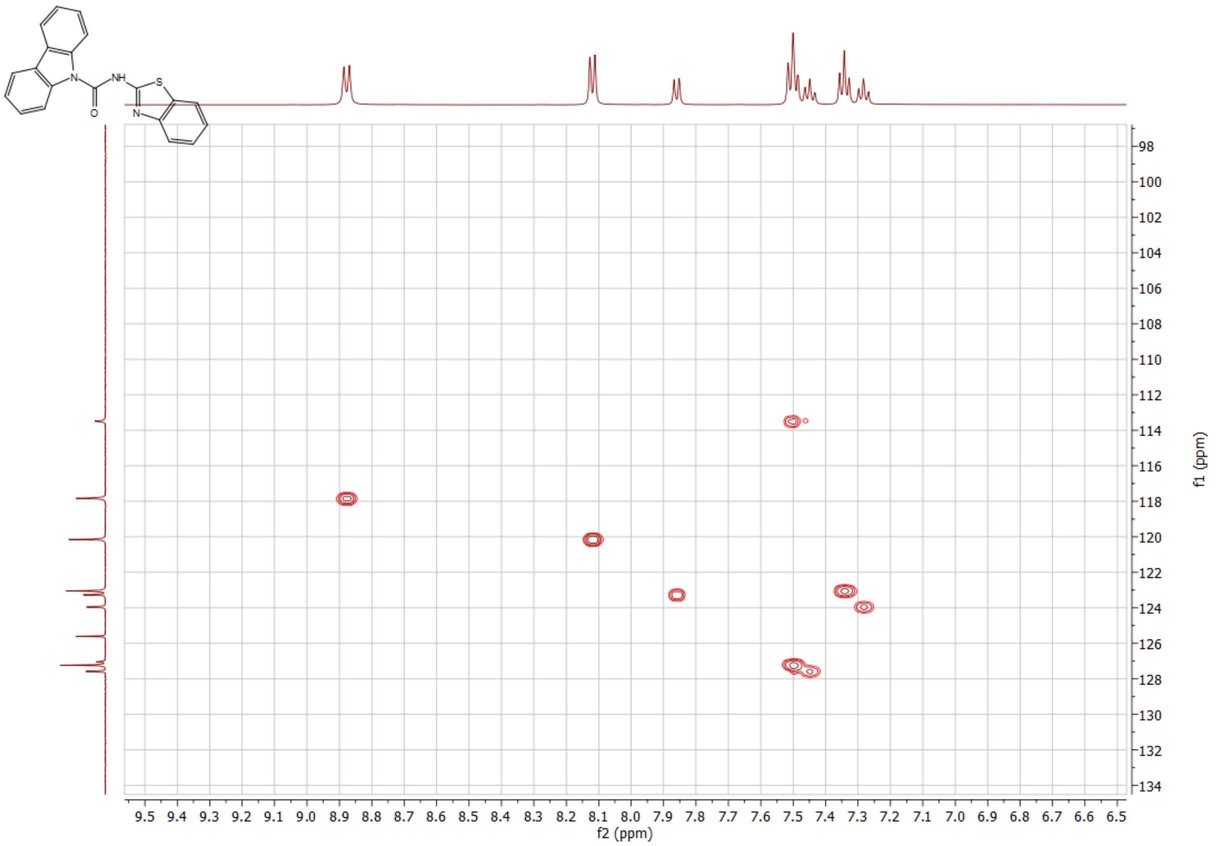

Figure S52. ^{1}H–^{13}C HSQC NMR (500 MHz, DMSO-d_6) spectrum of compound **15a.**

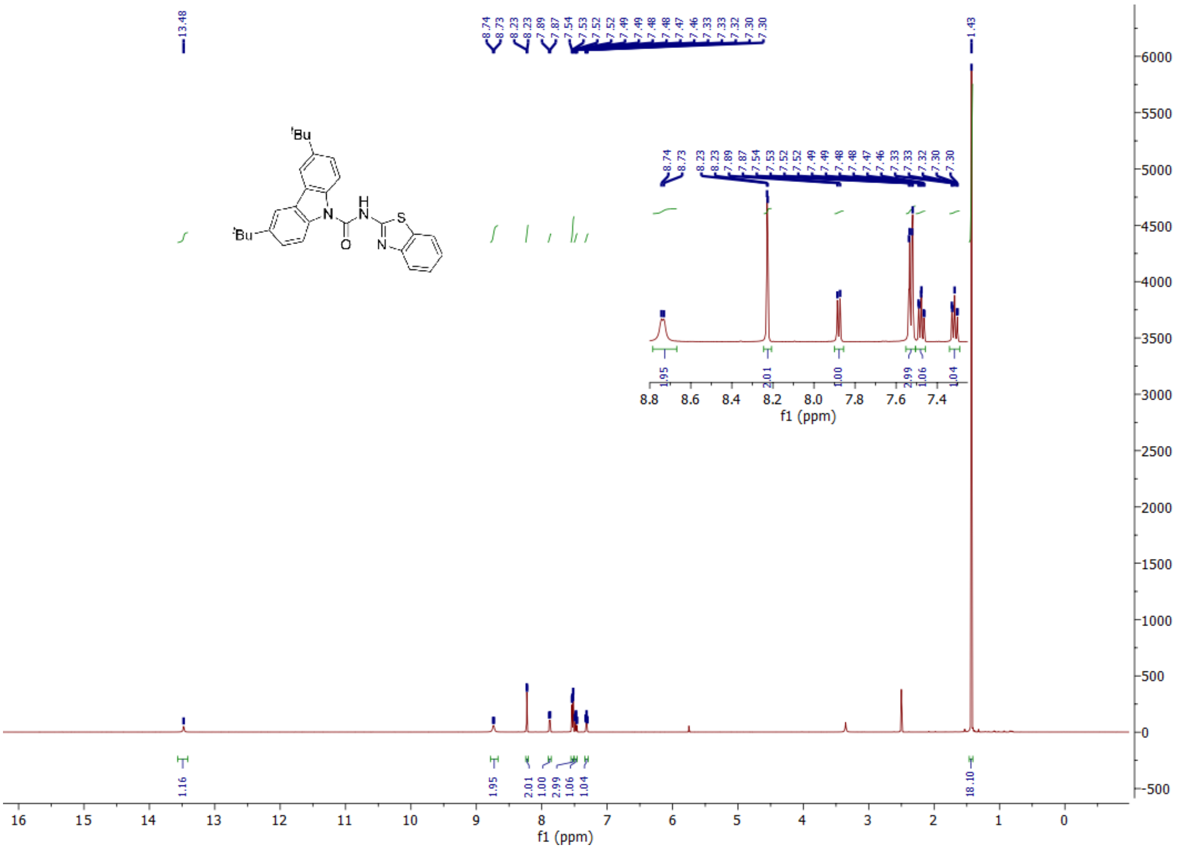

Figure S53. ^{1}H NMR (600 MHz, DMSO-d_6) spectrum of compound **15b**.

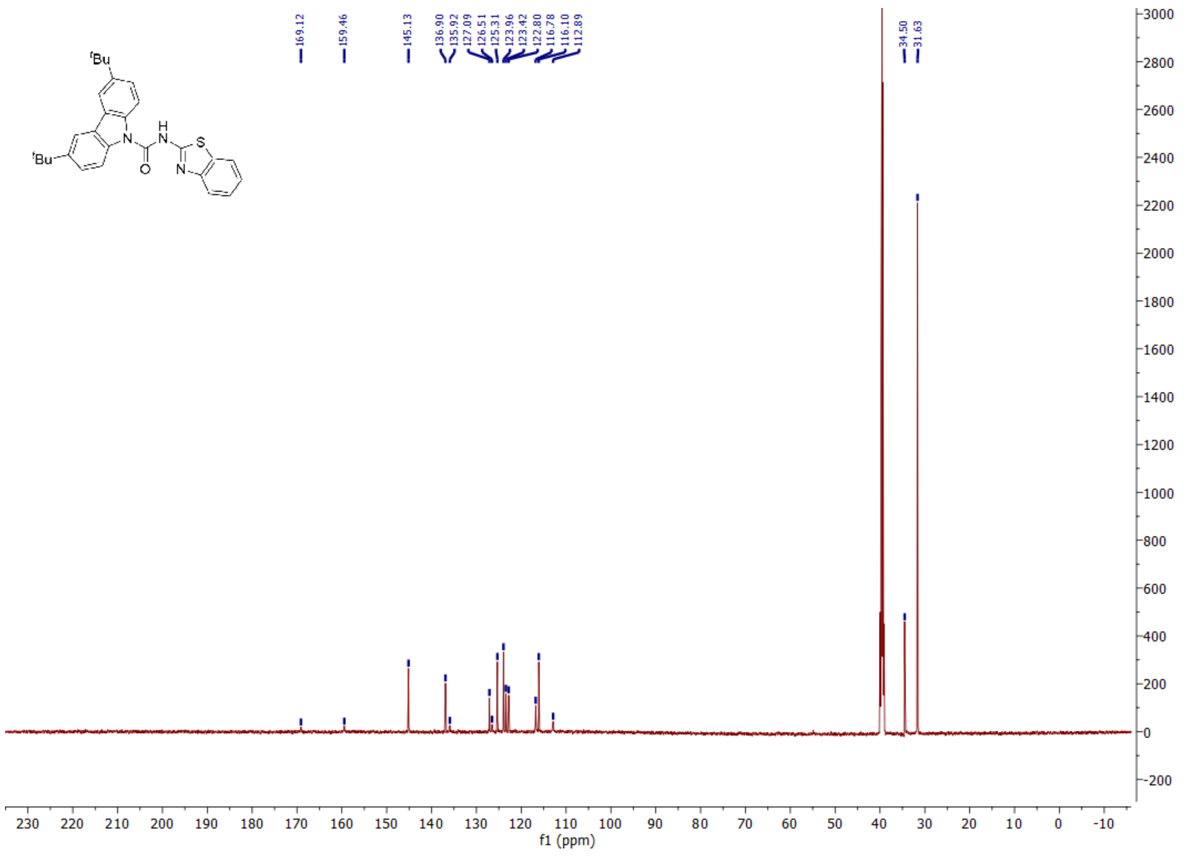

Figure S54. $^{13}C\{H\}$ NMR (150 MHz, DMSO-d_6) spectrum of compound **15b**.

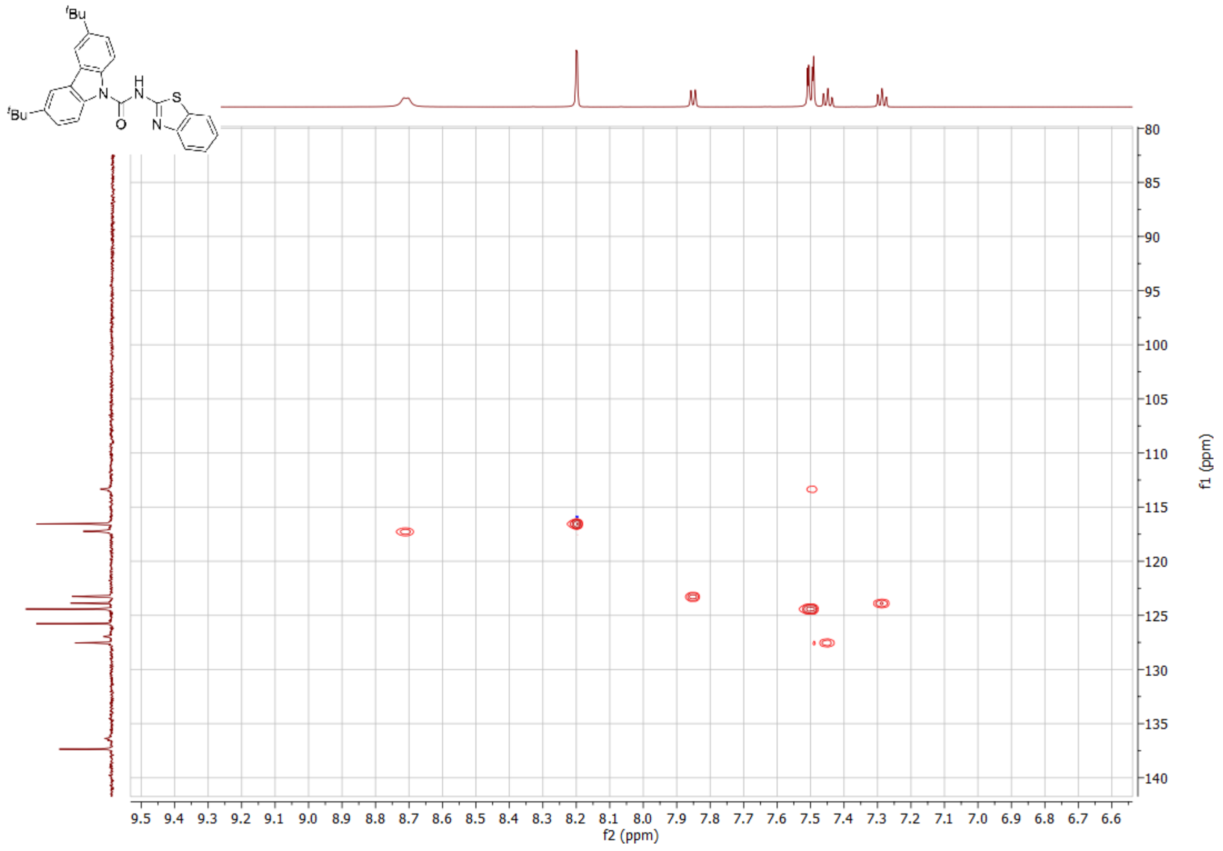

Figure S55. ^{1}H–^{13}C HSQC NMR (600 MHz, DMSO-d_6) spectrum of compound **15b.**

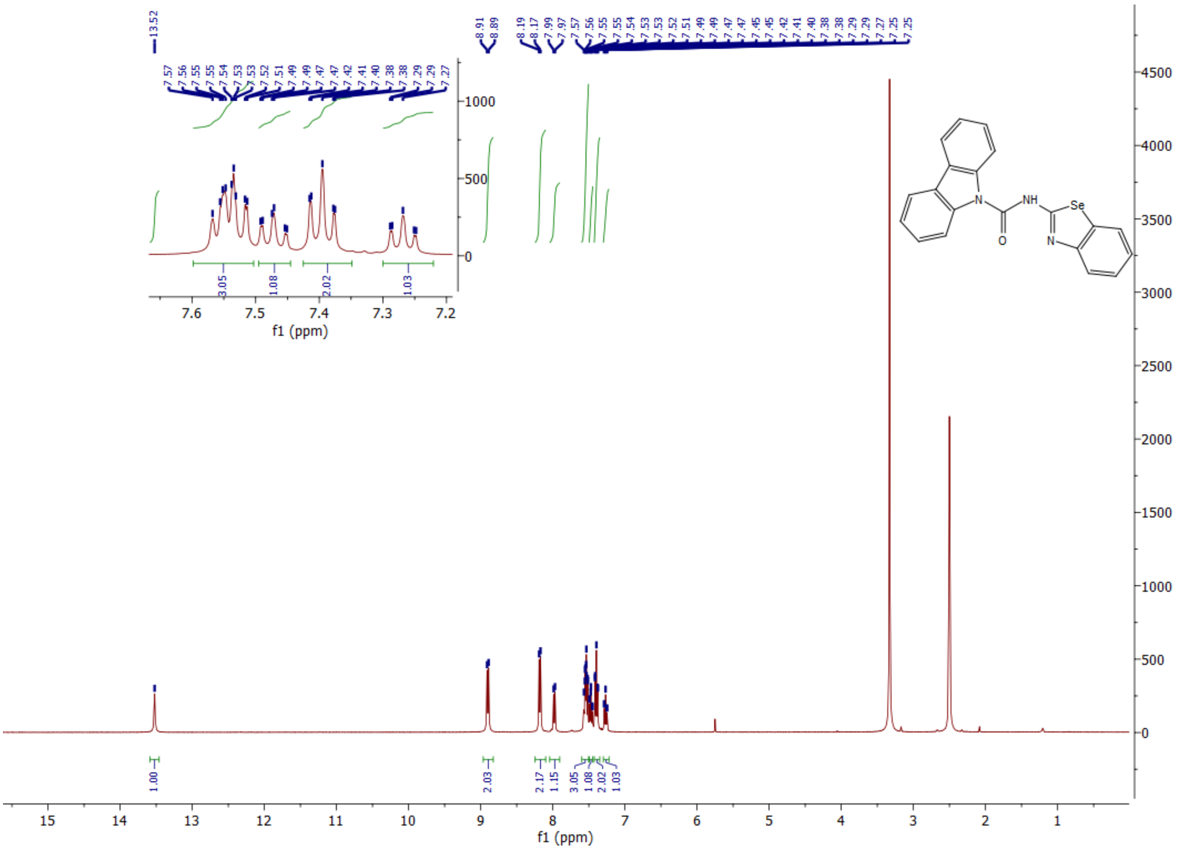

Figure S56. ^{1}H NMR (500 MHz, DMSO-d_6) spectrum of compound **16a**.

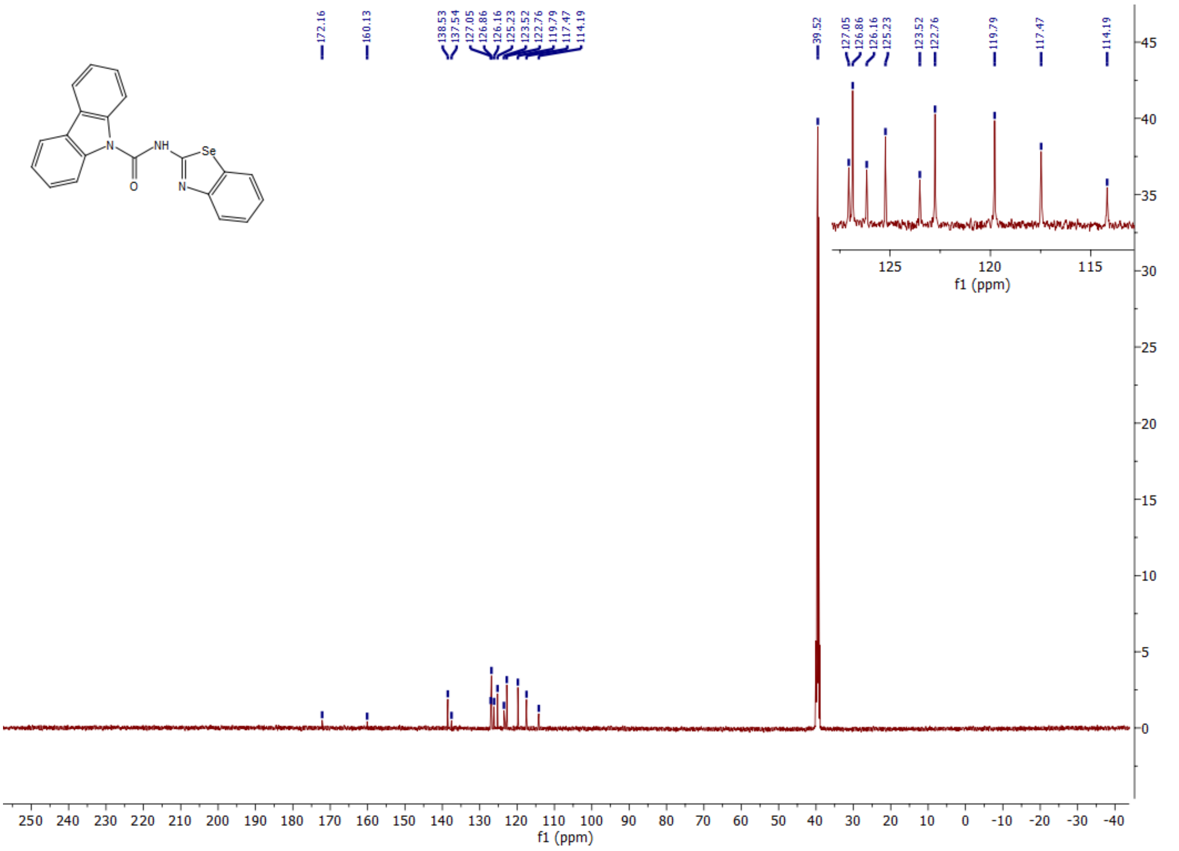

Figure S57. ^{13}C{H} NMR (125 MHz, DMSO-d_6) spectrum of compound **16a**.

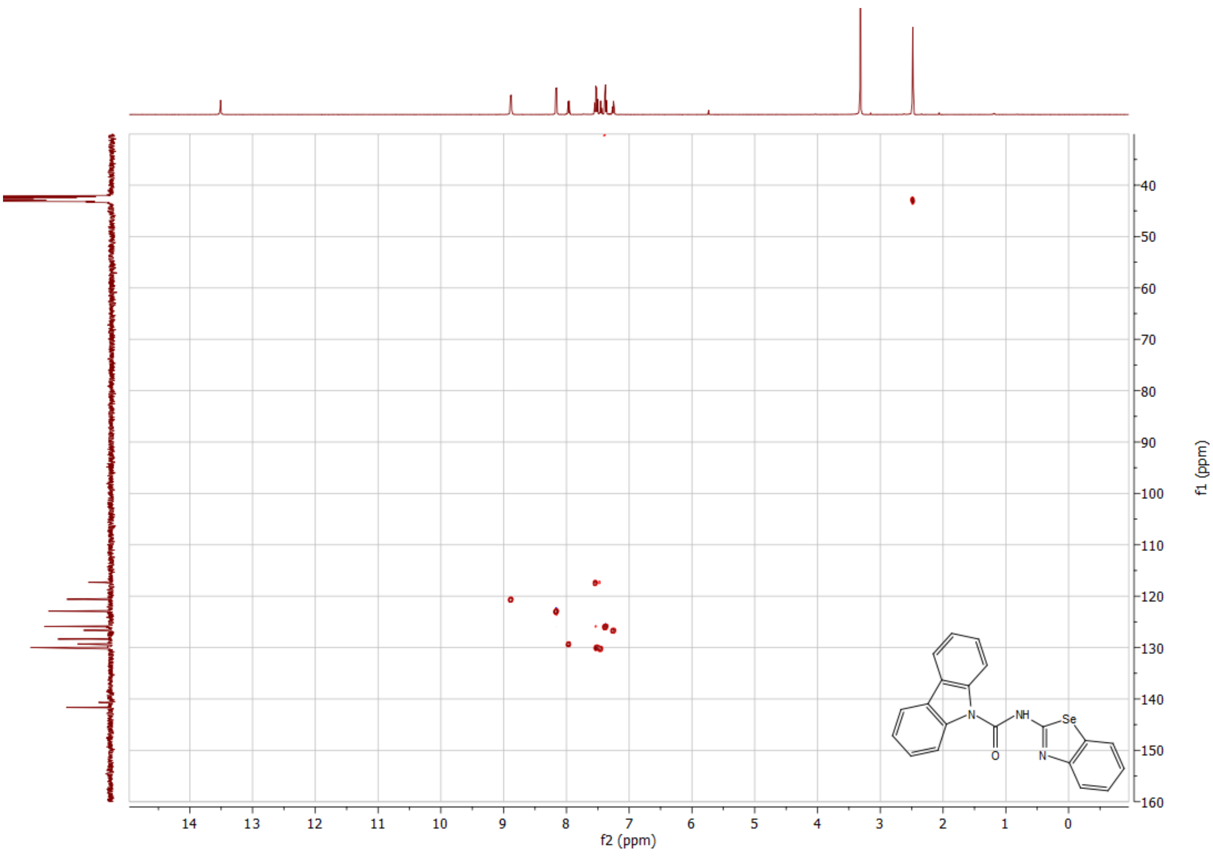

Figure S58. ¹H–¹³C HSQC NMR (125 MHz, DMSO-d_6) spectrum of compound **16a**.

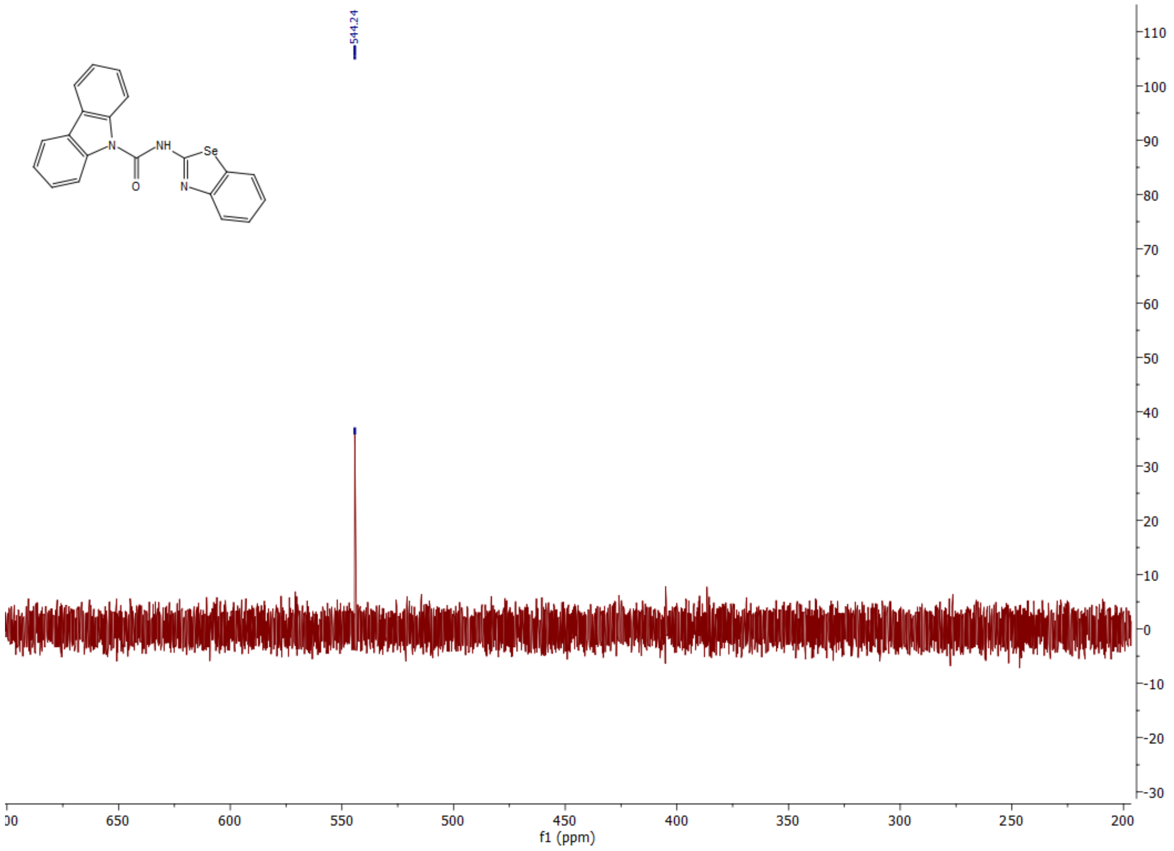

Figure S59. ⁷⁷Se NMR (95 MHz, DMSO-d_6) spectrum of compound **16a**.

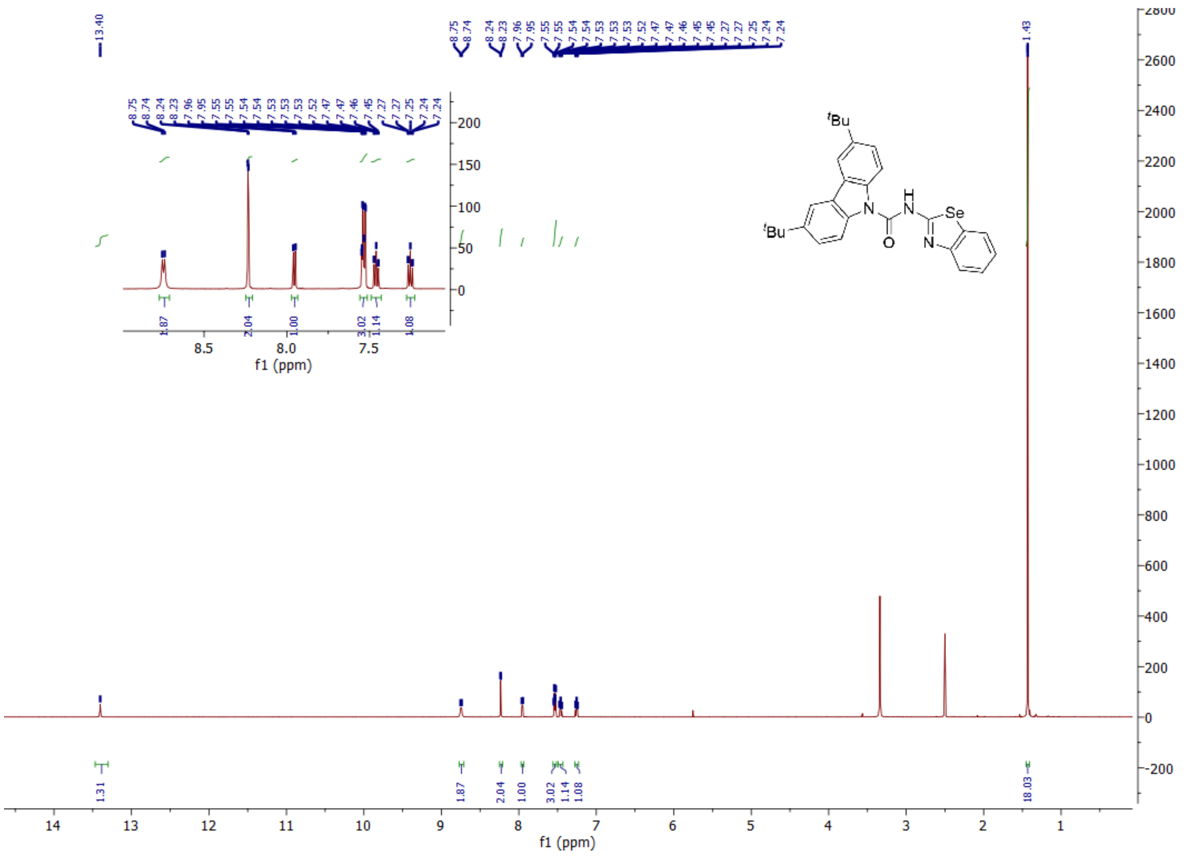

Figure S60. ^{1}H NMR (600 MHz, DMSO-d_6) spectrum of compound **16b**.

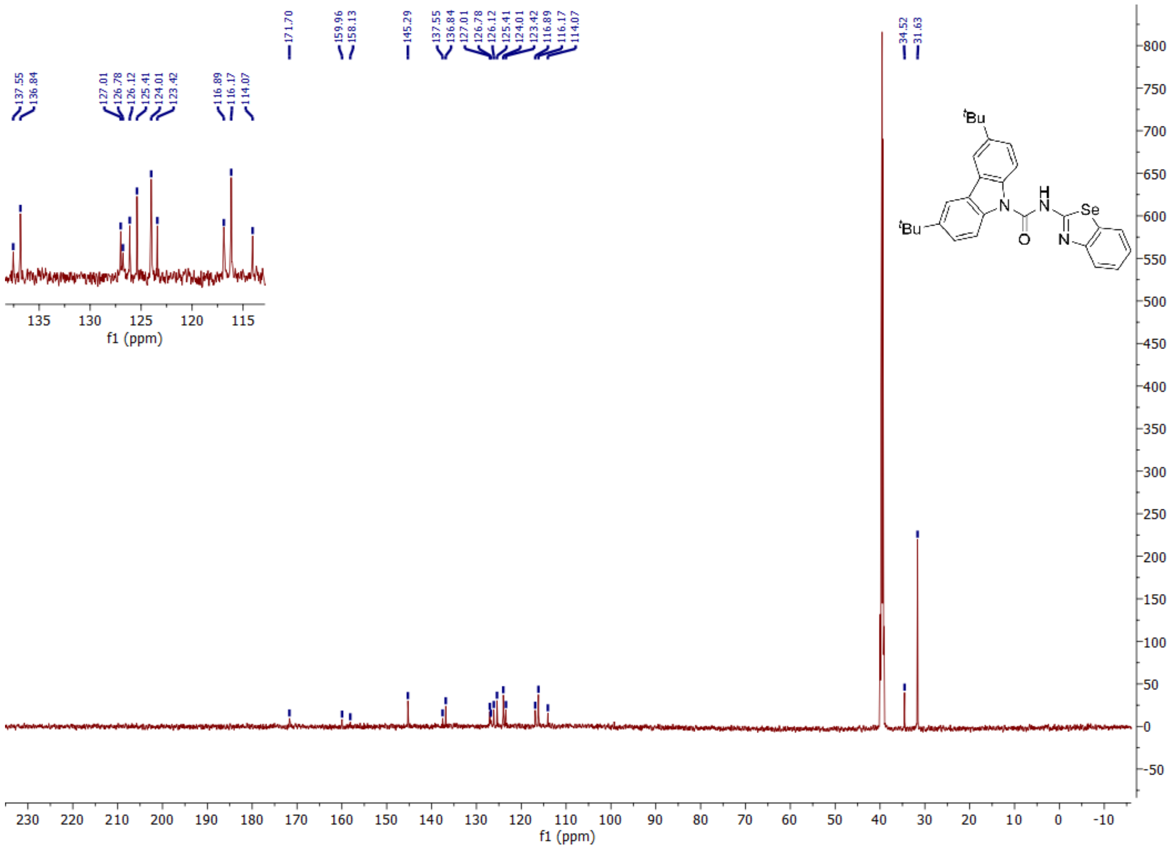

Figure S61. $^{13}C\{H\}$ NMR (150 MHz, DMSO-d_6) spectrum of compound **16b**.

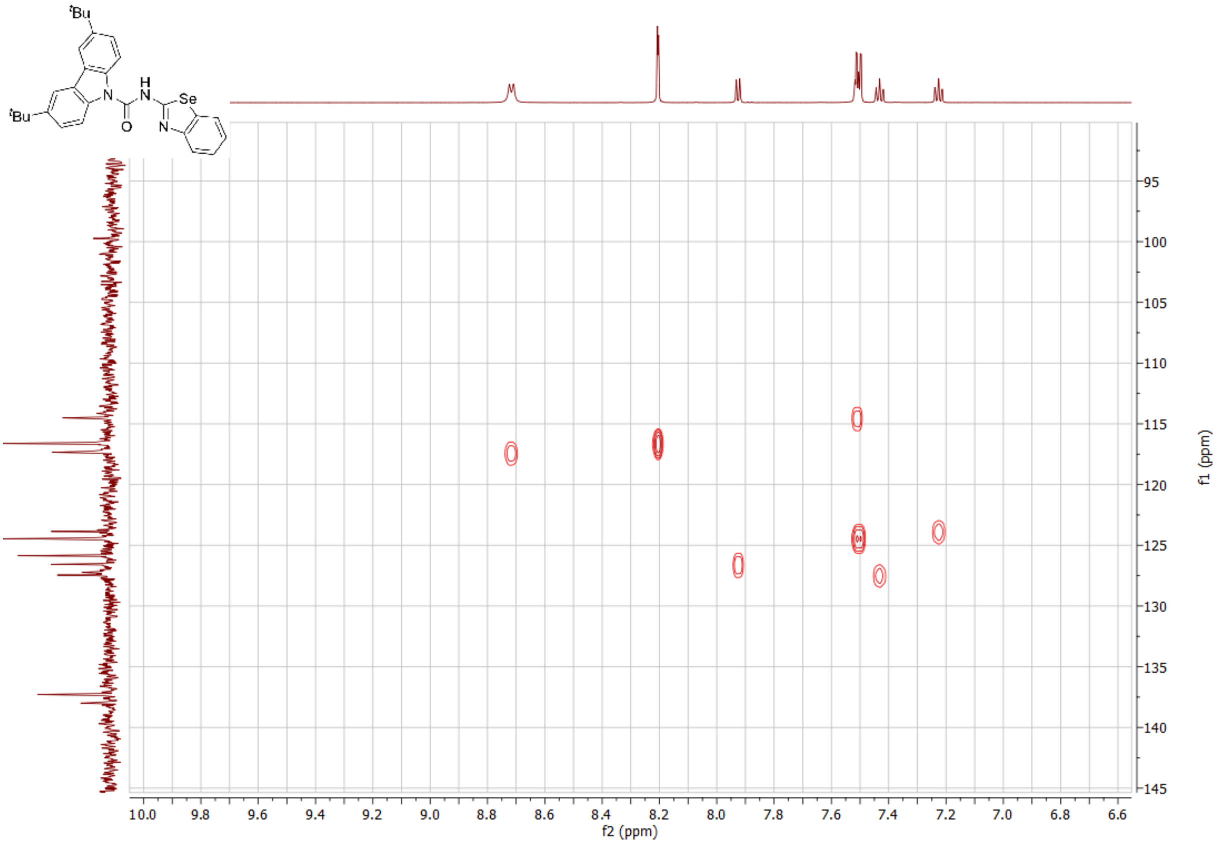

Figure S62. ^{1}H–^{13}C HSQC NMR (600 MHz, DMSO-d_6) spectrum of compound **16b**.

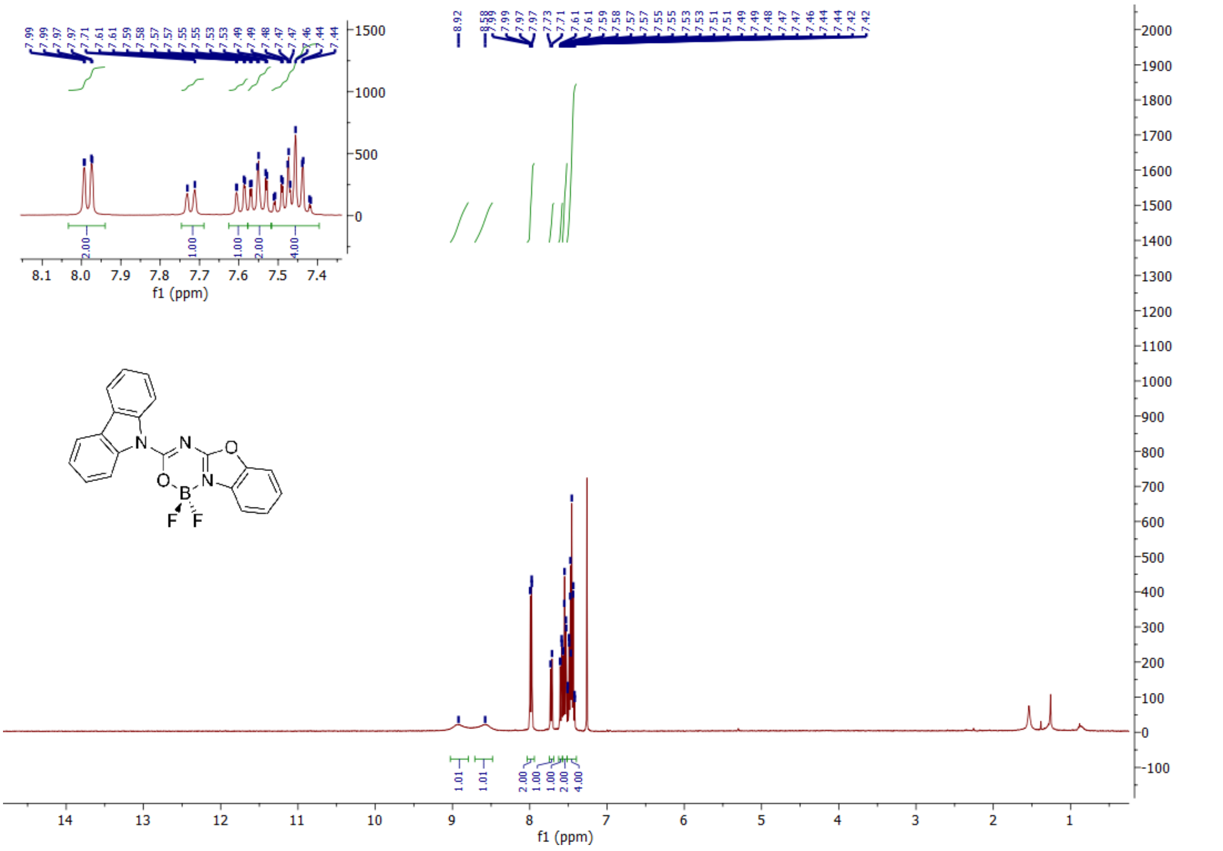

Figure S63. ^{1}H NMR (600 MHz, $CDCl_3$) spectrum of compound **1a**.

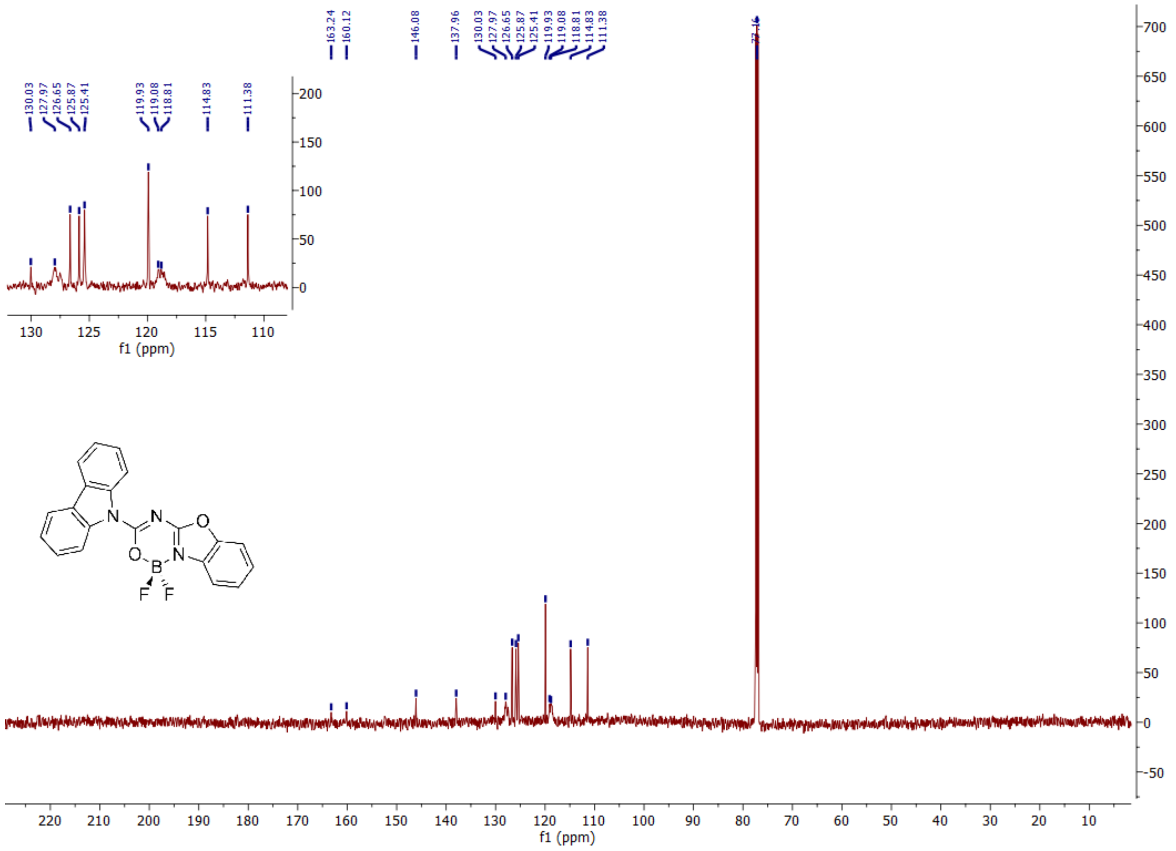

Figure S64. $^{13}C\{H\}$ NMR (150 MHz, $CDCl_3$) spectrum of compound **1a**.

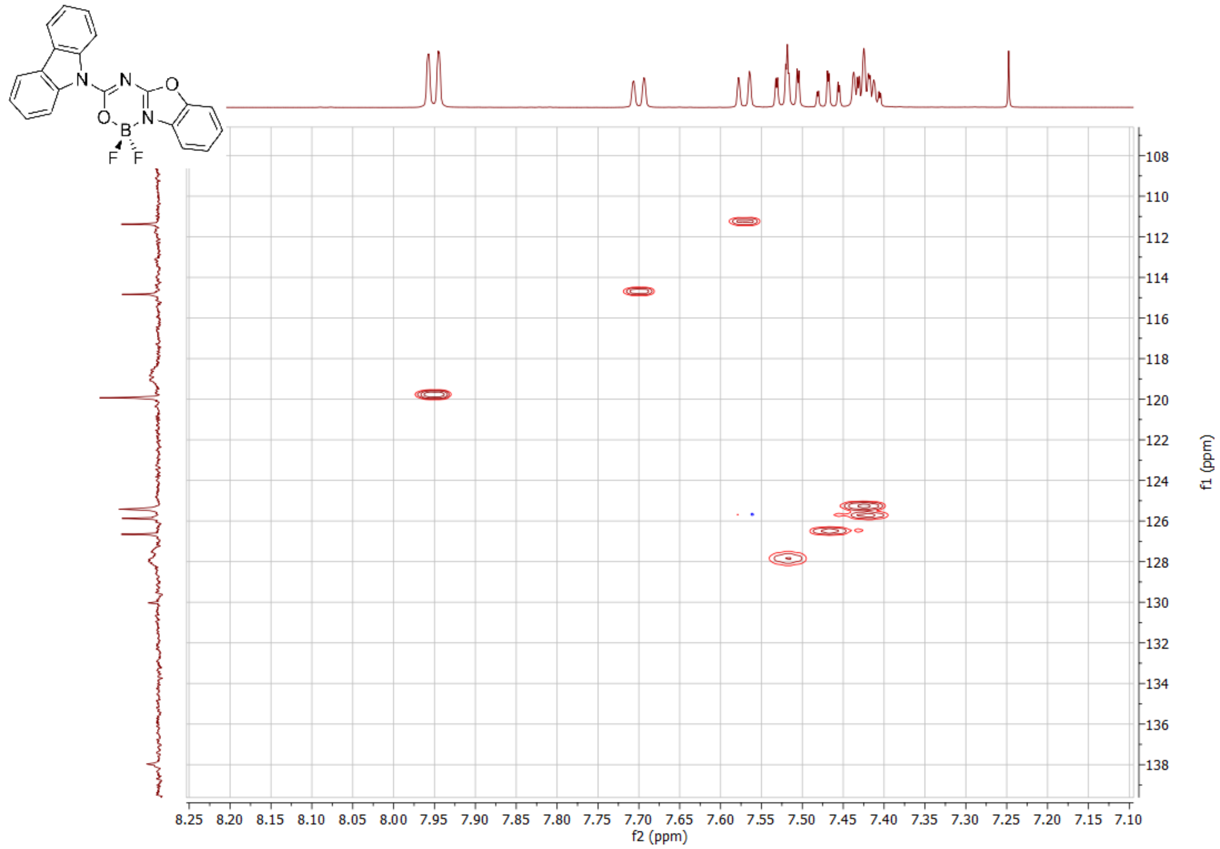

Figure S65. $^{1}H–^{13}C$ HSQC NMR (600 MHz, $CDCl_3$) spectrum of compound **1a**.

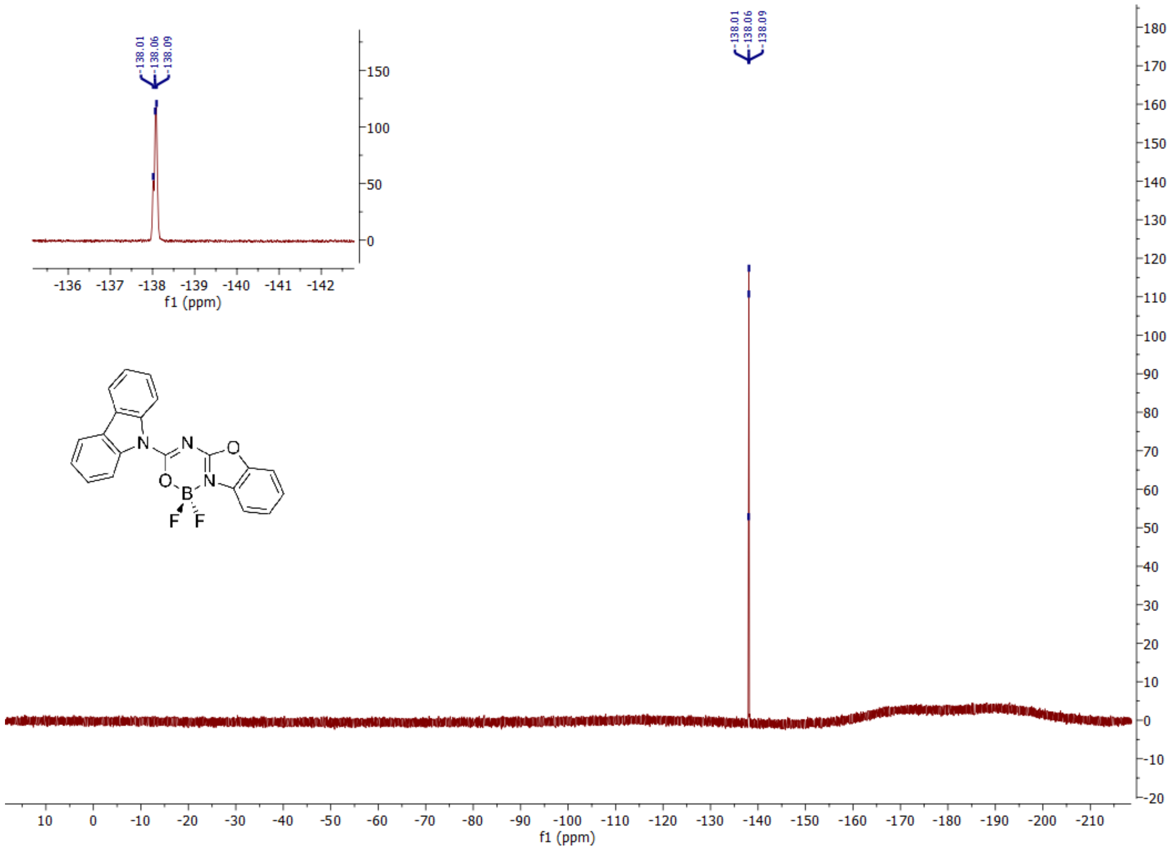

Figure S66. ^{19}F NMR (375 MHz, $CDCl_3$) spectrum of compound **1a**.

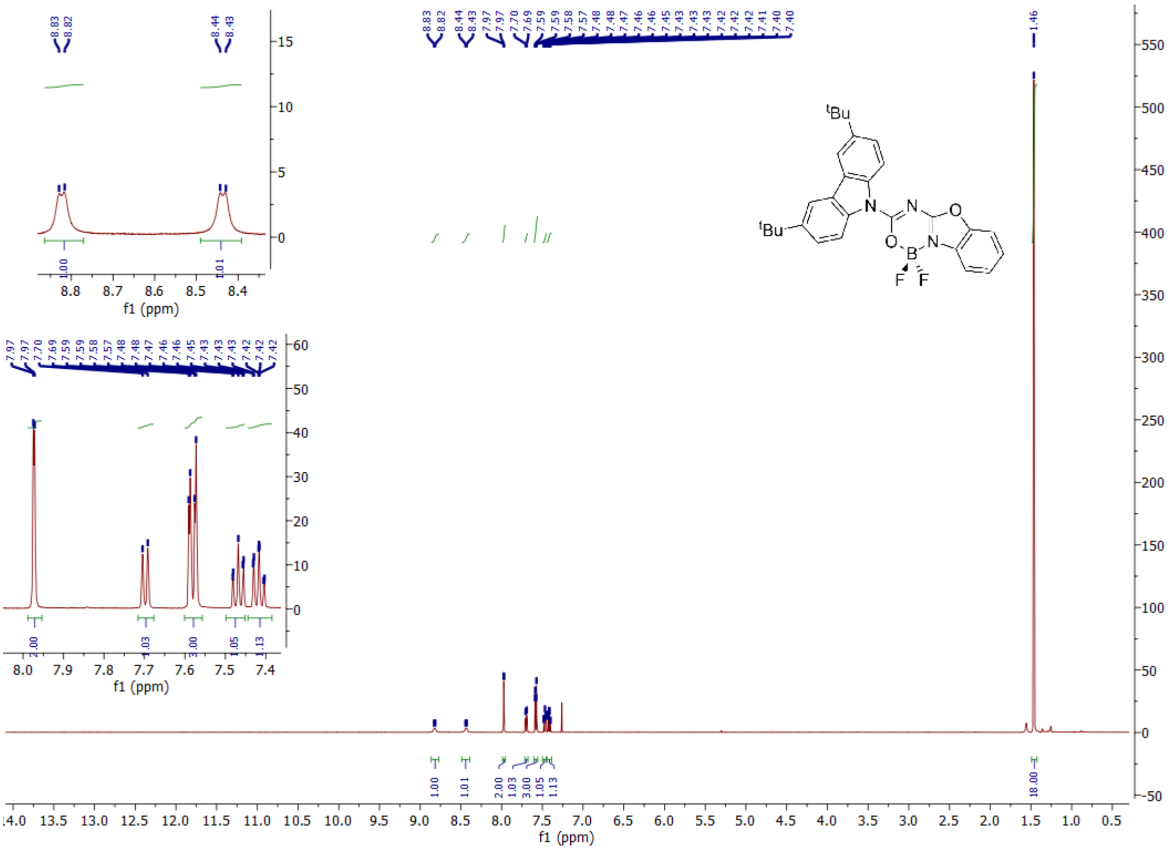

Figure S67. ^{1}H NMR (600 MHz, $CDCl_3$) spectrum of compound **1b**.

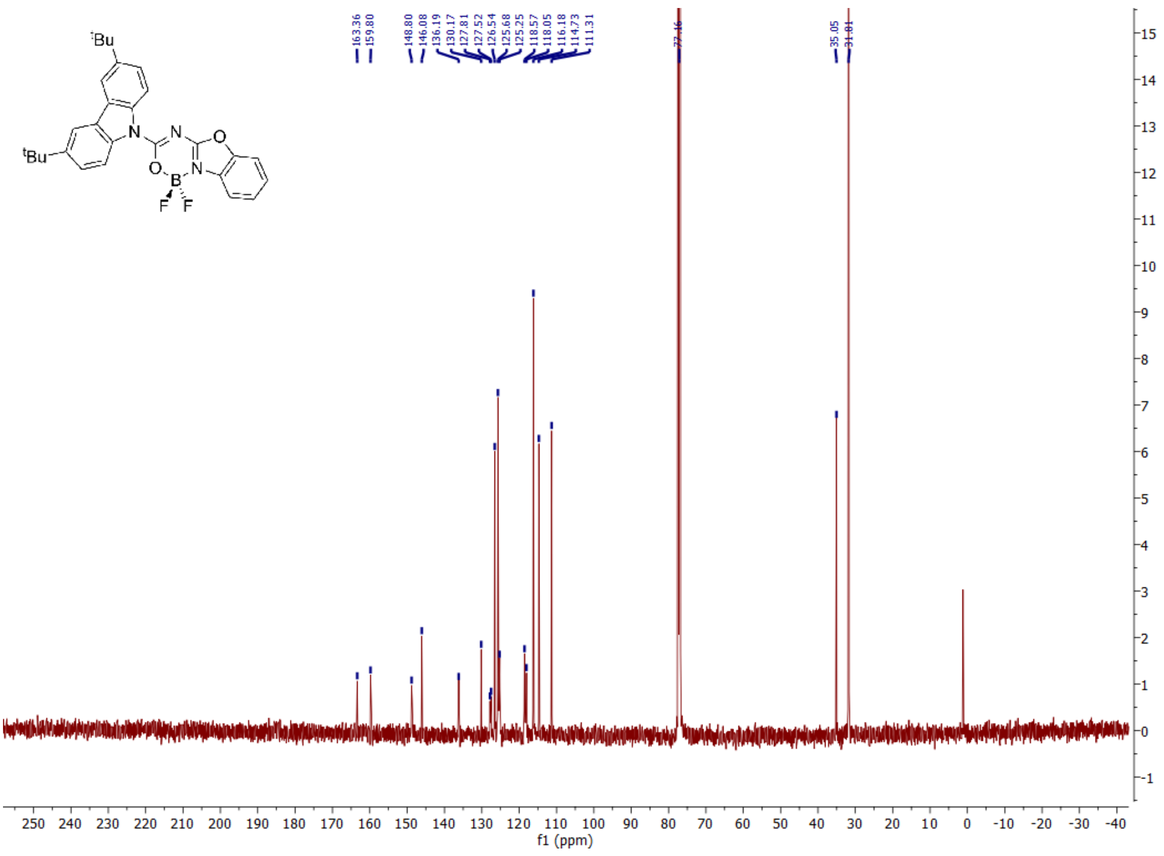

Figure S68. $^{13}C\{H\}$ NMR (150 MHz, $CDCl_3$) spectrum of compound **1b**.

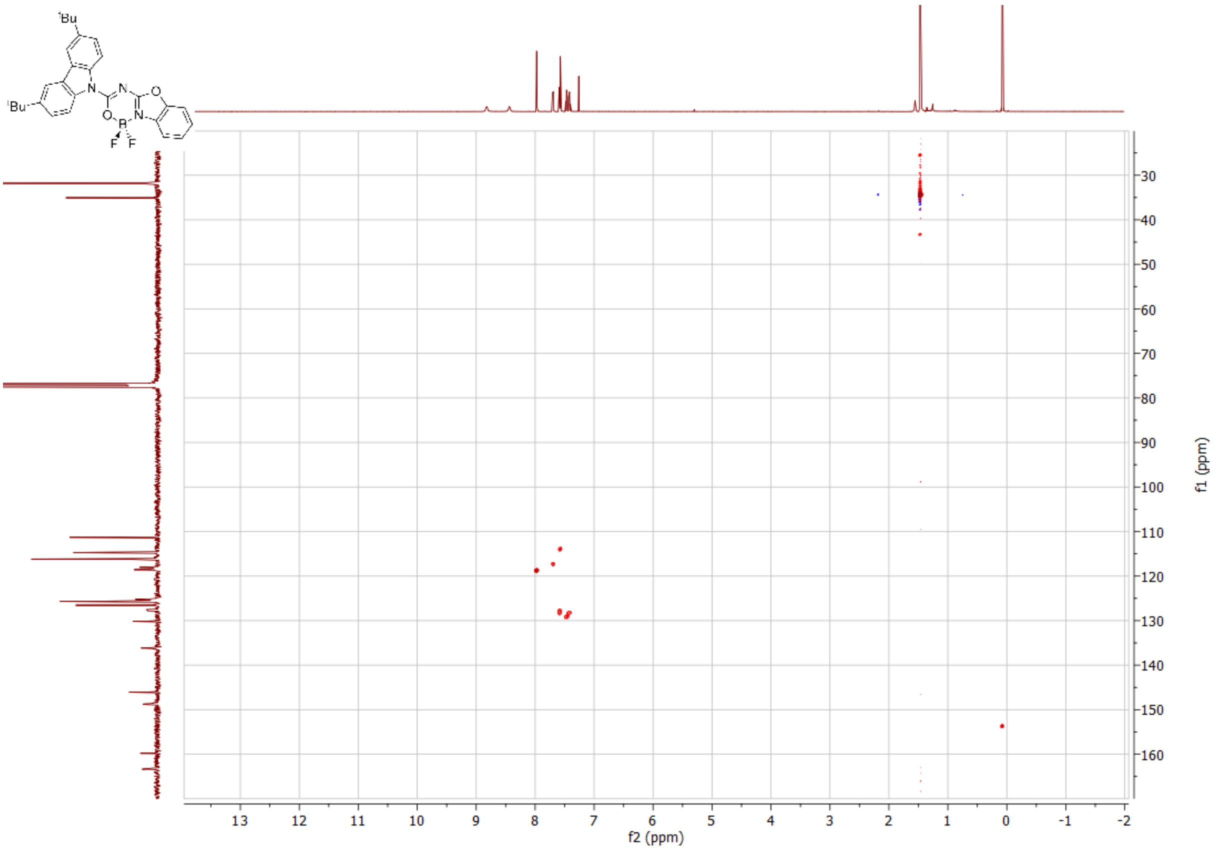

Figure S69. ^{1}H–^{13}C HSQC NMR (600 MHz, $CDCl_3$) spectrum of compound **1b**.

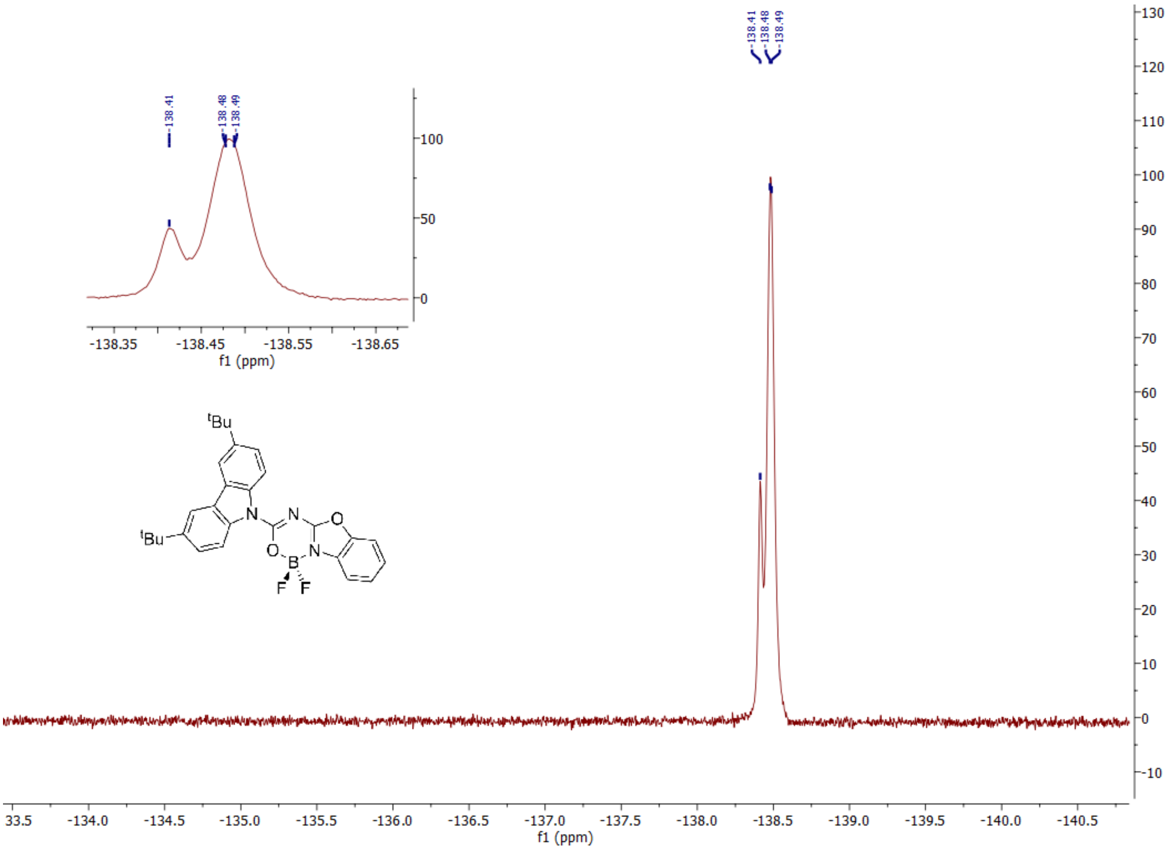

Figure S70. ^{19}F NMR (375 MHz, $CDCl_3$) spectrum of compound **1b**.

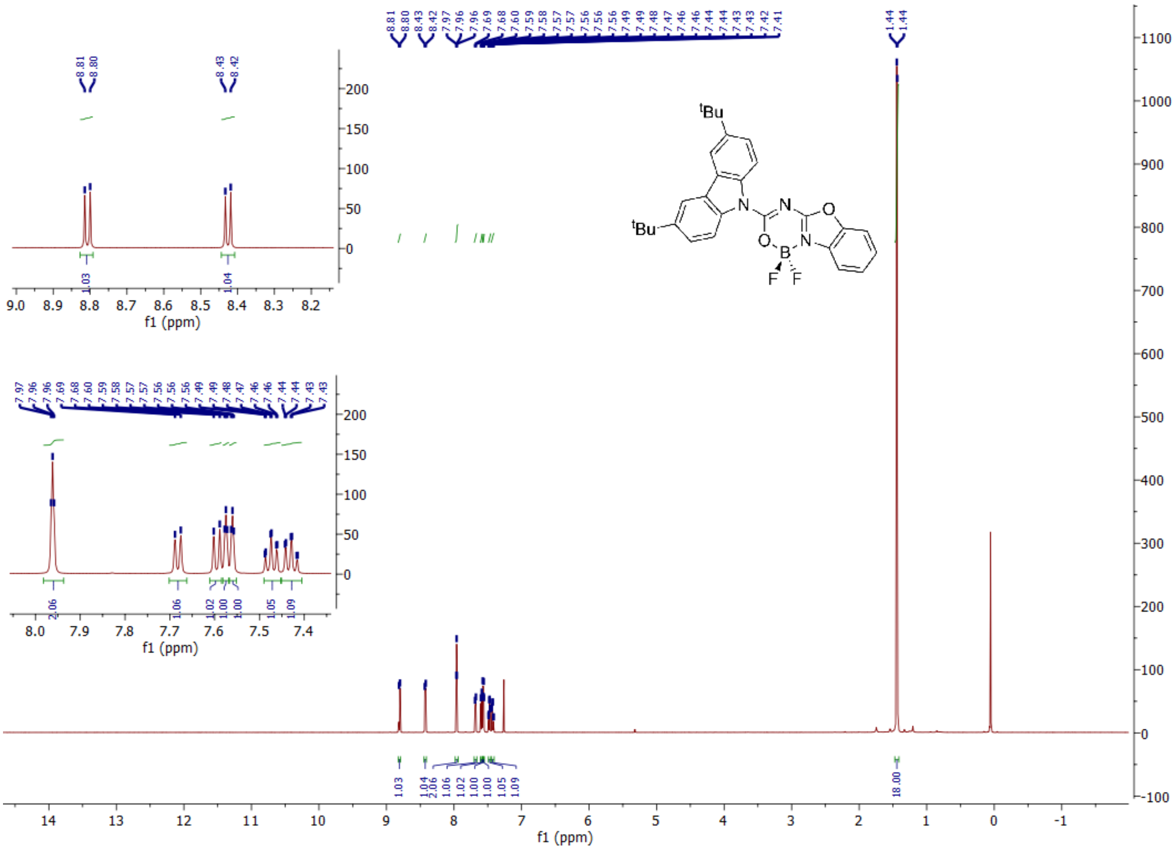

Figure S71. ^{1}H NMR (600 MHz, $CDCl_3$) spectrum of compound **1b** at -40°C.

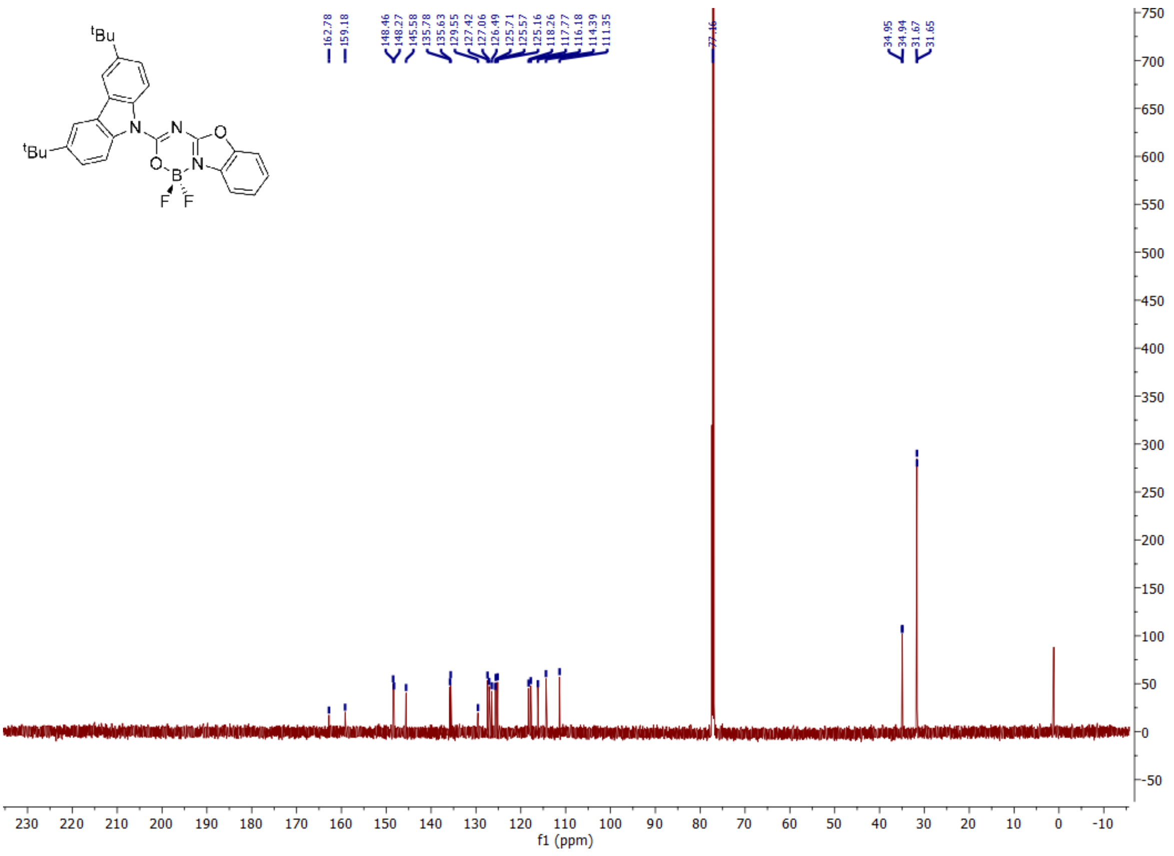

Figure S72. $^{13}C\{H\}$ NMR (150 MHz, $CDCl_3$) spectrum of compound **1b** at -40°C.

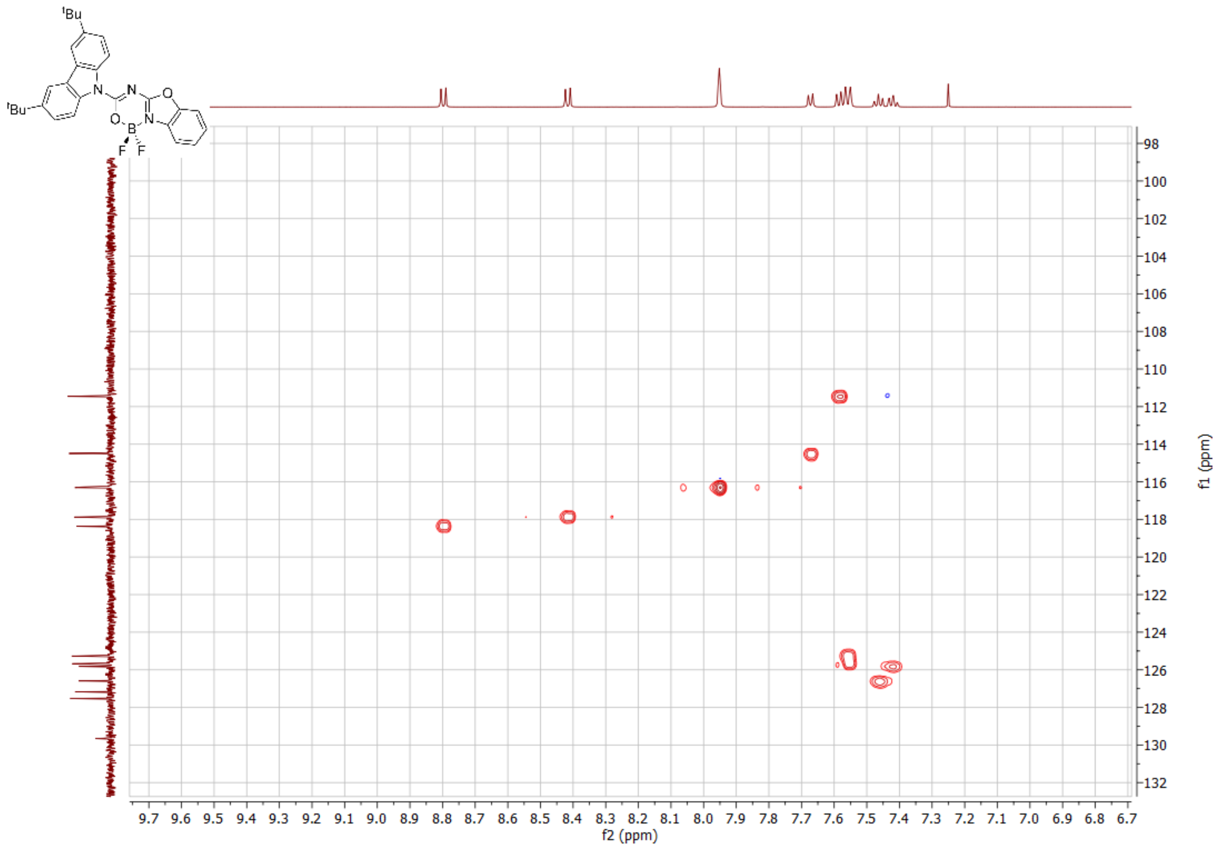

Figure S73. ^{1}H–^{13}C HSQC NMR (600 MHz, $CDCl_3$) spectrum of compound **1b** at -40°C.

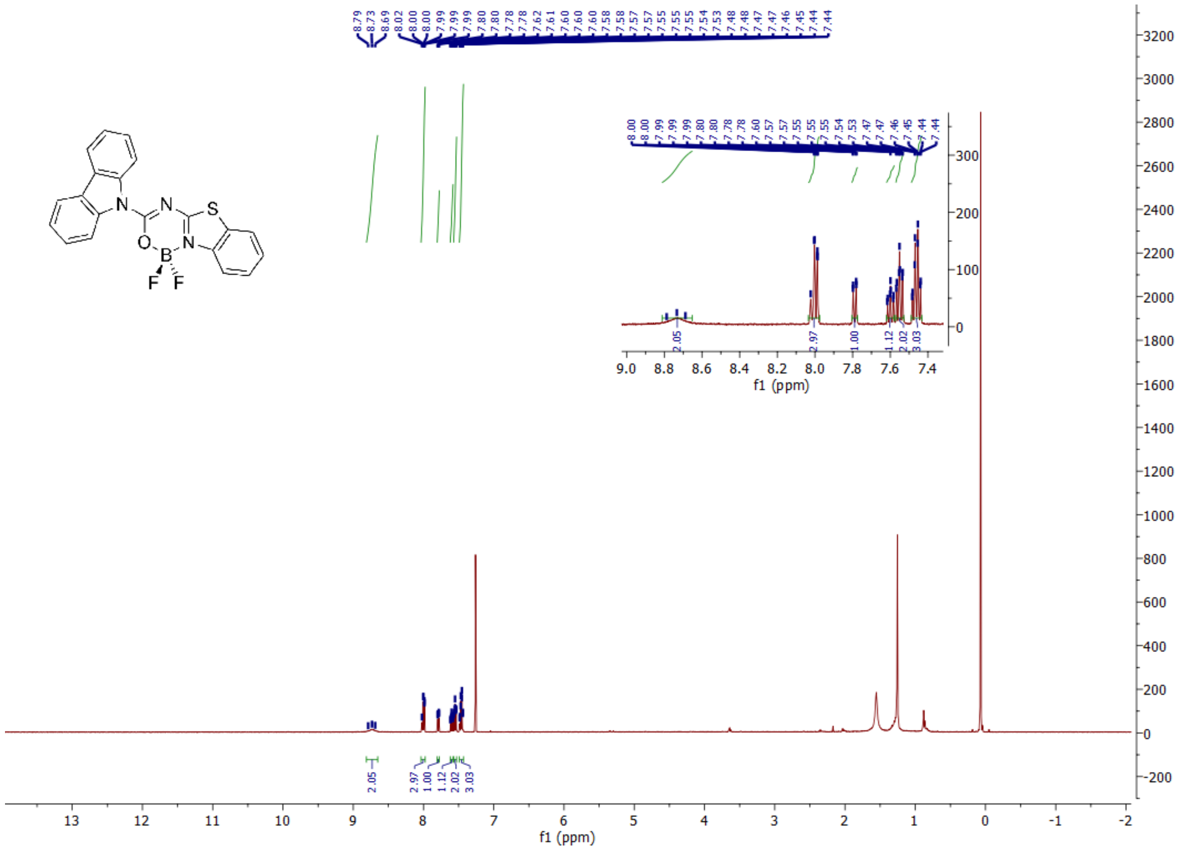

Figure S74. ^{1}H NMR (500 MHz, $CDCl_3$) spectrum of compound **2a**.

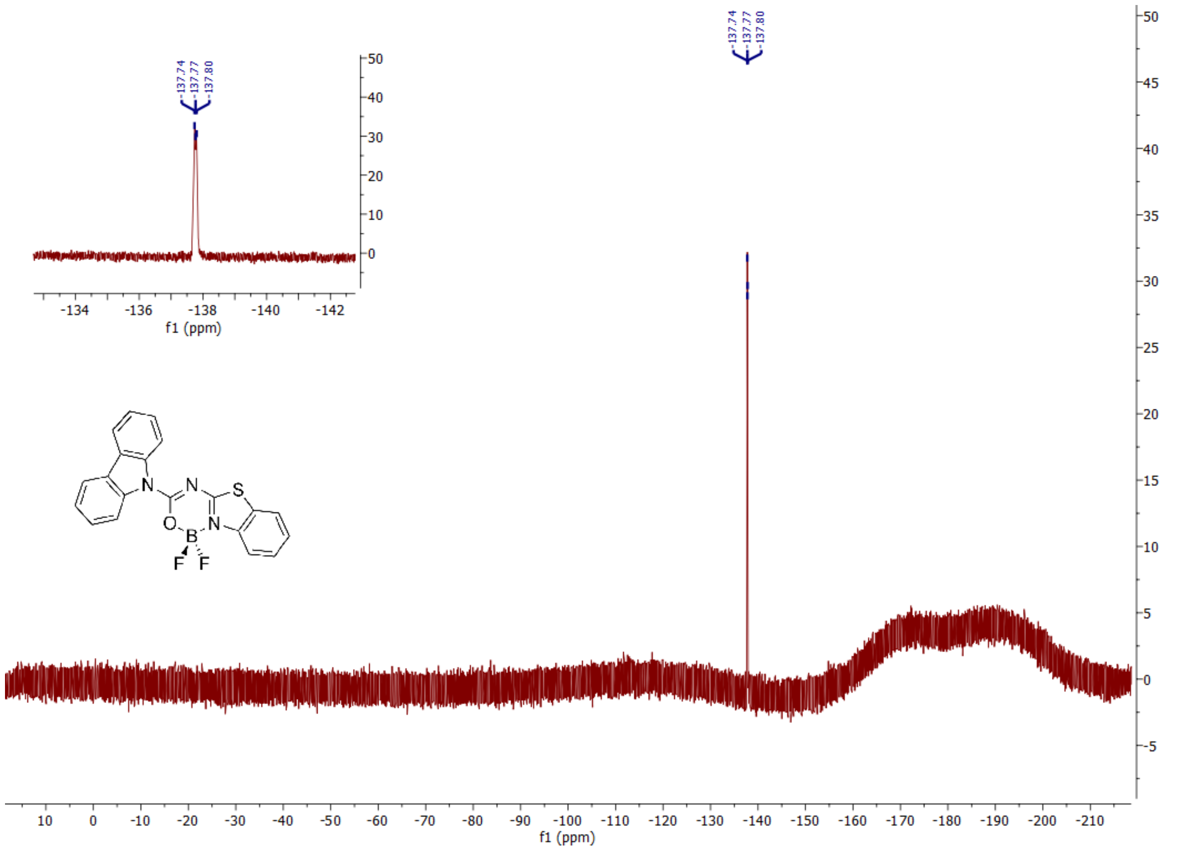

Figure S75. ^{19}F NMR (470 MHz, $CDCl_3$) spectrum of compound **2a**.

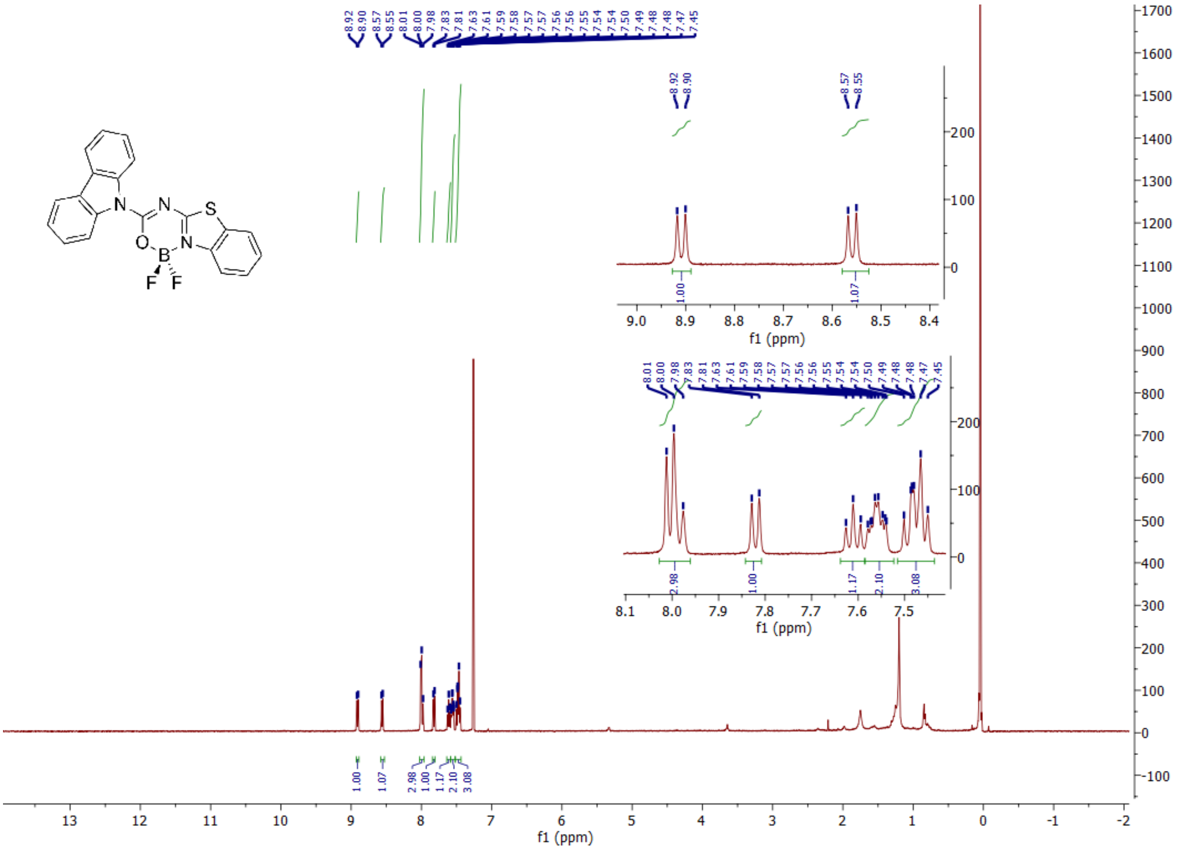

Figure S76. ^{1}H NMR (500 MHz, $CDCl_3$) spectrum of compound **2a** at -40°C.

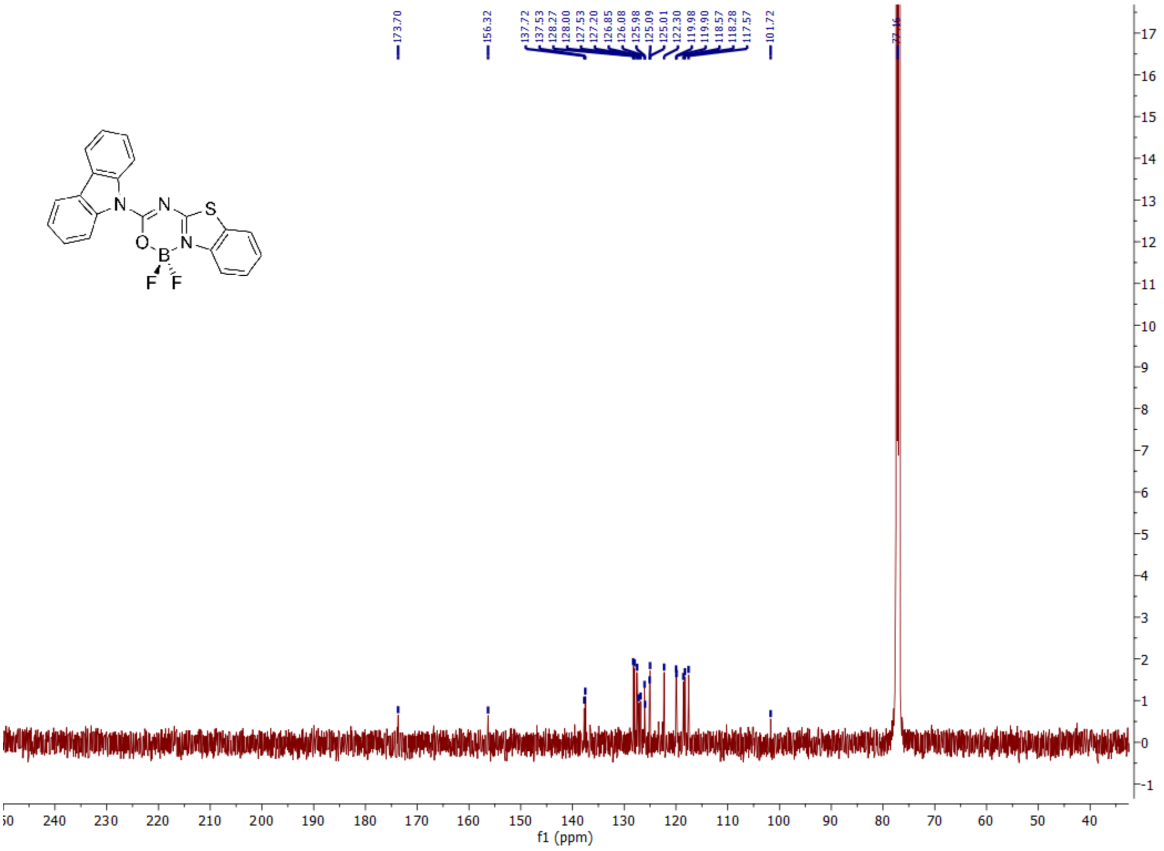

Figure S77. ^{13}C{H} NMR (125 MHz, $CDCl_3$) spectrum of compound **2a** at -40°C.

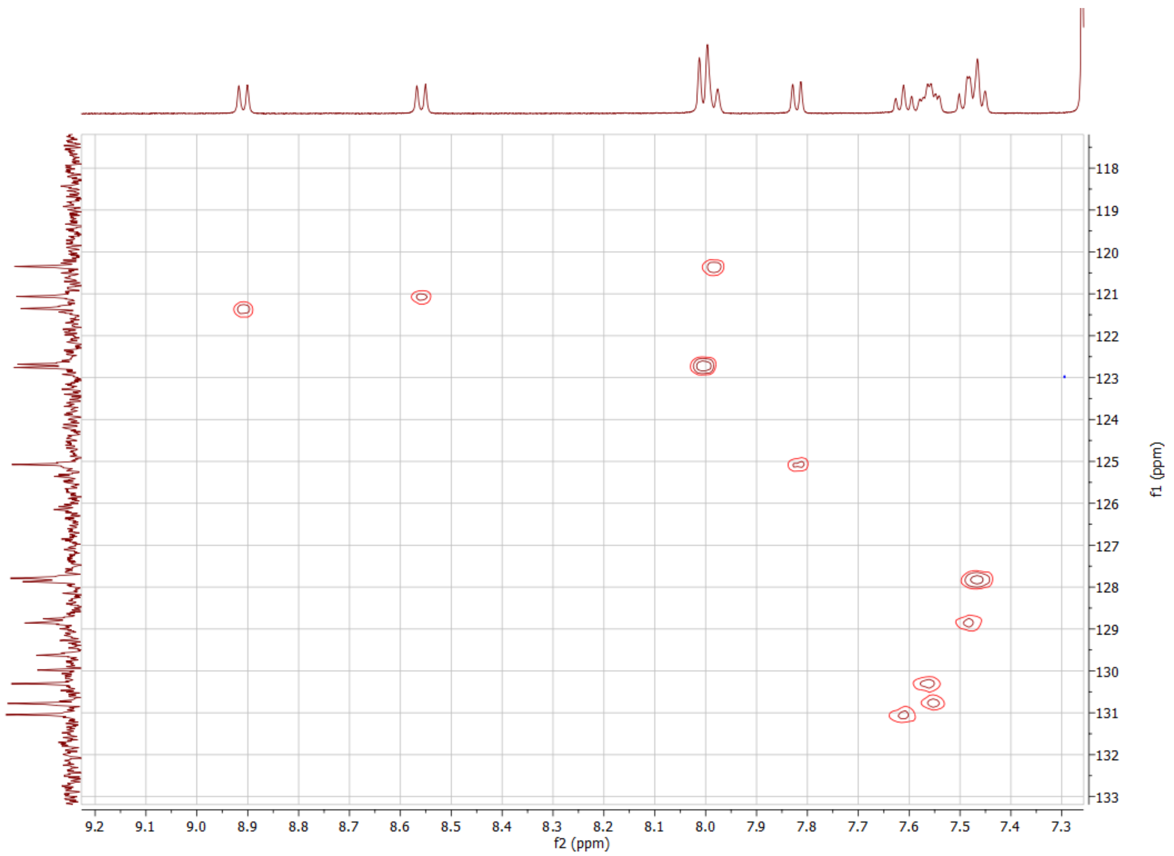

Figure S78. ^{1}H–^{13}C HSQC NMR (125 MHz, $CDCl_3$) spectrum of compound **2a** at -40°C.

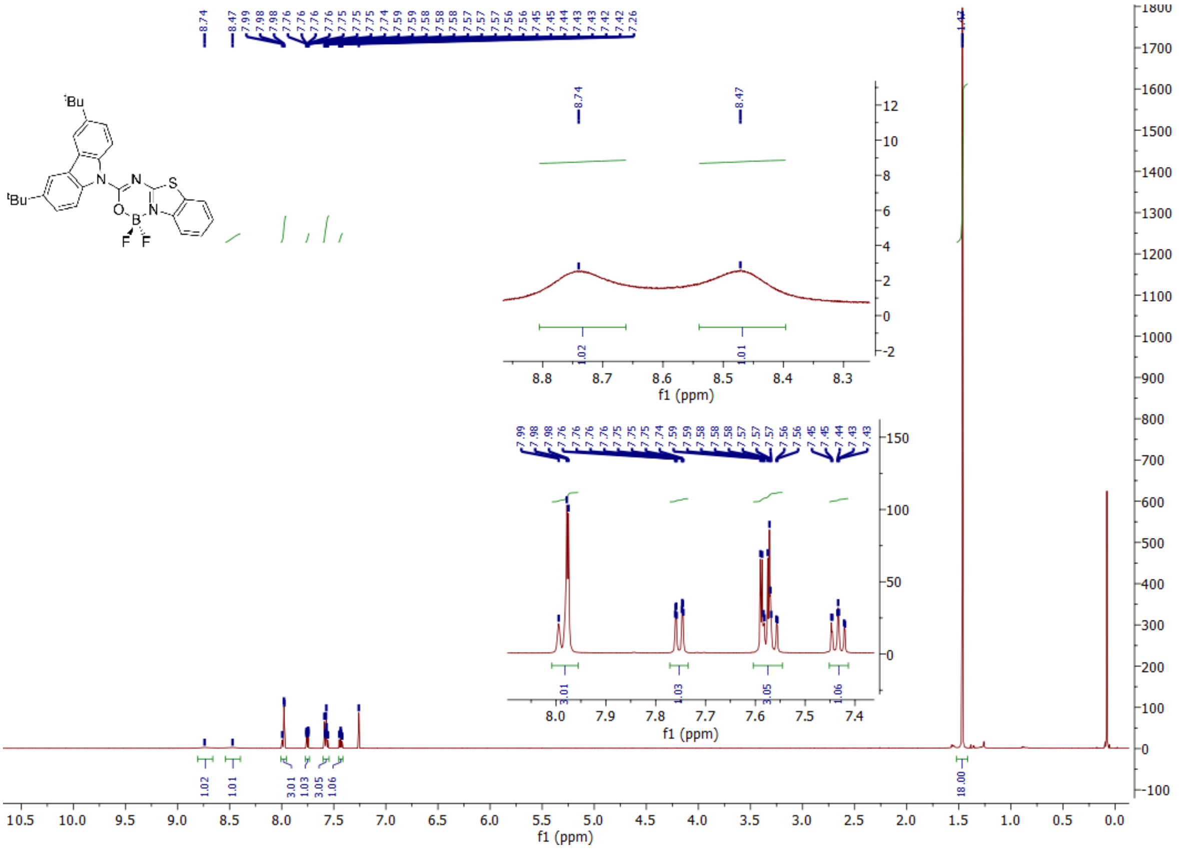

Figure S79. ^{1}H NMR (600 MHz, $CDCl_3$) spectrum of compound **2b**.

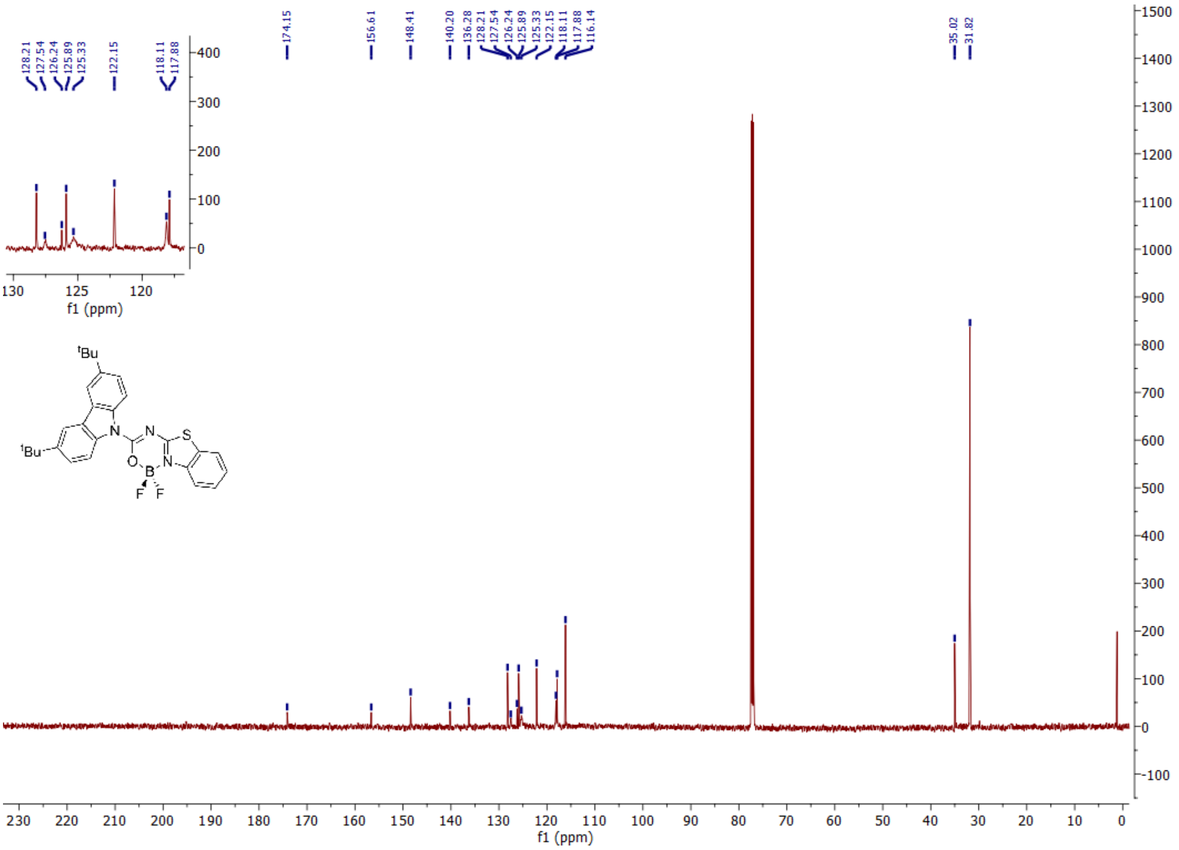

Figure S80. $^{13}C\{H\}$ NMR (150 MHz, $CDCl_3$) spectrum of compound **2b**.

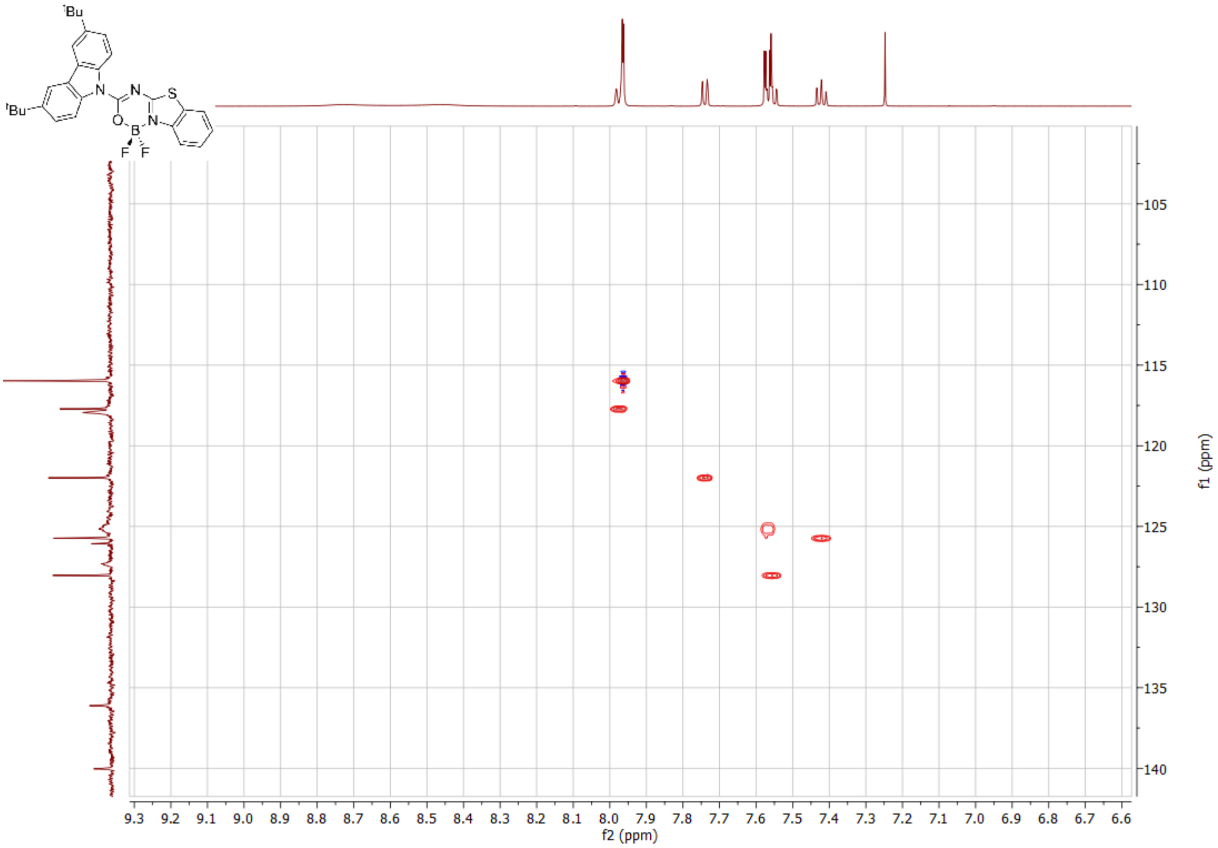

Figure S81. ^{1}H–^{13}C HSQC NMR (600 MHz, $CDCl_3$) spectrum of compound **2b**.

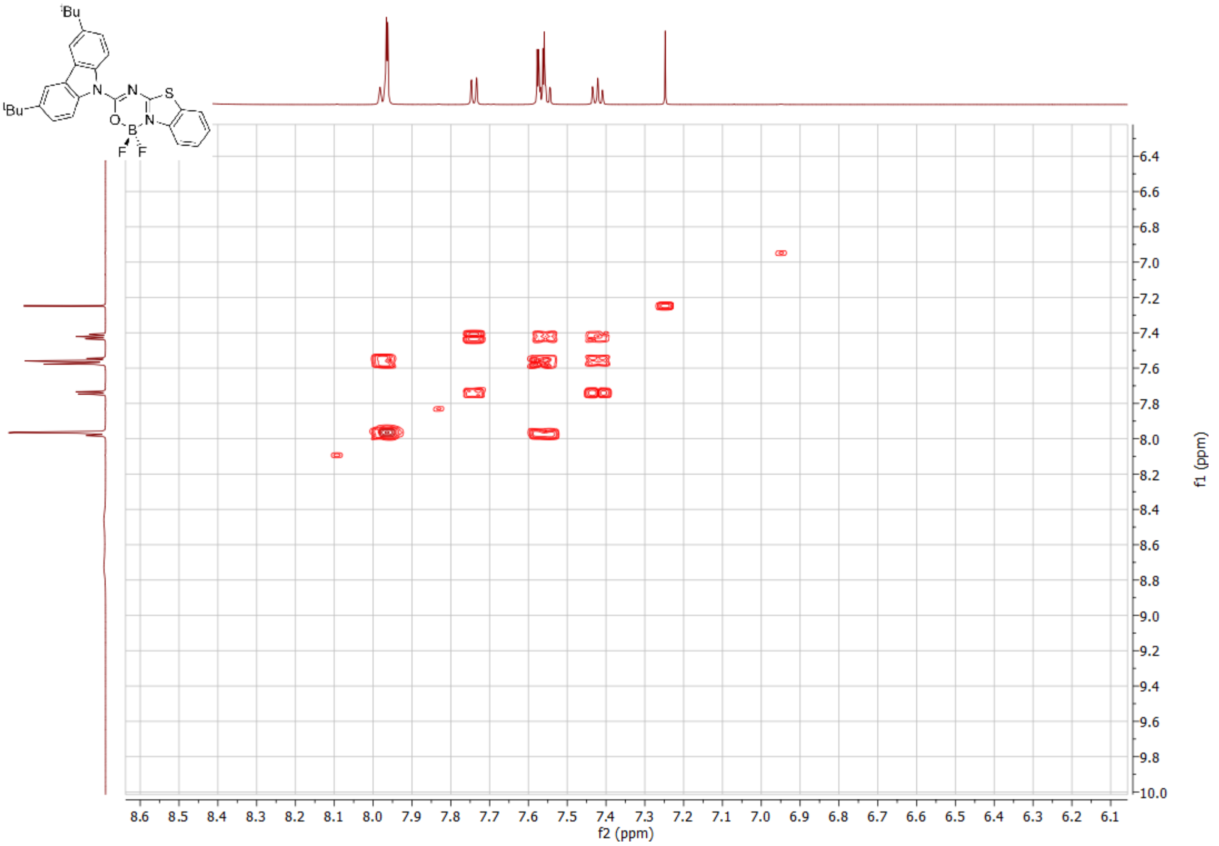

Figure S82. ^{1}H–^{1}H COSY NMR (600 MHz, CDCl$_3$) spectrum of compound **2b**.

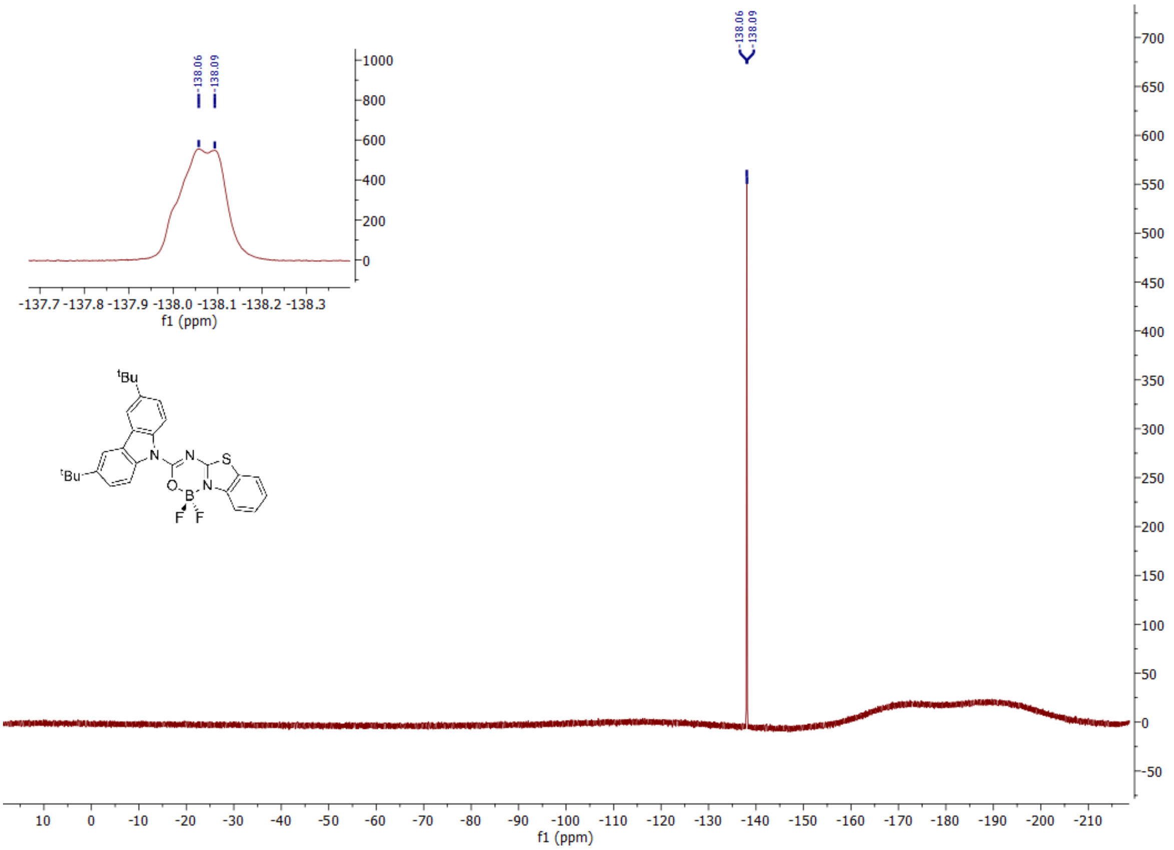

Figure S83. ^{19}F NMR (375 MHz, CDCl$_3$) spectrum of compound **2b**.

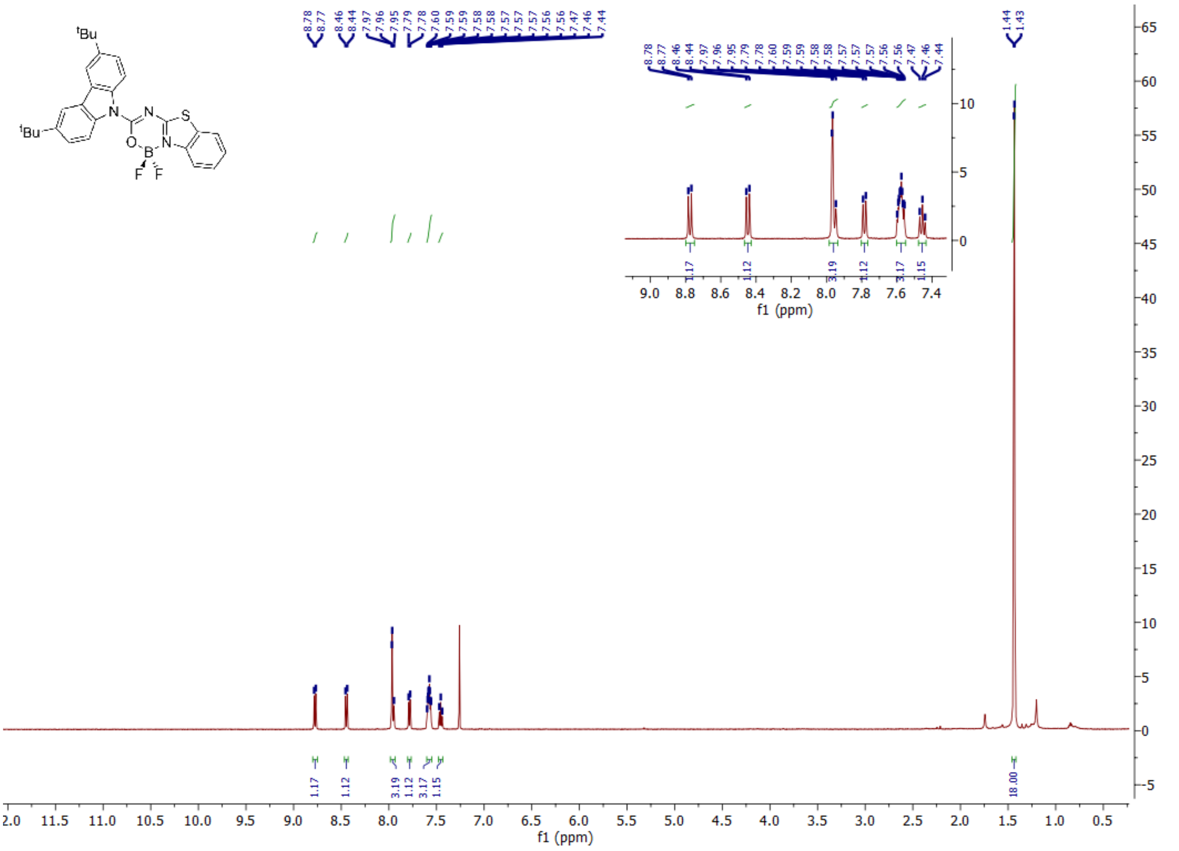

Figure S84. ^{1}H NMR (500 MHz, $CDCl_3$) spectrum of compound **2b** at -40°C.

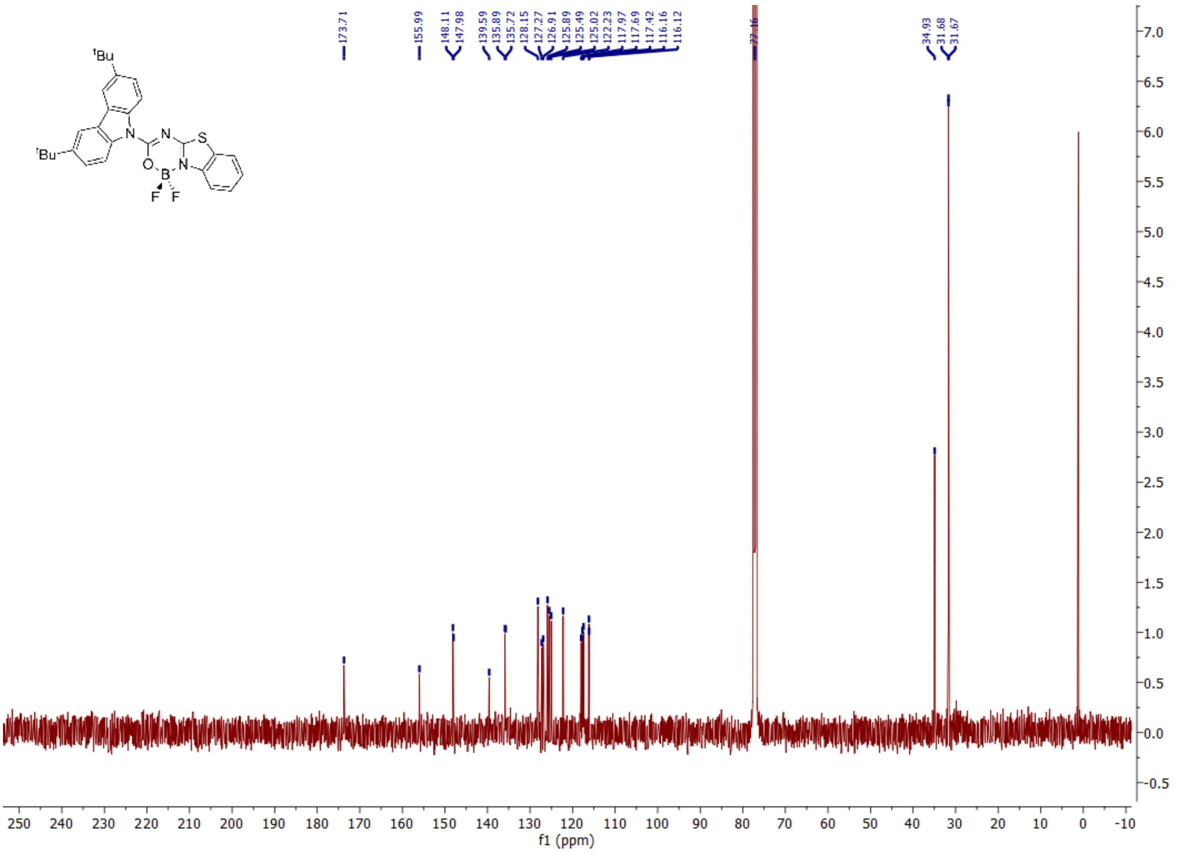

Figure S85. $^{13}C\{H\}$ NMR (125 MHz, $CDCl_3$) spectrum of compound **2b** at -40°C.

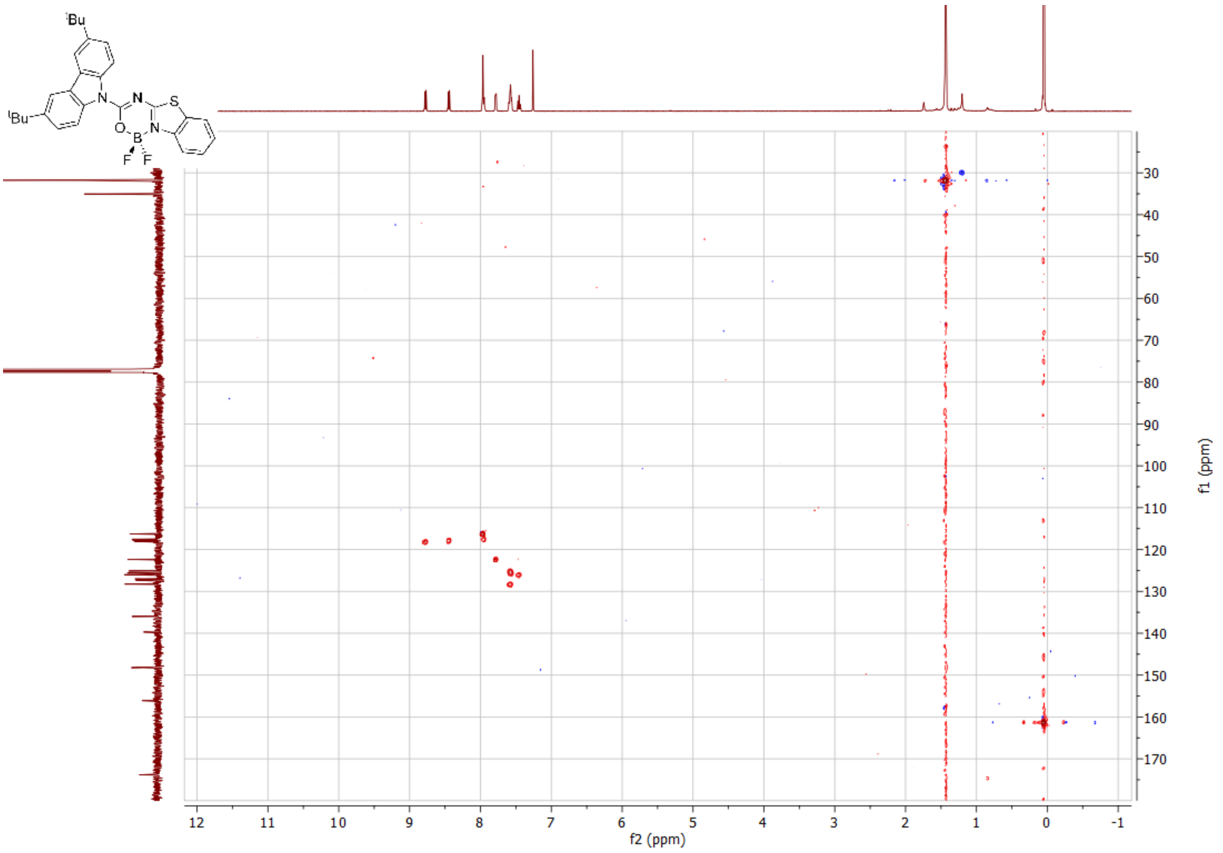

Figure S86. $^{1}H–^{13}C$ HSQC NMR (500 MHz, $CDCl_3$) spectrum of compound **2b** at -40°C.

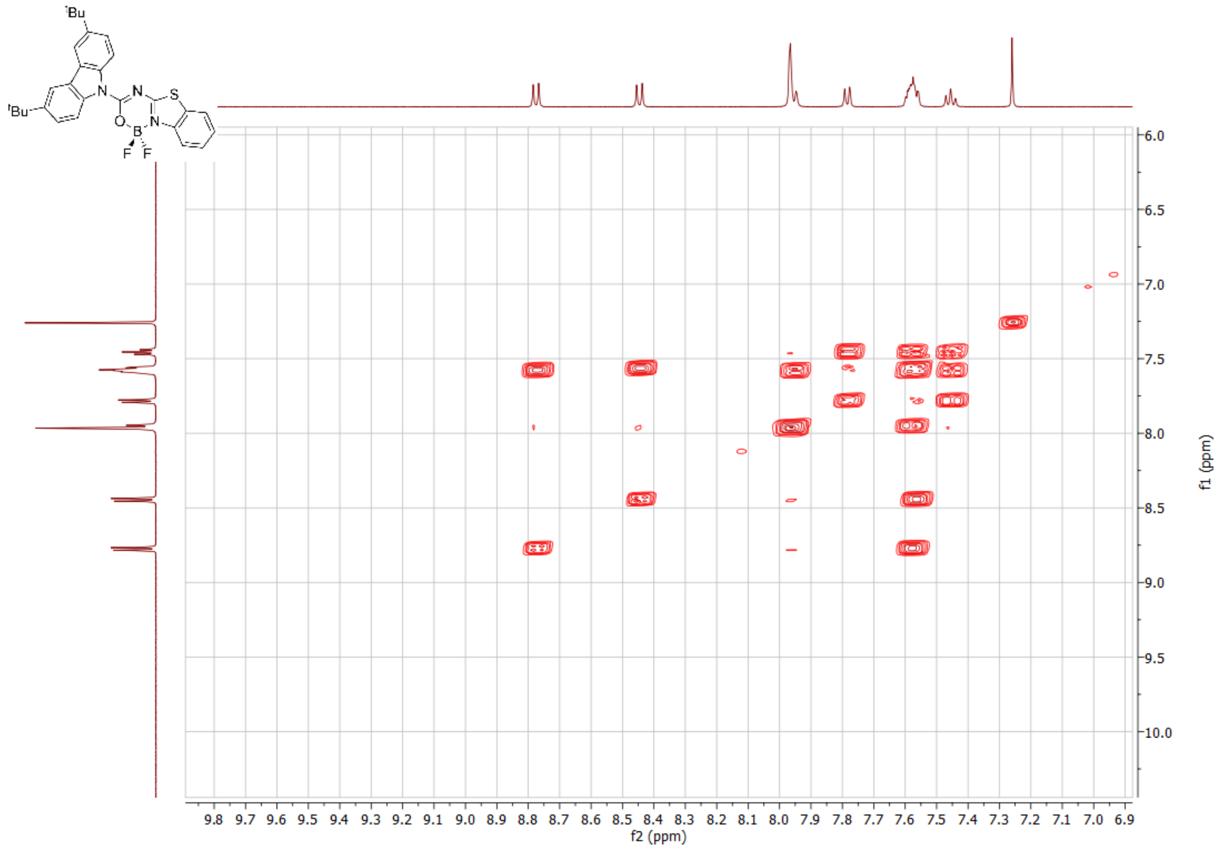

Figure S87. $^{1}H–^{1}H$ COSY NMR (500 MHz, $CDCl_3$) spectrum of compound **2b** at -40°C.

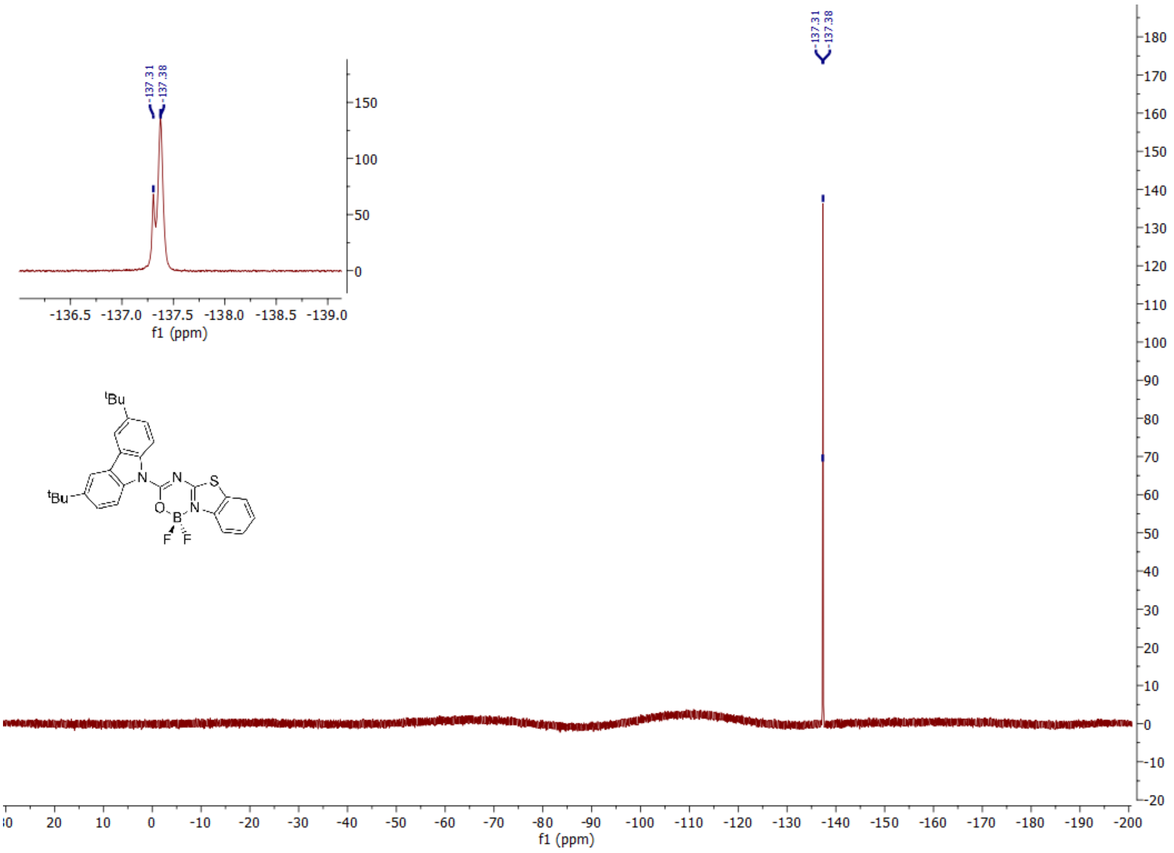

Figure S88. ^{19}F NMR (470 MHz, $CDCl_3$) spectrum of compound **2b** at -40°C.

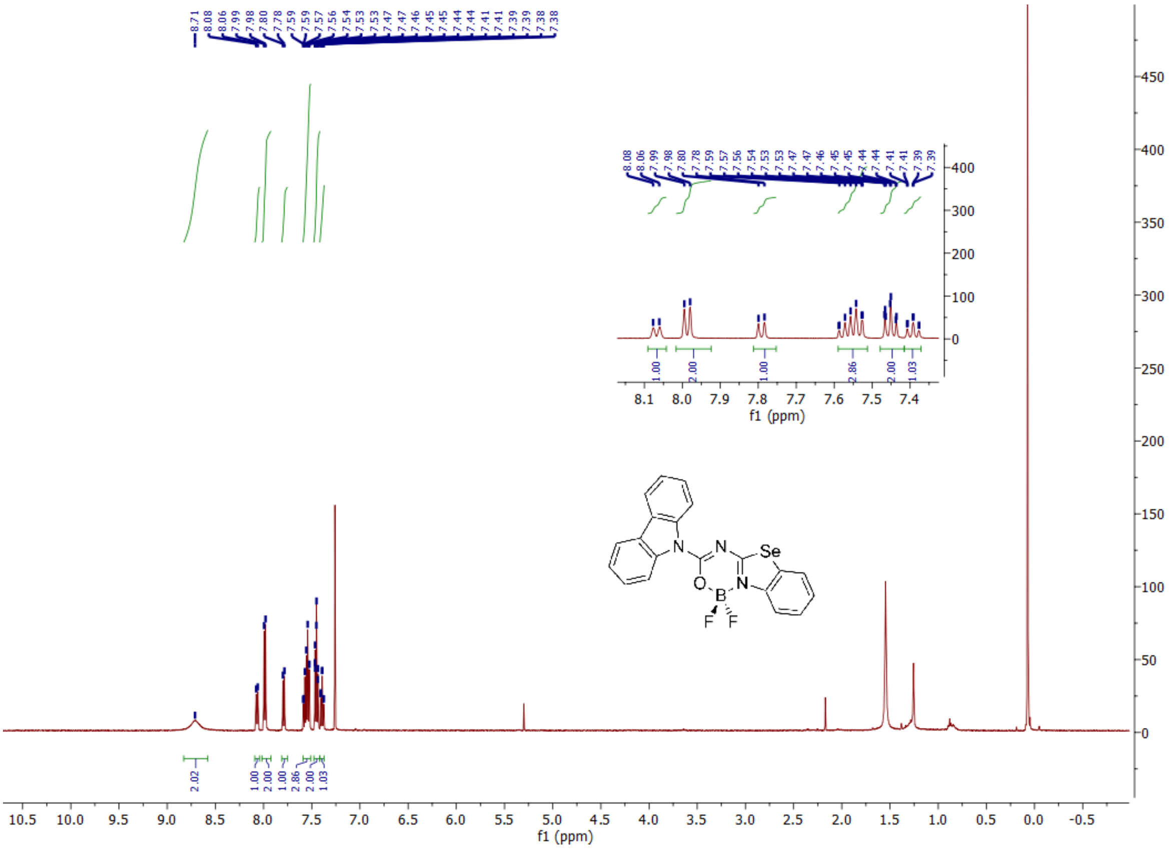

Figure S89. ^{1}H NMR (600 MHz, $CDCl_3$) spectrum of compound **3a**.

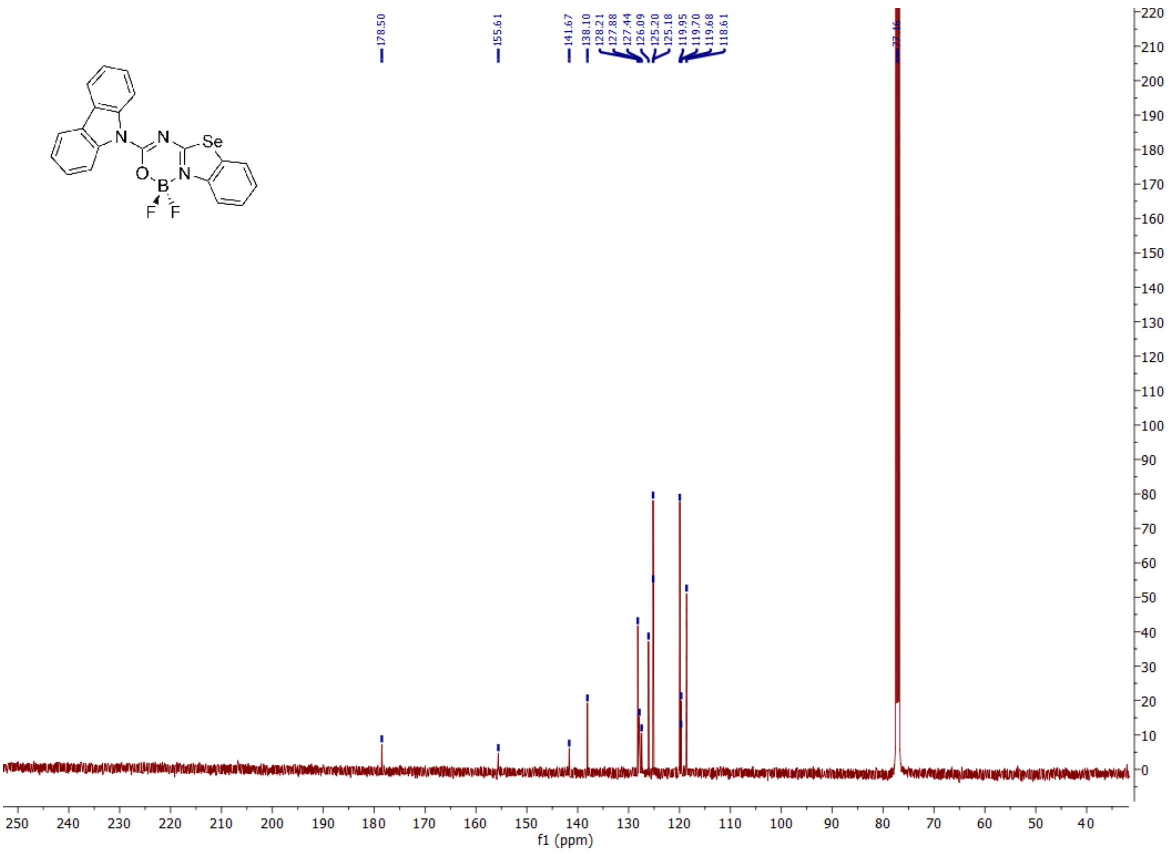

Figure S90. $^{13}C\{H\}$ NMR (150 MHz, $CDCl_3$) spectrum of compound **3a**.

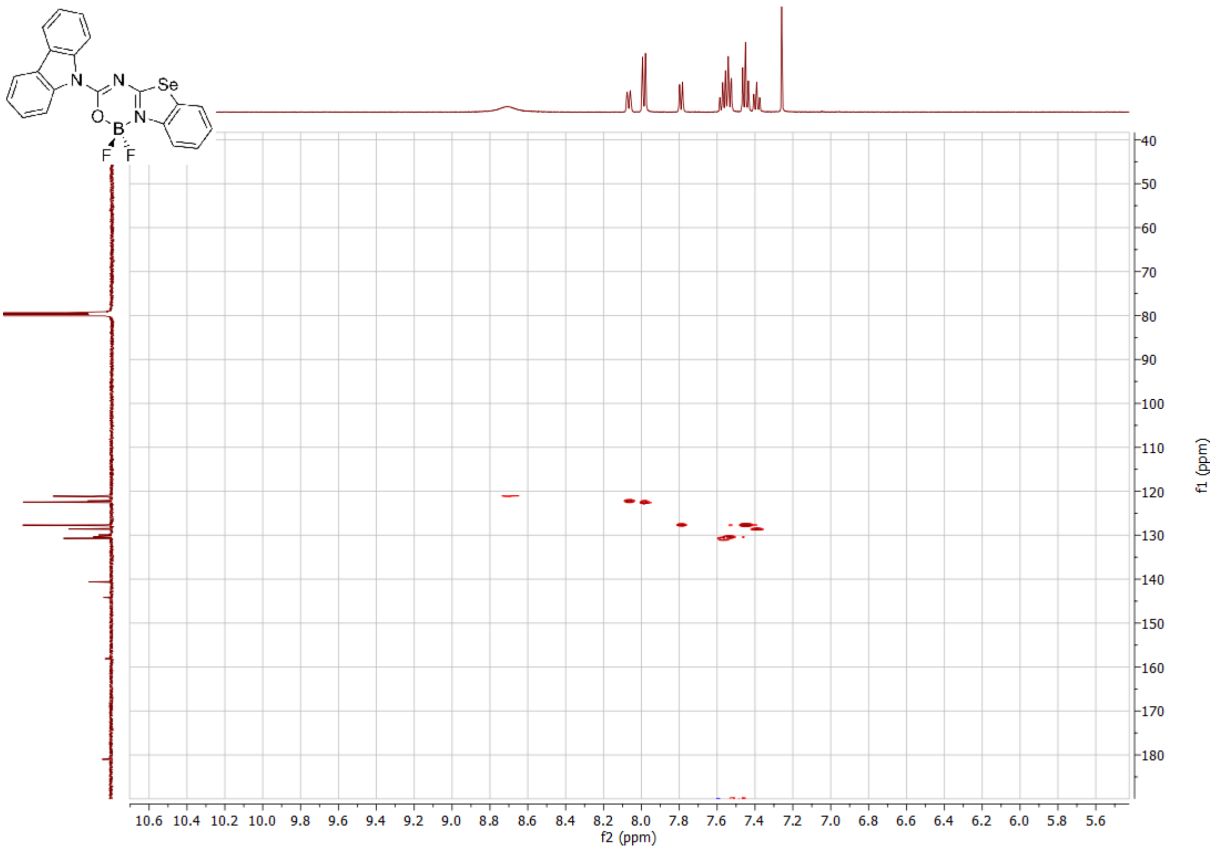

Figure S91. ^{1}H–^{13}C HSQC NMR (600 MHz, $CDCl_3$) spectrum of compound **3a**.

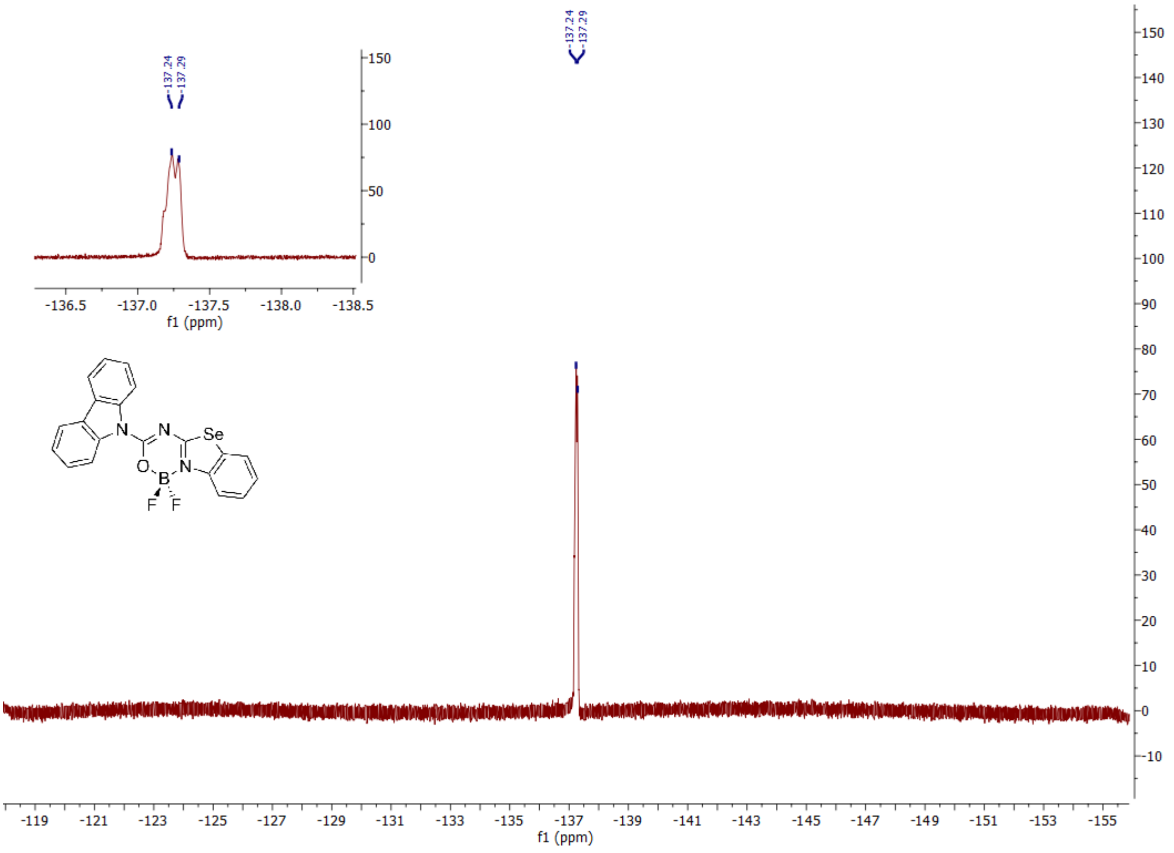

Figure S92. ^{19}F NMR (470 MHz, $CDCl_3$) spectrum of compound **3a**.

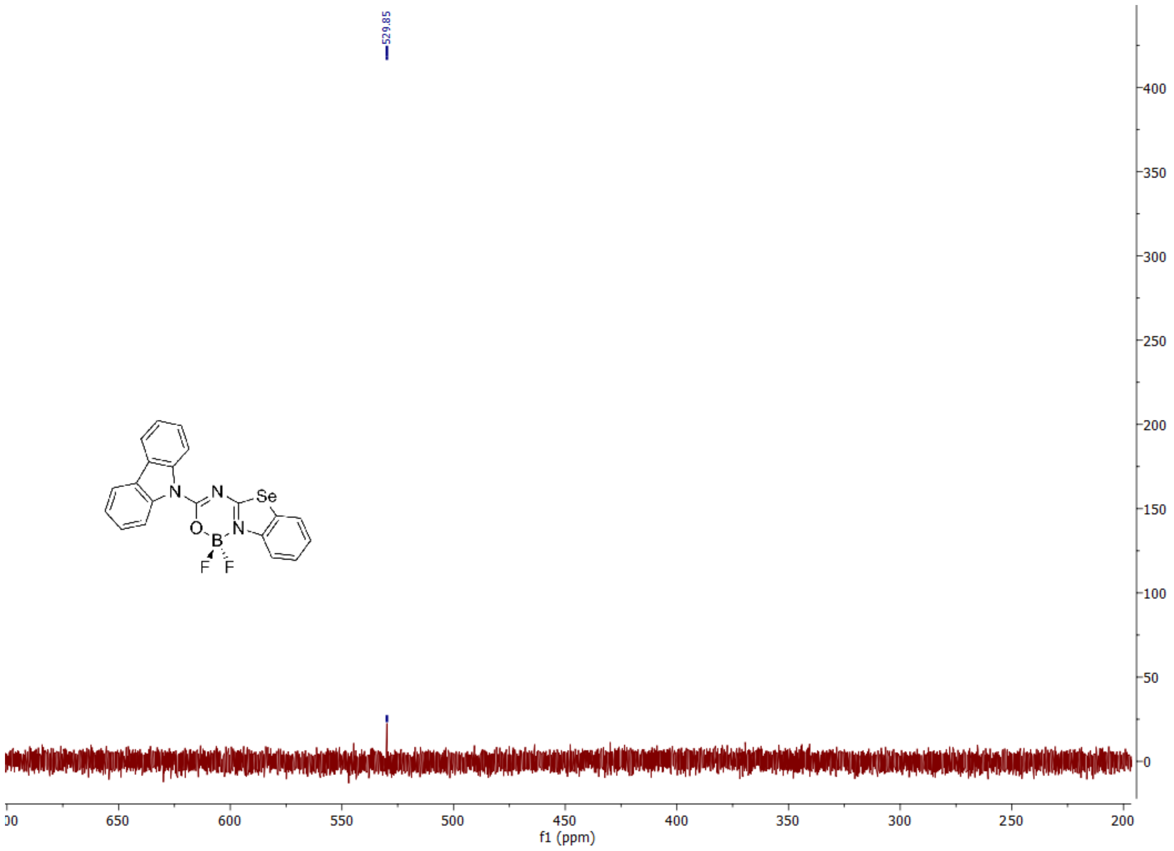

Figure S93. ^{77}Se NMR (95 MHz, $CDCl_3$) spectrum of compound **3a**.

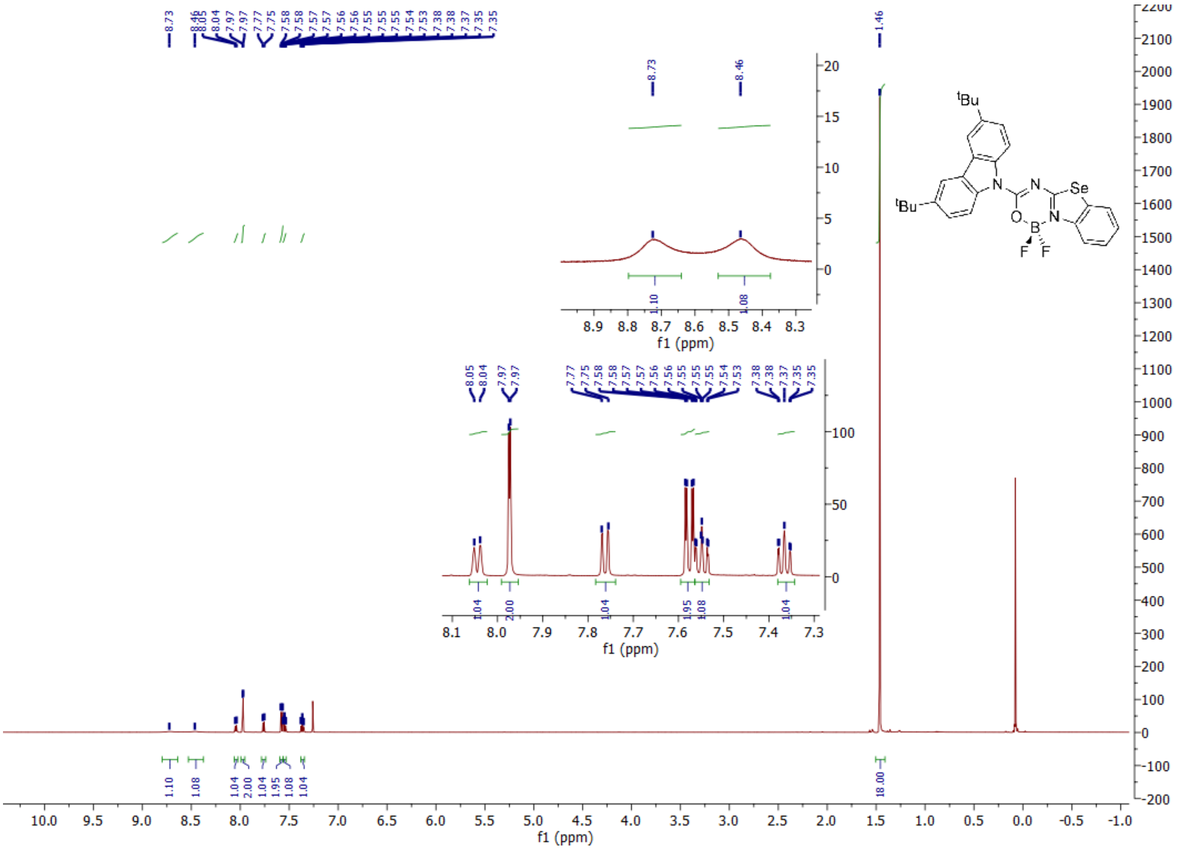

Figure S94. ^{1}H NMR (600 MHz, $CDCl_3$) spectrum of compound **3b**.

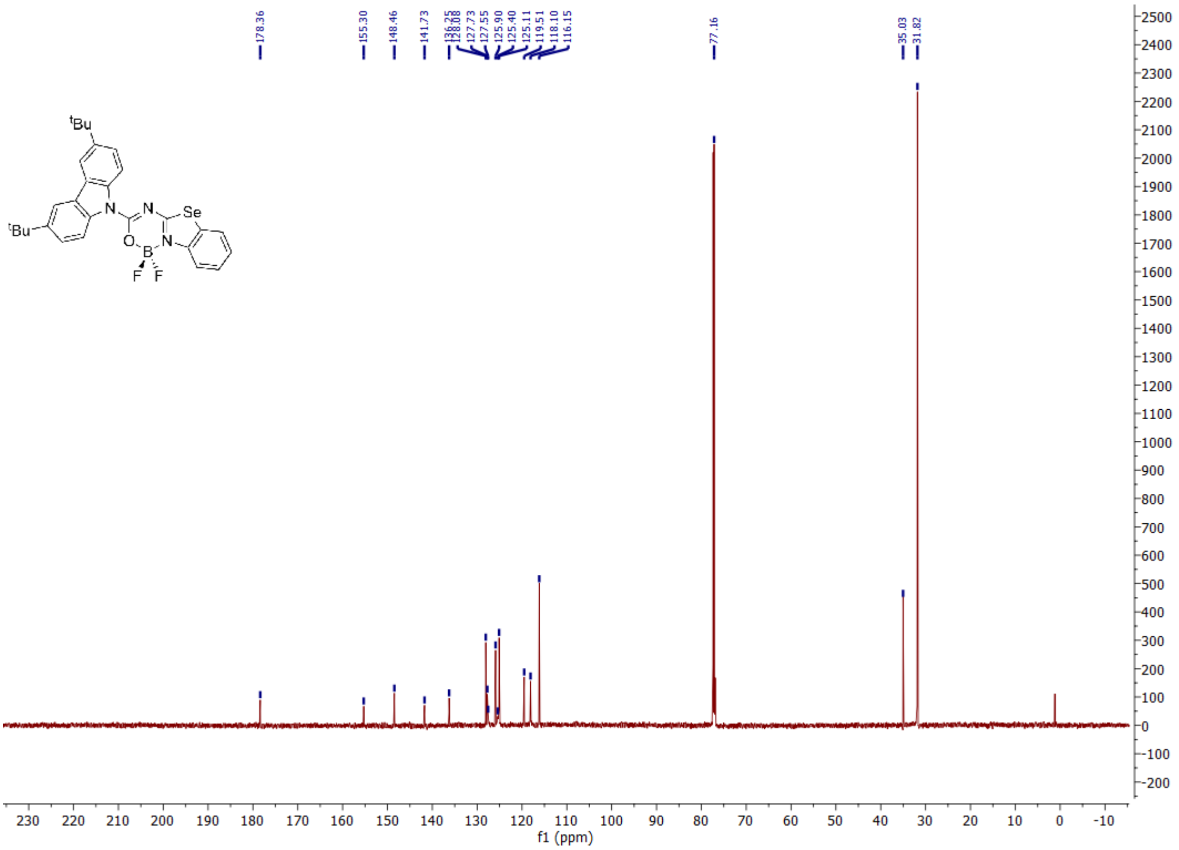

Figure S95. $^{13}C\{H\}$ NMR (150 MHz, $CDCl_3$) spectrum of compound **3b**.

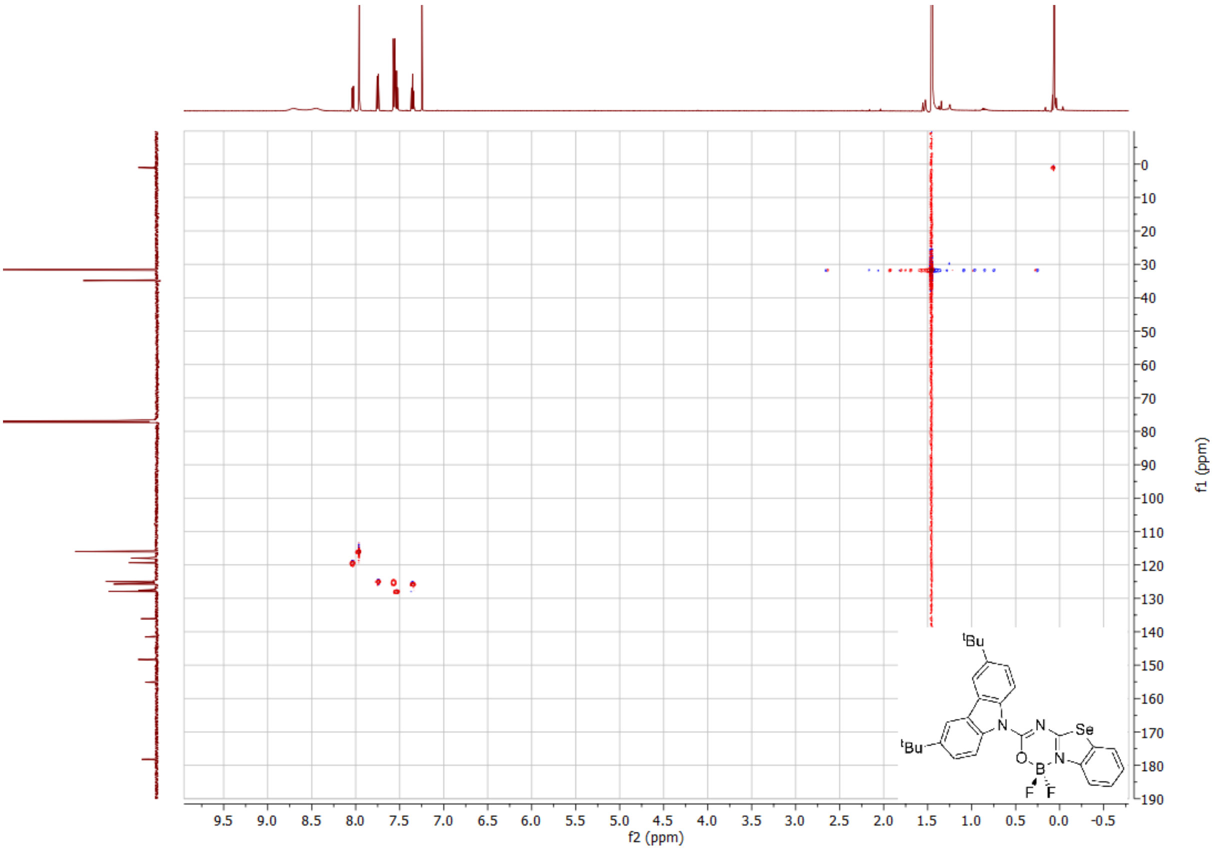

Figure S96. ^{1}H–^{13}C HSQC NMR (600 MHz, $CDCl_3$) spectrum of compound **3b**.

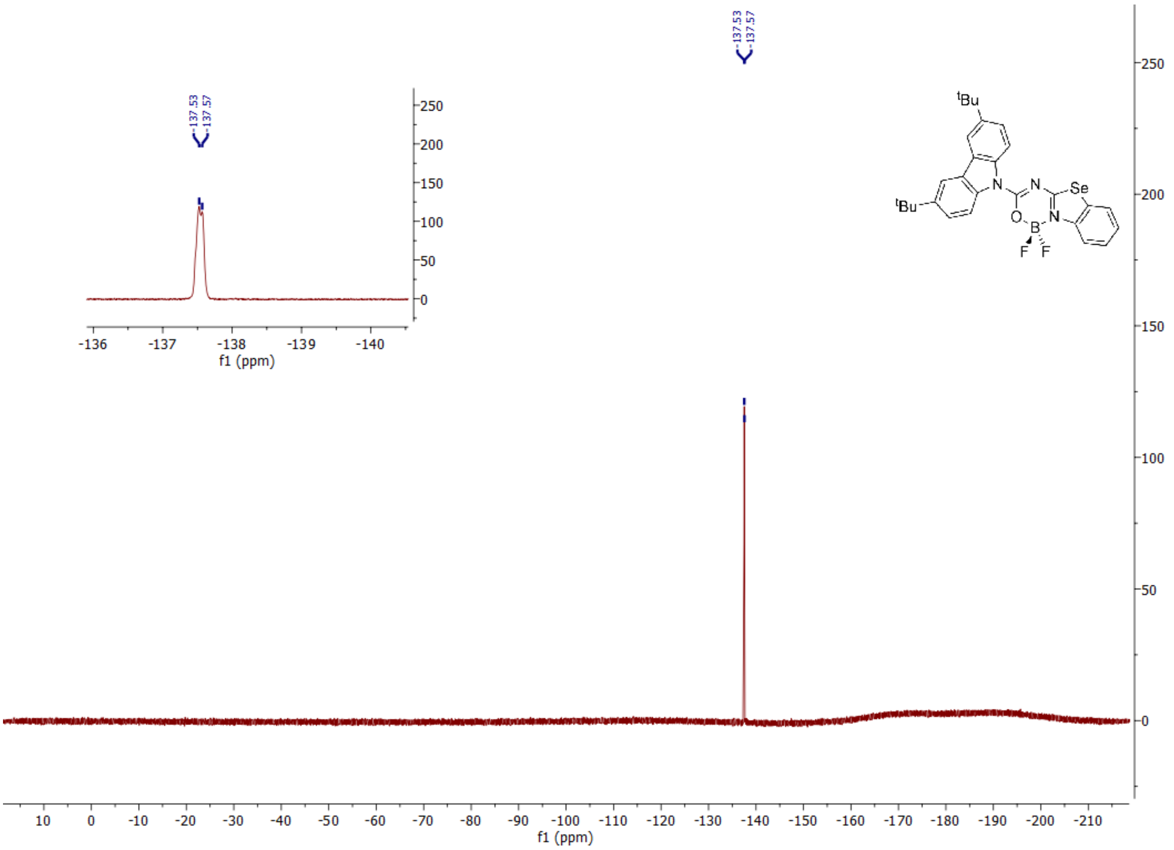

Figure S97. ^{19}F NMR (375 MHz, $CDCl_3$) spectrum of compound **3b**.

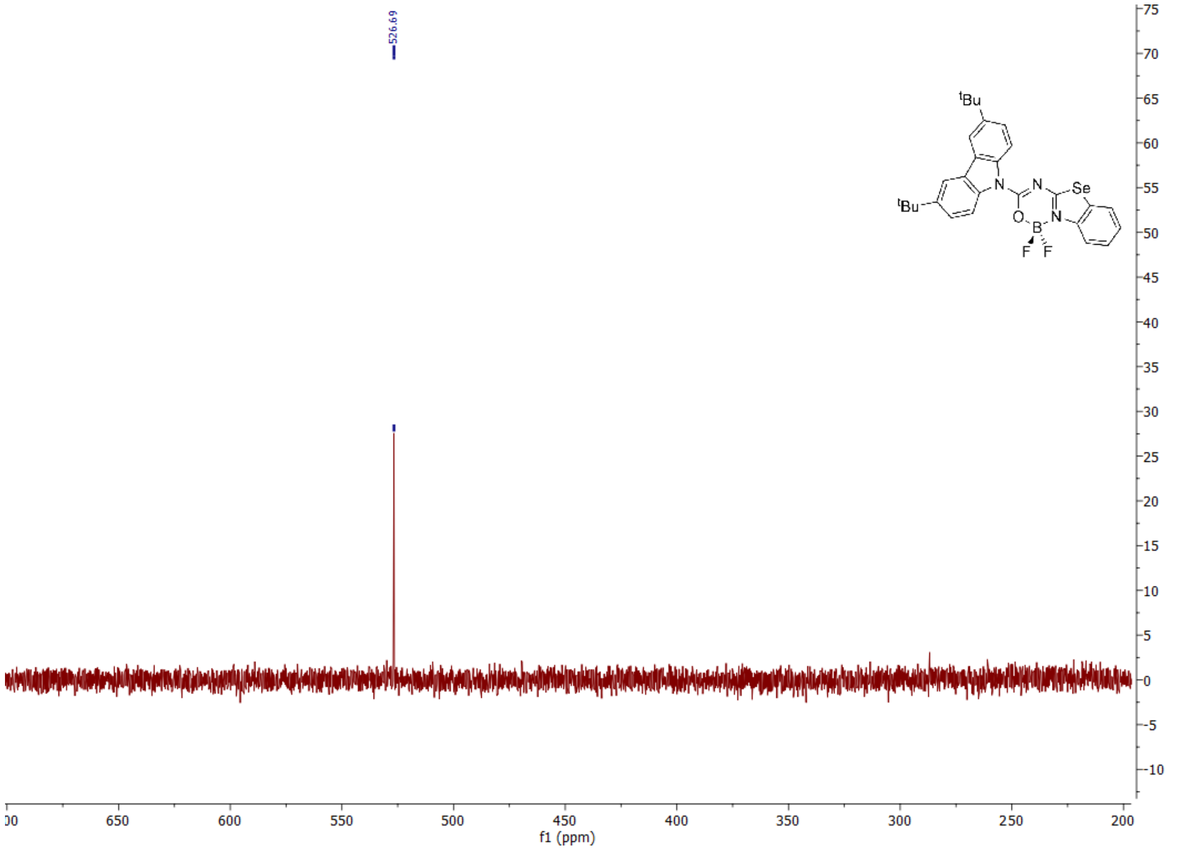

Figure S98. ^{77}Se NMR (95 MHz, $CDCl_3$) spectrum of compound **3b**.

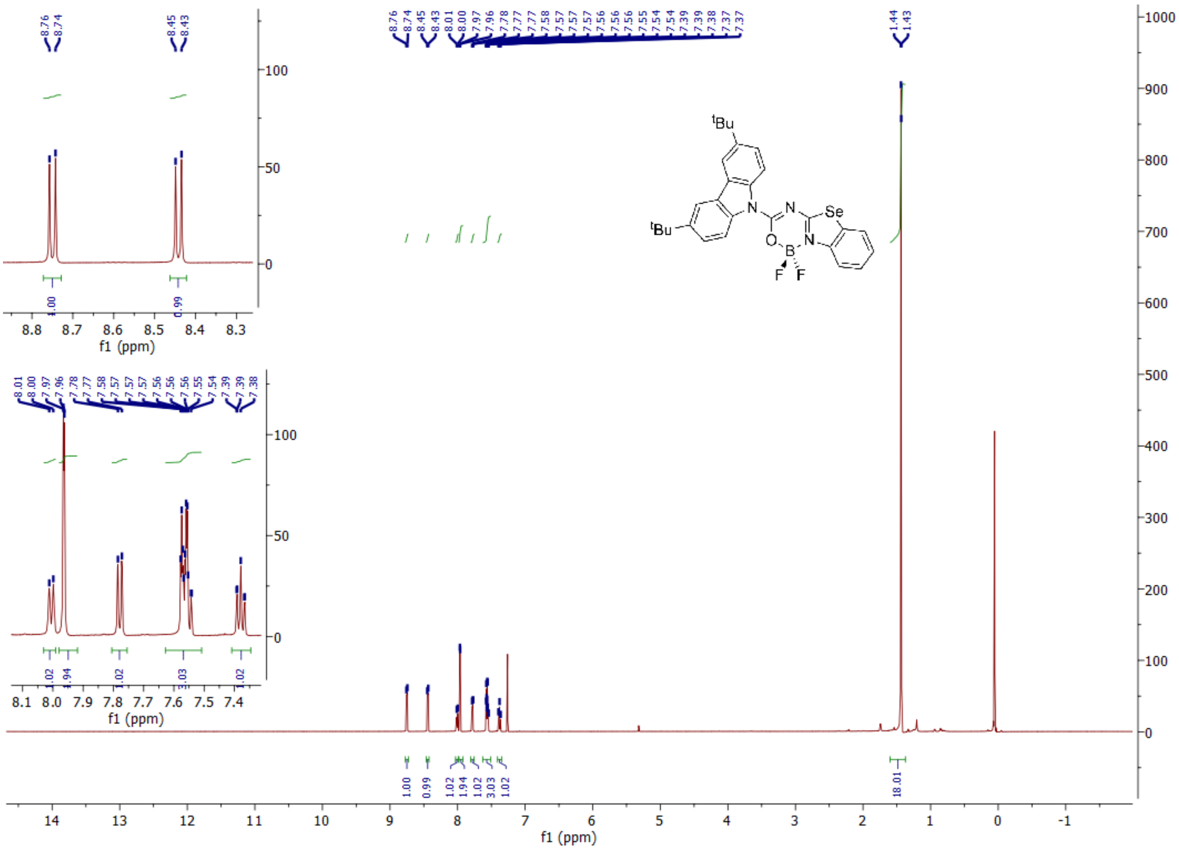

Figure S99. ^{1}H NMR (600 MHz, $CDCl_3$) spectrum of compound **3b** at -40°C.

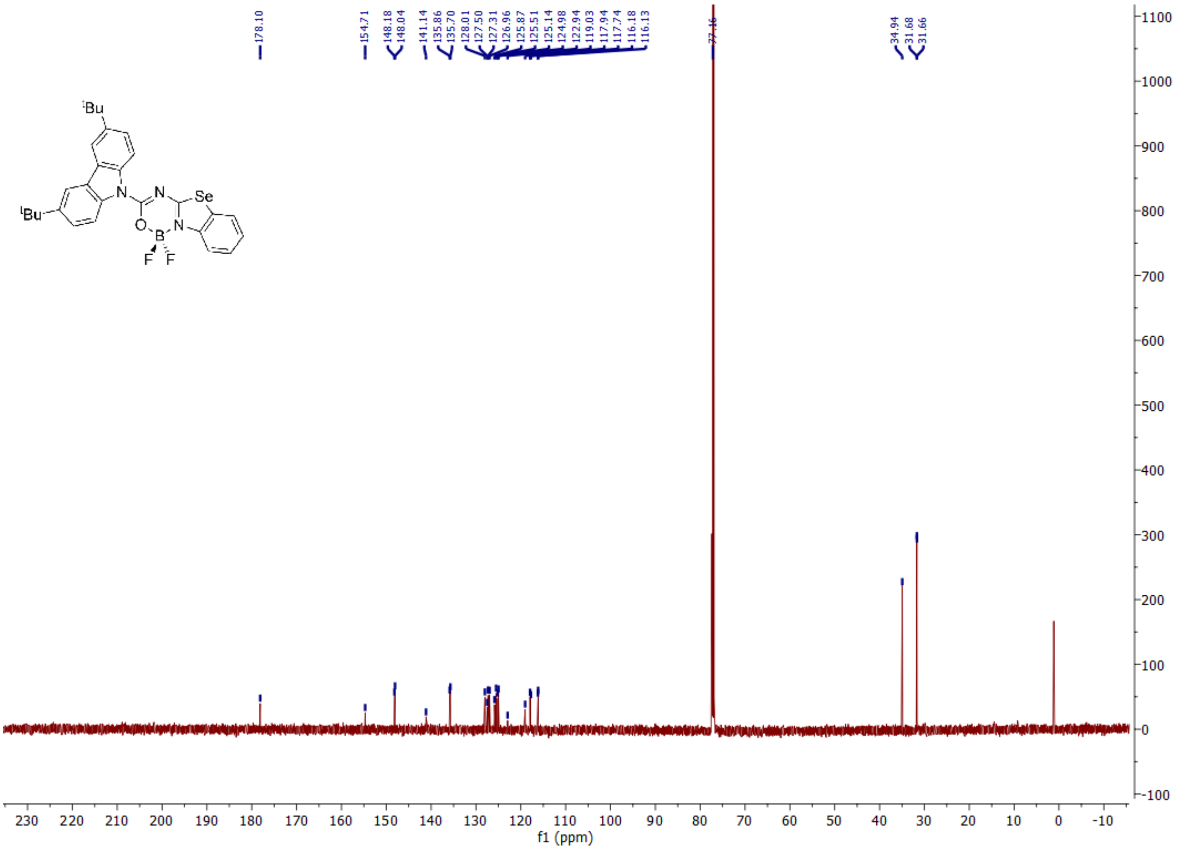

Figure S100. $^{13}C\{H\}$ NMR (150 MHz, $CDCl_3$) spectrum of compound **3b** at -40°C.

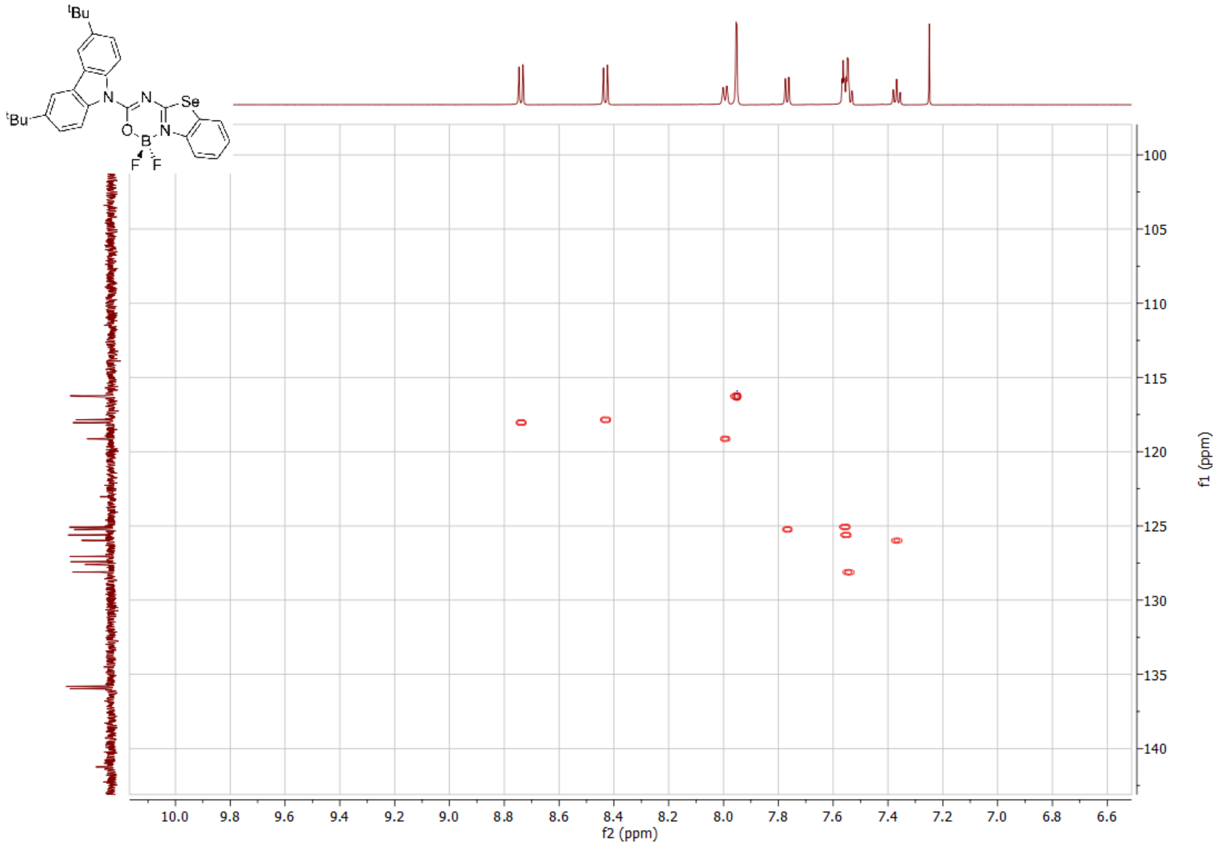

Figure S101. ^{1}H–^{13}C HSQC NMR (600 MHz, $CDCl_3$) spectrum of compound **3b** at -40°C.
